# The infection of mycovirus down regulates *Aa-milR13* to weaken the pathogenicity of the *Alternaria alternata* f. sp. *mali*

**DOI:** 10.3389/fpls.2025.1598183

**Published:** 2025-07-22

**Authors:** Jingyi Zhang, Chenjiao Li, Shaopeng You, Yuyao Feng, Mingyi Liu, Bo Li, Shutong Wang, Keqiang Cao, Yanan Wang

**Affiliations:** College of Plant Protection, Hebei Agricultural University, Baoding, Hebei, China

**Keywords:** *Alternaria alternata* f. sp. *mali*, mycovirus, milRNA, hypovirulence mechanism, interaction mechanism

## Abstract

This study explored the association between differentially expressed miRNA Aa-milR13 in *Alternaria alternata* f. sp. *mali* strains with and without virus infection, and the regulation of fungal host pathogenicity by viruses. MiRNA sequencing was conducted on a hypovirulent strain (QY21) with compound infection of Alternaria alternata chrysovirus 1 (AaCV1) and Alternaria alternata magoulivirus 1 (AaMV1), a hypovirulent strain (QY21-C1) with single AaMV1 infection, and a virus-free strain (QY21-C2). Fourteen miRNAs with significant differential expression were identified. Aa-milR13 was significantly downregulated in virus-infected strains and validated by RT-qPCR. Structural analysis of the Aa-milR13 sequence identified that the mature Aa-milR13 is located at the 5’ end of its precursor stem-loop structure. Knockout of Aa-milR13 in QY21-C2 resulted in slower hyphal growth, darker colony color, and reduced pathogenicity, resembling virus-infected strains. Conversely, overexpression of Aa-milR13 led to accelerated hyphal growth, lighter colony color, and significantly enhanced pathogenicity.Three target genes of Aa-milR13, subtilisin-like protein (AaSLP,cinr CC77DRAFT_1100266), DUF431-domain-containing protein (DUF431, CC77DRAFT_1022347), and high-affinity nitrate transporter NrtB (NrtB, CC77DRAFT_1056077), were predicted and confirmed by RT-qPCR to be negatively correlated with Aa-milR13 expression. Bioinformatics analysis indicated that Aa-milR13 targets the CDS regions of the potential target genes through cleavage, with Watson-Crick pairing of AU, CG, wobble pairing of GU, and various pairing patterns such as AA, AC, AG, GG, UU, UC, etc. This study suggests that fungal virus infection downregulates Aa-milR13, upregulating its targets, potentially contributing to reduced fungal pathogenicity. This is the first report of small RNAs acting as an intermediate bridge in the regulation of fungi virulence by fungal viruses. The research results lay the foundation for elucidating the mechanism of small RNA-involved regulation of fungi hypovirulence by fungal viruses and provide theoretical support for the use of fungal viruses in the control of crop fungal diseases.

## 1 Introduction

Alternaria blotch of apple, caused by *Alternaria alternata* f. sp. *mali*, is one of the major leaf diseases of apples (Cao et al., 2024). Chemical control is primarily used in production to manage this disease. However, the long-term and excessive use of chemicals may pose potential risks to human health and the ecological environment (Liu et al., 2022). Therefore, biological control, as a green and sustainable prevention and control measure, is gradually gaining attention. Studies have shown that mycoviruses can attenuate the pathogenicity of their fungal hosts and, in some cases, convert fungal hosts into plant endophytes, thereby promoting plant growth and disease resistance. Mycoviruses have emerged as a potential biological control measure (Kotta-Loizou, 2021). Revealing the mechanisms by which mycoviruses regulate host virulence will facilitate their application in crop disease control.

In recent years, progress has been made in studying the hypovirulence mechanisms of mycoviruses (Kotta-Loizou, 2021). From a cellular biology perspective, studies have found that mycovirus infection may be accompanied by abnormal morphological development of host hyphae, reduced conidia production, abnormal pigmentation, and other growth phenotypic changes, as well as physiological and metabolic abnormalities such as interference with the production of host cell wall-degrading enzymes (e.g., cutin) and impeded synthesis of pathogenic mycotoxins (e.g., Alternariol, AOH and Altersolanol A) (Li et al., 2022a; Liu et al., 2022). At the molecular level, transcriptome sequencing technology has been used to analyze the effects of virus infection on fungal host mRNA. As an example, stemphylium lycopersici alternavirus 1 (SlAV1) infection in *Stemphylium lycopersici* was found to significantly enrich genes associated with ribosome biogenesis, spliceosome RNA transport, and DNA replication pathways, based on transcriptome sequencing data (Cao et al., 2022). Small RNA sequencing technology has revealed significant differences in the expression of host small RNAs (sRNAs) before and after mycovirus infection (Iwakawa and Tomari, 2022; Piombo et al., 2022).

Fungal sRNAs play a critical role in the interaction between plant pathogens and their hosts, influencing both virulence and host defense. These sRNAs, including miRNA-like (milRNA) and siRNA, can be processed by enzymes such as Dicer and Argonaute (AGO) proteins to form miRISC complexes, which target host genes for cleavage or translational repression (Kobayashi and Tomari, 2016; Prinz et al., 2021). Studies have revealed that fungal sRNAs are involved in regulating their own gene expression, influencing developmental processes and virulence. For instance, *Fusarium oxysporum* f. sp. *cubense* produces miRNAs that target glycosyl hydrolase genes, promoting pathogenicity (Li et al., 2022b). Fungal sRNAs can also hijack the host’s RNA interference (RNAi) machinery to suppress plant immunity. For example, *Botrytis cinerea* secretes sRNAs that enter plant cells and silence defense genes (He et al., 2023). The ability of sRNAs to mediate these interactions has opened new avenues for biotechnological applications, such as the development of environmentally friendly RNA-based biocontrol agents.

Host-derived small RNAs induced and activated by animal and plant viruses play important roles in host-virus interactions (Trobaugh and Klimstra, 2017; Wang et al., 2022), but studies on this aspect of mycoviruses have not been reported. This study aims to reveal the mechanism by which small RNAs, as intermediate bridges, participate in the regulation of fungal host virulence by mycoviruses, providing theoretical support for the use of mycoviruses in the control of plant fungal diseases. In previous work, we obtained *A. alternata* f. sp. *mali* strains QY21 carrying mycoviruses Alternaria alternata chrysovirus 1 (AaCV1) and Alternaria alternata magoulivirus 1 (AaMV1). By virus-free culture, we obtained the virus-free strain QY21-C2 and the AaMV1 singly infected strain QY21-C1, demonstrating that infection by these two viruses is the primary cause of the decline in pathogenicity of *A. alternata* f. sp. *mali* strains. These are the first viruses found in *A. alternata* f. sp. *mali* that significantly reduce the pathogenicity of their fungal hosts, leading to host colony shrinkage, abnormal hyphal morphology, reduced sporulation, growth inhibition, abnormal pigmentation, and particularly, reduced synthesis of the pathogenicity-related factor AOH and decreased pathogenicity (Li et al., 2022a). This study focuses on the endogenous small RNAs activated by mycoviruses in *A. alternata* f. sp. *mali* and investigates their functions and mechanisms during the decline of fungal host virulence.

## 2 Materials and methods

### 2.1 *Alternaria alternata* f. sp. *mali* strains

The compound infection strain QY21 of *A. alternata* f. sp. *mali* by AaCV1 and AaMV1, as well as the AaMV1 single infection strain QY21-C1 and virus-free strain QY21-C2 obtained through detoxification of QY21, are preserved in Research Laboratory of Plant Disease Epidemiology and Integrated Pest Management of Hebei Agricultural University.

### 2.2 Small RNA isolation and sequencing

Three strains, QY21, QY21-C1, and QY21-C2, were inoculated separately onto potato dextrose agar (PDA) medium lined with cellophane at the bottom. After 72 hours of culture at 25°C, mycelia were scraped using a sterile disposable mycelium scraper, placed in RNA- and DNA-free centrifuge tubes, and rapidly frozen in liquid nitrogen. milRNA was extracted using the miRcute milRNA isolation kit (Tiangen, Beijing, China). Three biological replicates were set up for each strain, and milRNA sequencing was entrusted to Shanghai Majorbio Pharmaceutical Technology Co., Ltd (Shanghai, China). Reference species is *A. alternata* SRC1lrK2f. Reference genome version is Altal1 (Acc No: GCA_001642055). Specific steps were conducted, including extracting total RNA from the samples, measuring the concentration and purity of the extracted RNA using Nanodrop2000 (Thermo, MA, USA), assessing RNA integrity via agarose gel electrophoresis, and determining the RQN value using Agilent5300. Small RNA libraries were constructed using the QIAseq miRNA Library Kit (96) (Qiagen, Germany) according to the manufacturer’s protocol. The requirements for cDNA library construction are: total RNA amount of 1 μg, concentration ≥ 50 ng/μL, RQN > 7, and OD_260_/OD_280_ between 1.8 and 2.2. Adapter sequences were ligated to the 3’ and 5’ ends, respectively. Under the action of reverse transcriptase, a single strand of cDNA was reverse-synthesized using the RNA with ligated adapters as a template and random primers. This was followed by second-strand synthesis to form a stable double-strand structure. PCR amplification (11-12 cycles) was performed using sequencing primers to enrich the library. Target fragments were recovered by gel electrophoresis (6% Novex TBE PAGE gel, 1.0 mm, 10 well). The enriched 18-32 nt small RNA fragments were sequenced on the NovaSeq X Plus platform (Illumina Inc., San Diego, CA, USA).

### 2.3 Processing of high-throughput sequencing data

The sequencing data was aligned with the reference genome sequence Altal1 to identify contamination or confusion in the samples. Low-quality data was removed before data analysis (Jiang et al., 2011). Fastp (https://github.com/OpenGene/fastp) was used for quality filtering and adaptor trimming. For the raw reads obtained from small RNA sequencing, small RNA fragments <18 nts and >30 nts were discarded, reads with an undetermined base ratio >10% were removed, reads with 3’ and 5’ adapter contamination, no inserted sequences, and single-copy sequences were removed, and reads containing polyA were discarded to obtain clean reads. After data quality control, the quality-controlled data was again statistically analyzed and quality assessed, including base quality, error rate, and content distribution statistics. This resulted in high-quality quality-controlled data (clean data) to ensure the accuracy of subsequent analysis results.

### 2.4 Analysis of small RNA sequencing data

The quality-controlled raw data was aligned with the reference genome to obtain valid data (mapped reads) for subsequent analysis. TopHat2 (http://tophat.cbcb.umd.edu/) (Grabherr et al., 2011) and HISAT2 (http://ccb.jhu.edu/software/hisat2/index.shtml) (Smith-Unna et al., 2016) software were used to align the quality-controlled raw data with the reference genome. The distribution of raw data with a mapping rate higher than 65% (Total Mapped Reads) on the reference sequence was analyzed. Based on the existing reference genome, software Cufflinks (http://cole-trapnelllab.github.io/cufflinks/) (Li and Godzik, 2006) and StringTie (http://ccb.jhu.edu/software/stringtie/) (Bray et al., 2016) were used to assemble and splice the mapped reads and compare them with known transcripts. The reads aligned to the reference genome Altal1 were compared with the miRbase (Version 22.1) and Rfam (Version 14.6) databases to obtain known milRNA annotation information. MiReap software was used to predict new milRNAs for reads without annotation information using the aforementioned reference genome. DESeq2 (Zhou et al., 2014) was used to perform differential expression analysis on all small RNA loci in the nine strains, normalizing the raw read counts of each small RNA in each sample to the total read counts in that sample. The fold change (FC) between virus-infected and uninfected samples was calculated. The Benjamini and Hochberg method was used to correct the *P*-values obtained from the Wald test multiple times to obtain adjusted *P*-values (Padjust). Statistically supported differentially expressed milRNAs were retrieved (padjust ≤0.05, up-regulated when log_2_(FC) ≥0.6 or down-regulated when log_2_(FC) ≤-0.6). The RNAfold web server (http://rna.tbi.univie.ac.at/cgi-bin/RNAfold.cgi) was used to predict the secondary structure of milRNA precursors, with base pairing probabilities used for coloring. The online website predictprotein (https://predictprotein.org/) was used to predict the secondary structure of proteins. Protein tertiary structure analysis was performed according to SWISS-MODEL (https://swissmodel.expasy.org/interactive) was used for target gene prediction.

### 2.5 Extraction of mycelial DNA and RNA

The fungal genomic DNA rapid extraction kit (Sangon, Shanghai, China) was used to extract DNA from *A. alternata* f. sp. *mali* strains. Total RNA was extracted from the three strains, QY21, QY21-C1, and QY21-C2, using Trizol reagent (TransGen, Beijing, China). The integrity of the total RNA was detected using 1.0% agarose gel electrophoresis, and the concentration and purity of the nucleic acids were determined using the Eppendorf Biospectrometer^®^ basic UV-Vis fluorospectrophotometer (Eppendorf, HH, Germany).

### 2.6 Real-time fluorescent quantitative PCR

The RT-qPCR detection of key milRNAs was performed using 2×FastFire qPCR PreMix (Tiangen, Beijing, China) on a 7500 quantitative PCR system (ABI, CA, USA). The primers used were: pmilR13q-F: GCGCGTGTTTATCTTCCAGA, and pmilR13q-R: AGTGCAGGGTCCGAGGTATT. U6 (U6 small nuclear RNA) was used as the housekeeping gene for milRNAs, while the *Tubulin* gene served as the housekeeping gene for the target genes. The relative expression levels were calculated using the 2^-ΔΔCt^ method. The quantitative primers for the potential target genes were: pAaSLPq-F: ATGGTGACGGTGTGGAGGATG, pAaSLPq-R: CGCAATGTCGGGCTGGAAAC; pAaDUF431q-F: AGTTCCTGCTGTCTTCCGTACC, pAaDUF431q-R: TCTGTCCGCATAGATTTCTTCAATACC; pAaNrtBq-F: GTGCTACCGTCGTGTCCAATG, pAaNrtBq-R: TCGCAGGCTTGTCGTCCAG.

### 2.7 RT-PCR verification of the presence of the precursor of Aa-milR13 in *Alternaria alternata* f. sp. *mali*

To further validate that Aa-MIL1R13 serves as the functional precursor encoding mature Aa-milR13 and confirm its stability across fungal generations, we conducted a detailed precursor verification workflow. First, based on the miRNA sequencing-derived precursor sequences of Aa-milR13, we designed strain-specific forward (pmilR13-F: 5’-TGTTTATCTTCAGACTGTTGTTGT-3’) and reverse (pmilR13-R: 5’-AGTGTCAATCCACCAACACTGA-3’) primers using Premier 5.0 software. These primers were synthesized by Sangon Biotech (Shanghai) to ensure high specificity. Total RNA was extracted from mycelia of three *A. alternata* strains (QY2, QY21-C1, QY21-C2) subcultured for 3 generations using a standardized protocol (method 2.5). cDNA synthesis was performed with HiScript II Enzyme Mix (Vazyme) following the kit’s instructions. PCR amplification was conducted using a 25 μL reaction system with thermal cycling conditions: pre-denaturation at 95°C for 3 min, 35 cycles of 94°C (30 s), 58°C (30 s), and 72°C (30 s), followed by a final extension at 72°C for 5 min. PCR products were analyzed via 1.5% agarose gel electrophoresis and Sanger sequencing.

### 2.8 Generation of Aa-milR13 mutant or overexpression transformants in *Alternaria alternata* f. sp. *mali*

#### 2.8.1 Construction of Aa-milR13 knockout vector

The precursor sequence of Aa-milR13 in *A. alternata* was obtained through milRNA sequencing. BLAST alignment was performed using the NCBI database (http://www.mirbase.org/). Based on the precursor sequence, 200 base pairs (bp) were extended towards the 5’ and 3’ ends, respectively. These extended precursor sequences were then located in the genome, and adjacent sequences of 1000 bp upstream and downstream were obtained. Based on these sequence information, specific primer pairs were designed for PCR amplification (upstream amplification primers: pΔmilR13-1-F: AAAAACGACGGCCAGTGAATTCTTTGTCCATTGAAGCGCATCG, pΔmilR13-1-R: TGCTCCTTCAAAAGGACCTTGCTGTCAAAGCTGC; downstream amplification primers: pΔmilR13-2-F: CCCTGGGTTCGCAAAAGATAAAAAGACAGTTAGACTGTTGTAAGGAAAAAAG, pΔmilR13-2-R: CAGGTCGACTCTAGAGGATCCCGTTATCGTACCTGACAGCCTG). PCR amplification was performed using hygromycin B as the resistance screen. Primers for the hygromycin B resistance gene with homologous parts upstream and downstream of the Aa-milR13 precursor were designed (phyg-F: CAAGGTCCTTTGAAGGAGCATTTTTTGGGC, phyg-R: TTATCTTTGCGAACCCAGGG) and amplified using the pUChyg plasmid as a template. The amplification products were ligated into the pUC19 vector, transformed, and verified by colony PCR. Sequencing was performed by Sangon Biotech (Shanghai, China). to confirm positive transformants.

#### 2.8.2 Construction of Aa-milR13 overexpression vector

To construct the overexpression vector for Aa-milR13 in *A. alternata*, we first double-digested the pSilent-1 plasmid with FlyCut^®^ *Xho*I and FlyCut^®^ *Hind*III (Thermo Fisher Scientific) to generate compatible ends, followed by gel extraction (Qiagen) to purify the linearized vector. Using genomic DNA from *A. alternata* QY21-C2 as a template, the Aa-milR13 overexpression precursor fragment was amplified with primers pOE-milR13-F (5’-atcgataccgtcgac*ctcgag*TCTTGGTACCAATGTCGTTGCTA-3’) and pOE-milR13-R (5’-cctgtatcctccagc*aagctt*TGTGAAGTTGAATTCAATCAGTATCCG-3’), where lowercase letters denote vector backbone sequences and italicized lowercase letters mark restriction enzyme recognition sites (*Xho*I and *Hind*III). The PCR product was ligated to the linearized pSilent-1 vector using the Vazyme Seamless Cloning Kit, and the ligation mixture was transformed into *E. coli* DH5α(Takara Bio Inc., Beijing) via heat shock (42°C for 45 s) with recovery in SOC medium. Positive transformants were selected on LB agar with 50 μg/mL hygromycin B, validated by colony PCR with the same primers, and confirmed by Sanger sequencing (Sangon Biotech, Shanghai). Verified recombinant plasmids were extracted for subsequent fungal transformation, ensuring high-purity DNA for downstream experiments.

#### 2.8.3 PEG-mediated genetic transformation of *Alternaria alternata* f. sp. *mali* protoplasts

Protoplasts of the *A. alternata* strain QY21-C2 were prepared following a modified method of Zhou et al. (2014): mycelia were digested with lytic enzymes to generate protoplasts, which were filtered through a 40-μm nylon mesh and washed three times with sterile distilled water to remove enzyme residues. The protoplast concentration was adjusted to 1×10^7^ cells/mL using a hemocytometer. Enriched fragments of the Aa-milR13 precursor knockout vector and overexpression vector were then subjected to PEG-mediated protoplast transformation: equal volumes of the protoplast suspension (1×10^7^ cells/mL) and linearized DNA fragments were mixed gently in a 50-mL RNase & DNase free aseptic centrifuge tube, followed by the addition of PEG 4000 (Sigma-Aldrich) to a final concentration of 40% (w/v) and incubation at room temperature for 30 min to facilitate DNA uptake. After transformation, 1 mL of potato dextrose broth (PDB) containing 0.6 M sorbitol was added to stabilize the protoplasts, which were incubated at 28°C for 24 h to allow recovery and expression of the selectable marker (Zhou et al., 2014).

For knockout mutants, stable transformants growing on PDA plates supplemented with 100 μg/mL hygromycin B were verified by PCR using primers targeting the hygromycin resistance gene (phyg: phyg-F/phyg-R), Aa-milR13 precursor (pfmilR13-F: 5’-TCTTGGTACCAATGTCGTTGCT-3’/pfmilR13-R: 5’-TGTGAAGTTGAATTCAATCAGTATCC-3’), and Aa-milR13 precursor knockout vector backbone (pΔmilR13-1-F/pΔmilR13-2-R). RT-qPCR with primers pfmilR13-qF (5’-CGAAACCGTATAATCTCA-3’) and pfmilR13-qR (5’-TACCAAGTATGGCAACC-3’) and β-tubulin as the reference gene further confirmed downregulation of the Aa-milR13 precursor in these mutants. For overexpression mutants, genomic DNA was extracted from stable transformants growing on hygromycin-containing PDA plates, and PCR was performed using the overexpression fragment amplification primers pOE-milR-F/R to verify vector integration. RT-qPCR with the primers (pfmilR13-qF/pfmilR13-qR) was also used to validate Aa-milR13 overexpression.

Stable overexpression and knockout mutants were collected by centrifugation (5,000 × g, 5 min), resuspended in 50% glycerol (v/v) in TE buffer (10 mM Tris-HCl, 1 mM EDTA, pH 8.0), and stored at –80°C for subsequent experiments.

### 2.9 Phenotype and pathogenicity assay of Aa-milR13 knockout and overexpression strains

To evaluate the phenotypic and pathogenic characteristics of Aa-milR13 knockout and overexpression strains, two sets of experiments were conducted: colony morphology observation and detached plant tissue inoculation assays.

#### 2.9.1 Colony morphology observation

Five-millimeter mycelial plugs from Aa-milR13 knockout mutants, overexpression strains, wild-type QY21, and QY21-C2 (control) were aseptically placed at the center of PDA plates. Plates were incubated upside down at 25°C in the dark for 7 days. Each treatment was repeated three times, with three replicate plates per strain. Daily observations were made to record colony morphology and measure colony diameter.

#### 2.9.2 Detached leaf and fruit pathogenicity assay

Uniformly grown crabapple (*Malus* sp*ectabilis*) leaves and ‘Tianhong 2’ apple (*M. domestica*) fruits with consistent maturity and health status were selected. Tissues were rinsed thoroughly with deionized water to remove surface contaminants, then placed on sterile trays lined with double-layered, pre-soaked filter paper. All tissues were injured uniformly using a sterile inoculation needle (2 mm deep, 1 mm wide) to create standardized wounds. A 5-mm mycelial plug from each strain (Aa-milR13 knockout, overexpression, QY21, or QY21-C2) was placed on the wounded area with the mycelial surface facing the leaf or fruit tissue. Trays were sealed with double-layer plastic wrap to maintain high humidity during incubation.

Inoculated tissues were incubated at 25°C with 80% relative humidity for 14 days. Disease progression was assessed by tracing the margins of lesions onto red grid paper (1 cm × 1 cm squares) at 7-day intervals. Lesion areas were calculated by counting the number of grid squares covered by necrotic tissue and converting to square centimeters (cm²). Each treatment included three biological replicates, with three tissue samples per replicate.

## 3 Results and analysis

### 3.1 Differences in milRNA expression and base preference in strains of *Alternaria alternata* f. sp. *mali* with different virus

To elucidate the impact of virus infection on differential milRNA expression in *A. alternata*, we constructed milRNA libraries for the AaCV1 and AaMV1 co-infected strain QY21, the AaMV1 singly infected strain QY21-C1, and the virus-free strain QY21-C2, and performed deep sequencing. The results are shown in **Table 1**. The average raw reads for the three strain libraries were 10,341,053, 12,478,073, and 11,684,185, respectively. After removing contaminants and low-quality sequences, the average clean reads obtained for the libraries were 7,899,450, 8,910,066, and 8,049,814, respectively. The average error rates of the sequenced bases were all below 0.1%, and both Q20 and Q30 were above 95%, indicating good library construction quality and sequencing quality, and reliable data.

**Table 1:** Average sequencing data of small RNA from different virus-infected strains of *Alternaria alternata* f. sp. *mali*.

Sample	Raw reads	Raw bases	Clean reads	Clean bases	Error rate (%)	Q20 (%)	Q30 (%)	GC content (%)
QY21-C2	11684185	876313900	8049814	210127829	0.0234	98.55	95.89	48.82
QY21-C1	12478073	935855525	8910066	213766602	0.0226	98.95	96.68	46.29
QY21	10341053	775579000	7899450	210066494	0.0226	98.98	96.68	50.68

**Figure 1:**
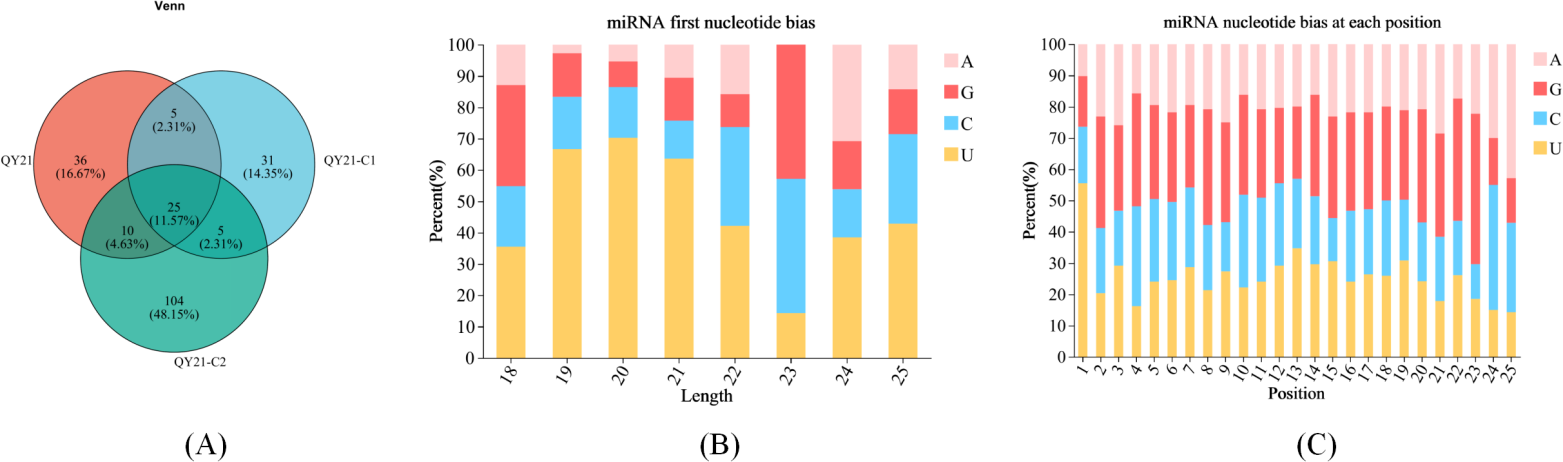
Base preference of milRNAs in strains of *Alternaria alternata* f. sp. *mali* with different virus types. **(A)** Venn analysis among samples; **(B)** Base preference of miRNAs of different lengths; **(C)** Base preference at different positions of miRNAs.

The reads of the reference genome were aligned with the miRBase and Rfam databases to obtain annotation information for known milRNAs. The miReap software was used to predict new milRNAs for reads without annotation information. Venn analysis of all milRNAs among samples was performed, and the results are shown in **Figure 1A**. Twenty-five milRNAs were expressed in all three strains, accounting for 11.25%. There were 36 specific milRNAs in QY21, accounting for 16.67%, 31 specific milRNAs in QY21-C1, accounting for 14.35%, and 104 specific milRNAs in QY21-C2, accounting for 48.15%. This suggests that virus infection can cause a decrease in the number of milRNAs. In addition, we analyzed the base preference of milRNAs in the three strains, and the results are shown in **Figures 1B, C**. Except for the first base of 23 nt milRNAs preferring G, milRNAs of other lengths preferred U. We also found certain preferences at other sites, such as a general lack of U at positions 2 to 4, and a preference for A at position 25.

### 3.2 Identification of differentially expressed milRNA in strains of *Alternaria alternata* f. sp. *mali* with different virus

**Figure 2:**
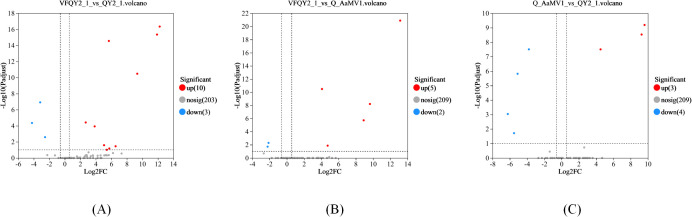
Volcano plots of differential endogenous milRNA expression in *Alternaria alternata* f. sp. *mali* strains with different virus infection statuses. **(A)** Volcano plot showing the difference in miRNA expression between QY21-C2 and QY21; **(B)** Volcano plot showing the difference in miRNA expression between QY21-C2 and QY21-C1; **(C)** Volcano plot showing the difference in miRNA expression between QY21-C1 and QY21.

**Figure 3:**
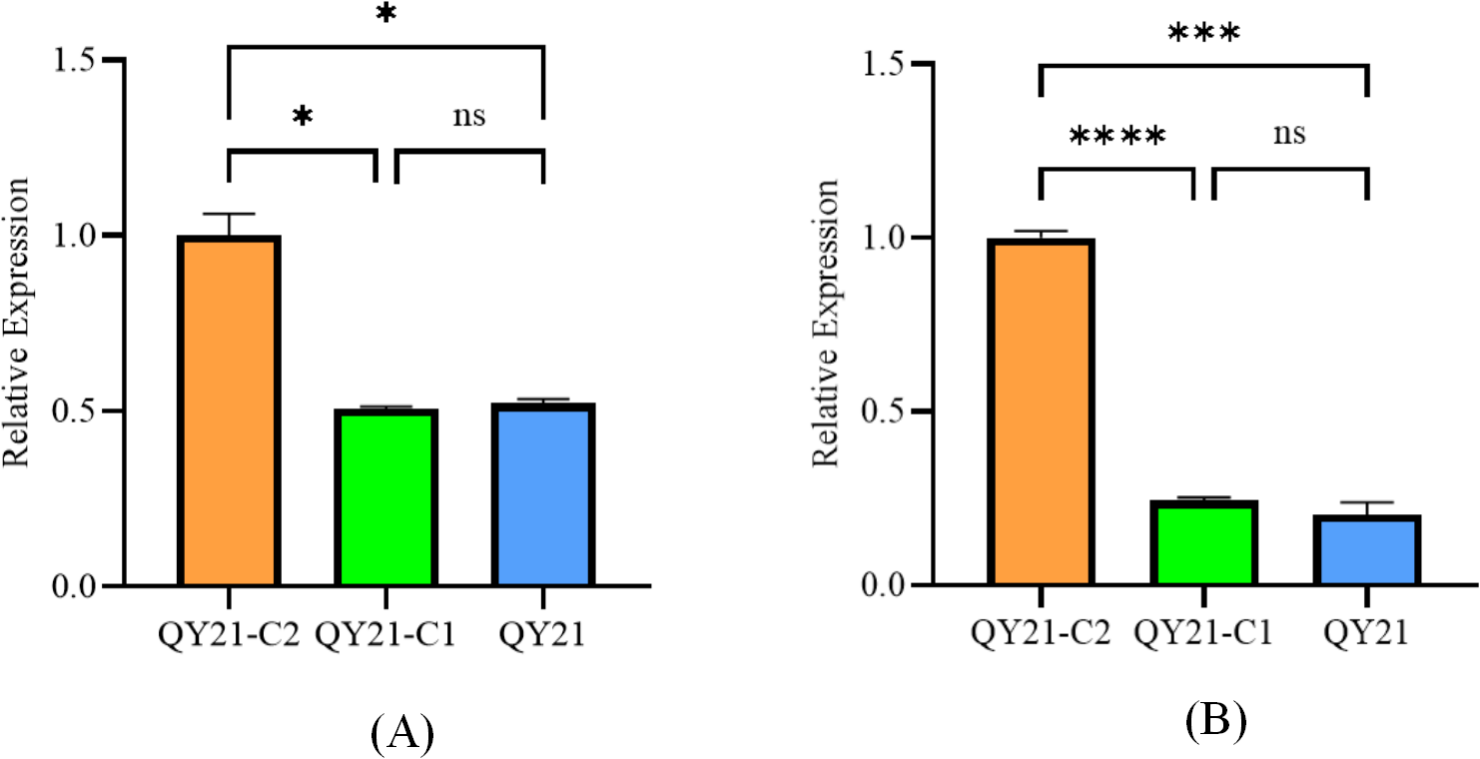
Effects of Aa-milR13 on the pathogenicity of *Alternaria alternata* f. sp. *mali* strains (detached apple fruits) (14 days). **(A)** Symptoms on detached on detached apple (*Malus domestica*) fruits; **(B)** The corresponding box plot represents the lesion area (cm²) for each treatment. The box plot displays the median (central line), interquartile range (IQR, box edges), and the range of the data (whiskers). Statistical significance between different strains was evaluated using the ordinary one-way ANOVA test (non-parametric, *P*< 0.0001). Different letters above the boxes indicate statistically significant differences (*P*< 0.05). Data are presented as the mean ± standard error of the mean (SEM) from three biological replicates. QY21-C2: Virus-free strain; ΔQYC2-milR13: Aa-milR13 knockout mutant strain; OE-milR13: Aa-milR13 overexpression strain; QY21: Virus-coninfection strain. Symbols, *, **, ***, and **** denote statistical significance at the p<0.05, p<0.01, p<0.001, and p<0.0001 levels, respectively.

Using DESeq2, we analyzed the differential expression of milRNA in strains with different virus types. The obtained *P*-values were corrected for multiple testing using the Benjamini and Hochberg method. The results, shown in **Figure 2** and **Table 2**, revealed a total of 14 significantly differentially expressed milRNA. The largest number of significantly differentially expressed milRNA was found between the virus-free strain QY21-C2 and the co-infected strain QY21. Among the two virus-taking strains QY21 and QY21-C1, and the virus-free strain QY21-C2, there were two milRNA, Aa-milR87 (KV441478_174387) and Aa-milR13 (KV441481_212713), that showed significant differences in expression between the virus-free strain and the two virus-taking strains, but not between the two virus-taking strains themselves. Quantitative analysis of these two milRNA, shown in **Figure 3**, confirmed the sequencing results, with Aa-milR13 exhibiting a more significant difference in expression. Therefore, Aa-milR13 was selected for further investigation.

**Table 2:** Differential expression of endogenous milRNA in *Alternaria alternata* f. sp. *mali* strains with different virus infection.

milRNA ID	QY21/QY21-C2			QY21-C1/QY21-C2			QY21/QY21-C1		
	Log_2_FC	Padjust	Significant/ Regulate	Log_2_FC	Padjust	Significant/ Regulate	Log_2_FC	Padjust	Significant/ Regulate
KV441486_262192	5.05	2.59E-02	yes|up	3.62	9.19E-01	no|up	1.43	1.00E+00	no|up
KV441470_40766	5.67	2.76E-15	yes|up	1.17	1.00E+00	no|up	4.50	3.11E-08	yes|up
KV441471_51702	3.85	1.18E-04	yes|up	1.24	1.00E+00	no|up	2.61	1.88E-01	no|up
KV441478_174387	-3.18	1.21E-07	yes|down	-2.12	5.10E-03	yes|down	-1.06	1.00E+00	no|down
KV441469_16614	3.06	1.99E-01	no|up	4.73	1.34E-02	yes|up	-1.67	1.00E+00	no|down
KV441487_271159	-4.23	4.40E-05	yes|down	0.87	1.00E+00	no|up	-5.11	1.51E-06	yes|down
mtr-miR5293	6.54	3.51E-02	yes|up	3.58	1.00E+00	no|up	2.95	1.00E+00	no|up
KV441481_212713	-2.55	2.59E-03	yes|down	-2.23	1.92E-02	yes|down	-0.32	1.00E+00	no|down
KV441498_333084	11.88	4.41E-16	yes|up	0.00	1.00E+00	no|no_change	9.26	2.93E-09	yes|up
KV441487_265463	0.00	1.00E+00	no|no_change	8.89	1.89E-06	yes|up	-5.52	1.93E-02	yes|down
KV441492_305016	9.33	3.29E-11	yes|up	13.13	1.28E-21	yes|up	-3.80	3.09E-08	yes|down
KV441477_157230	12.22	4.46E-17	yes|up	0.00	1.00E+00	no|no_change	9.60	6.39E-10	yes|up
KV441490_292151	2.69	3.84E-05	yes|up	4.07	3.28E-11	yes|up	-1.38	3.66E-01	no|down
KV441490_291394	0.00	1.00E+00	no|no_change	9.62	6.22E-09	yes|up	-6.24	9.19E-04	yes|down

Log_2_FC (s1/s2): The base-2 logarithm of the fold change in miRNA expression between two samples (s2 serves as the control group). Padjust: The *P*-value adjusted for multiple hypothesis testing.; “yes” indicates significant difference in expression level; “no” indicates insignificant difference in expression level; “up” means up-regulation of expression level in the latter compared to the former; “down” means down-regulation of expression level in the latter compared to the former.

**Figure 4:**
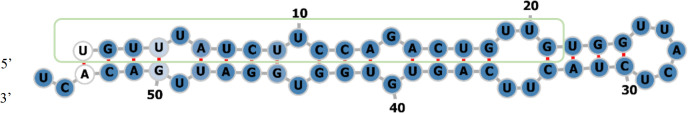
Precursor stem-loop structure of milRNA Aa-milR13 in *Alternaria alternata* f. sp. *mali*.

The stem-loop structure of Aa-milR13 was predicted using the RNAfold web server. The result, shown in **Figure 4**, indicated that the mature Aa-milR13 is located on the 5’ arm of the stem-loop structure, suggesting a highly conserved position of Aa-milR13 on its precursor gene. The thermodynamic predictions showed that the free energy of the thermodynamic system was -23.32 kcal/mol, and the minimum free energy was -22.90 kcal/mol, with a small difference between the two and both in a relatively low energy state, indicating that the structure is thermodynamically stable. In addition, the frequency of the Minimum Free Energy (MFE) structure was 50.99%, and the ensemble dispersion was 1.01, indicating a relatively high frequency and a relatively low dispersion, which suggests that this structure dominates in the thermodynamic system and is relatively concentrated, further demonstrating the stability of the Aa-milR13 stem-loop structure.

### 3.3 Verification of the presence of the precursor of Aa-milR13 in *Alternaria alternata* f. sp. *mali*

**Figure 5:**
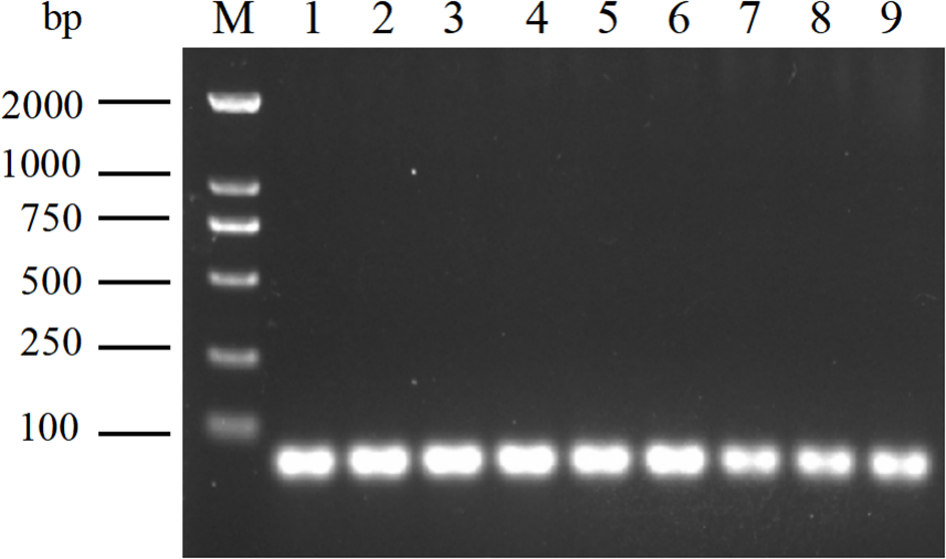
Amplification of Aa-milR13 precursors in *Alternaria alternata* f. sp. *mali* strains by RT-PCR. M: DL2000; 1~3: Subculture of 1-3 generations of virus-free strains; 4~6: Subculture of 1-3 generations of singly infected strains; 7~9: Subculture of 1-3 generations of mix-infected strains.

To further validate the stable presence of the Aa-milR13 precursor in *A. alternata* trains, three independent isolates (QY2, QY21-C1, and QY21-C2) were subcultured for 3 generations. RT-PCR amplification using strain-specific primers targeting the predicted Aa-milR13 precursor sequence produced a single prominent band of approximately 57 bp in all three subcultured strains (**Figure 5**), consistent with the predicted length of the Aa-milR13 precursor (57 bp). Sanger sequencing of the PCR products confirmed 100% sequence identity with the genomic sequence of the predicted Aa-milR13 precursor, with no detectable mutations or sequence divergence across generations. These results demonstrate that the Aa-milR13 precursor is stably maintained in *A. alternata* strains, confirming its role as the functional precursor encoding mature Aa-milR13.

### 3.4 Effects of knockout and overexpression of Aa-milR13 on *Alternaria alternata* f. sp. *mali* strains

**Figure 6:**
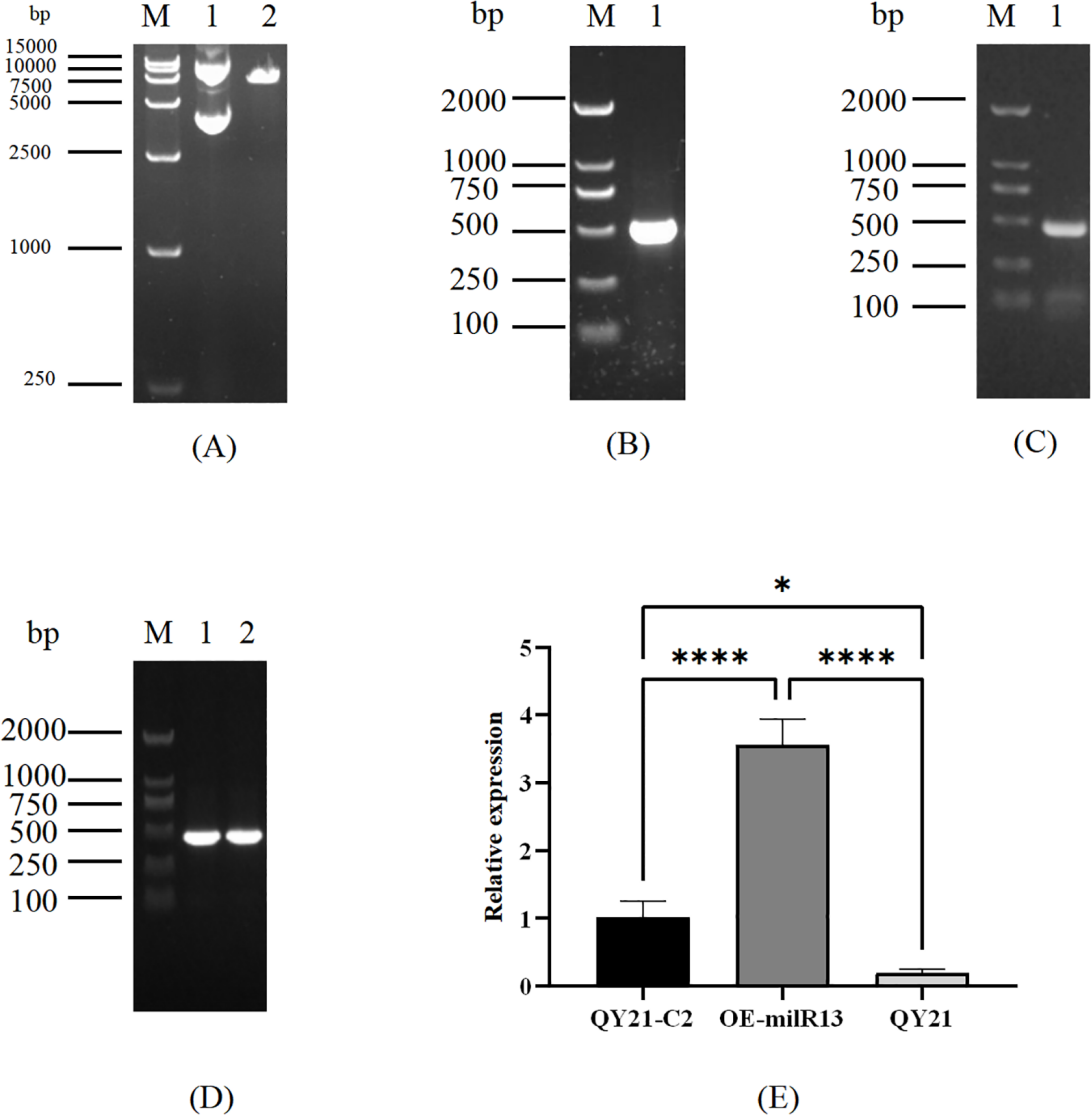
Obtaining of Aa-miLR13 knockout mutant in *Alternaria alternata* f. sp. *mali* strain QY21-C2. **(A)** Double enzyme digestion of vector pUC19 (M: DL5000; 1. pUC19 plasmid before *Eco*RI and *Bam*HI digestion; 2. pUC19 after *Eco*RI and *Bam*HI digestion); **(B)** Amplification of upstream and downstream fragments of Aa-milR13 precursor and hygromycin resistance gene HYG (M: DL5000; 1: Amplification of upstream fragment of Aa-milR13 precursor; 2: Amplification of hygromycin resistance gene HYG; 3: Amplification of downstream fragment of Aa-milR13 precursor); **(C)** PCR verification of Aa-milR13 precursor knockout vector (M: DL5000; 1. Amplification of Aa-milR13 precursor knockout vector using primers pΔmilR13-1-F and pΔmilR13-2-R); **(D)** Validation of Aa-milR13 knockout transformants (M: DL5000; 1: Amplification of hygromycin resistance gene within Aa-milR13 knockout transformant ΔQYC2-milR13 using primers hygF and hygR; 2: Amplification of Aa-milR13 precursor and 200 bp upstream and downstream within ΔQYC2-milR13 using primers pfmilR13-F and pfmilR13-R; 3: Amplification of Aa-milR13 precursor knockout vector within ΔQYC2-milR13 using primers pΔmilR13-1-F and pΔmilR13-2-R; 4: Amplification of Aa-milR13 precursor and 200 bp upstream and downstream within QY21-C2 using primers pfmilR13-F and pfmilR13-R; 5: Water control); **(E)** RT-qPCR verification of Aa-milR13 expression in knockout mutant ΔQYC2-milR13. Symbols ,* ,**,*** , and**** denote statistical significance at the p<0.05, p<0.01, p<0.001, and p<0.0001 levels, respectively.

**Figure 7:**
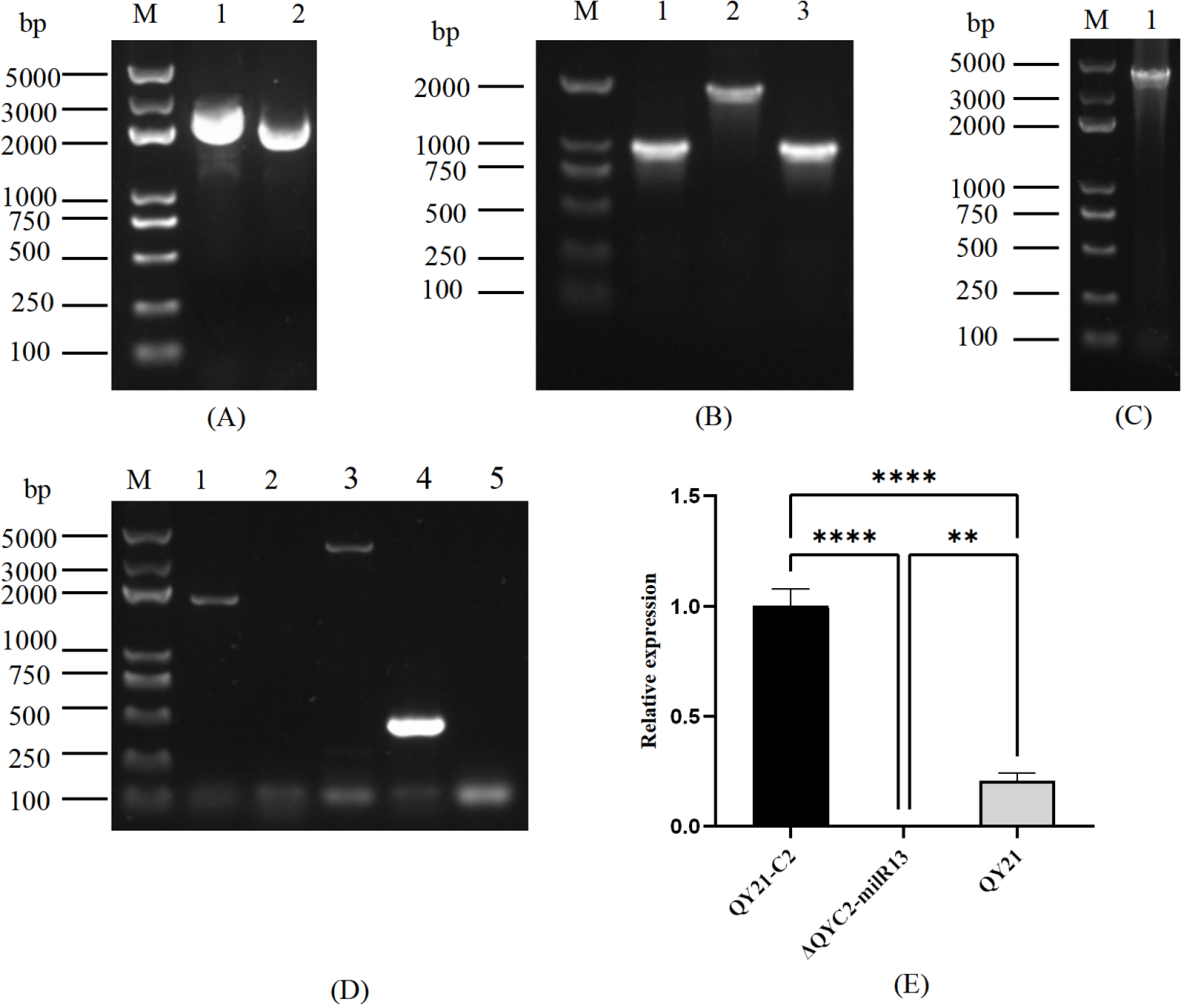
Obtaining of Aa-miLR13 precursor overexpression mutants in *Alternaria alternata* f. sp. *mali* strain QY21-C2. **(A)** Double enzyme digestion of vector pSilent-1 (M: DL15000; 1: pSilent-1 plasmid before *Xho*I and *Hind*III digestion; 2: pSilent-1 plasmid after *Xho*I and *Hind*III digestion); **(B)** PCR amplification of Aa-milR13 overexpression fragments (M: DL2000; 1: Aa-milR13 amplification fragment); **(C)** Colony PCR verification of the Aa-milR13 overexpression vector (M: DL2000; 1: Aa-milR13 detection fragment); **(D)** PCR Verification of overexpression fragments in Aa-milR13 overexpression strains (M: DL2000; 1-2: Aa-milR13 verification fragments); **(E)** RT-qPCR verification of Aa-milR13 overexpression strain OE-milR13. Symbols ,* ,**,*** , and**** denote statistical significance at the p<0.05, p<0.01, p<0.001, and p<0.0001 levels, respectively.

To elucidate the effect of Aa-milR13 on *A. alternata* strains, an Aa-milR13 knockout vector and overexpression vector were constructed (as shown in **Figures 6A–C**, **7A–C**). Knockout and overexpression transformants were obtained via PEG-mediated protoplast transformation. Stable genetically knocked-out mutant ΔQYC2-milR13 and overexpression mutant OE-milR13 were selected through hygromycin resistance screening. PCR and RT-qPCR identification results are shown in **Figures 6D, E**, **7D, E**.

**Figure 8:**
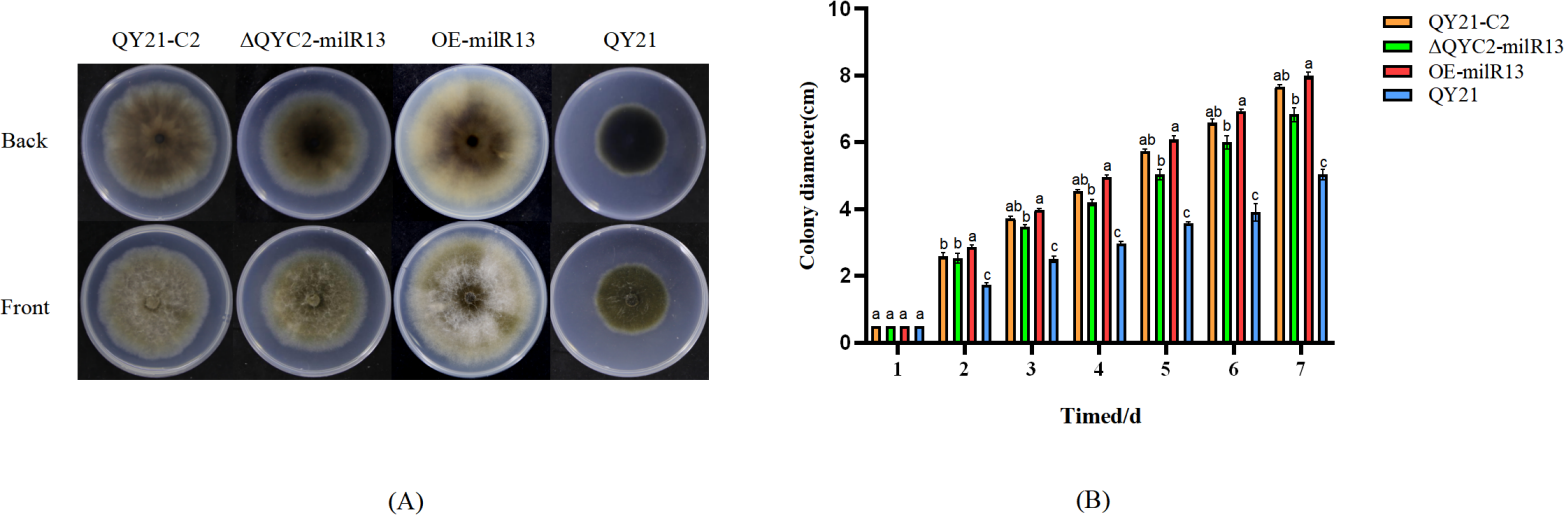
Effects of Aa-milR13 on colony phenotype and hyphal growth of *Alternaria alternata* f. sp. *mali* strains (cm^2^). **(A)** Colony phenotype; **(B)** Colony diameter. QY21-C2: Virus-free strain; ΔQYC2-milR13: Aa-milR13 knockout mutant strain; OE-milR13: Aa-milR13 overexpression strain; QY21: Virus-coinfection strain. Lowercase letters represent significant statistical differences.

For phenotypic analysis, QY21-C2, QY21, ΔQYC2-milR13, and OE-milR13 were placed at the center of PDA plates and cultured for 1–7 days. Colony diameters were measured daily, and the results are shown in **Figure 8**. The mycelial growth rate of ΔQYC2-milR13 was slower, with a significantly smaller colony diameter compared to QY21-C2. Its colony color and diameter were closer to those of QY21. In contrast, the mycelial growth rate of OE-milR13 was accelerated, and its colony diameter showed no significant difference from that of QY21-C2.

**Figure 9:**
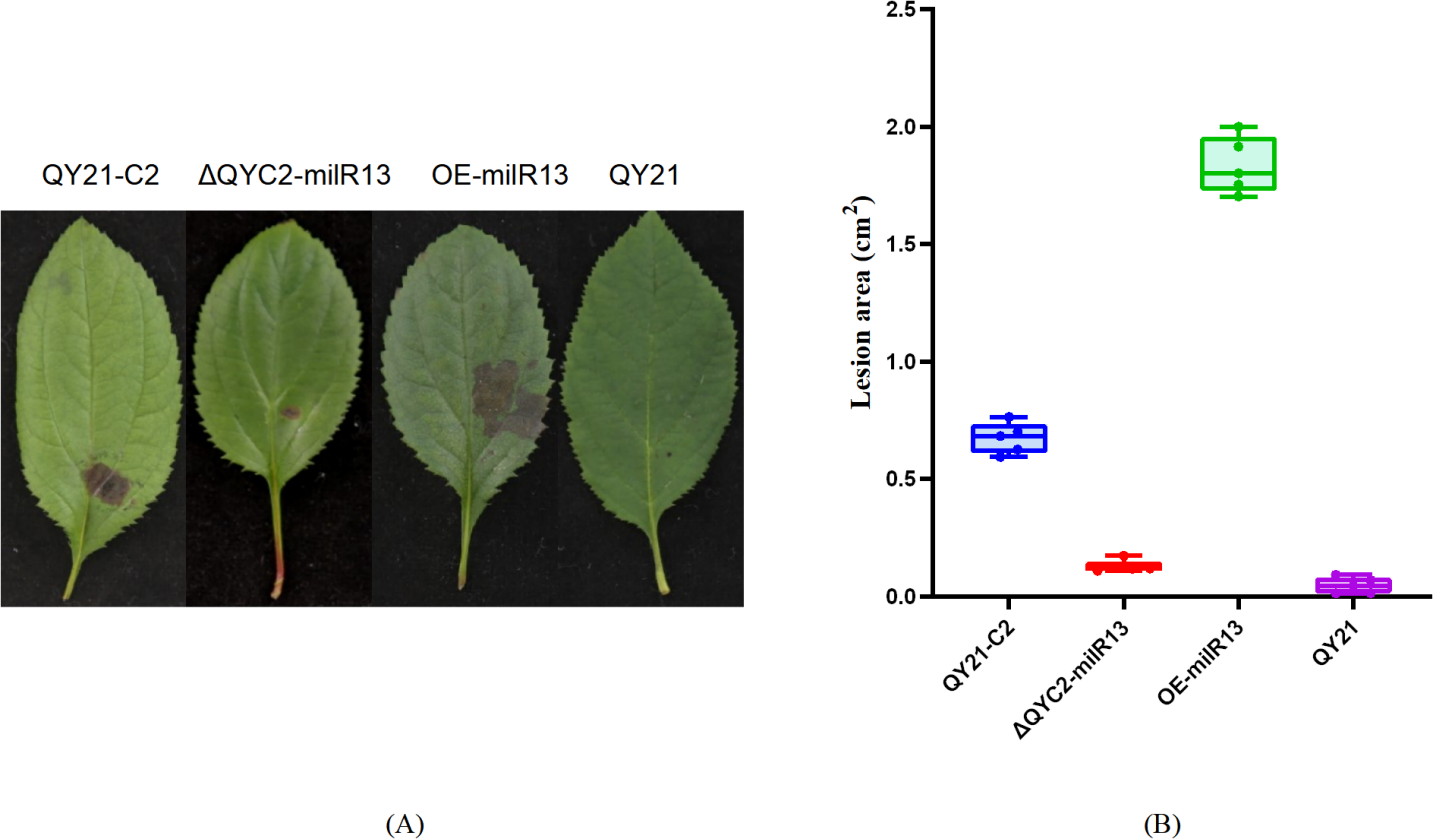
Effects of Aa-milR13 on the pathogenicity of *Alternaria alternata* f. sp. *mali* strains (detached crabapple leaves) (14 days). **(A)** Symptoms on detached crabapple leaves under various treatments; **(B)** The corresponding box plot represents the lesion area (cm²) for each treatment. The box plot displays the median (central line), interquartile range (IQR, box edges), and the range of the data (whiskers). Statistical significance between different strains was evaluated using the ordinary one-way ANOVA test (non-parametric, *P*< 0.0001). Different letters above the boxes indicate statistically significant differences (*P*< 0.05). Data are presented as the mean ± standard error of the mean (SEM) from three biological replicates. QY21-C2: Virus-free strain; ΔQYC2-milR13: Aa-milR13 knockout mutant strain; OE-milR13: Aa-milR13 overexpression strain; QY21: Virus-coninfection strain.

**Figure 10:**
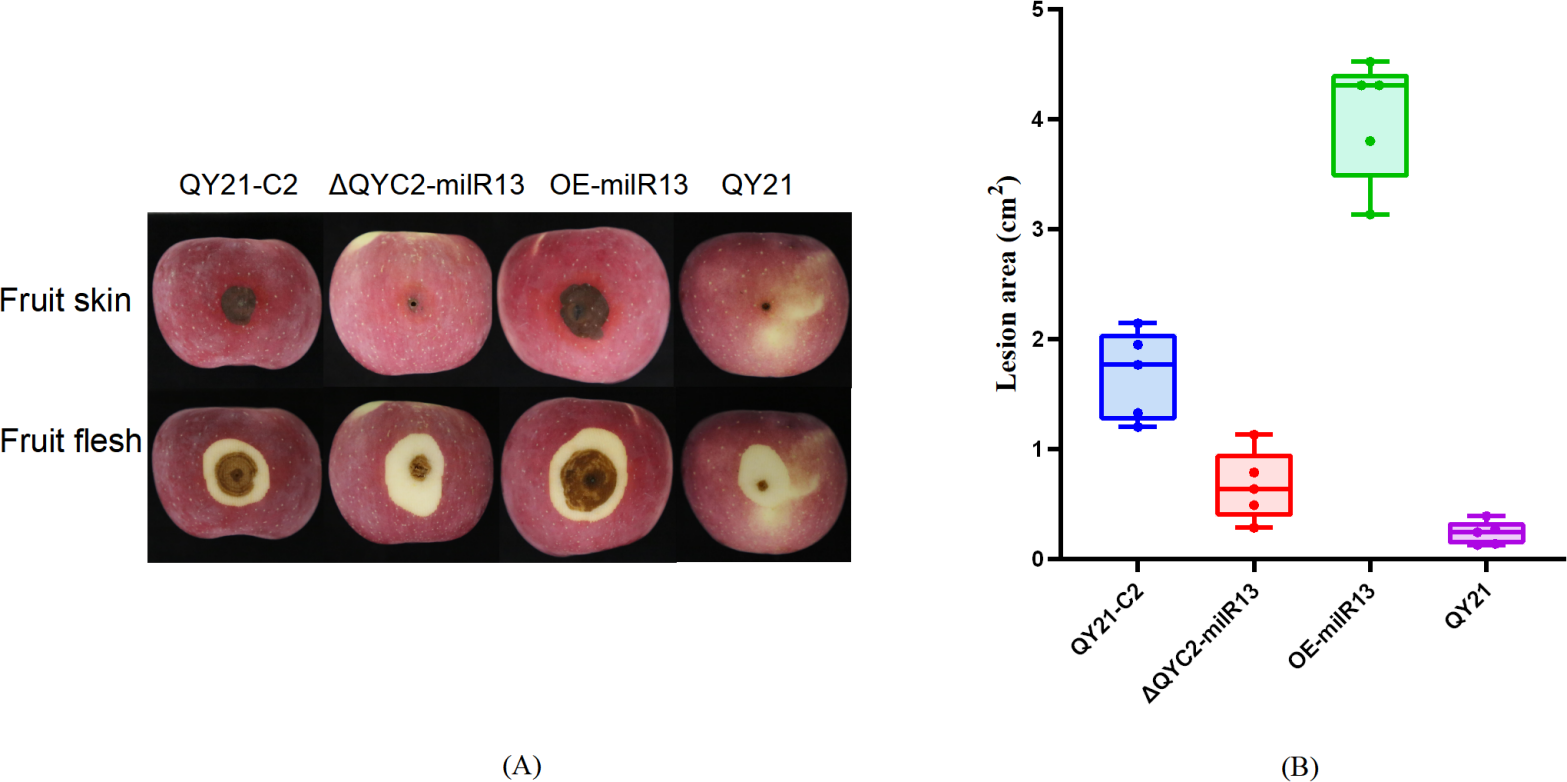
Analysis of the expression of endogenous key milRNAs in *Alternaria alternata* f. sp. *mali* strains by RT-qPCR. **(A)** Aa-milR87(KV441478_174387); **(B)** Aa-milR13(KV441481_212713). QY21-C2: virus-free *Alternaria alternata* f. sp. *mali* strain; QY21-C1: AaMV1-infected *Alternaria alternata* f. sp. *mali* strain; QY21: AaCV1nad AaMV1-infected *Alternaria alternata* f. sp. *mali* strain.

To investigate the effect of Aa-milR13 on pathogenicity, QY21-C2, QY21, OE-milR13, and ΔQYC2-milR13 were cultivated under equivalent conditions (28°C in the dark for 5 days). Five-millimeter-diameter mycelial plugs were collected from each strain and inoculated onto detached crabapple leaves and apple fruits (cv. ‘Tianhong 2’). Lesion diameters were measured 14 days post-inoculation, and the results are shown in **Figures 9**, **10**. OE-milR13 exhibited the strongest pathogenicity, with leaf and fruit lesions significantly larger than those of the virus-free strain QY21-0C2 and other strains (QY21-C2 > ΔQYC2-milR13> QY21). Lesions of ΔQYC2-milR13 were significantly smaller than those of QY21-C2, indicating a marked reduction in pathogenicity. The QY21 strain showed the smallest lesions. These results suggest that deletion of Aa-milR13 significantly reduces the pathogenicity of *A. alternata* strains, whereas overexpression of Aa-milR13 significantly enhances their pathogenicity.

### 3.5 Target genes analysis of Aa-milR13 in *Alternaria alternata* f. sp. *mali*

To further investigate the role of Aa-milR13 in the pathogenicity of *A. alternata*, we predicted the target genes of Aa-milR13. The results are shown in **Table 3**. Three potential intracellular target genes, *AaSLP*, *DUF431*, and *NrtB*, were predicted using the RNAhybrid online website. These genes correspond to a subtilisin-like protease (CC77DRAFT_1100266), a DUF431 domain-containing protein (CC77DRAFT_1022347), and a high-affinity nitrate transporter NrtB (CC77DRAFT_1056077), respectively. 0

**Table 3:** Information on intracellular target genes of Aa-milR13 in *Alternaria alternata* f. sp. *mali*.

Name of target gene	Target_Acc.	Length of target gene	Target regions	Start and stop site of target	Binding free energy	Interaction mode	Encoded protein
*AaSLP*	CC77DRAFT_1100266	2059	1046-1066	U、U	-31.1	Cleavage	Subtilisin-like protein
*DUF431*	CC77DRAFT_1022347	1434	873-893	G、U	-31.4	Cleavage	domain-containing protein
*NrtB*	CC77DRAFT_1056077	1743	1051-1071	U、G	-31.4	Cleavage	High affinity nitrate transporter NrtB

**Figure 11:**
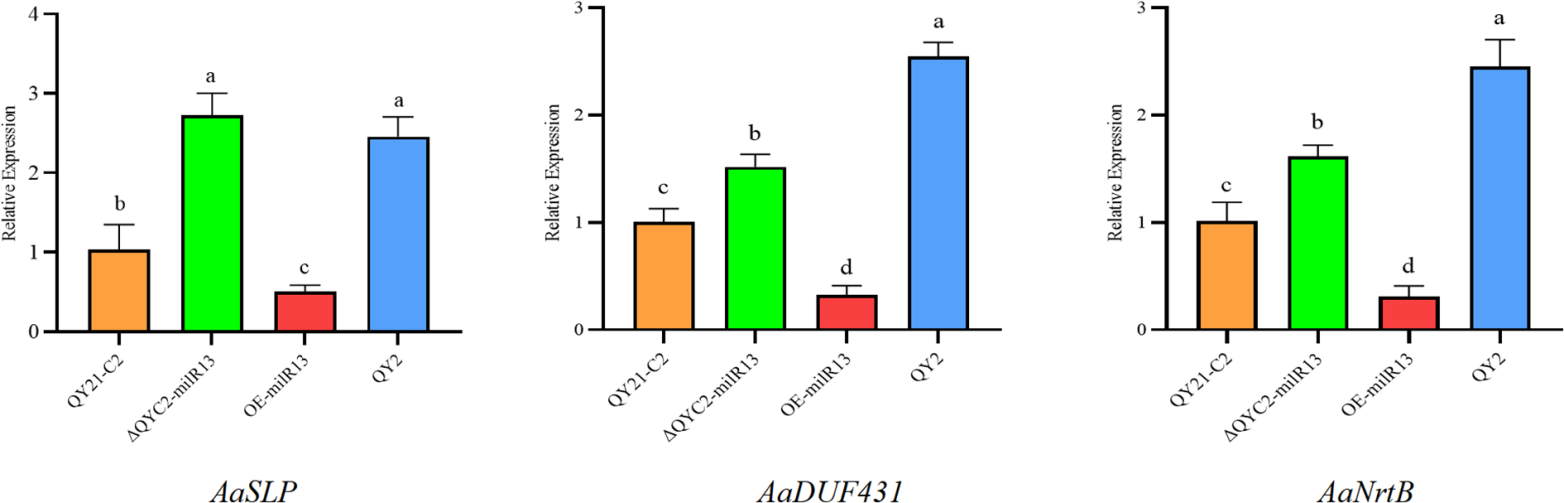
Matching mode between Aa-milR13 from *Alternaria alternata* f. sp. *mali* and its potential target genes. Symbols, *, **, ***, and **** denote statistical significance at the p<0.05, p<0.01, p<0.001, and p<0.0001 levels, respectively. Lowercase letters represent significant statistical differences.

To clarify the synergistic expression changes between these three genes and Aa-milR13 in the strain, RT-qPCR analysis was conducted. The results, shown in **Figure 11**, indicate that the expression levels of the three genes significantly increased following Aa-milR13 knockout, whereas they were significantly downregulated in the overexpression strain compared to QY21-C2. This pattern suggests a significant negative correlation between Aa-milR13 and the expression levels of these target genes, implying that Aa-milR13 may directly target *AaSLP*, *DUF431*, and *NrtB*. Notably, the highest expression levels of the target genes were observed in the virus-infected strain, indicating that viral infection-induced downregulation of Aa-milR13 might be the primary driver of the upregulated expression of these three genes.

**Figure 12:**
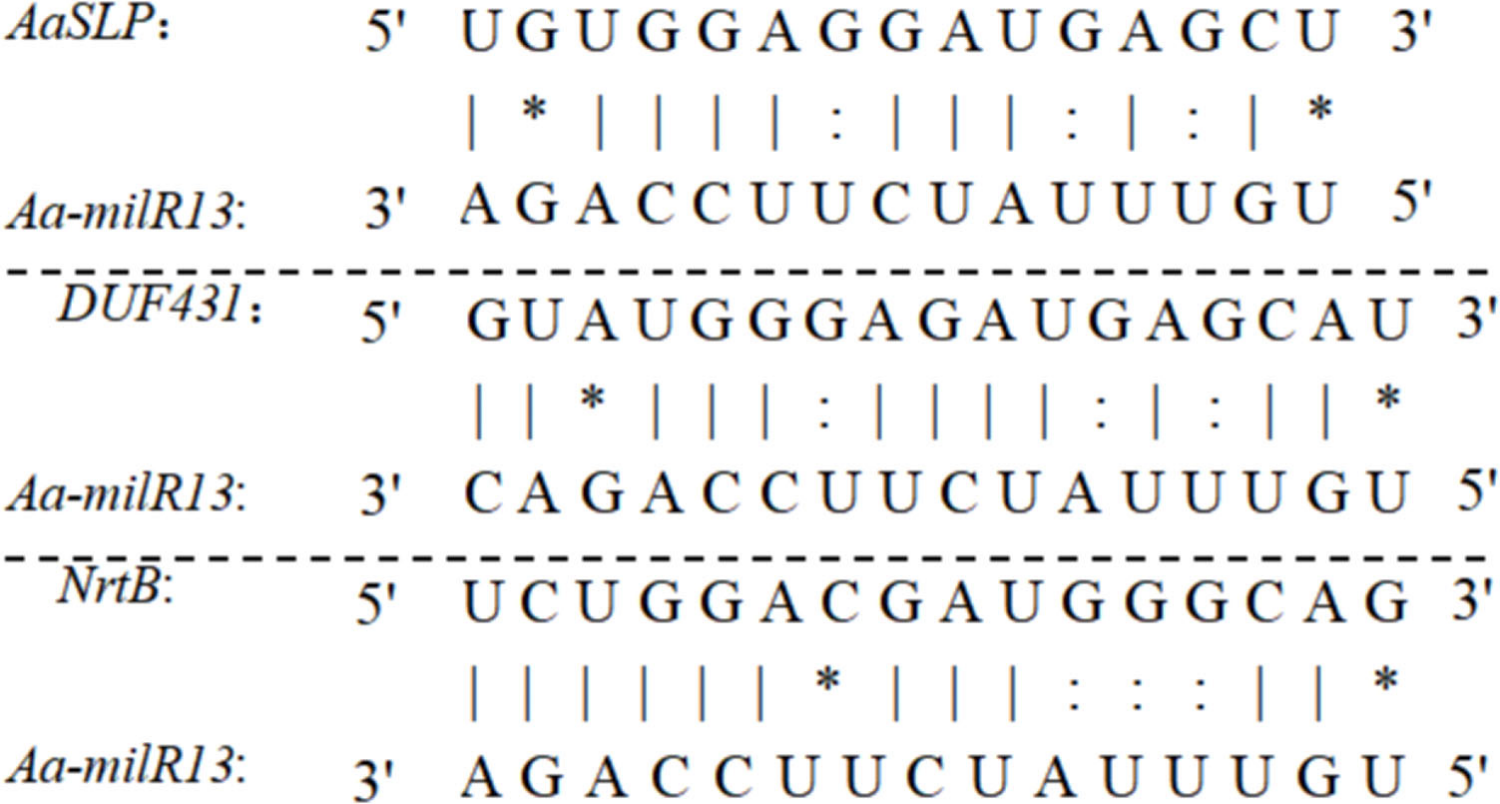
Quantitative RT-qPCR analysis of potential target genes in Aa-milRNAs mutants of *Alternaria alternata* f. sp. *mali*.

Upon reviewing the literature, the functions of the aforementioned potential target genes in *Alternaria* species have not been reported. We analyzed the cleavage mode and target regions of Aa-milR13 to its target genes. The results, shown in **Figure 12**, indicate that the target regions of Aa-milR13 are all located in the CDS regions of the target genes and act through cleavage. The pairing between Aa-milR13 and the target genes involves Watson-Crick pairing of AU and CG, wobble pairing of GU, and various other pairing modes such as AA, AC, AG, GG, UU, and UC.

The primary structures of AaSLP, DUF431, and NrtB were analyzed using the Portparam online software. The results show that AaSLP has a molecular mass of 50 kD, a theoretical isoelectric point (pI) of 9.25, and a molecular formula of C2198H3482N642O656S20. It contains multiple amino acids, with a high content of alanine (Ala) at 8.7%. DUF431 has a molecular mass of 23 kD, a theoretical pI of 4.88, and a molecular formula of C_2567_H_3920_N_646_O_676_S_23_. It has the highest leucine (Leu) content at 9.7%, an instability index of 38.9, and is classified as a stable protein. NrtB has a molecular mass of 55 kD, a theoretical pI of 8.89, and a molecular formula of C2567H3920N646O676S23. It has a high glycine (Gly) content at 10.4%, an instability index of 25.66, and is considered a stable protein.

**Figure 13:**
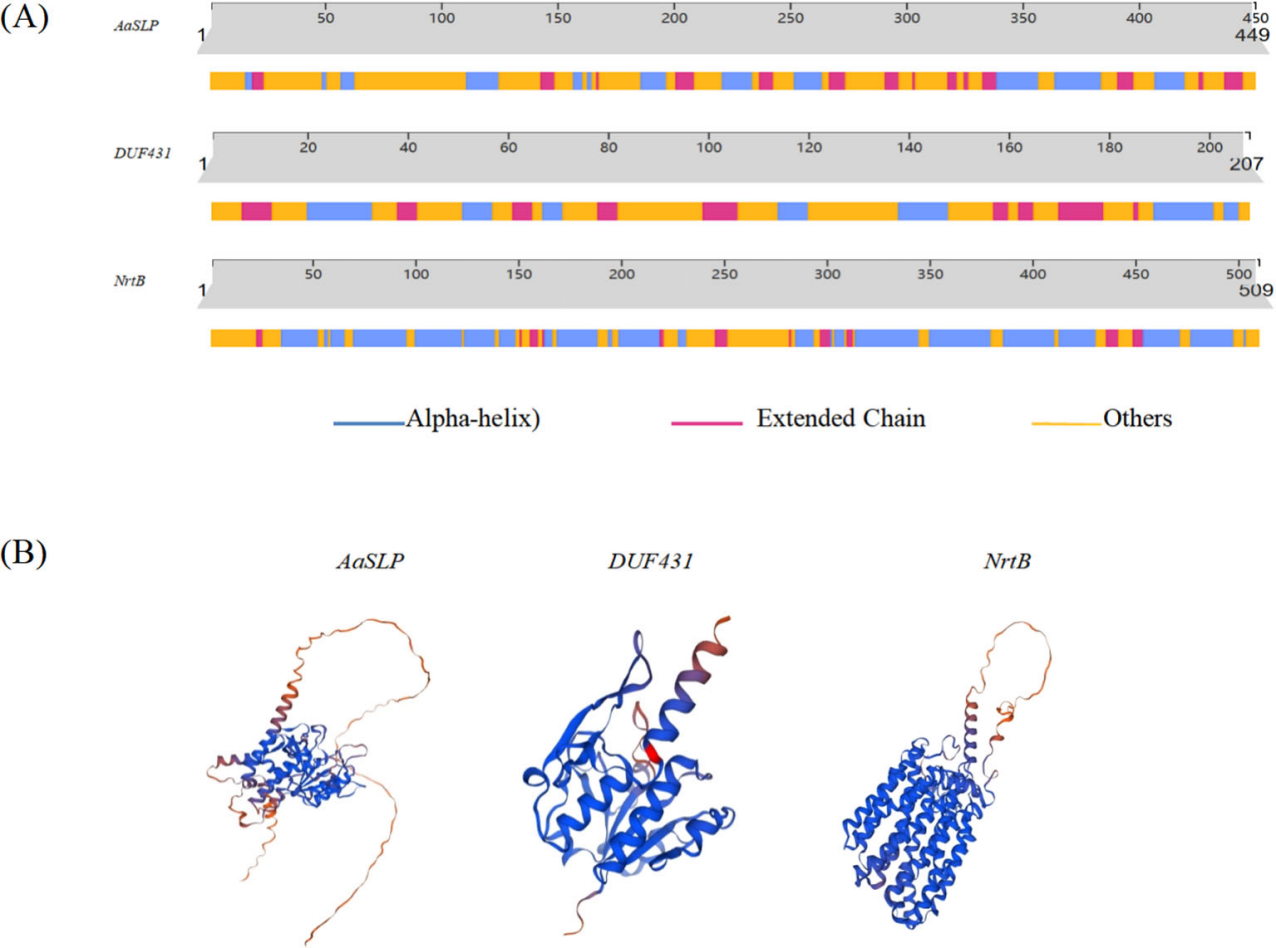
Structures of potential target proteins of Aa-milR13 from *Alternaria alternata* f. sp. *mali.* **(A)** Secondary structures of potential target proteins; **(B)** Tertiary structures of potential target proteins.

The secondary structures of AaSLP, DUF431, and NrtB proteins were predicted using the predictprotein software. The results, shown in **Figure 13**, indicate that all three are proteins with heterogeneous secondary structure compositions. AaSLP and DUF431 are dominated by random coils, accounting for more than 50%, while NrtB is dominated by α-helices, accounting for 56.19% (**Figure 13A**). Homologous three-dimensional structural modeling using the SWISS-MODEL online tool revealed that the three genes are mainly composed of α-helices and random coils, with a small amount of β-sheets, which is consistent with the secondary structure prediction results (**Figure 13B**).

## 4 Discussion

### 4.1 Aa-milR13 may mediate the regulation of host virulence by mycovirus

The RNA silencing system serves as a primary signaling pathway for virus-host interactions. RNA interference (RNAi) is a conserved mechanism in eukaryotes that regulates gene expression through mRNA cleavage, transcriptional or translational repression (Chang et al., 2012). MicroRNAs (miRNAs) are one of the key regulatory factors of RNAi in eukaryotes (Ghildiyal and Zamore, 2009). When a virus infects a host, it can alter the levels of host miRNAs, thereby regulating the expression of numerous target genes and achieving widespread gene silencing effects. This, in turn, affects the physiological metabolism and various critical functions of the host, such as growth and reproduction, substance transport and metabolism, stress response and resistance, pathogenicity, and parasitism ability. The discovery of milRNA in fungi lags behind that in plants, with a relatively small number and low conservation. With the advancement of sequencing technology, an increasing number of fungal milRNA have been discovered and identified. Using sequencing technology, two milRNA and 42 novel milRNA have been found in *Sclerotinia sclerotiorum* (Padmanabhan et al., 2009), 19 milRNA have been identified in *F. oxysporum* (Ruiz-Ferrer and Voinnet, 2009), and 57 milRNA have been identified in *Valsa mali* (Park and Shin, 2015). In this study, 44 putative milRNAs (homologous to miRBase-annotated miRNAs) and 172 novel milRNAs were identified in *A. alternata* f. sp. *mali* strains, representing the first comprehensive characterization of milRNAs in this species. We analyzed the differentially expressed milRNA in strains with mycovirus and without mycovirus. The expression levels of Aa-milR87 and Aa-milR13 were significantly lower in the virus-taking strains QY21 and QY21-C1 than in the virus-free strain QY21-C2, with no significant difference between the virus-taking strains. This was verified by RT-qPCR, and Aa-milR13 exhibited a more significant difference. When Aa-milR13 was knocked out in the virus-free strain, the biological traits of the mutant were closer to those of the virus-taking strain, and its pathogenicity was also significantly reduced. This confirms that Aa-milR13 is likely involved in the process of virus-regulated hypovirulence in the host.

### 4.2 The targeting mode of Aa-milR13 to target genes

Fungal virus infection induces differential expression of functional milRNA in the host, which forms the miRISC silencing complex by binding to AGO proteins and targets key virulence genes, leading to host virulence decline (Kobayashi and Tomari, 2016; Iwakawa and Tomari, 2022). In plants and animals, different AGOs determine the type of bound sRNA based on their 5’ terminal nucleotide preference (Eggleston, 2009). Arabidopsis AGO2 and AGO4 mainly bind sRNA starting with 5’ adenine (A), while AGO1 primarily binds 5’ uracil (U) sRNA (Mi et al., 2008). In this study, Except for 23 nt milRNA with a preference for G as the first base, milRNA of other lengths preferred U. This suggests that among the AGOs, at least one prefers binding to 5’ uracil (U) and one prefers binding to 5’ guanine (G), but further research is needed to determine which AGO proteins play a major role.

In addition, unlike plant and animal miRNAs, Aa-milR13 employs a multi-compatible base-pairing mechanism with its potential fungal target genes (*AaSLP*, *DUF431*, and *NrtB*). Aa-milR13 primarily interacts with the coding sequences (CDS) of its targets, diverging from animal miRNAs (3’UTR-focused) or plant miRNAs (CDS-start-site-focused) (Bartel, 2004). This CDS-centric targeting allows direct disruption of fungal protein synthesis. Aa-milR13 accommodates Watson-Crick (AU/CG), G-U wobble, and non-canonical (AA/AC/AG/GG/UU/UC) pairs, relaxing the “seed-region rigidity” of canonical miRNAs (Axtell et al., 2011). Aa-milR13 drives efficient mRNA cleavage in a pairing-pattern-dependent manner, contrasting with animal miRNAs (rare cleavage) or plant miRNAs (cleavage restricted to near-perfect complementarity). This cleavage dominance reflects fungal evolutionary adaptations to rapidly silence critical genes (Dang et al., 2011). The findings of this study provide clues for a deeper understanding of the regulatory mechanisms of gene expression.

### 4.3 Prediction of Aa-milR13 potential target gene functions

We further predicted the function of the potential target genes of Aa-milR13. We speculated that virus infection triggers the downregulation of Aa-milR13 in the host *A. alternata*, which in turn upregulates the expression of target genes, thereby affecting the host’s biological traits and virulence.

The predicted Aa-milR13 potential target genes in the host include Subtilisin-like protein, high affinity nitrate transporter NrtB, and DUF431-domain-containing protein. The functions of these genes in *A. alternata* have not been reported. Firstly, as a type of protease, Subtilisin-like protein involved in protein hydrolysis and degradation processes. It has been reported that Subtilisin-like proteins in microorganisms are associated with inducing programmed cell death in host cells, which can affect the expression of defense enzymes and defense-related genes in the host, and trigger host immune responses (Jiang et al., 2011; Wang et al., 2022; Xiong et al., 2024). High Affinity Nitrate Transporter (NrtB) is usually found in plants, responsible for transmembrane transport of nitrate in environments with low nitrate concentrations, and is a key protein for plant nitrogen nutrition absorption. Akhtar et al. (2015) reported disruption of the *Aspergillus nidulans* high-affinity nitrate transporter genes NrtB prevents growth on nitrate (Akhtar et al., 2015). DUF431 domain-containing protein is predicted to be an SAM-dependent RNA methyltransferase in NCBI, catalyzing RNA methylation and RNA modification enzymes, which has not been reported in *A. alternata*. however, in *F. verticillioides*, it was found that RNA m5C methylation modification was negatively correlated with fumonisin biosynthesis (Hou et al., 2024). In our study, when *A. alternata* is infected by virus, the expression of Aa-milR13 is downregulated. This downregulation may relieve the inhibitory effect of Aa-milR13 on its target genes. Consequently, these alterations might indirectly influence the overall physiology and metabolism of the fungus, including its pathogenicity. Further in-depth research is required to determine the precise interactions.

In addition, we also analyzed the target genes of Aa-milR13 in plants (**Table 4**) and found that the number of target genes in apples was much higher than that in *A. alternata*. Most of the target genes in apples are related to growth and development regulation and apple host defense immunity. To further clarify the cross-kingdom regulatory potential of Aa-milR13, we could perform dual-luciferase reporter assays in *Nicotiana benthamiana* to validate its direct post-transcriptional regulation of high-confidence apple defense genes (Feng et al., 2023), which would help reveal how Aa-milR13 interacts with host gene expression during infection. Second, to investigate the molecular mechanisms underlying its cross-kingdom activity, we could track Aa-milR13 subcellular localization in *N. benthamiana* using fluorescent labeling and live-cell imaging, as previously observed for fungal small RNAs like those from *B. cinerea* (He et al., 2023). Additionally, testing the dependence of Aa-milR13-mediated regulation on host RNAi components via knockout assays could clarify its reliance on plant machinery (Feng et al., 2017). Collectively, these studies will clarify Aa-milR13’s role as a cross-kingdom regulator.

**Table 4:** Information of target genes of Aa-milR13 from *Alternaria alternata* f. sp. *mali* in apple.

Target_Acc.	Target regions	Binding free energy	Encoded protein
LOC103454784	1923-1946	-20.33	developmentally-regulated G-protein 3-like
LOC103426542	643-665	-21.94	starch synthase 4, loroplastic/amyloplastic
LOC103412857	1083-1103	-21.35	protein SUPPRESSOR OF PHYA-105 1
LOC103427323	696-716	-22.43	LRR receptor-like serine/threonine-protein kinase At3g47570
LOC103432454	2721-2741	-20.11	elongation factor Tu, mitochondrial
LOC103436426	2717-2738	-20.17	homeobox protein HAT3.1-like
LOC103443237	531-551	-23.74	xyloglucan glycosyltransferase 5
LOC103453238	644-665	-20.37	early light-induced protein 1, chloroplastic-like
LOC103437620	626-647	-20.11	heavy metal-associated isoprenylated plant protein 23
LOC103433724	25827-25847	-21.2	rust resistance kinase Lr10-like
LOC103420956	5759-5777	-22.91	TMV resistance protein N-like
LOC103434853	1683-1708	-20.37	peroxidase 19
LOC103439930	1297-1314	-20.31	CDPK-related kinase 4-like
LOC103412597	3450-3472	-21.36	endo-1,4-beta-xylanase 5-like
LOC108169478	1199-1219	-21.61	DNA (cytosine-5)-methyltransferase CMT3-like
LOC114819274	3109-3129	-21.86	probable WRKY transcription factor 20

### 4.4 Theoretical significance and practical application value of studying Aa-milR13

The study of Aa-milR13 in *A. alternata* has important theoretical significance and practical application value. From a theoretical perspective, studying Aa-milR13 helps to gain a deeper understanding of the life activity mechanisms of fungi, especially the role of small RNAs in fungal growth, development, and pathogenicity. This will provide new clues and ideas for further revealing the biological characteristics of fungi. From a practical application perspective, research on Aa-milR13 is expected to provide new strategies and methods for the prevention and control of apple diseases. For example, inhibitors or interference technologies targeting Aa-milR13 can be developed to reduce fungal pathogenicity and enhance apple defense capabilities, thereby reducing the occurrence of apple diseases. In addition, Aa-milR13 can be utilized as a biomarker for early detection and diagnosis of apple diseases, providing a basis for timely adoption of prevention and control measures.

## 5 Conclusion

In this study, through miRNA sequencing and a series of molecular biological experiments, we revealed the relationship between Aa-milR13 and the regulation of host pathogenicity by mycovirus in *A. alternata* f. sp. *mali* strains. The results showed that the expression levels of Aa-milR13 in the two virus-taking strains with low pathogenicity were significantly lower than those in the virus-free strain, Knockout of Aa-milR13 in virus-free strain resulted in slower hyphal growth, darker colony color, and reduced pathogenicity, resembling virus-infected strains. Conversely, overexpression of Aa-milR13 led to accelerated hyphal growth, lighter colony color, and significantly enhanced pathogenicity. Meanwhile, the expression levels of three potential target genes of Aa-milR13 (*AaSLP*, *DUF431* and *NrtB*) were significantly upregulated in the knockout mutant, but remarkably downregulated in the Aa-milR13-overexpressed strain, showing a negative correlation with the expression level of Aa-milR13. Bioinformatics analysis indicated that Aa-milR13 targets the CDS regions of these genes through cleavage. This study is the first to report the involvement of small RNAs as an intermediate bridge in the regulation of host virulence by mycovirus, laying the foundation for elucidating the mechanism of small RNA-mediated regulation of host fungal hypovirulence by mycovirus and providing theoretical support for the utilization of mycovirus in the control of crop fungal diseases.

## Data Availability

The original contributions presented in the study are publicly available. This data can be found here: https://www.jianguoyun.com/p/DbH_84cQzbvGDRjcvokGIAA.
