# Supplementary figures and images for: The infection of mycovirus down regulates Aa-milR13 to weaken the pathogenicity of the Alternaria alternata f. sp. mali
Source: Front Plant Sci. 2025 Jul 22;16:1598183. doi: 10.3389/fpls.2025.1598183 (PMC12322705; doi:10.3389/fpls.2025.1598183)

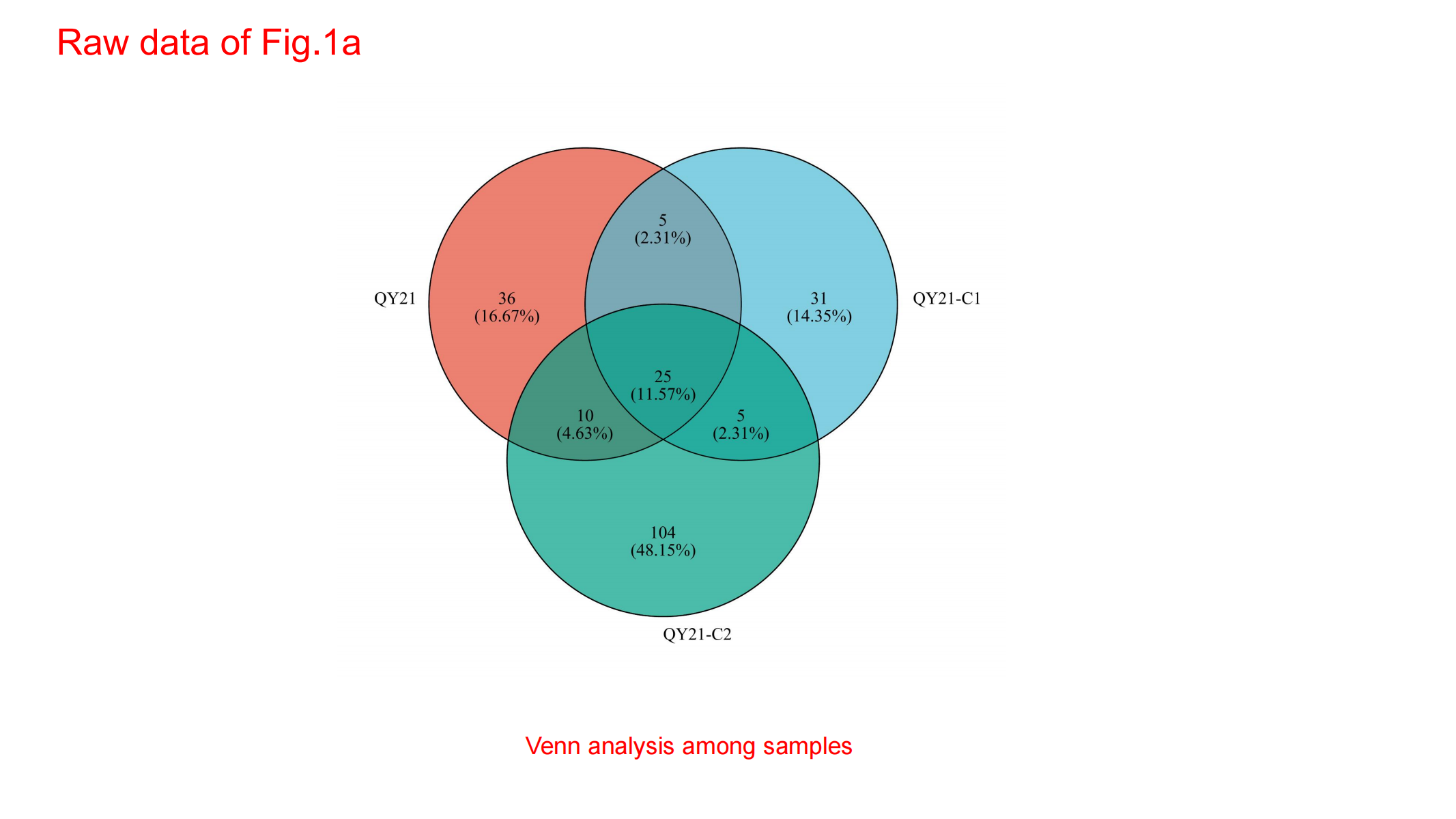

Supplement: Supplementary file 1 [file DataSheet1.zip › Raw images Fig1-6/Fig 1a Venn analysis among samples.tif]

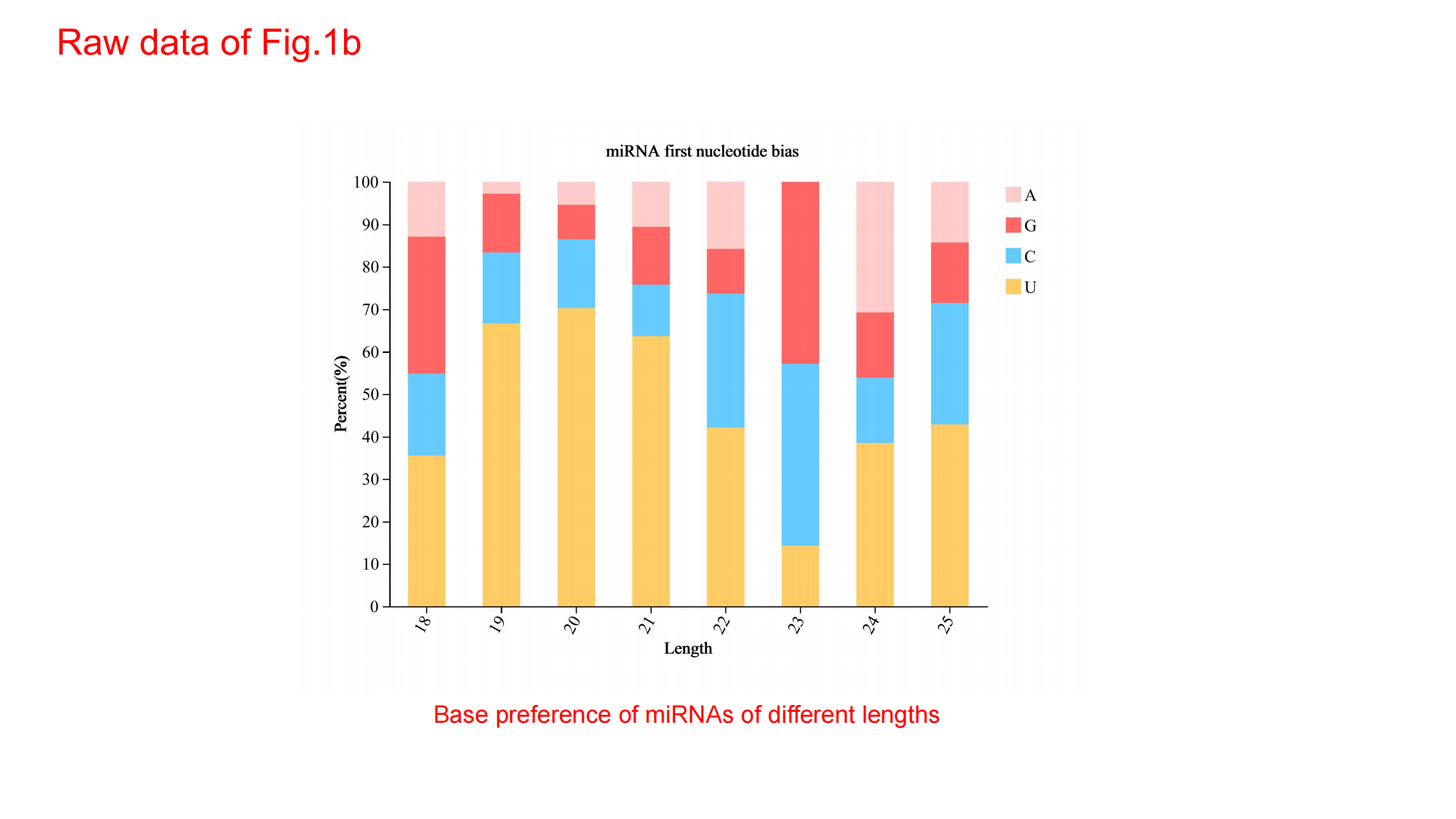

Supplement: Supplementary file 1 [file DataSheet1.zip › Raw images Fig1-6/Fig 1b Base preference of miRNAs of different lengths.tif]

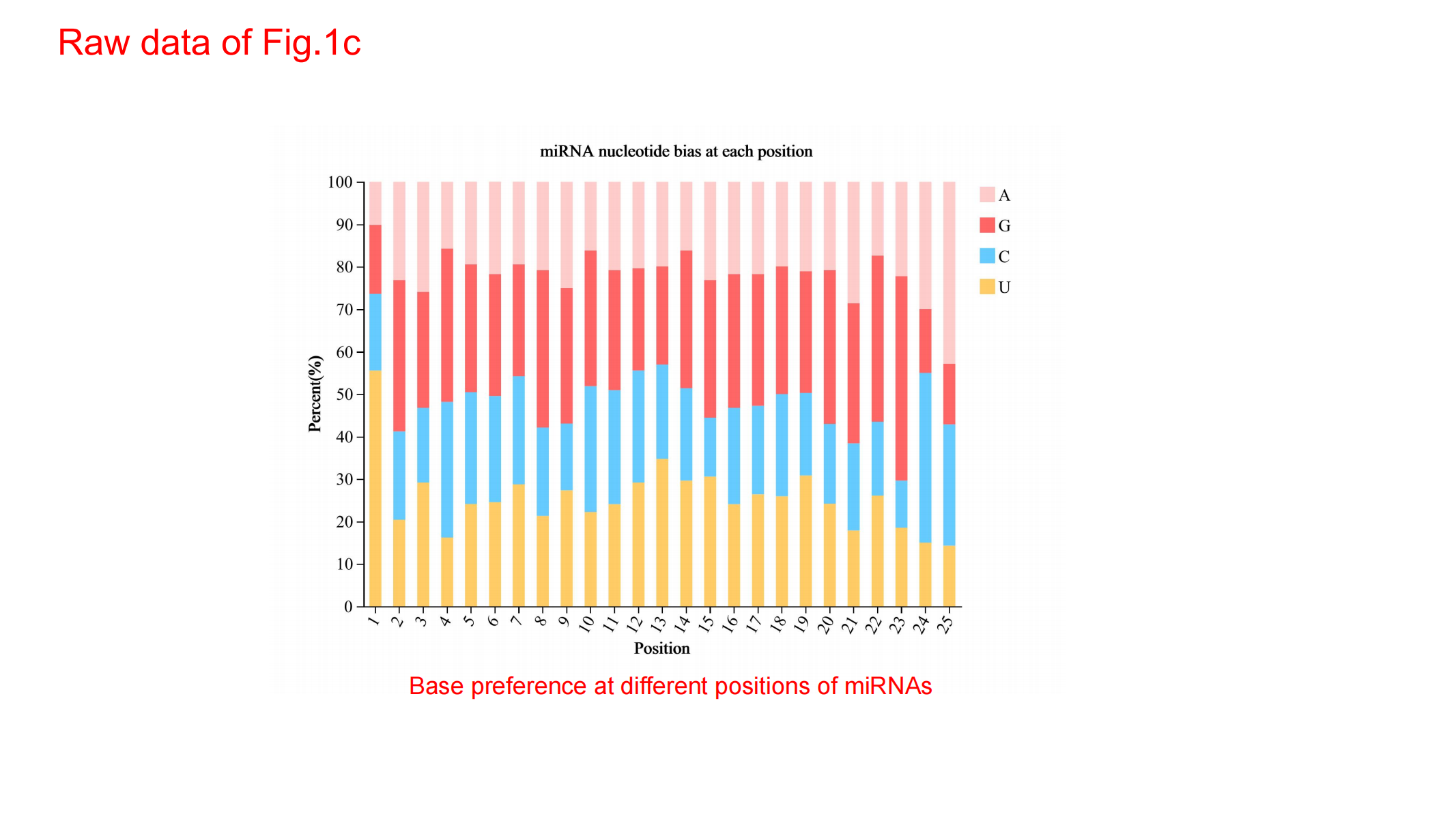

Supplement: Supplementary file 1 [file DataSheet1.zip › Raw images Fig1-6/Fig 1c Base preference at different positions of miRNAs.tif]

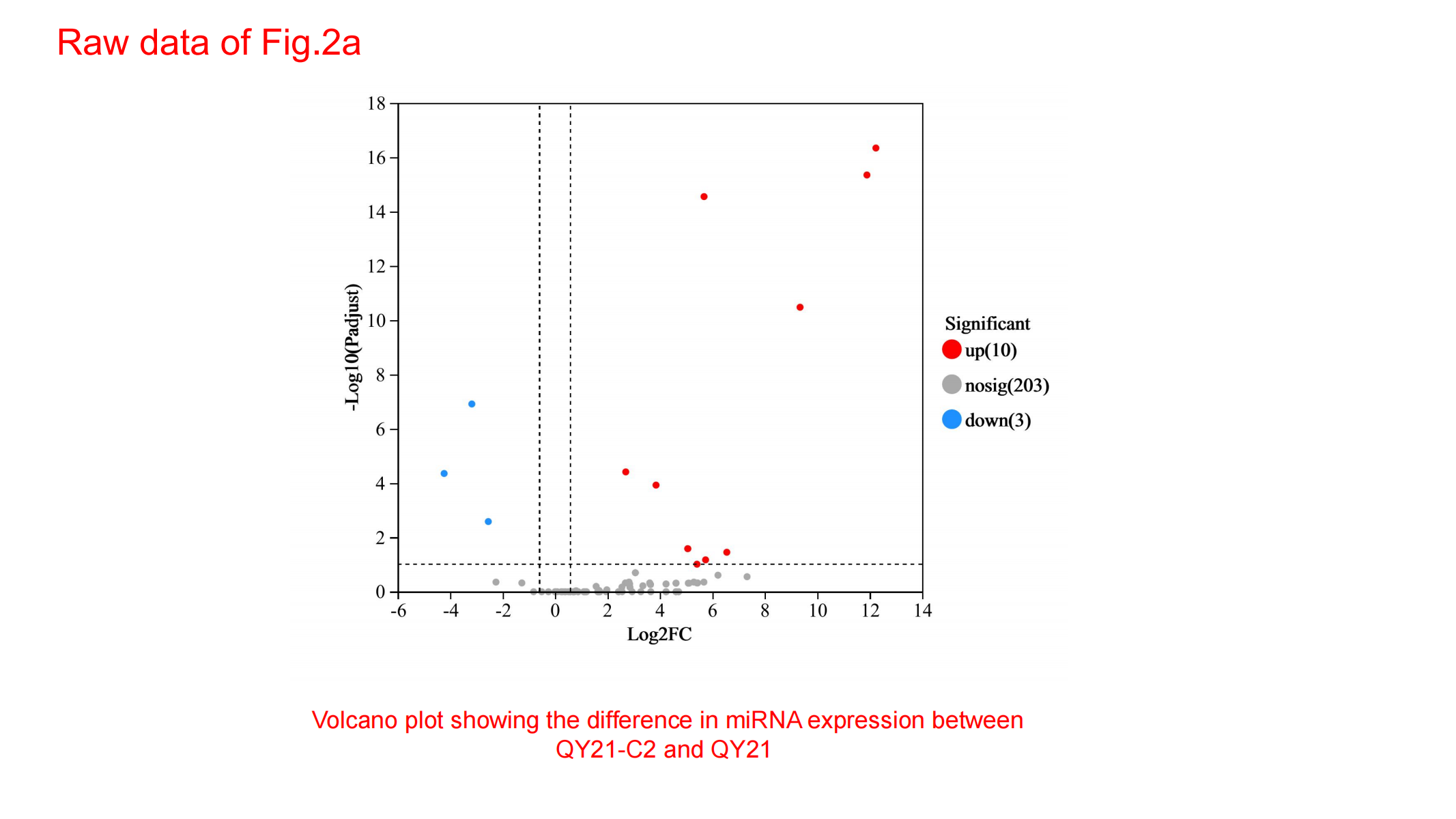

Supplement: Supplementary file 1 [file DataSheet1.zip › Raw images Fig1-6/Fig 2a Volcano plot showing the difference in miRNA expression between QY21-C2 and QY21.tif]

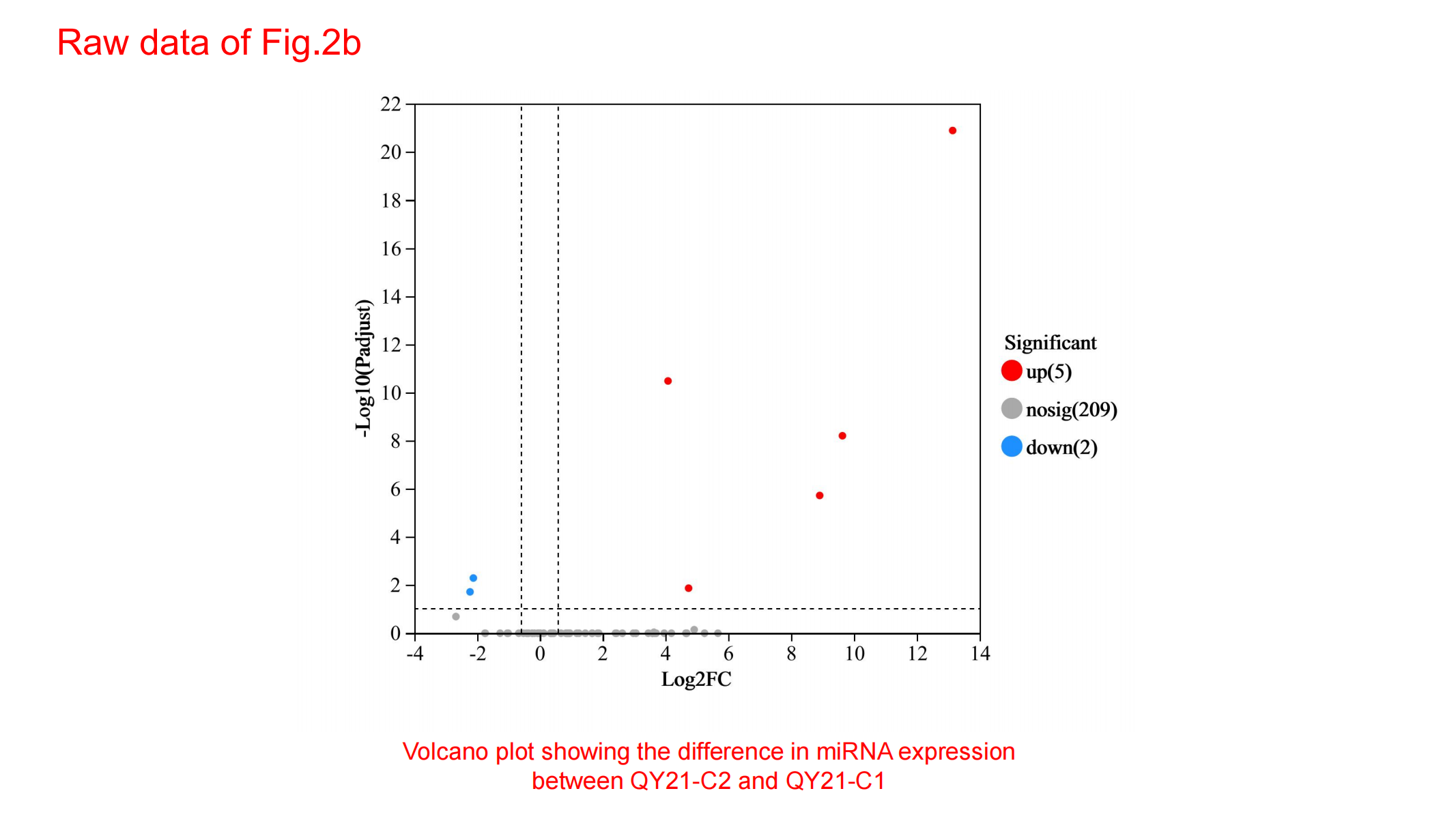

Supplement: Supplementary file 1 [file DataSheet1.zip › Raw images Fig1-6/Fig 2b Volcano plot showing the difference in miRNA expression between QY21-C2 and QY21-C1.tif]

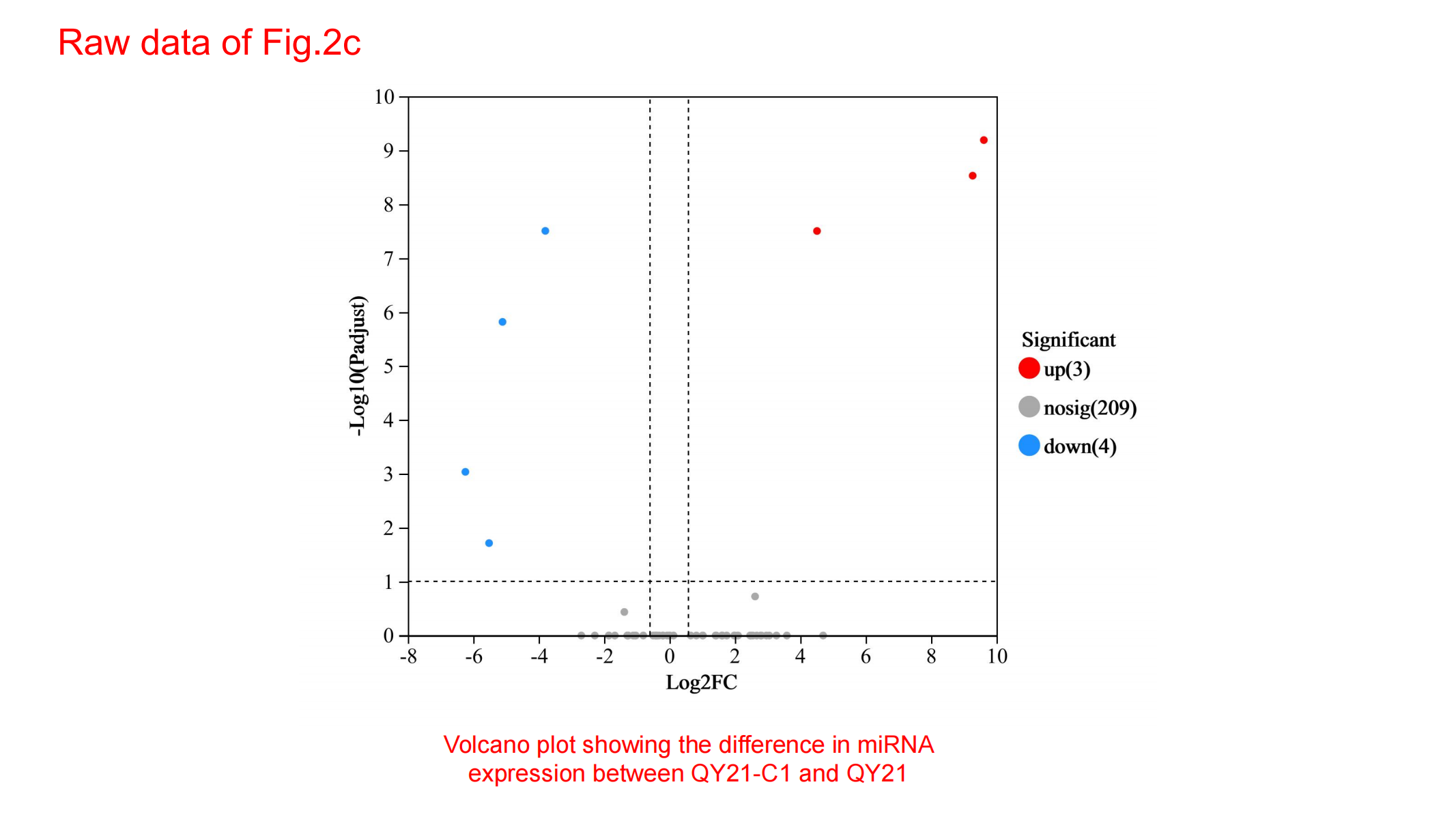

Supplement: Supplementary file 1 [file DataSheet1.zip › Raw images Fig1-6/Fig 2c Volcano plot showing the difference in miRNA expression between QY21-C1 and QY21.tif]

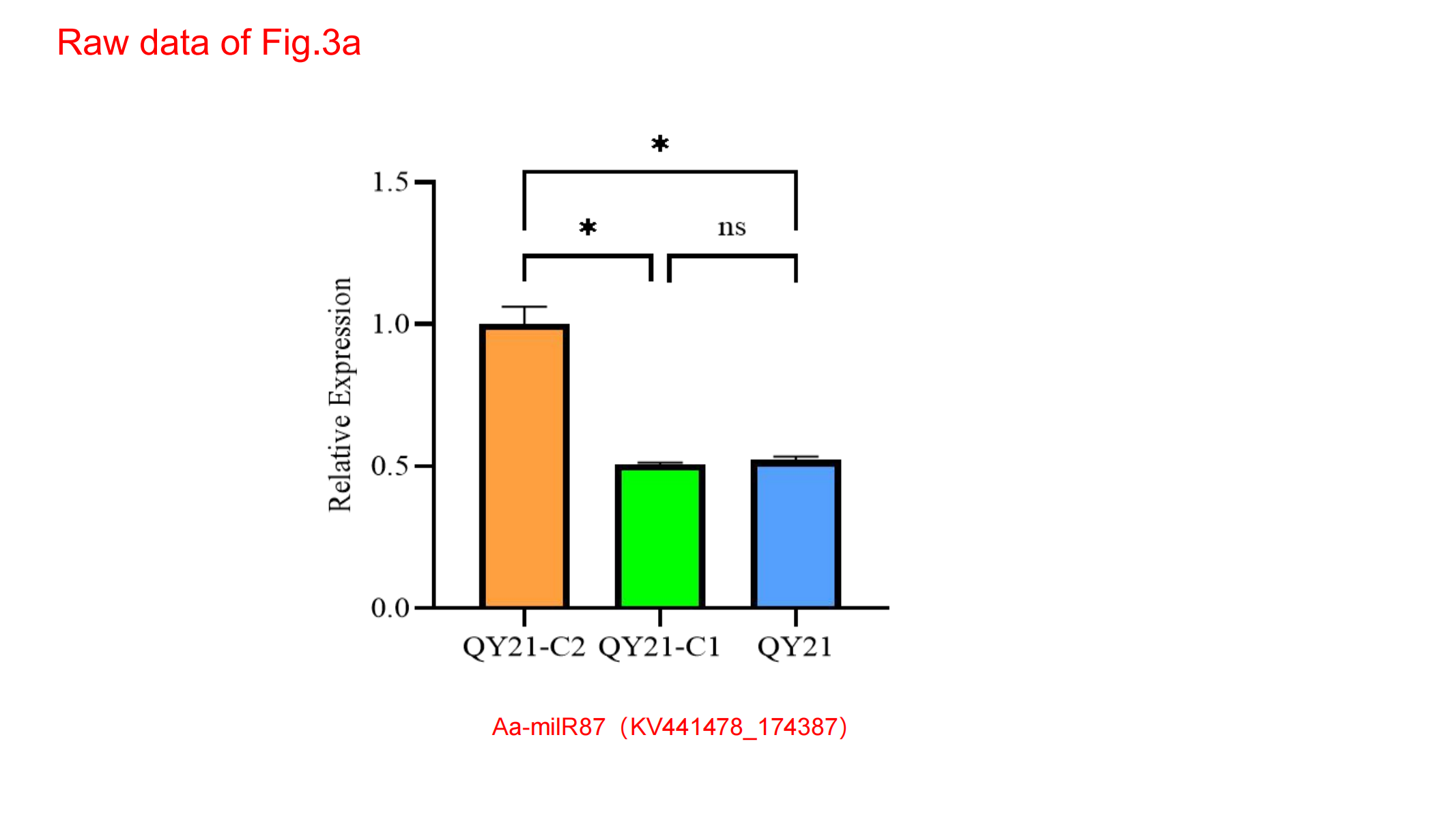

Supplement: Supplementary file 1 [file DataSheet1.zip › Raw images Fig1-6/Fig 3a Aa-milR87(KV441478_174387).tif]

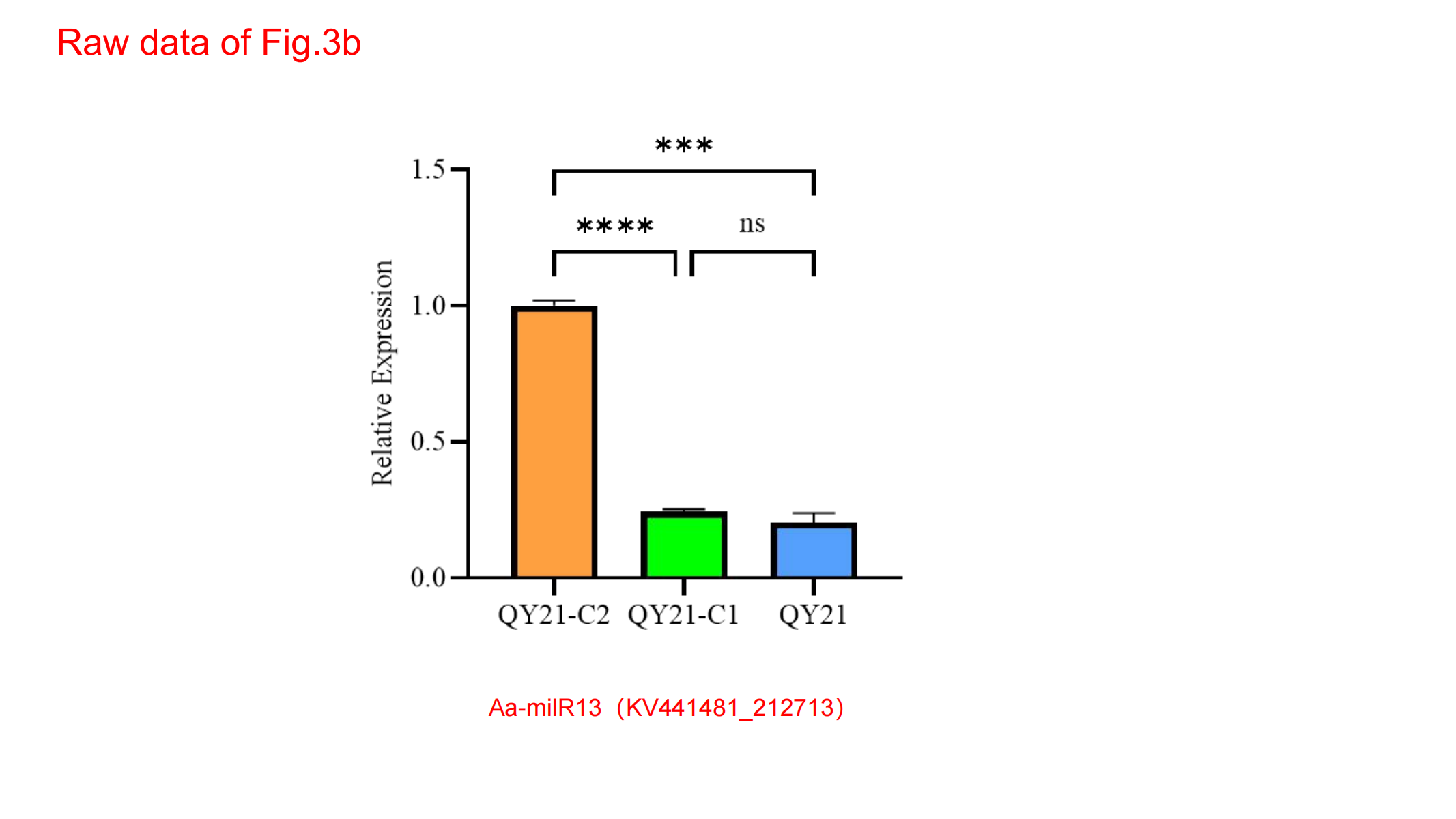

Supplement: Supplementary file 1 [file DataSheet1.zip › Raw images Fig1-6/Fig 3b Aa-milR13(KV441481_212713).tif]

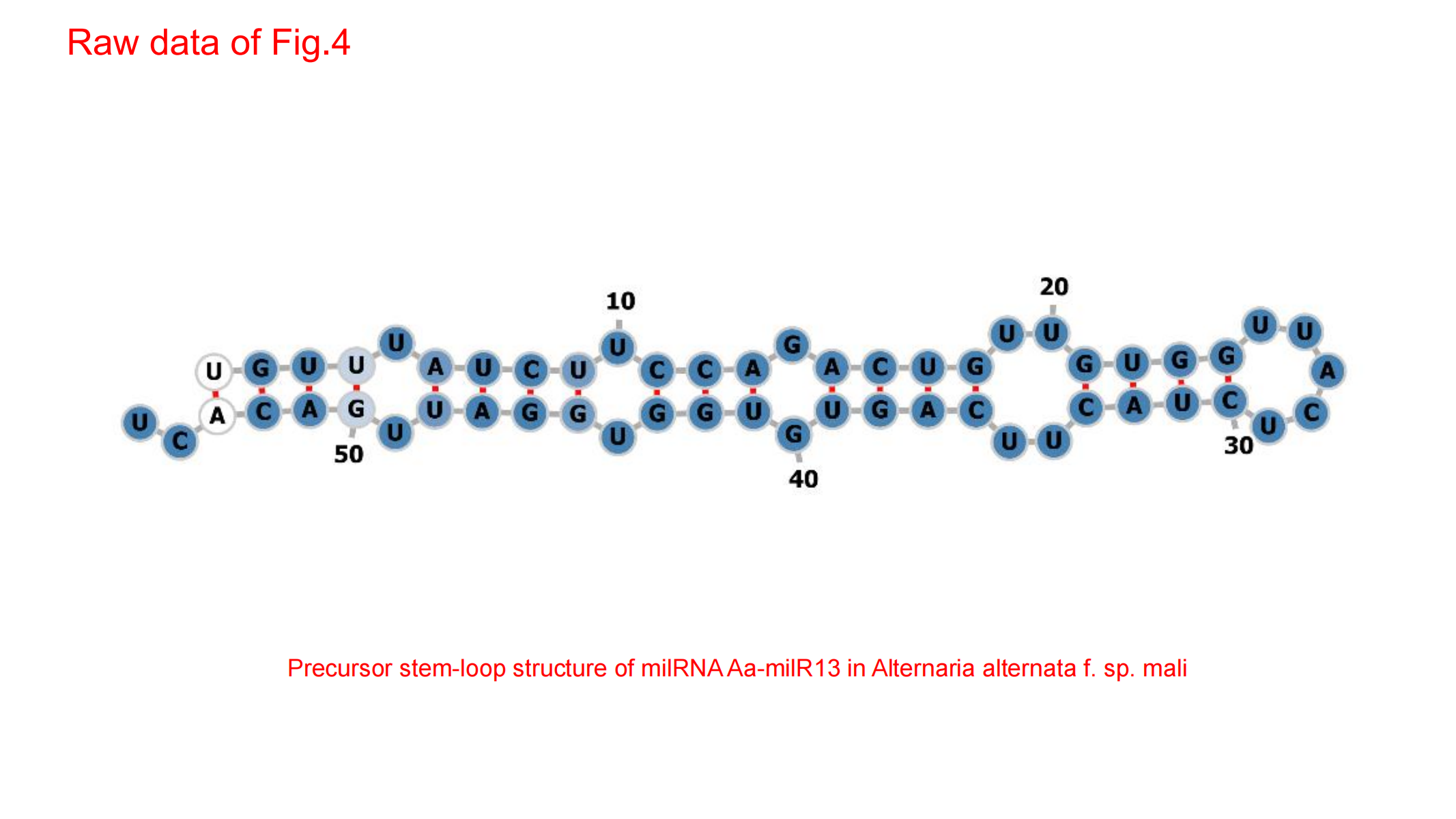

Supplement: Supplementary file 1 [file DataSheet1.zip › Raw images Fig1-6/Fig 4 Precursor stem-loop structure of milRNA Aa-milR13 in Alternaria alternata f. sp. mali.tif]

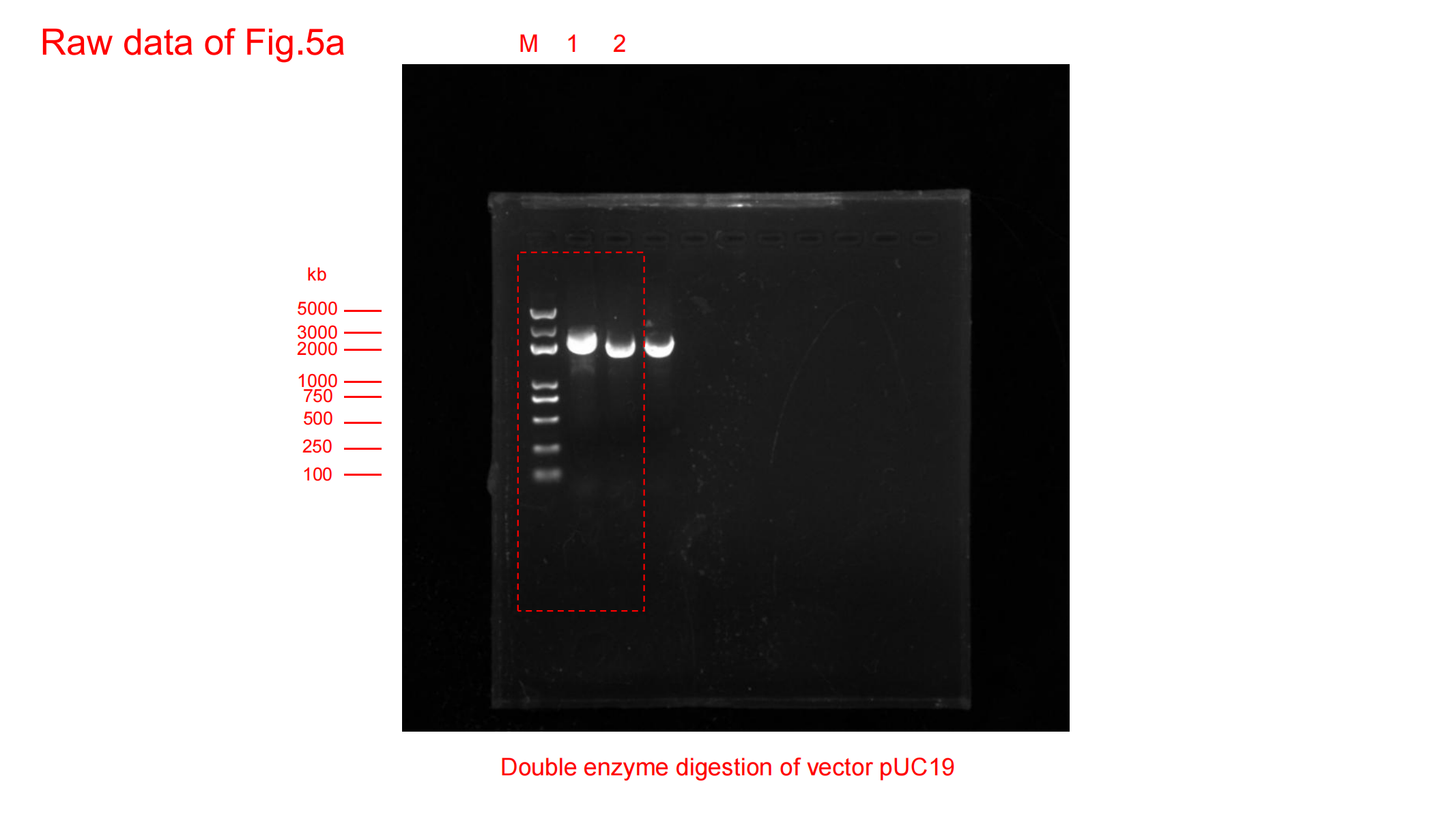

Supplement: Supplementary file 1 [file DataSheet1.zip › Raw images Fig1-6/Fig 5a Double enzyme digestion of vector pUC19.tif]

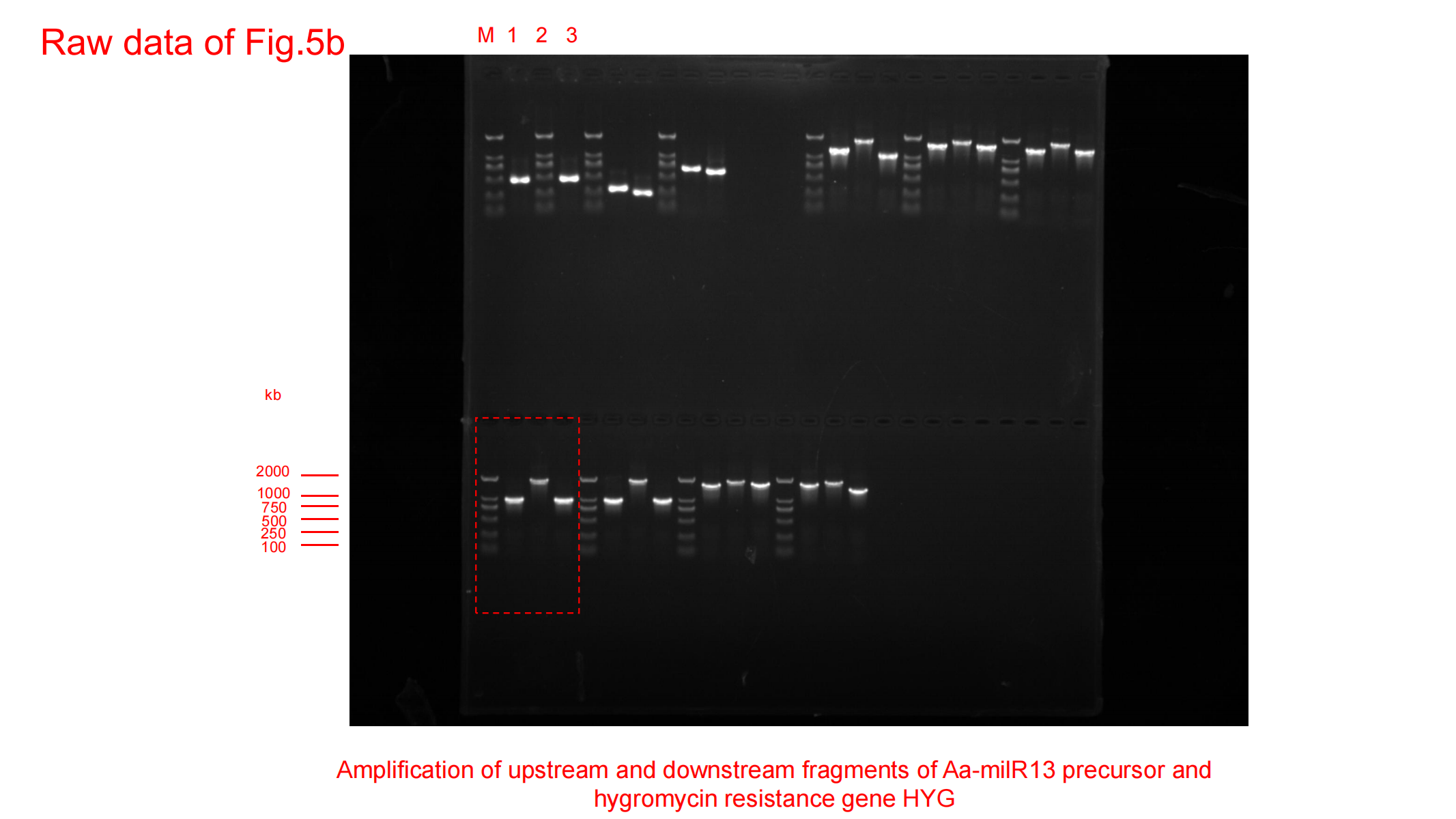

Supplement: Supplementary file 1 [file DataSheet1.zip › Raw images Fig1-6/Fig 5b Amplification of upstream and downstream fragments of Aa-milR13 precursor and hygromycin resistance gene HYG.tif]

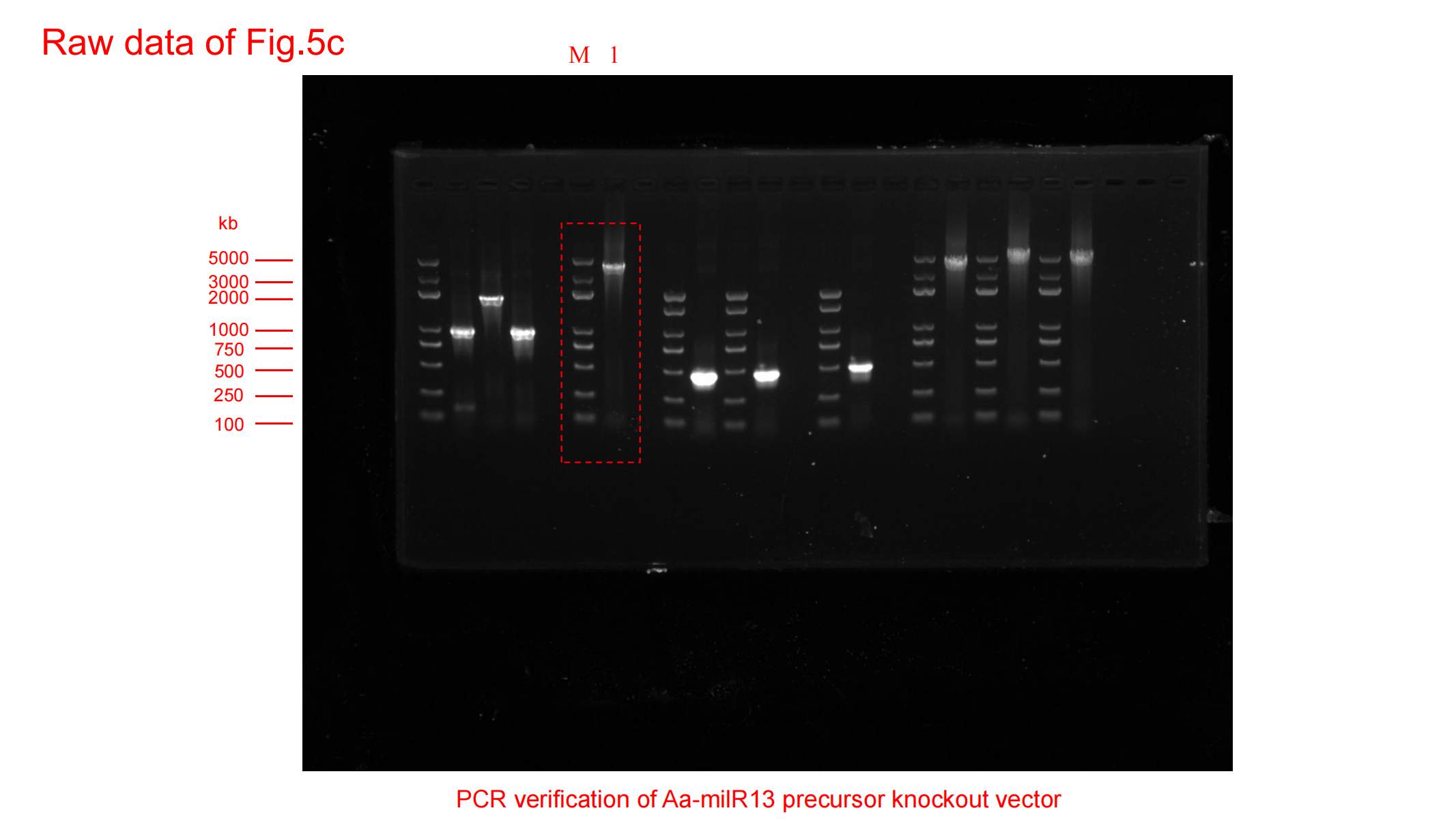

Supplement: Supplementary file 1 [file DataSheet1.zip › Raw images Fig1-6/Fig 5c PCR verification of Aa-milR13 precursor knockout vector.tif]

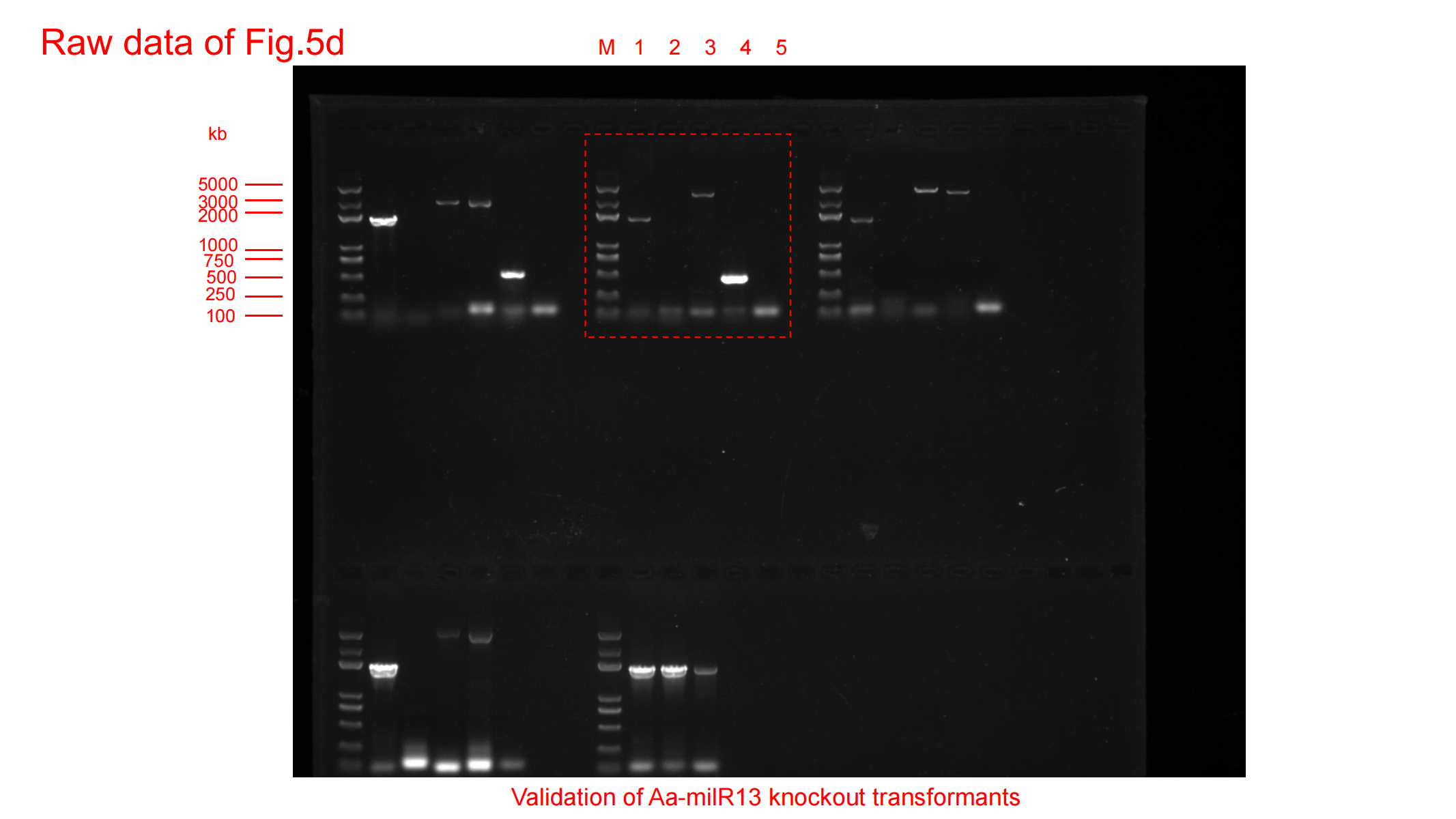

Supplement: Supplementary file 1 [file DataSheet1.zip › Raw images Fig1-6/Fig 5d Validation of Aa-milR13 knockout transformants.tif]

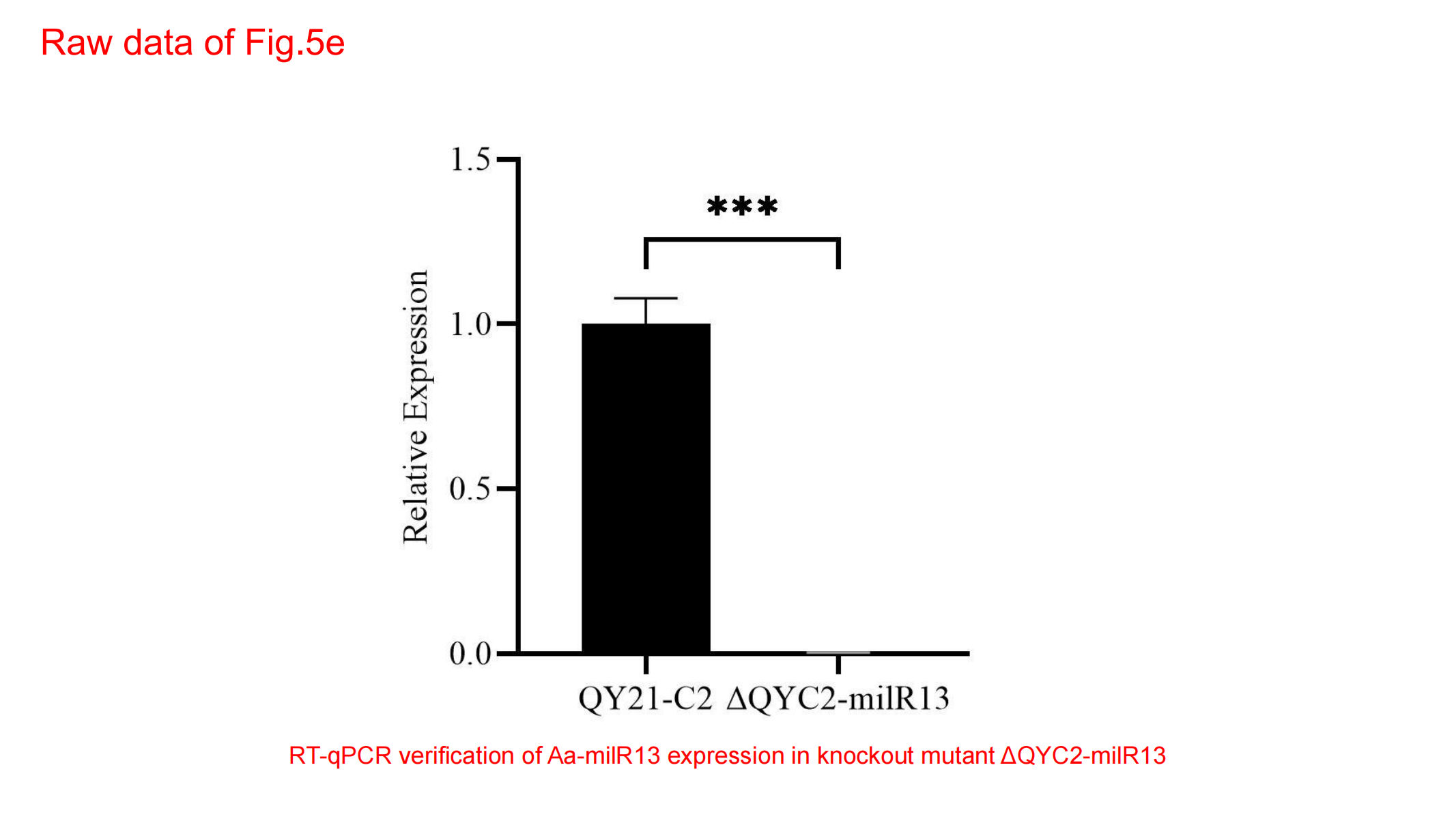

Supplement: Supplementary file 1 [file DataSheet1.zip › Raw images Fig1-6/Fig 5e RT-qPCR verification of Aa-milR13 expression in knockout mutant ΔQYC2-milR13.tif]

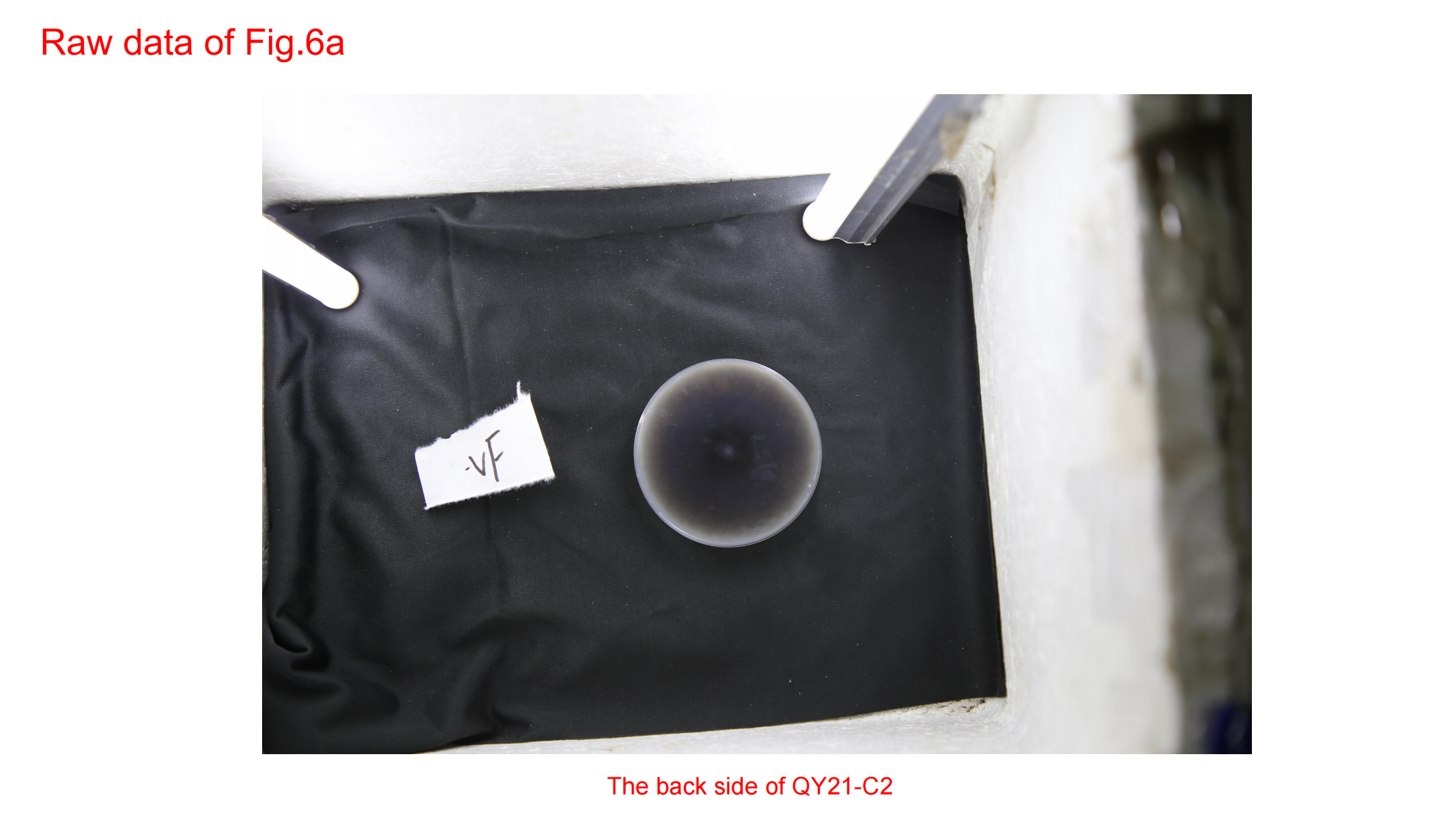

Supplement: Supplementary file 1 [file DataSheet1.zip › Raw images Fig1-6/Fig 6a-1 The back side of QY21-C2.tif]

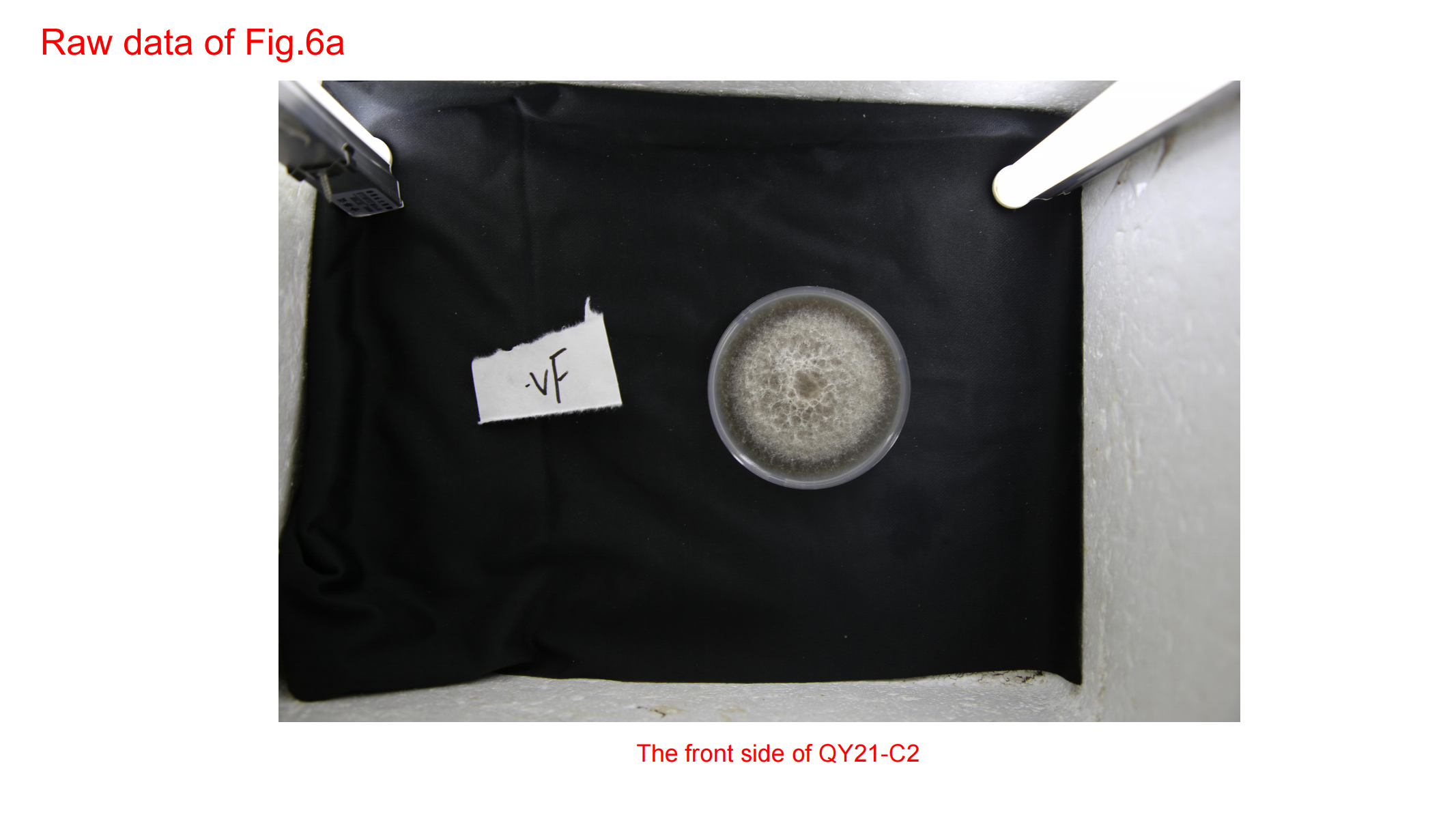

Supplement: Supplementary file 1 [file DataSheet1.zip › Raw images Fig1-6/Fig 6a-2 The front side of QY21-C2.tif]

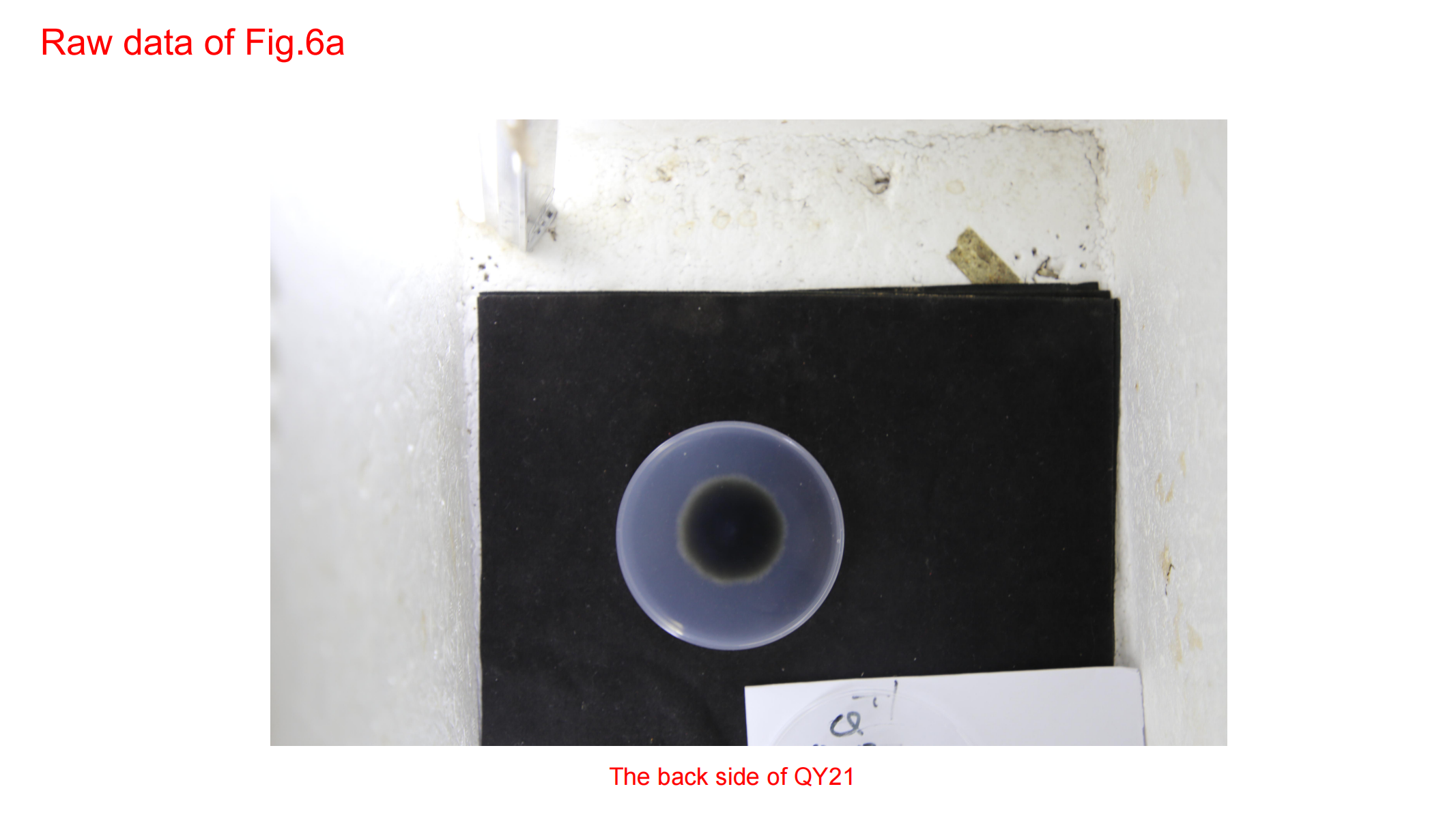

Supplement: Supplementary file 1 [file DataSheet1.zip › Raw images Fig1-6/Fig 6a-3 The back side of QY21.tif]

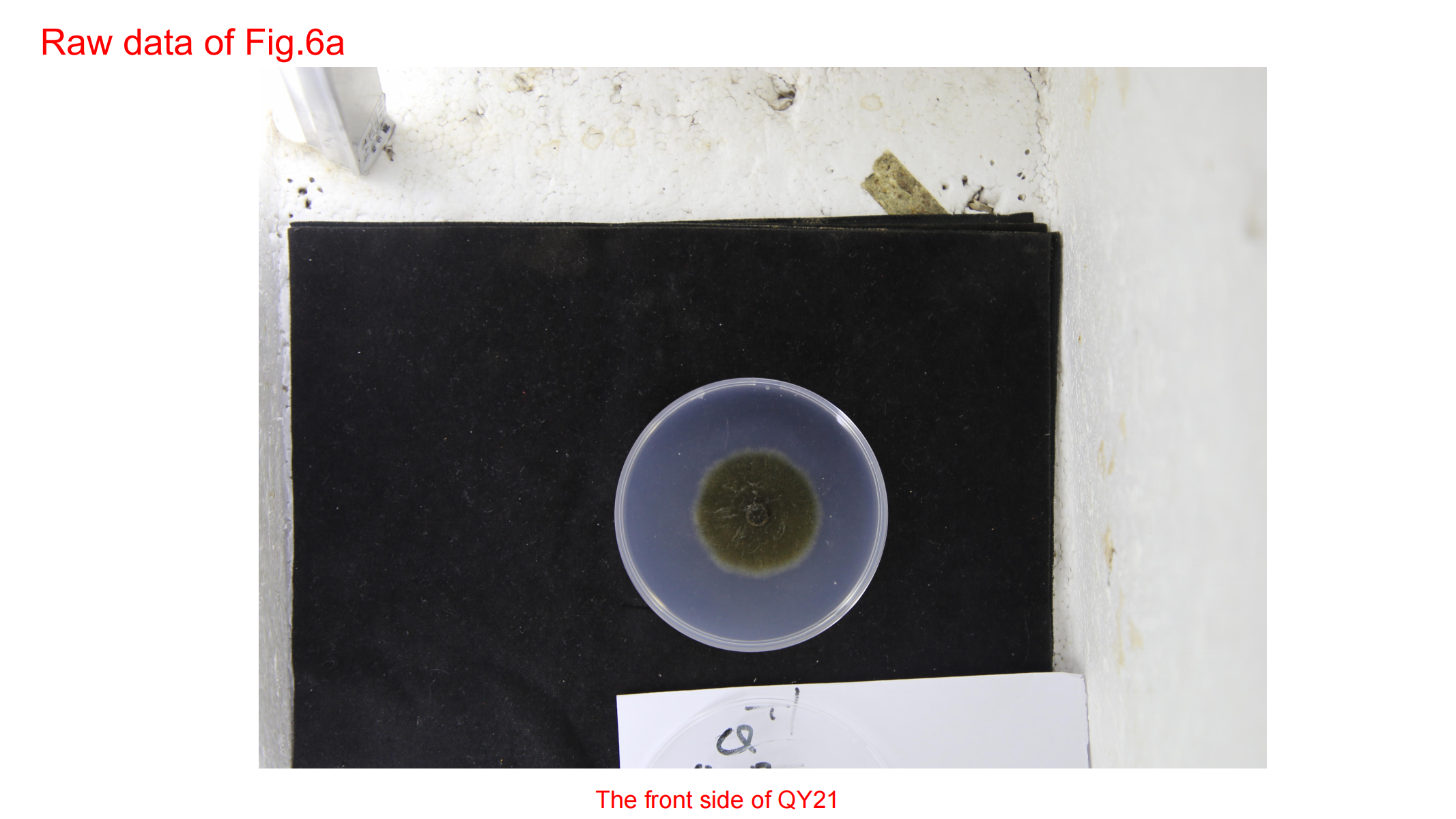

Supplement: Supplementary file 1 [file DataSheet1.zip › Raw images Fig1-6/Fig 6a-4 The front side of QY21.tif]

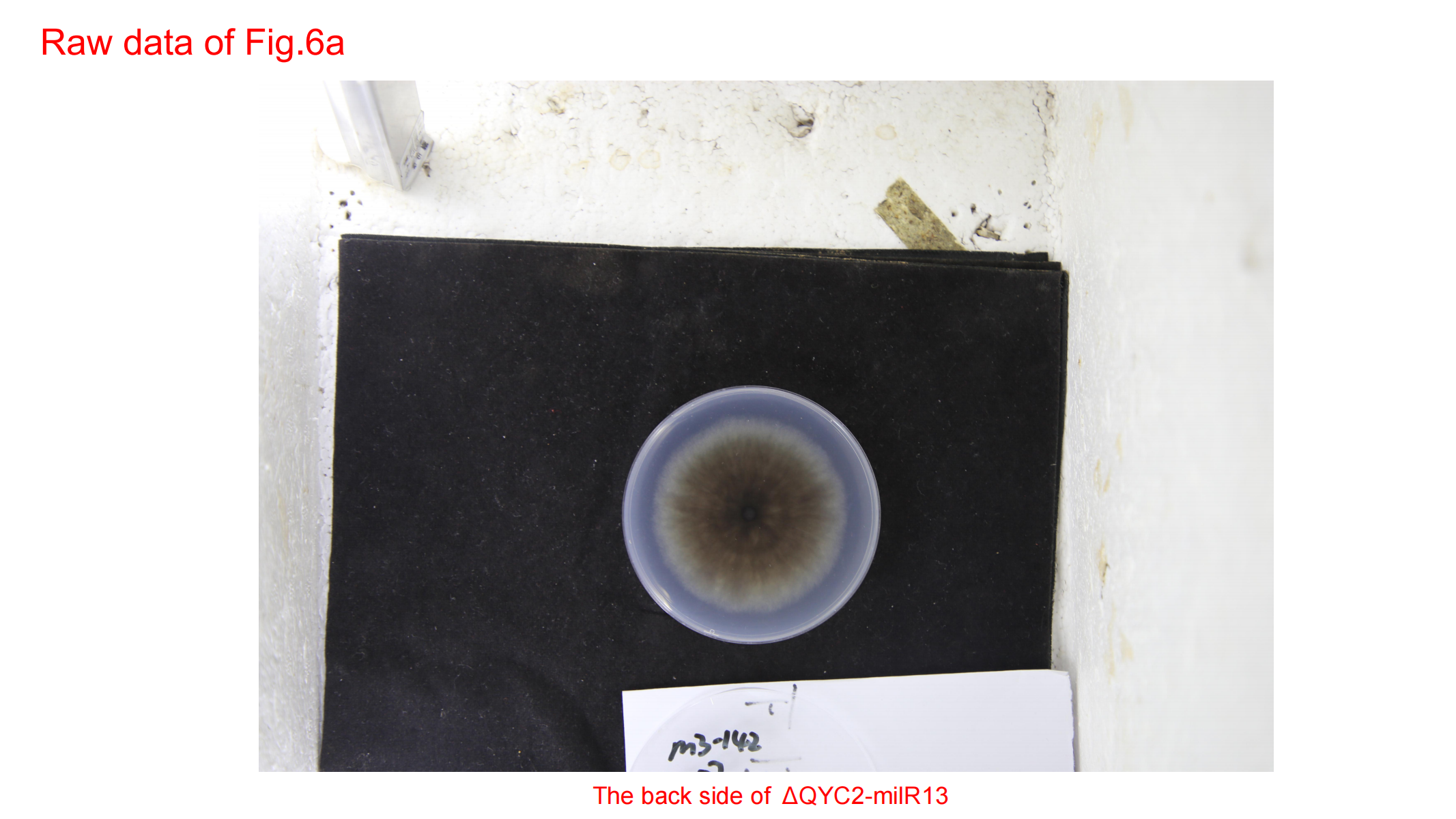

Supplement: Supplementary file 1 [file DataSheet1.zip › Raw images Fig1-6/Fig 6a-5 The back side of ΔQYC2-milR13.tif]

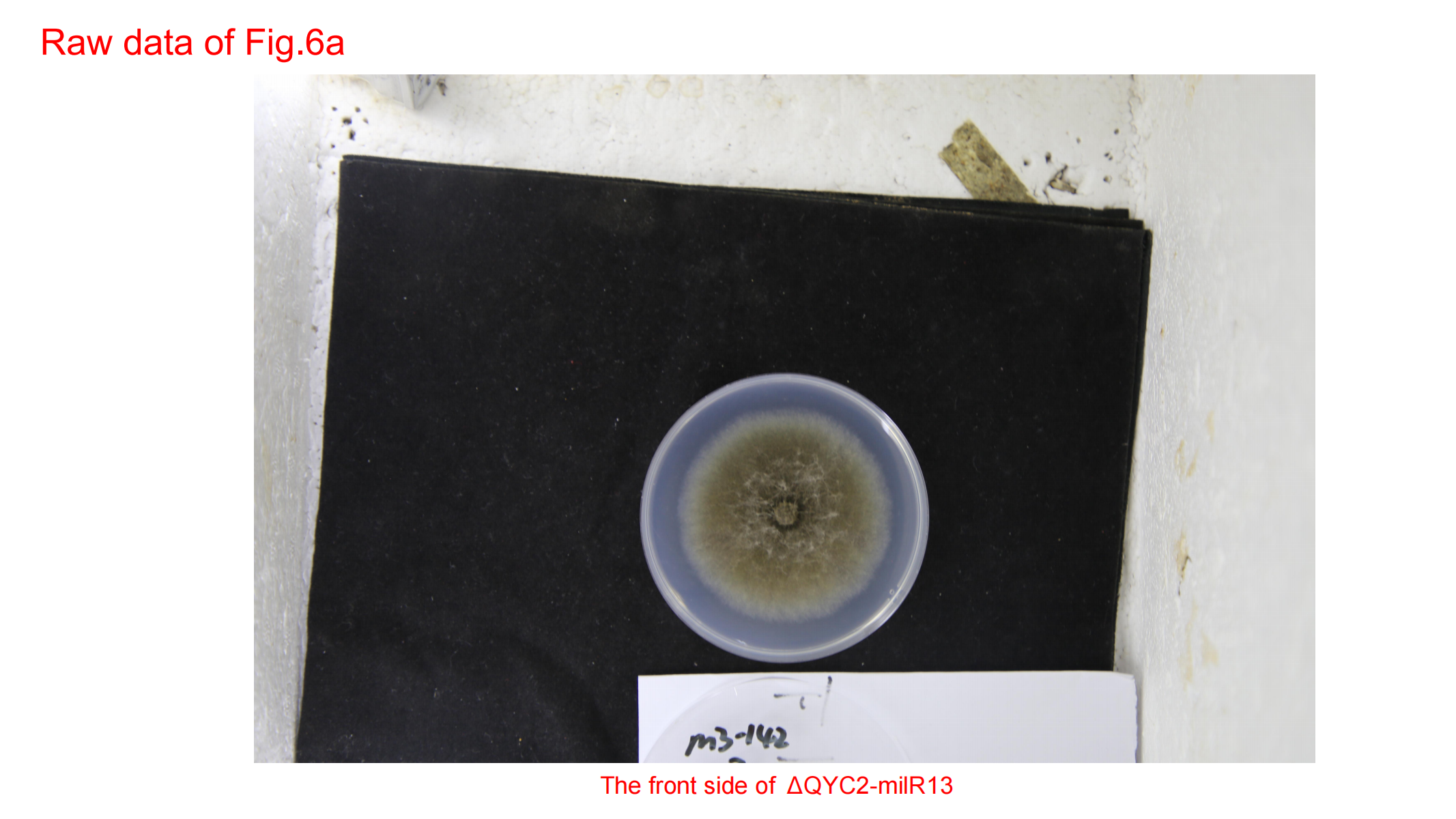

Supplement: Supplementary file 1 [file DataSheet1.zip › Raw images Fig1-6/Fig 6a-6 The front side of ΔQYC2-milR13.tif]

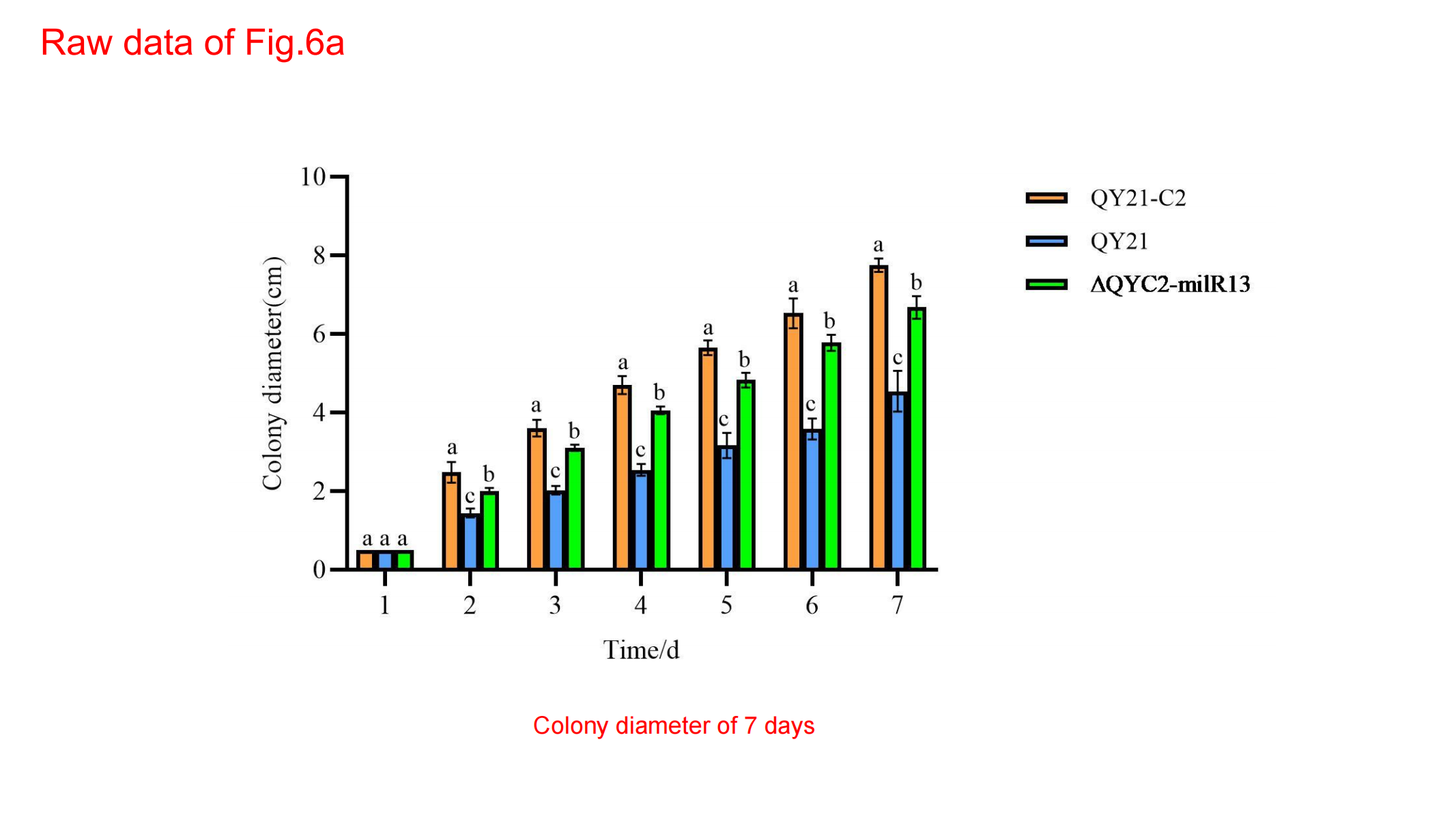

Supplement: Supplementary file 1 [file DataSheet1.zip › Raw images Fig1-6/Fig 6a-7 Colony diameter of 7 days.tif]

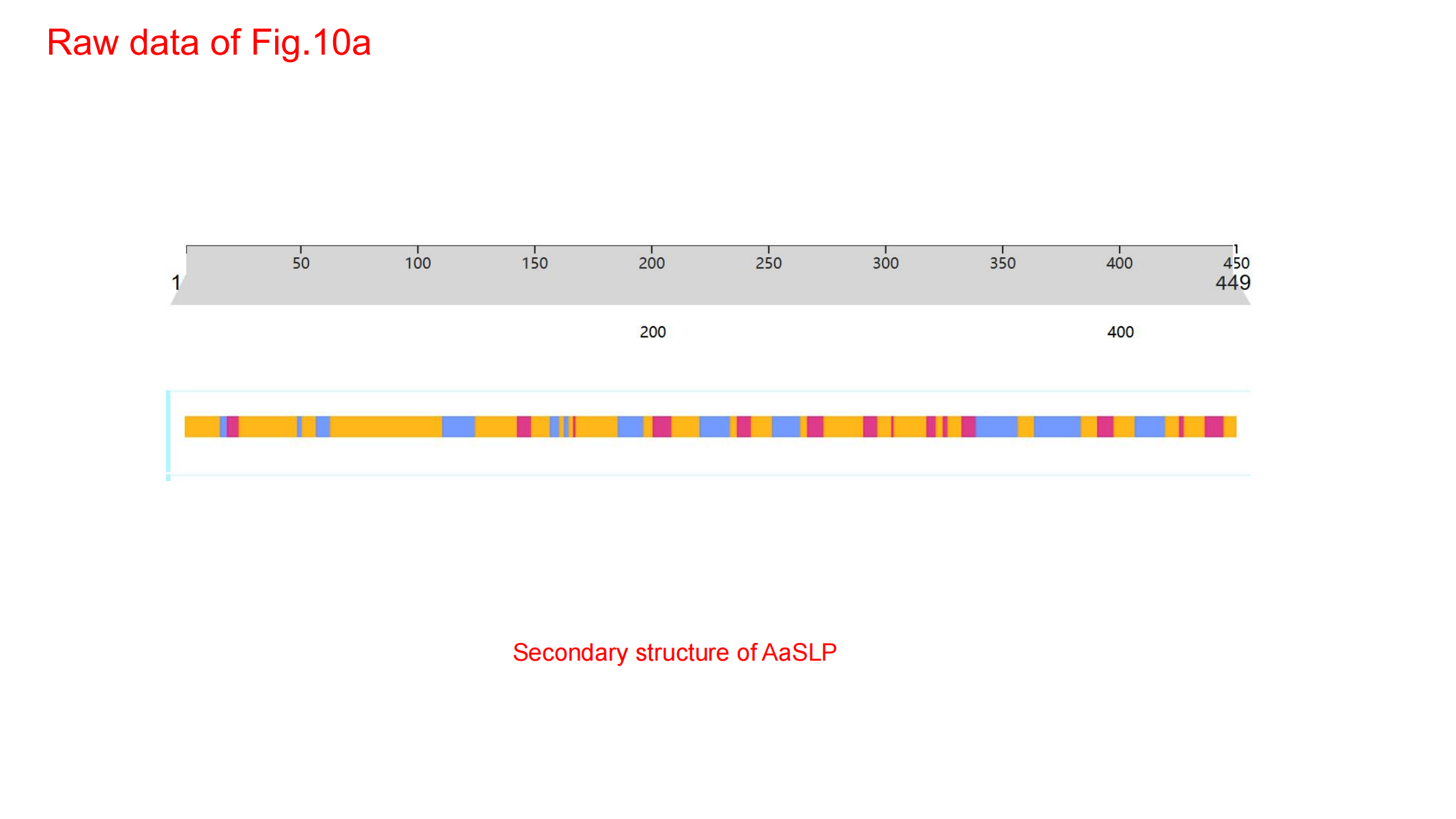

Supplement: Supplementary file 2 [file DataSheet2.zip › Raw images Fig7-10/Fig 10a-1 Secondary structure of AaSLP.tif]

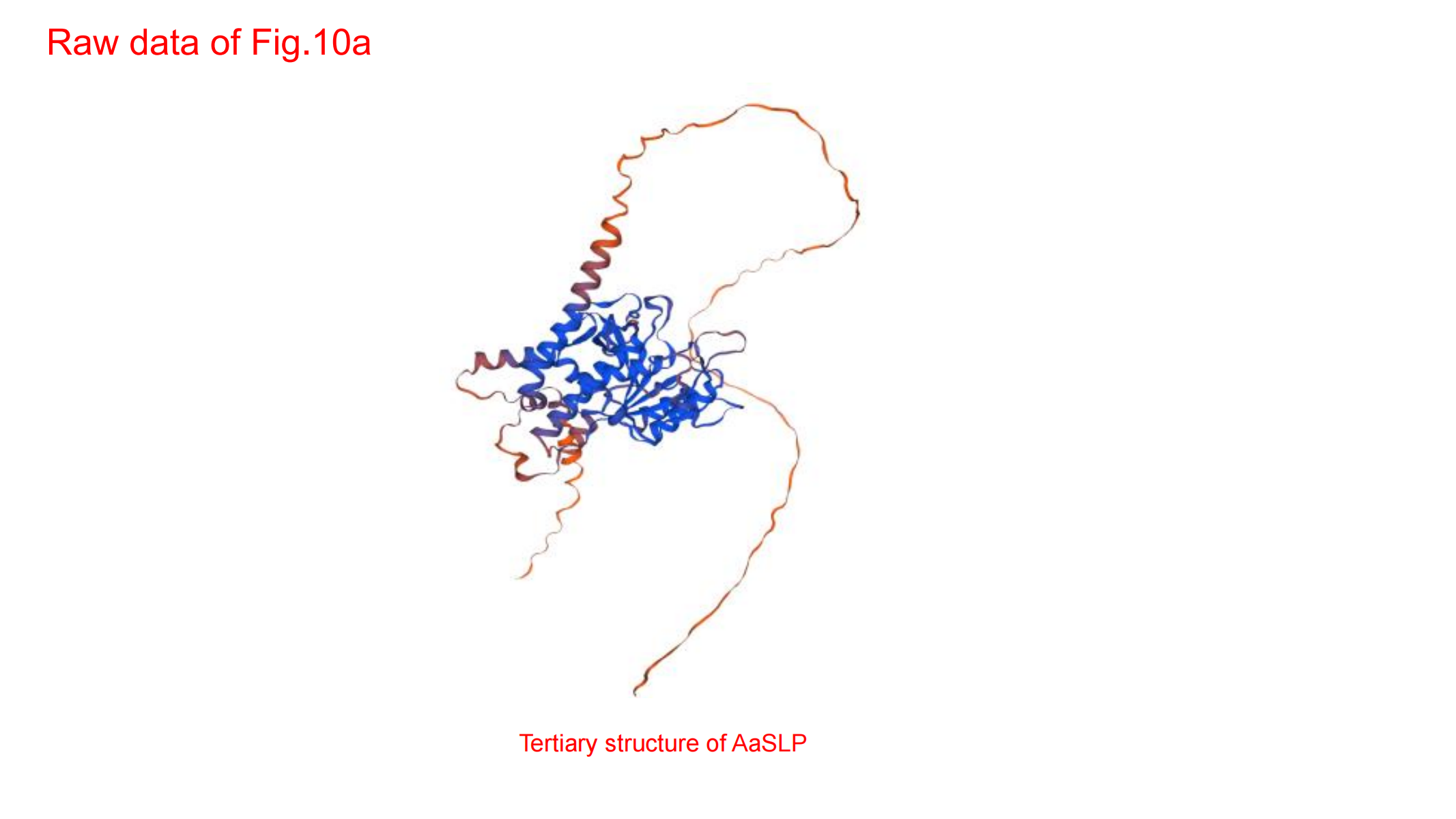

Supplement: Supplementary file 2 [file DataSheet2.zip › Raw images Fig7-10/Fig 10a-2 Tertiary structure of AaSLP.tif]

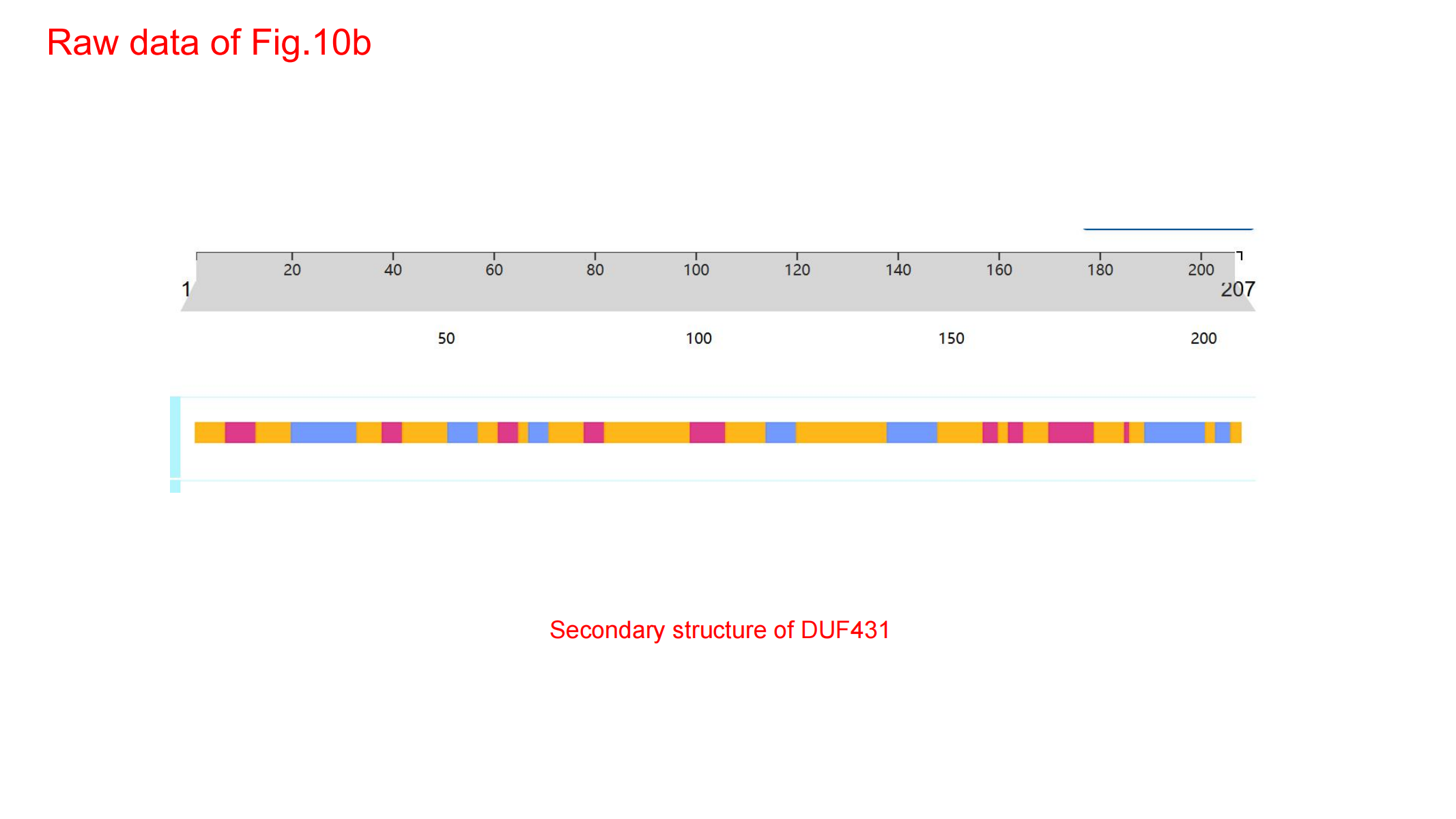

Supplement: Supplementary file 2 [file DataSheet2.zip › Raw images Fig7-10/Fig 10b-1 Secondary structure of DUF431.tif]

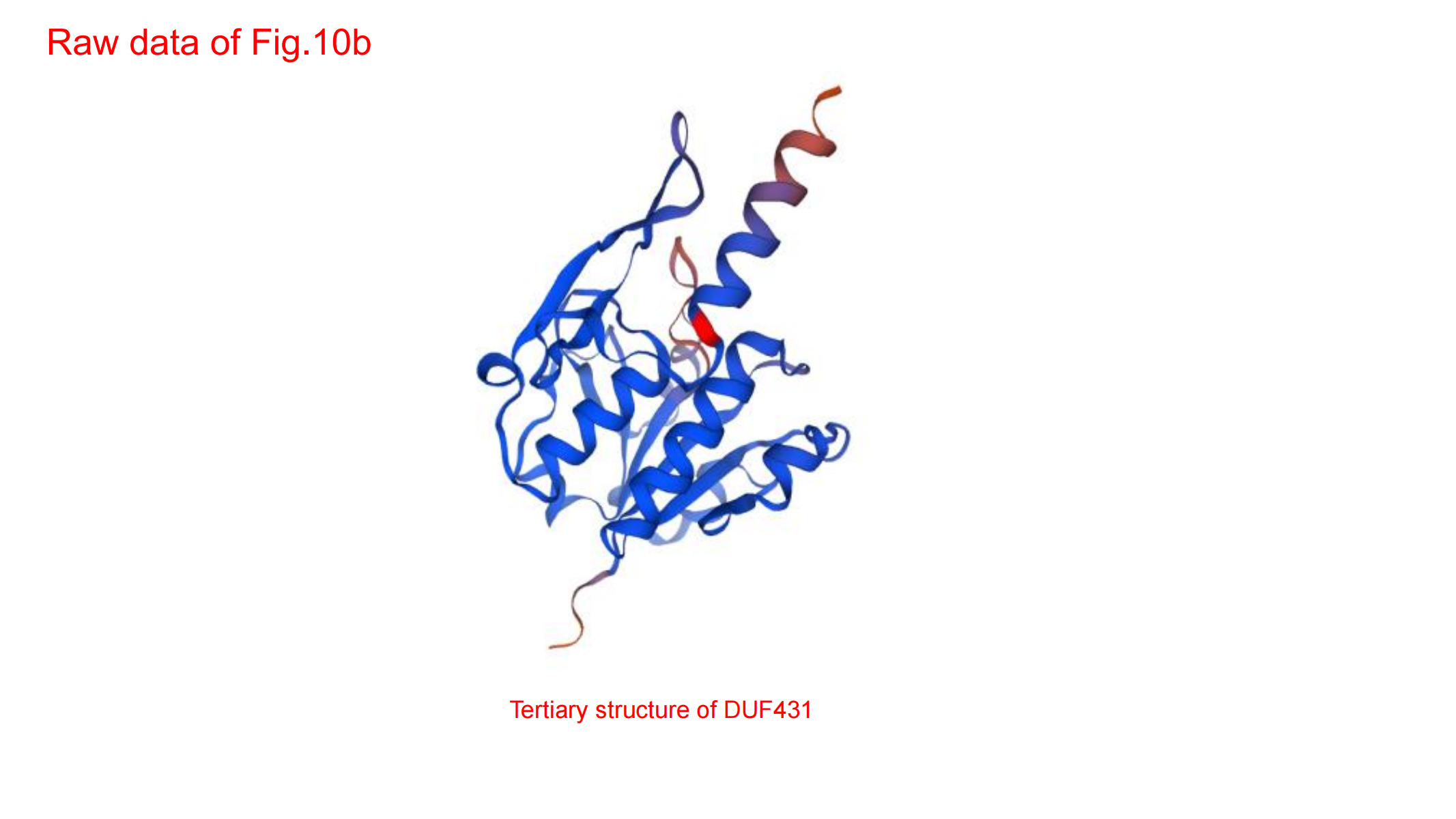

Supplement: Supplementary file 2 [file DataSheet2.zip › Raw images Fig7-10/Fig 10b-2 Tertiary structure of DUF431.tif]

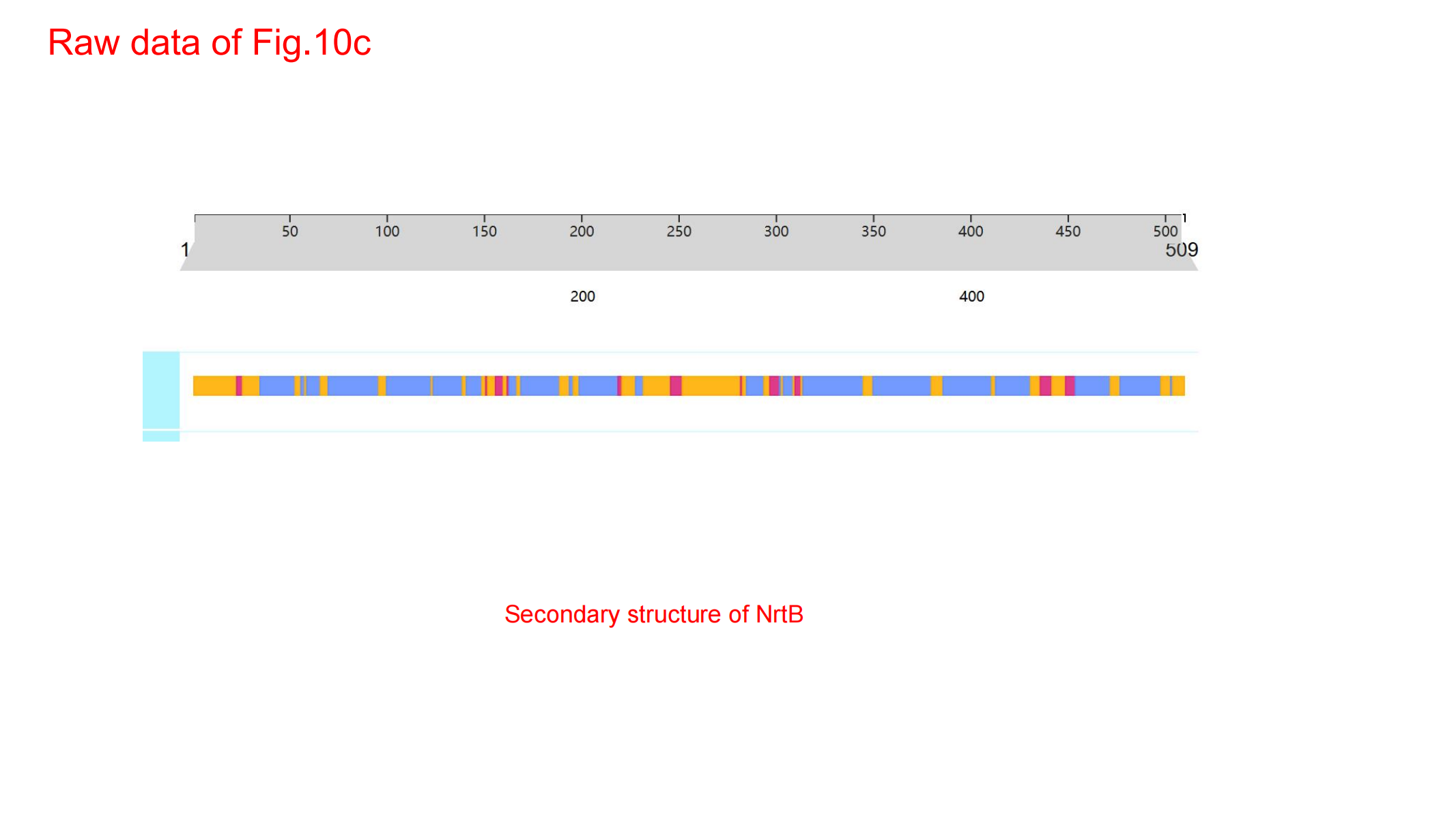

Supplement: Supplementary file 2 [file DataSheet2.zip › Raw images Fig7-10/Fig 10c-1 Secondary structure of NrtB.tif]

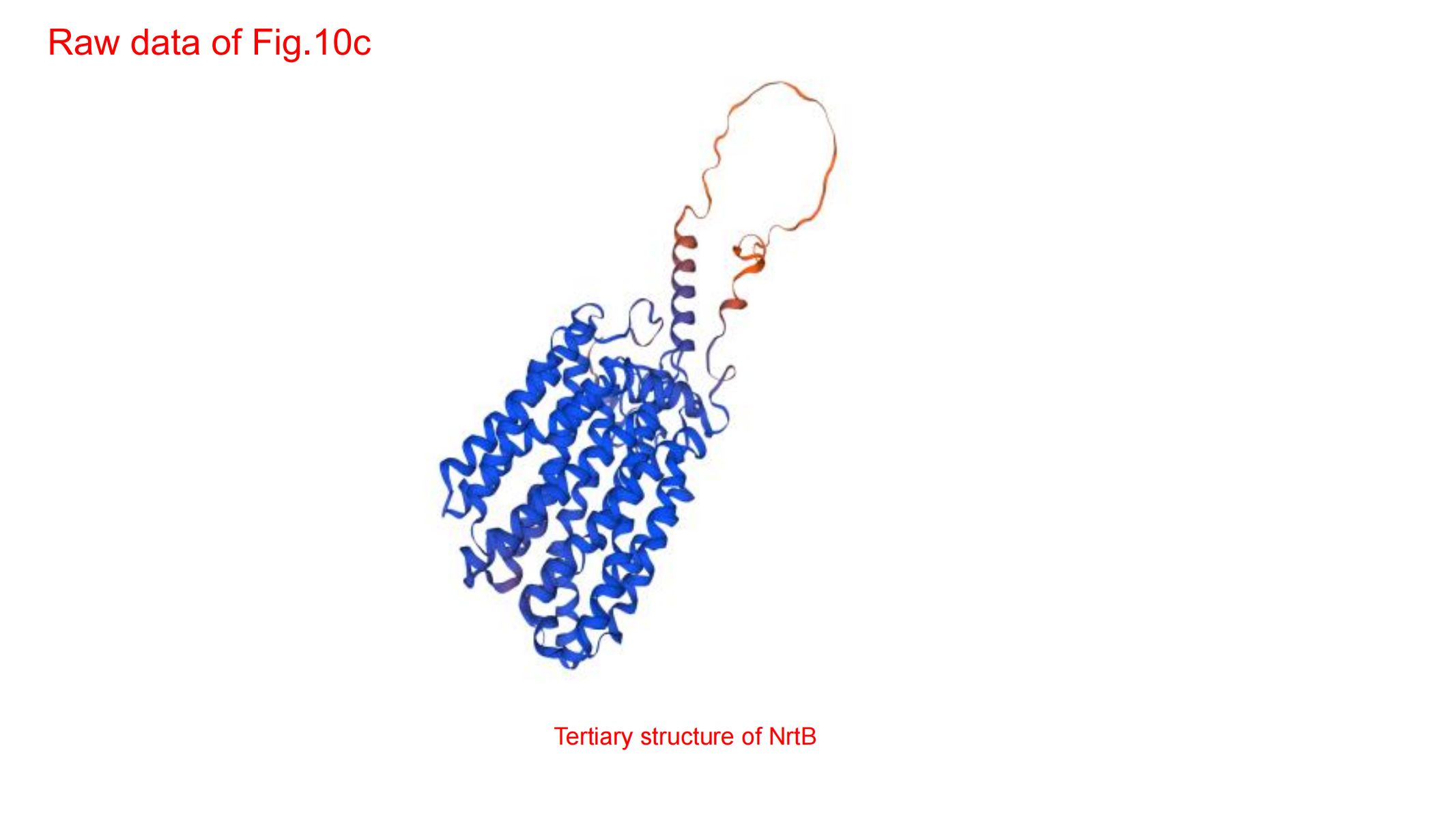

Supplement: Supplementary file 2 [file DataSheet2.zip › Raw images Fig7-10/Fig 10c-2 Tertiary structure of NrtB.tif]

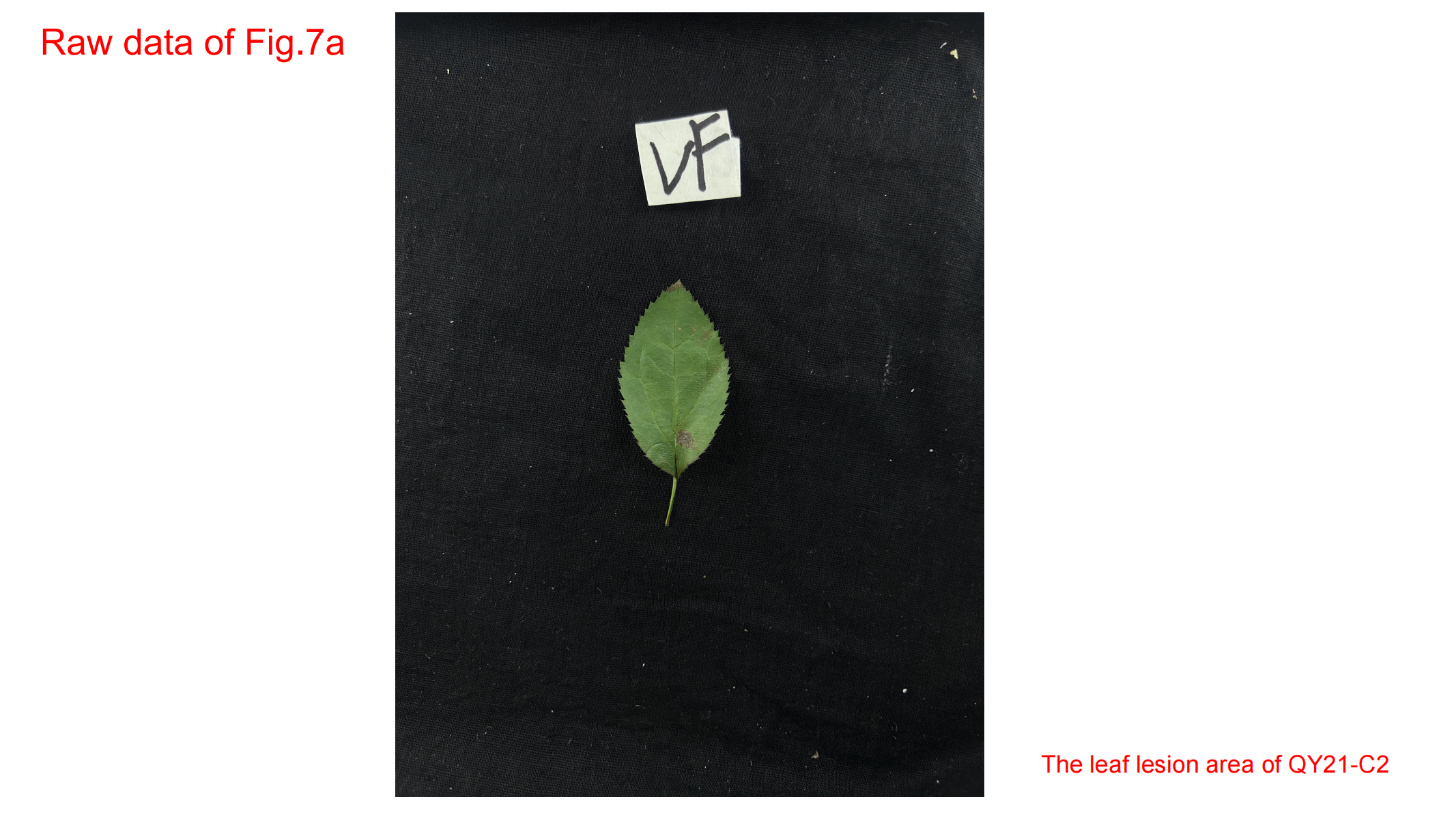

Supplement: Supplementary file 2 [file DataSheet2.zip › Raw images Fig7-10/Fig 7a-1 The leaf lesion area of QY21-C2.tif]

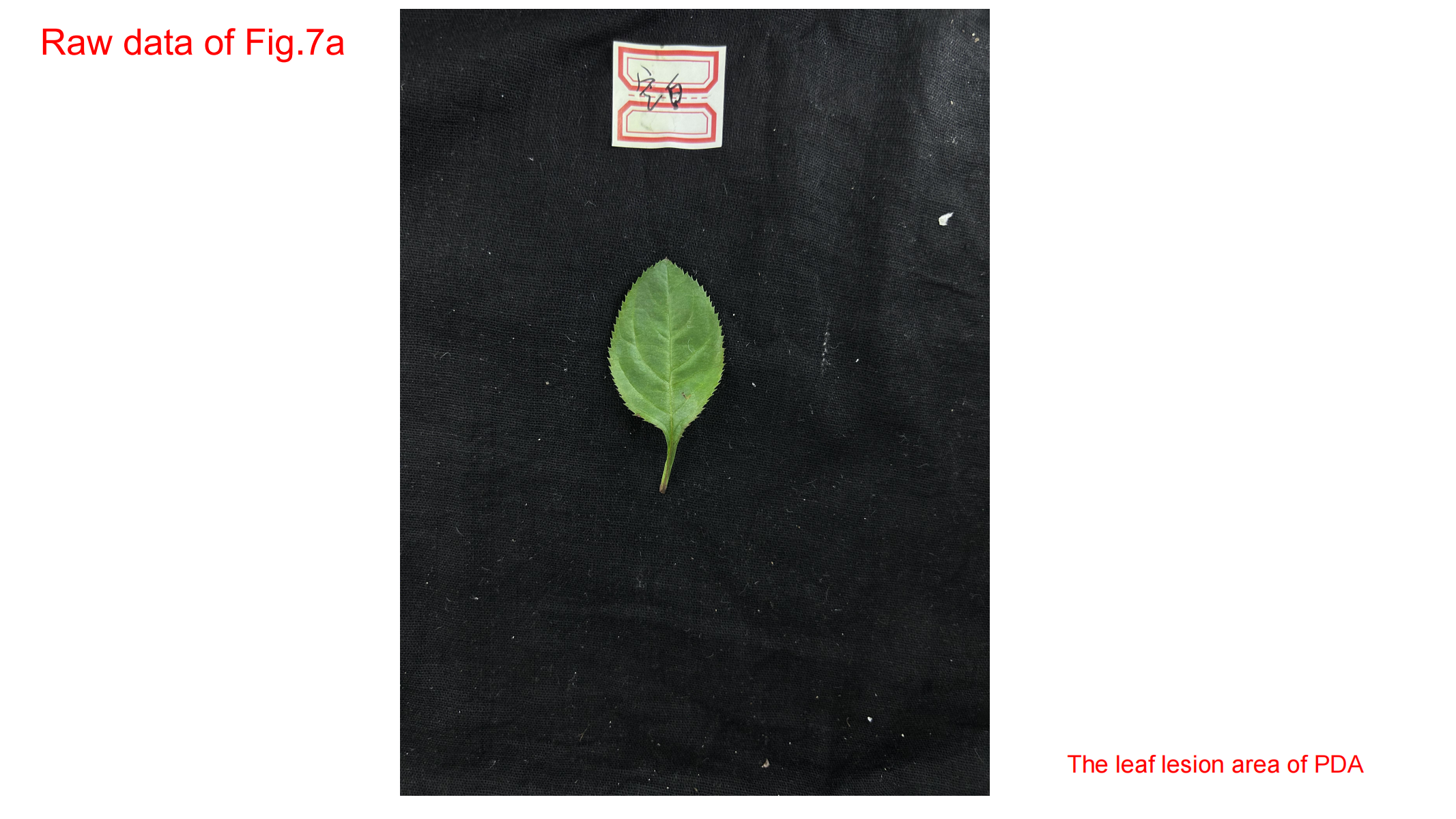

Supplement: Supplementary file 2 [file DataSheet2.zip › Raw images Fig7-10/Fig 7a-2 The leaf lesion area of PDA.tif]

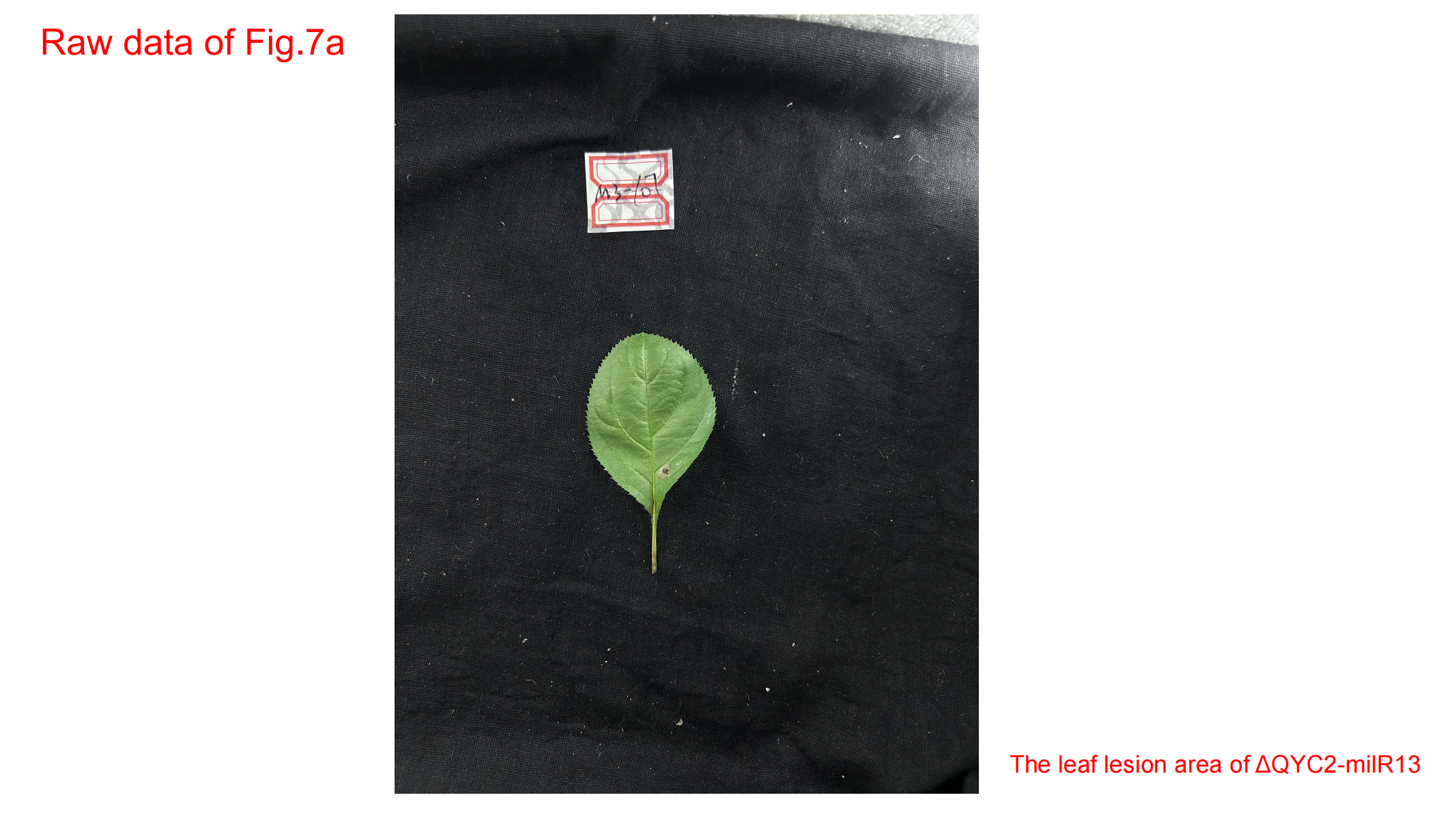

Supplement: Supplementary file 2 [file DataSheet2.zip › Raw images Fig7-10/Fig 7a-3 The leaf lesion area of ΔQYC2-milR13.tif]

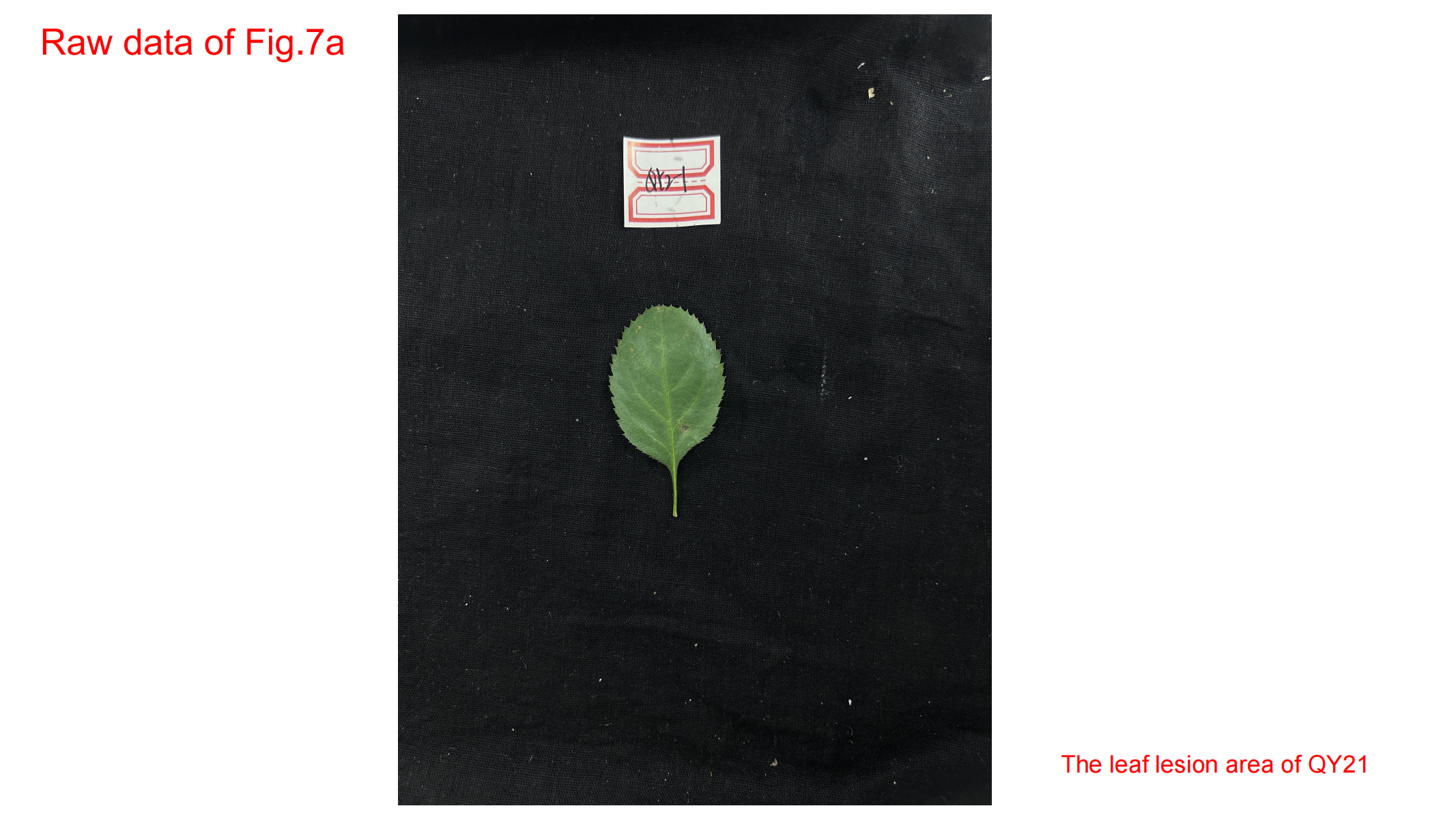

Supplement: Supplementary file 2 [file DataSheet2.zip › Raw images Fig7-10/Fig 7a-4 The leaf lesion area of QY21.tif]

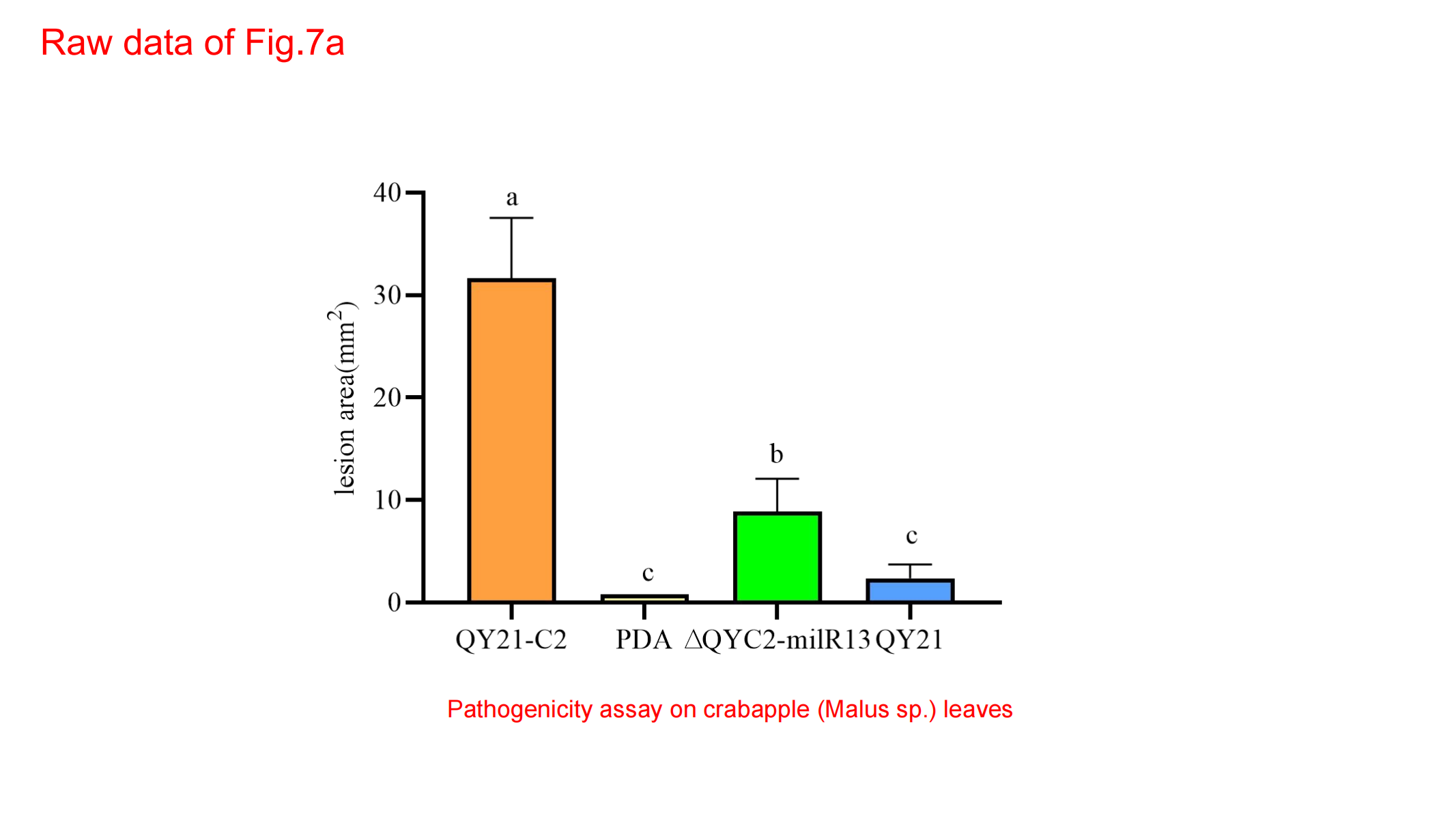

Supplement: Supplementary file 2 [file DataSheet2.zip › Raw images Fig7-10/Fig 7a-5 Pathogenicity assay on crabapple (Malus sp.) leaves.tif]

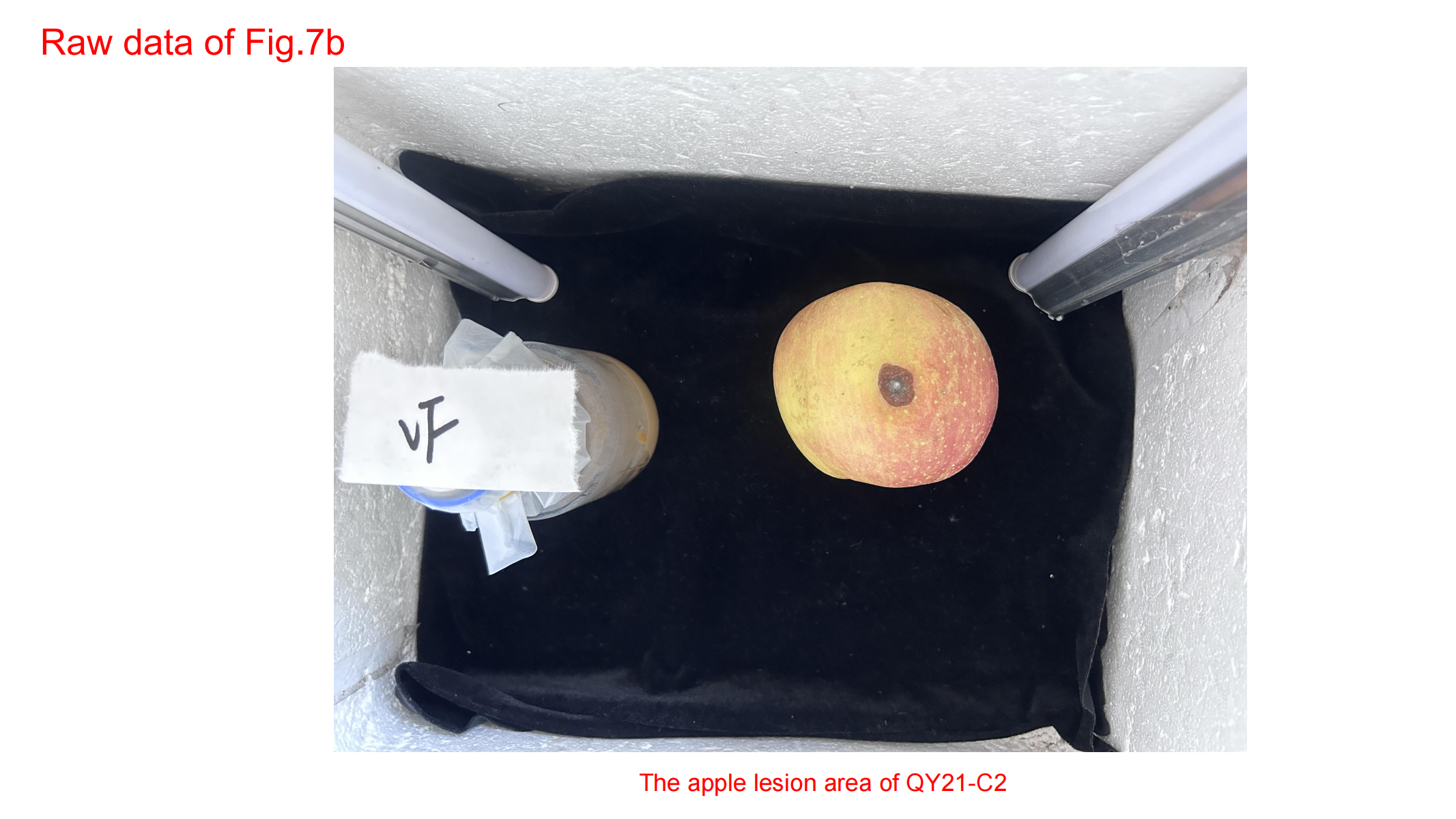

Supplement: Supplementary file 2 [file DataSheet2.zip › Raw images Fig7-10/Fig 7b-1 The apple lesion area of QY21-C2.tif]

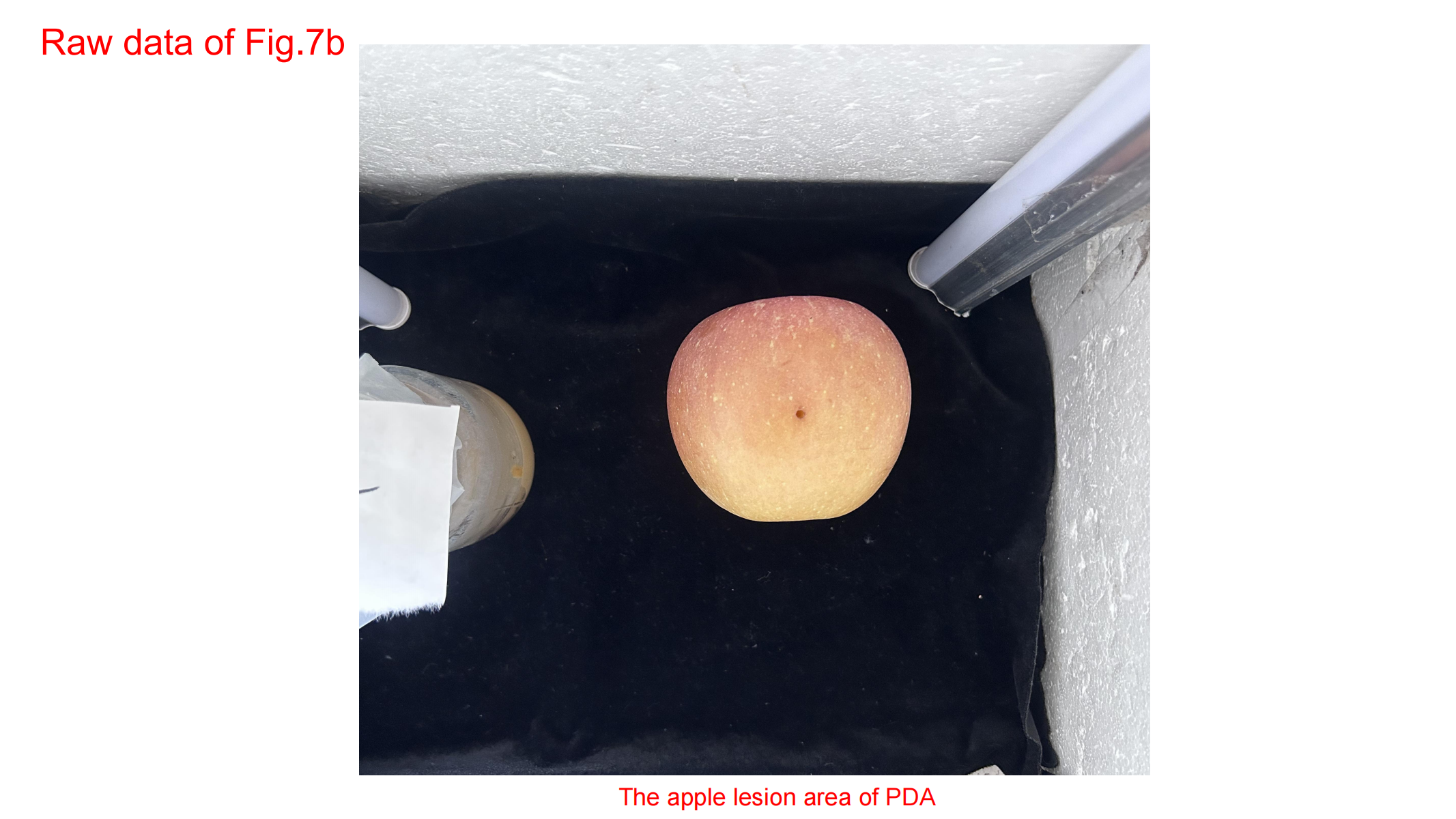

Supplement: Supplementary file 2 [file DataSheet2.zip › Raw images Fig7-10/Fig 7b-2 The apple lesion area of PDA.tif]

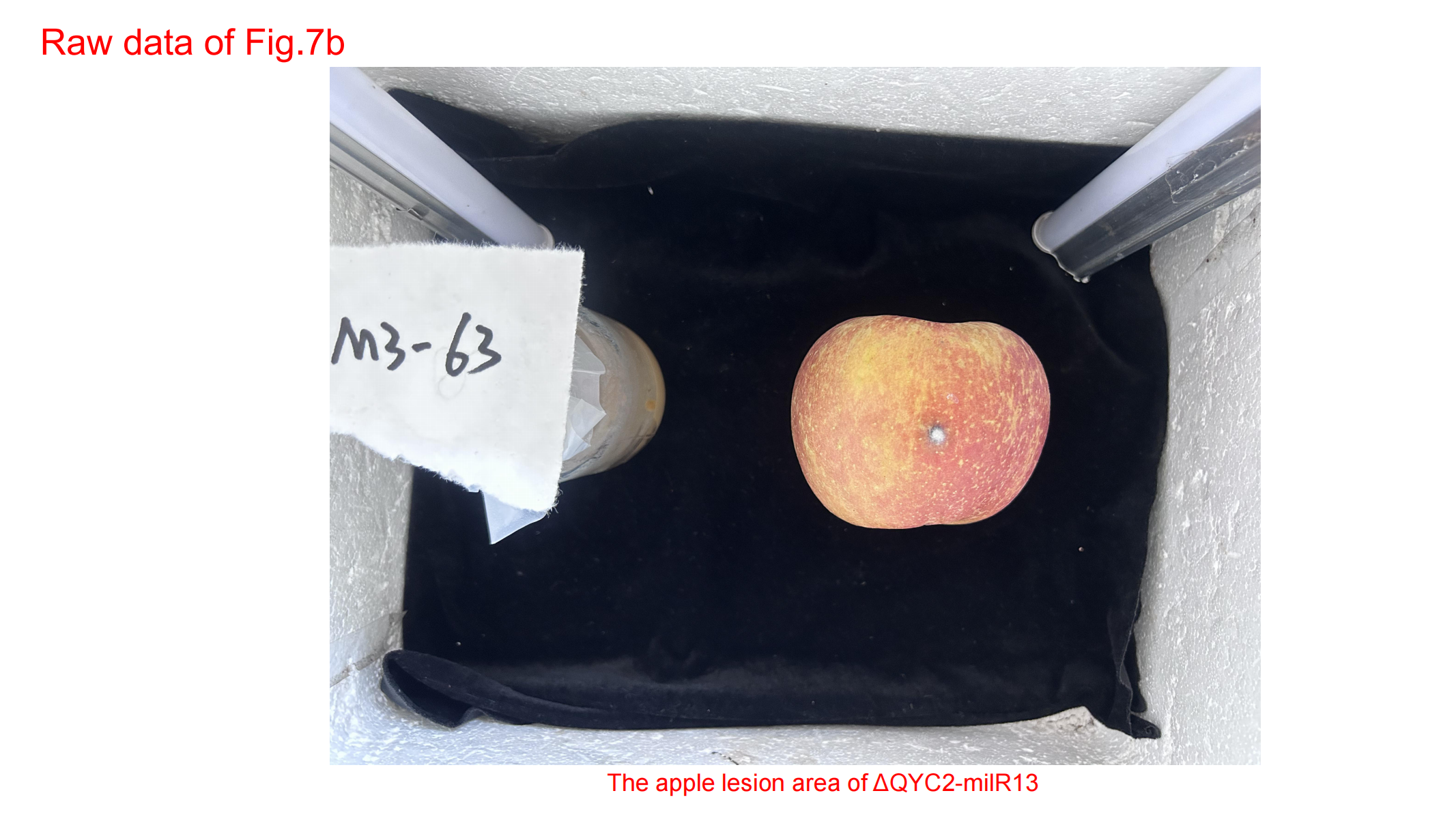

Supplement: Supplementary file 2 [file DataSheet2.zip › Raw images Fig7-10/Fig 7b-3 The apple lesion area of ΔQYC2-milR13.tif]

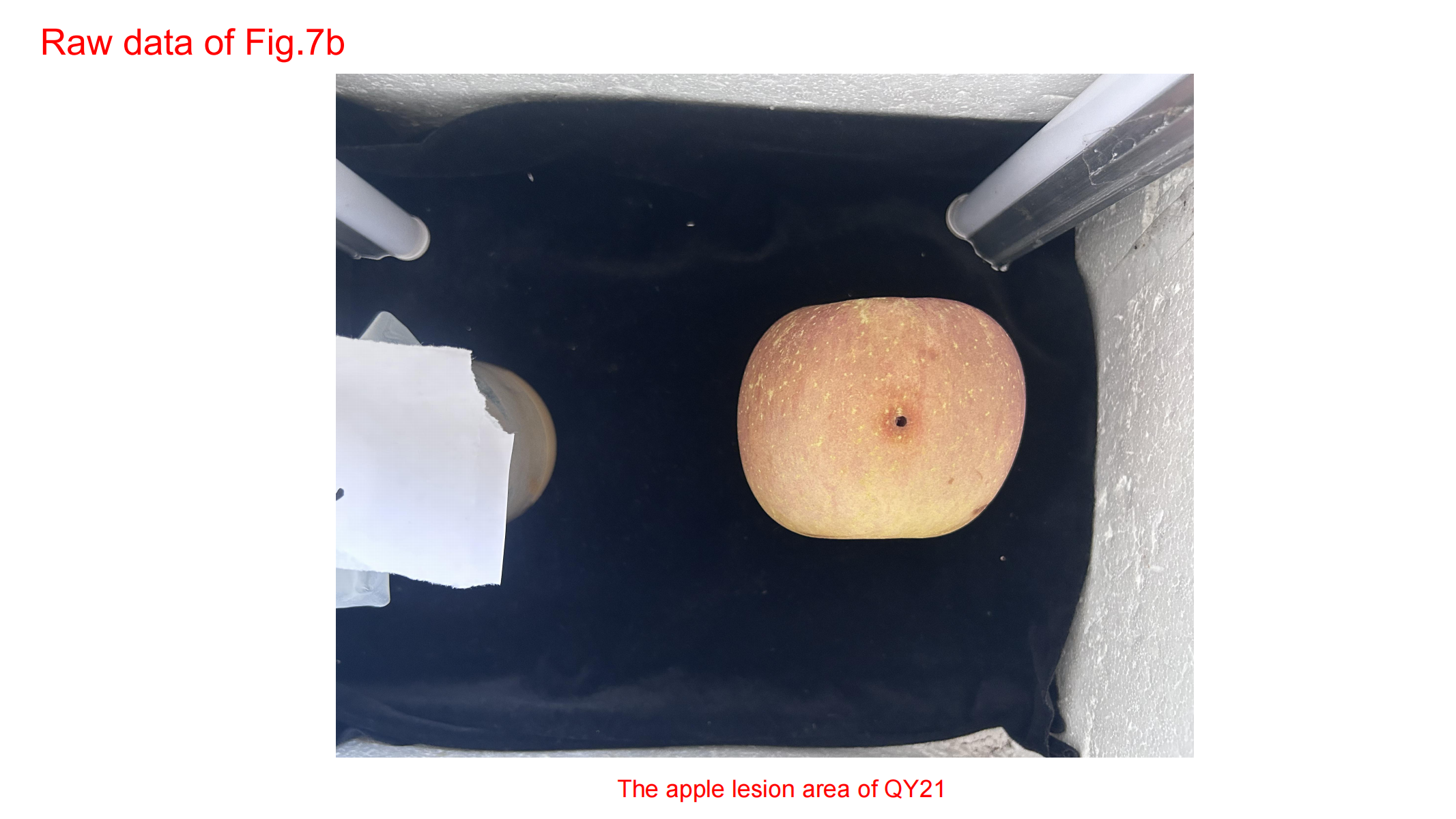

Supplement: Supplementary file 2 [file DataSheet2.zip › Raw images Fig7-10/Fig 7b-4 The apple lesion area of QY21.tif]

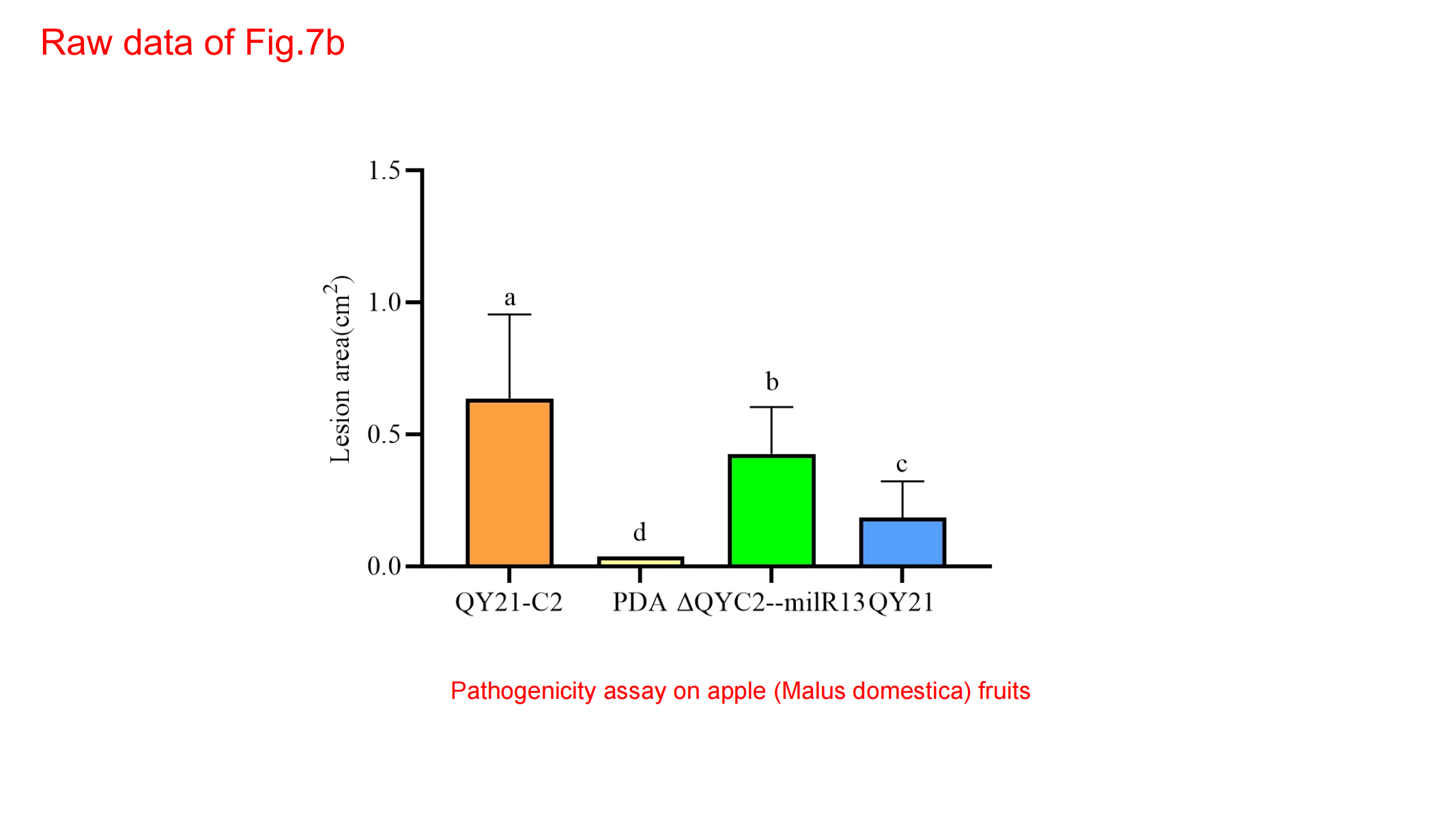

Supplement: Supplementary file 2 [file DataSheet2.zip › Raw images Fig7-10/Fig 7b-5 Pathogenicity assay on apple (Malus domestica) fruits.tif]

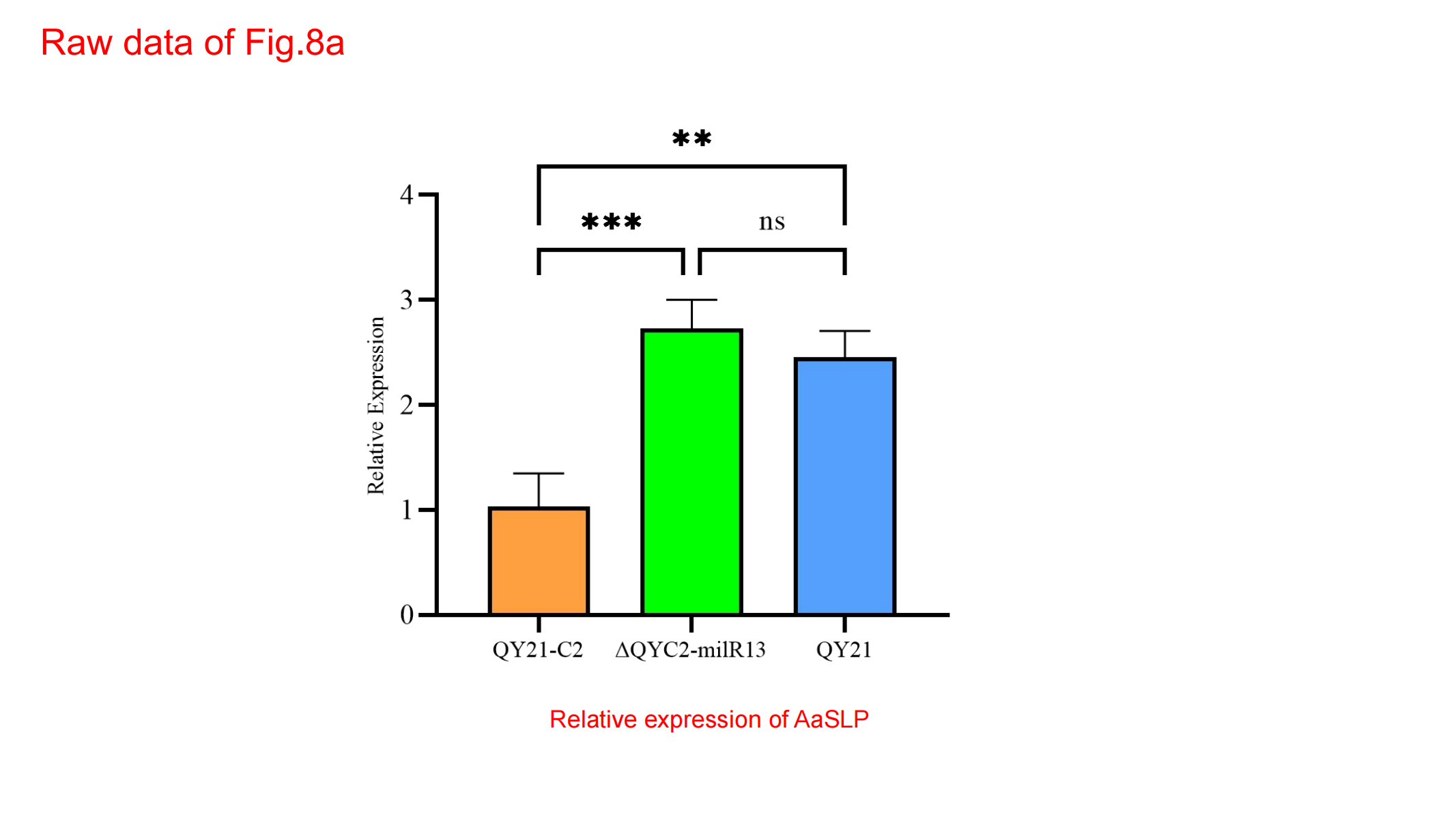

Supplement: Supplementary file 2 [file DataSheet2.zip › Raw images Fig7-10/Fig 8a Relative expression of AaSLP.tif]

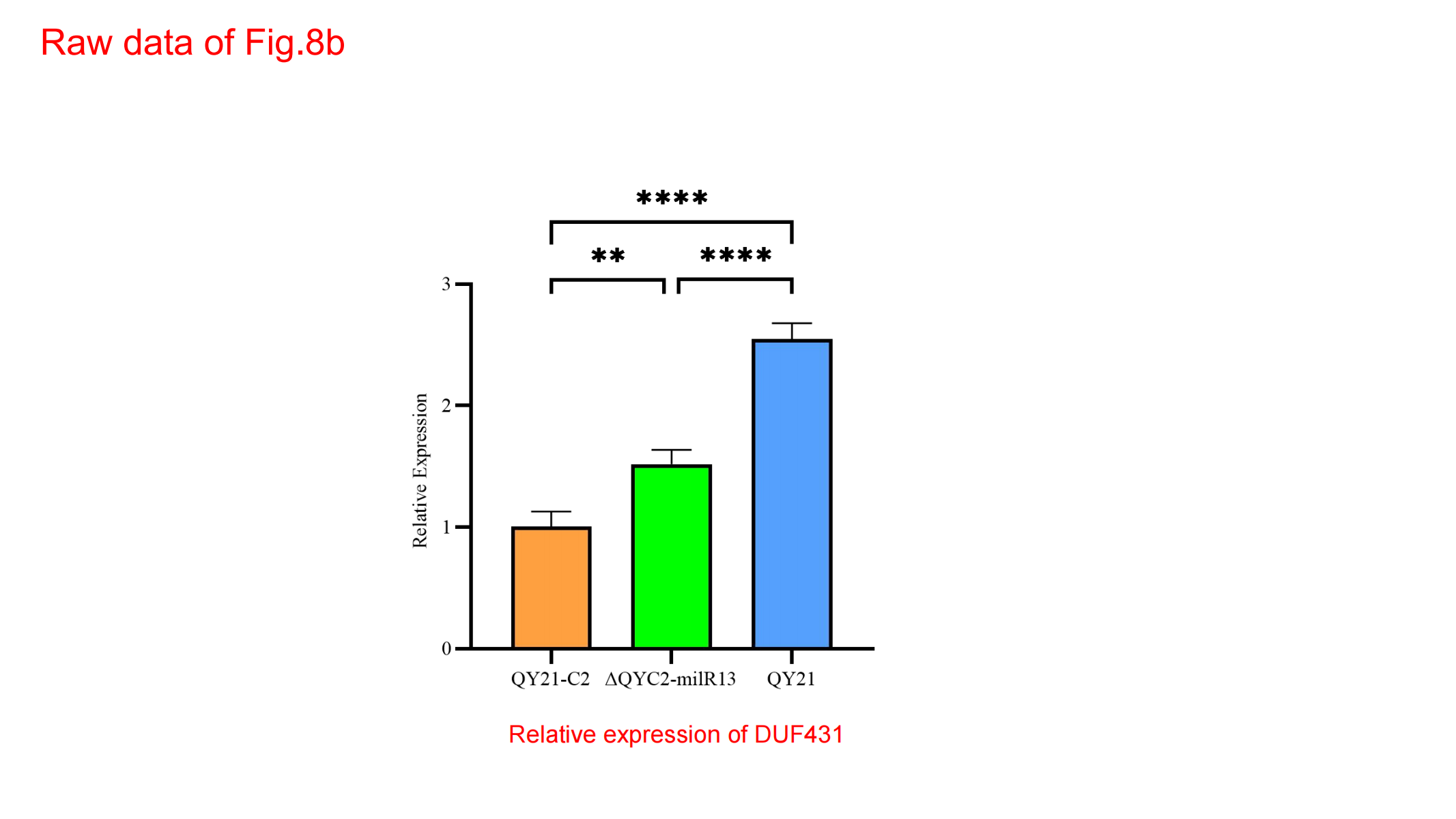

Supplement: Supplementary file 2 [file DataSheet2.zip › Raw images Fig7-10/Fig 8b Relative expression of DUF431.tif]

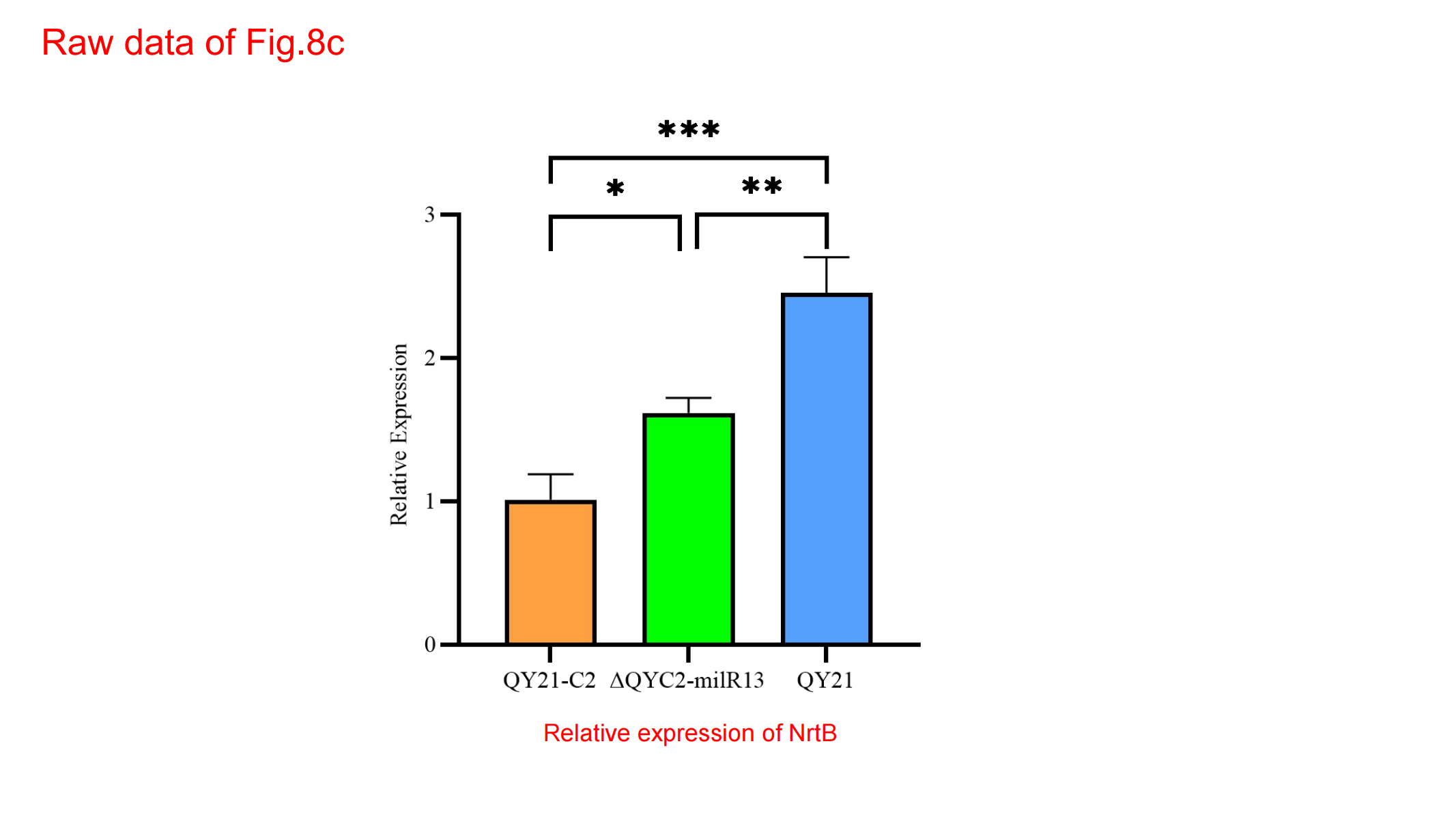

Supplement: Supplementary file 2 [file DataSheet2.zip › Raw images Fig7-10/Fig 8c Relative expression of NrtB.tif]

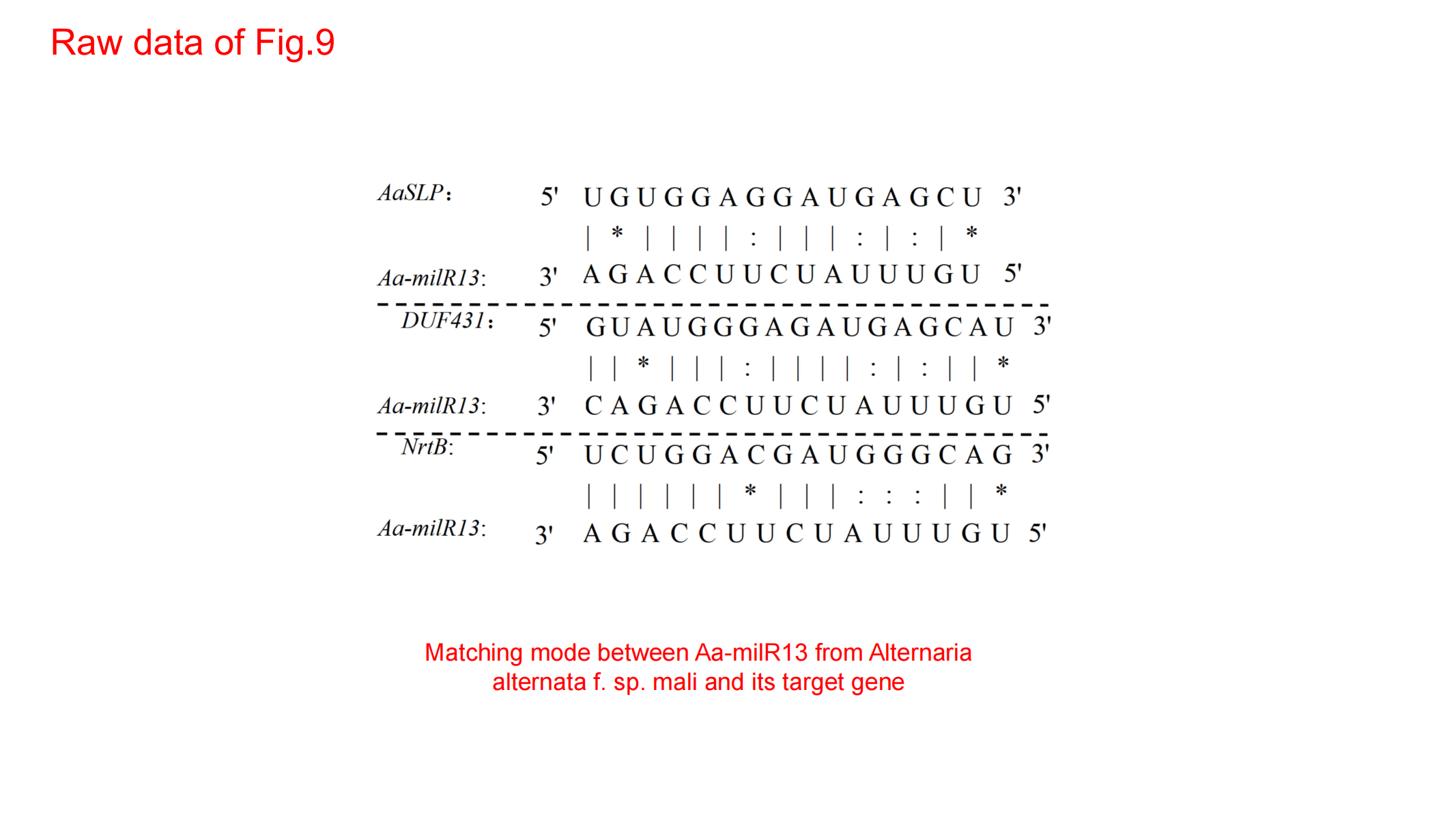

Supplement: Supplementary file 2 [file DataSheet2.zip › Raw images Fig7-10/Fig 9 Matching mode between Aa-milR13 from Alternaria alternata f. sp. mali and its target gene.tif]

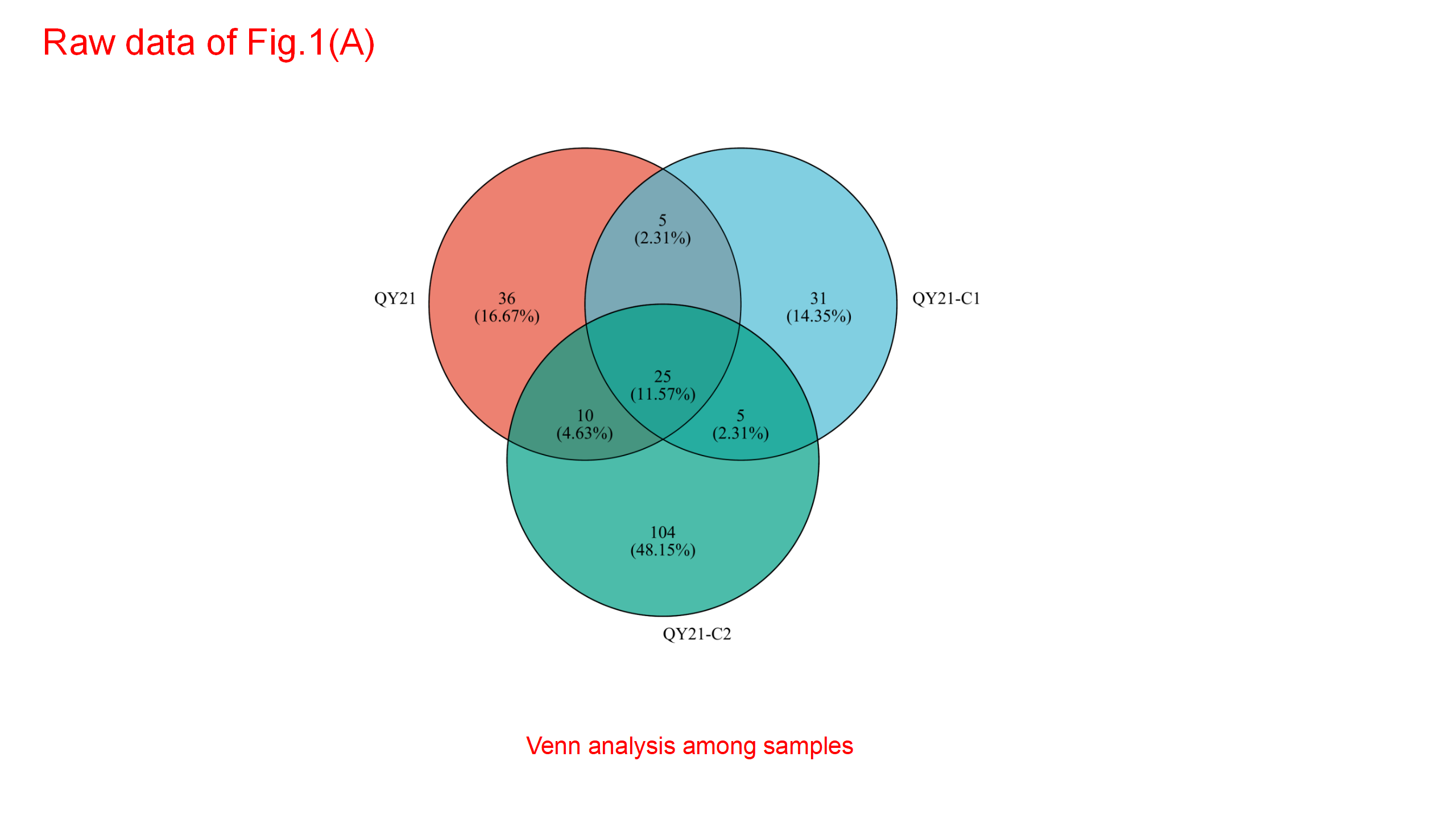

Supplement: Supplementary file 3 [file DataSheet3.zip › New Raw Images Fig1-6/New Fig.1 (A) Venn analysis among samples.tif]

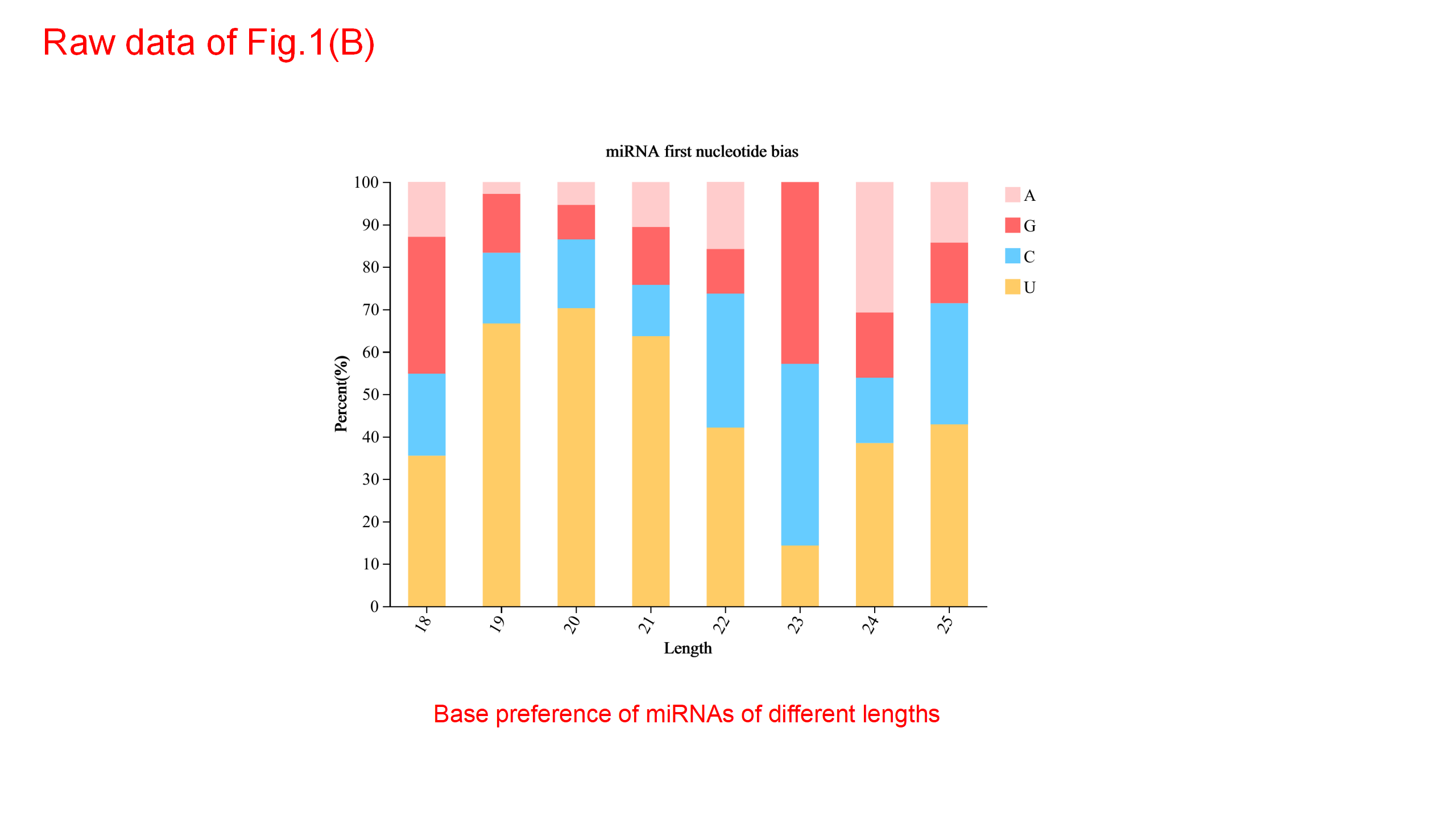

Supplement: Supplementary file 3 [file DataSheet3.zip › New Raw Images Fig1-6/New Fig.1 (B) Base preference of miRNAs of different lengths.tif]

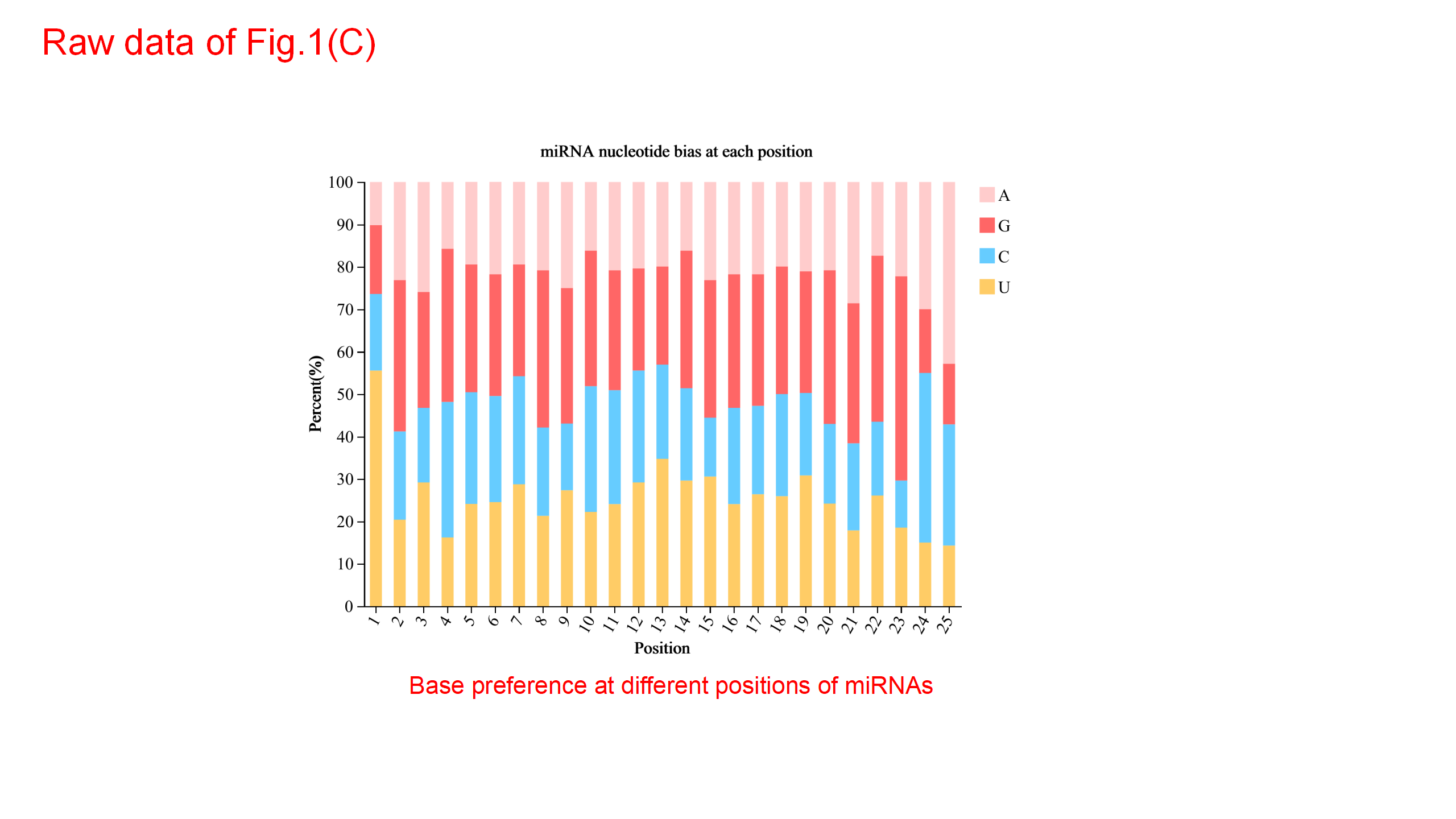

Supplement: Supplementary file 3 [file DataSheet3.zip › New Raw Images Fig1-6/New Fig.1 (C) Base preference at different positions of miRNAs.tif]

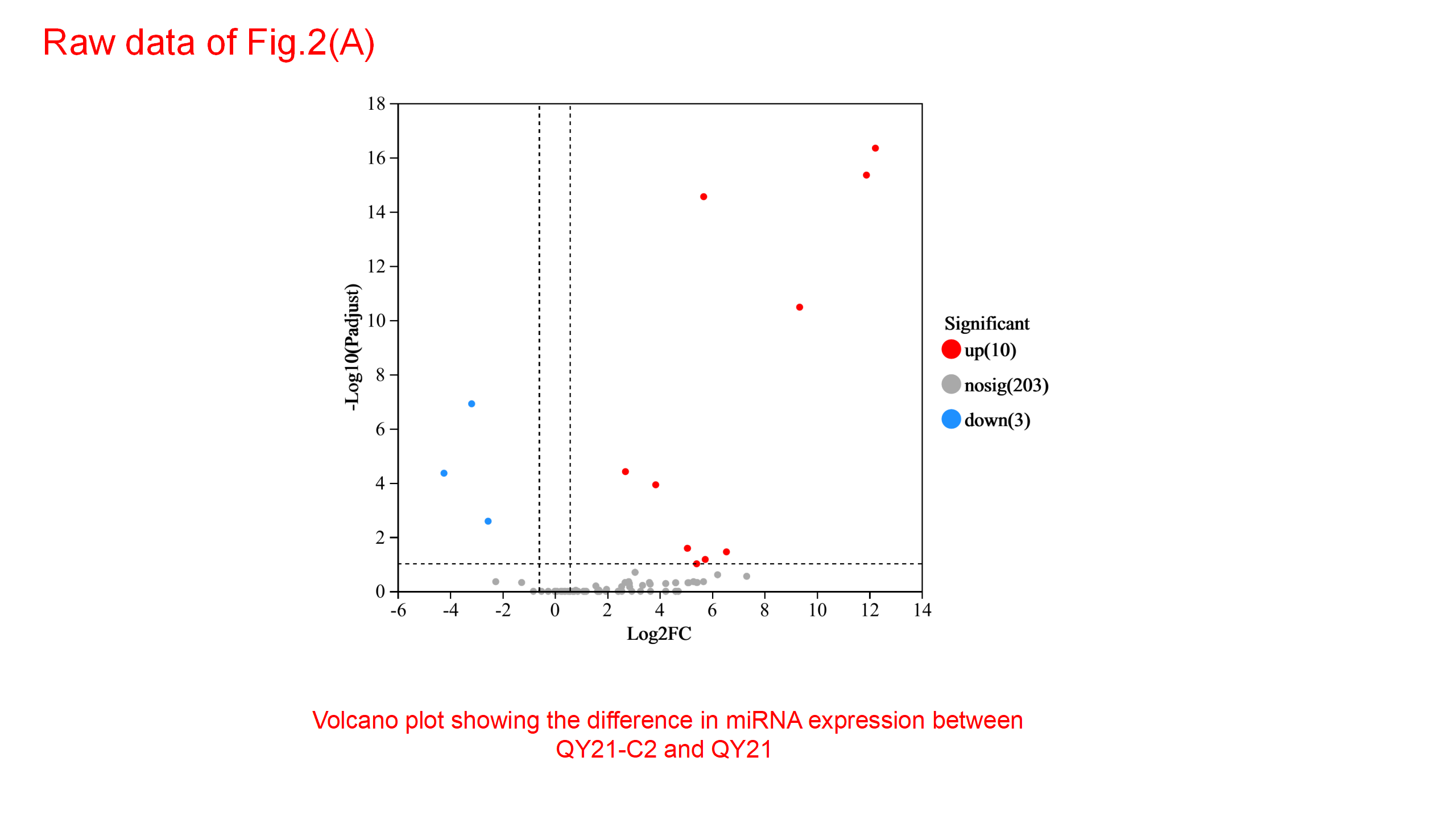

Supplement: Supplementary file 3 [file DataSheet3.zip › New Raw Images Fig1-6/New Fig.2 (A) Volcano plot showing the difference in miRNA expression between QY21-C2 and QY21.tif]

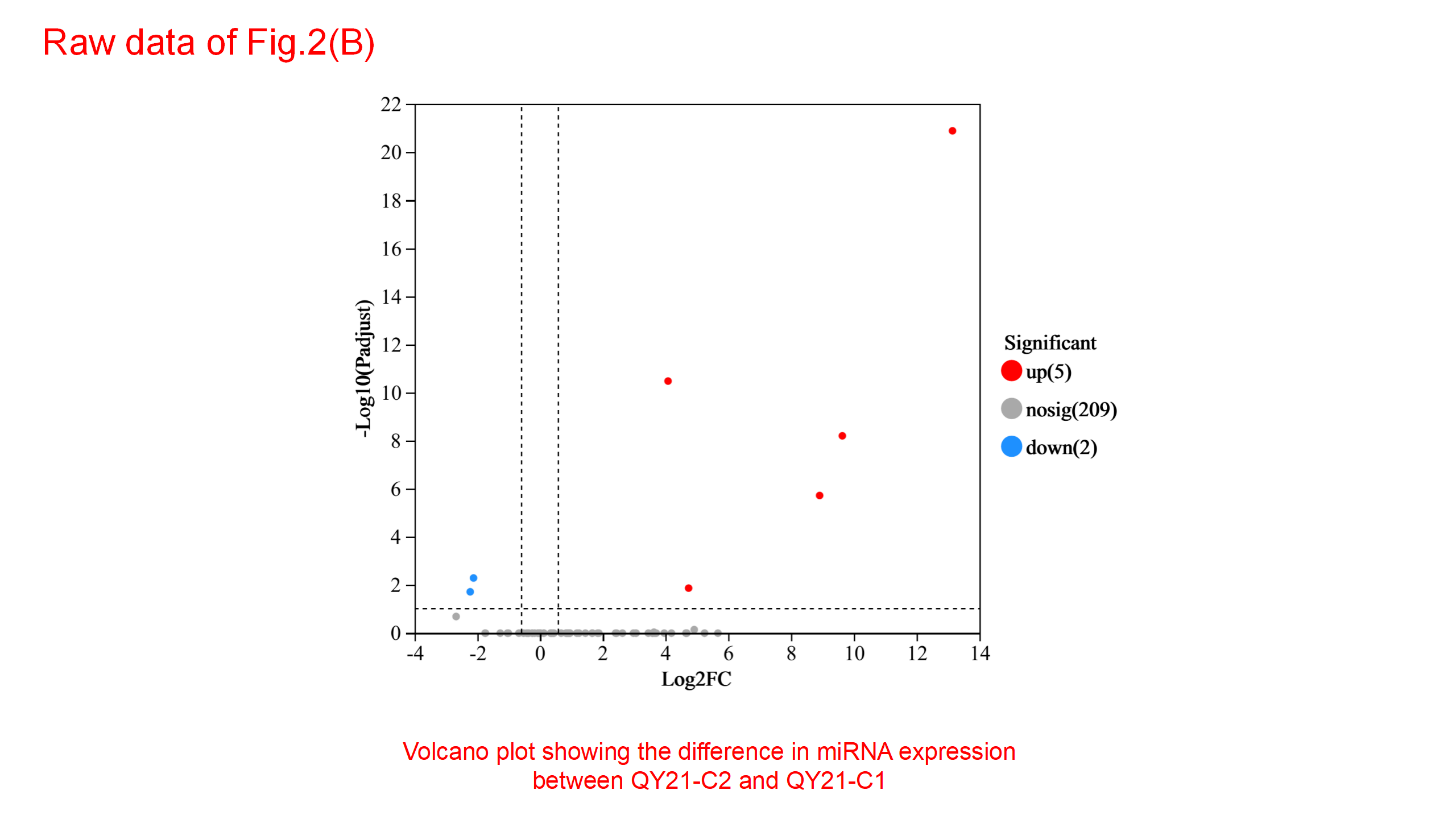

Supplement: Supplementary file 3 [file DataSheet3.zip › New Raw Images Fig1-6/New Fig.2 (B) Volcano plot showing the difference in miRNA expression between QY21-C2 and QY21-C1.tif]

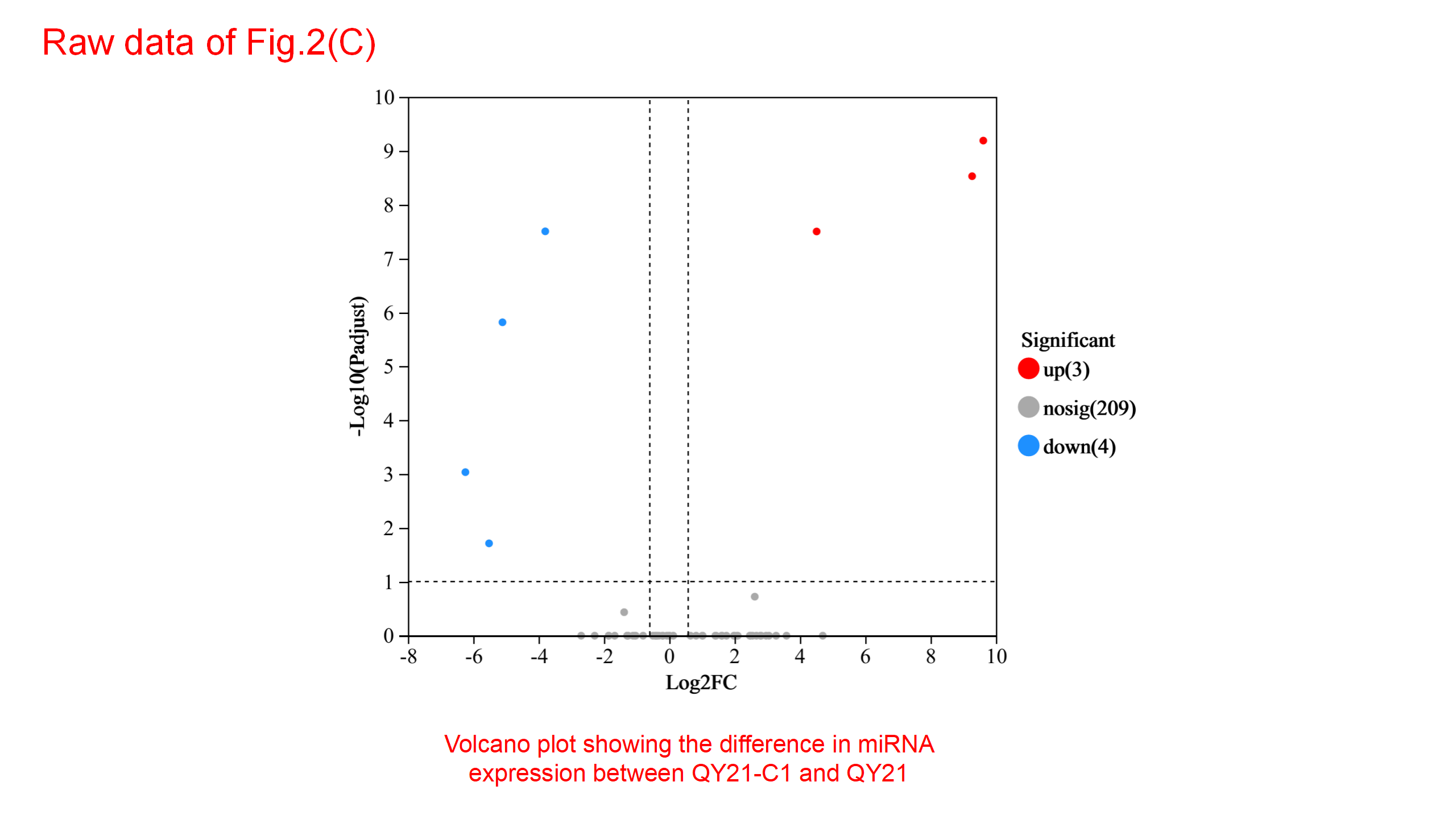

Supplement: Supplementary file 3 [file DataSheet3.zip › New Raw Images Fig1-6/New Fig.2 (C) Volcano plot showing the difference in miRNA expression between QY21-C1 and QY21.tif]

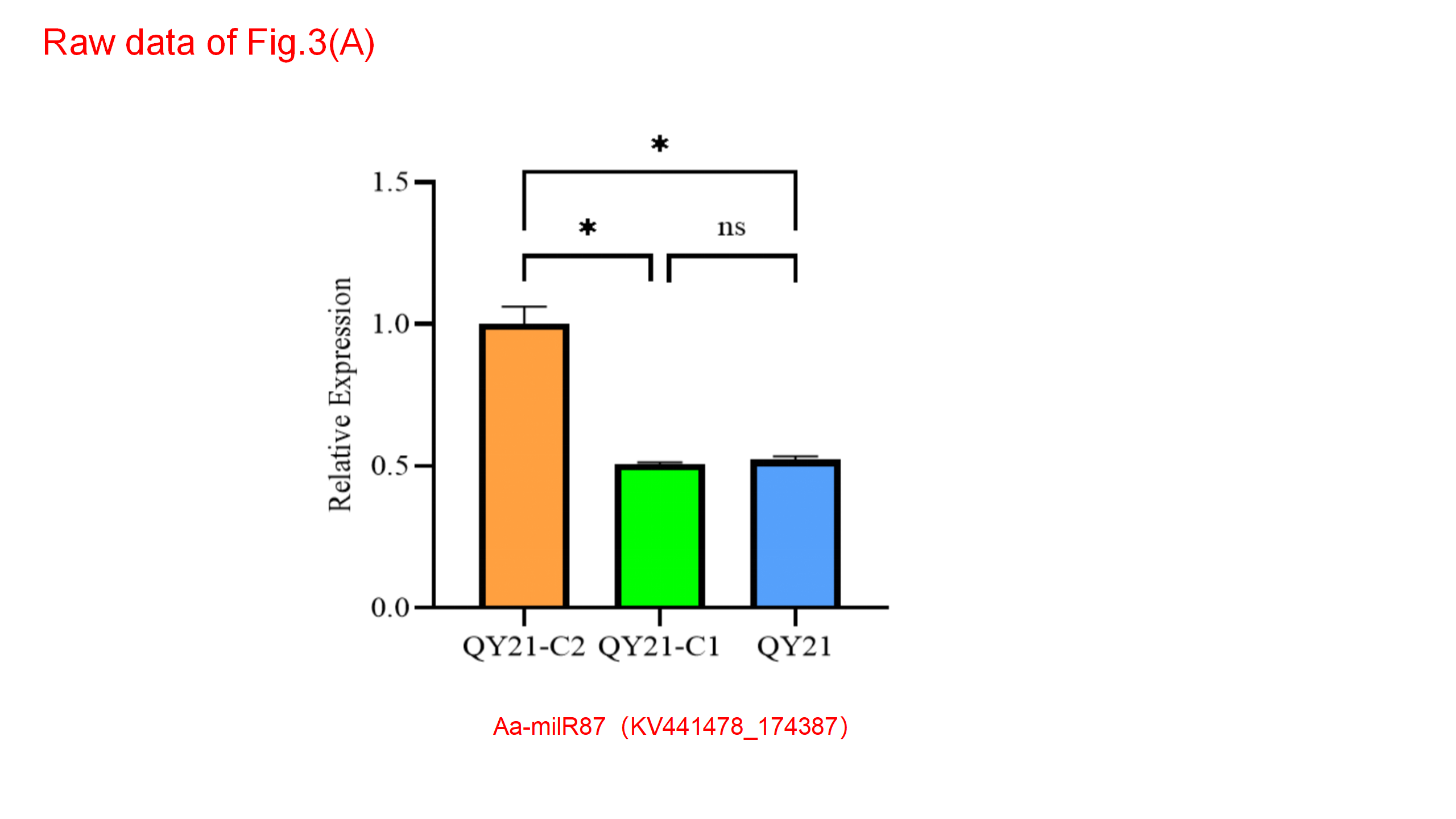

Supplement: Supplementary file 3 [file DataSheet3.zip › New Raw Images Fig1-6/New Fig.3 (A) Aa-milR87(KV441478_174387).tif]

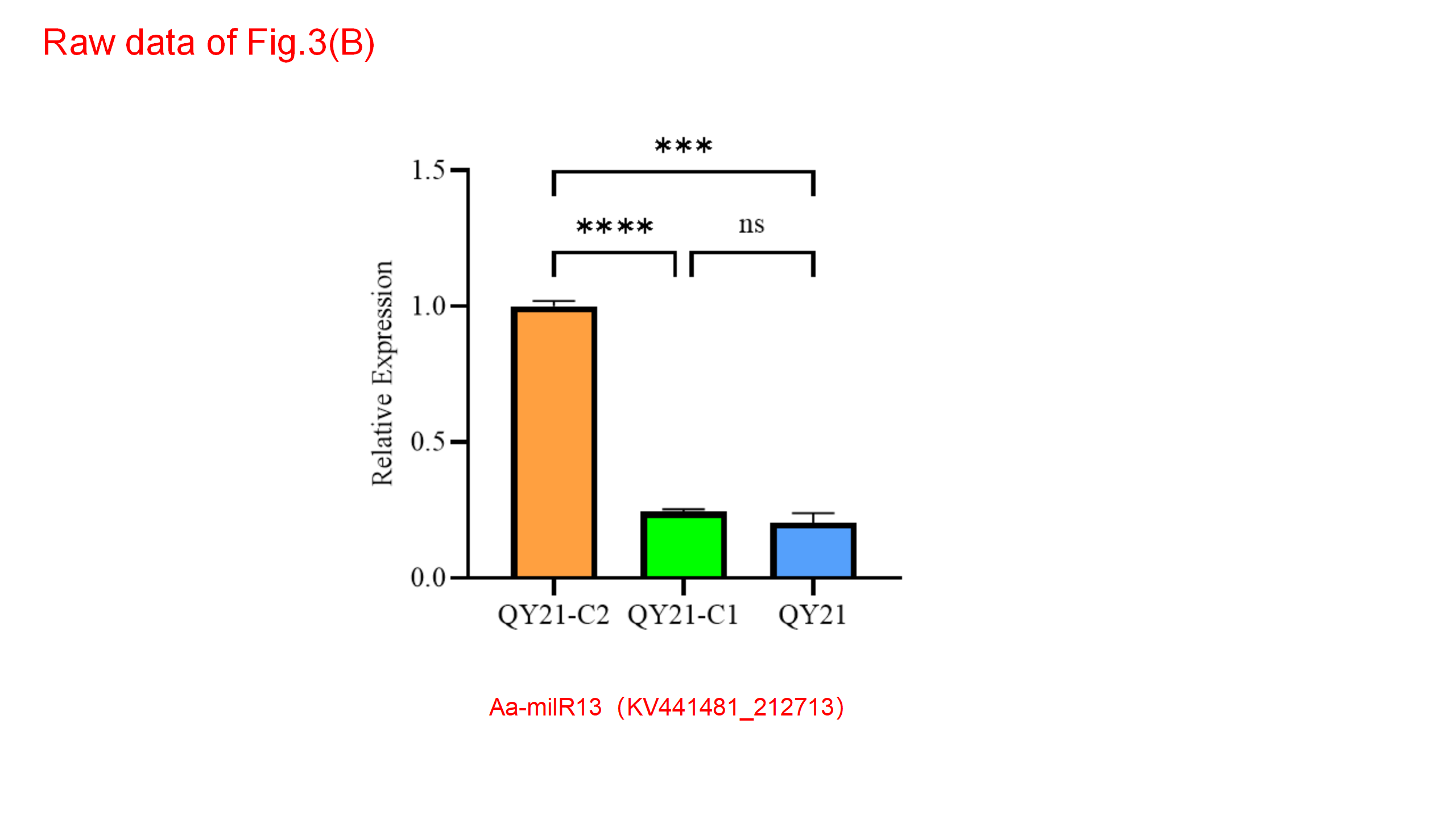

Supplement: Supplementary file 3 [file DataSheet3.zip › New Raw Images Fig1-6/New Fig.3 (B) Aa-milR13(KV441481_212713).tif]

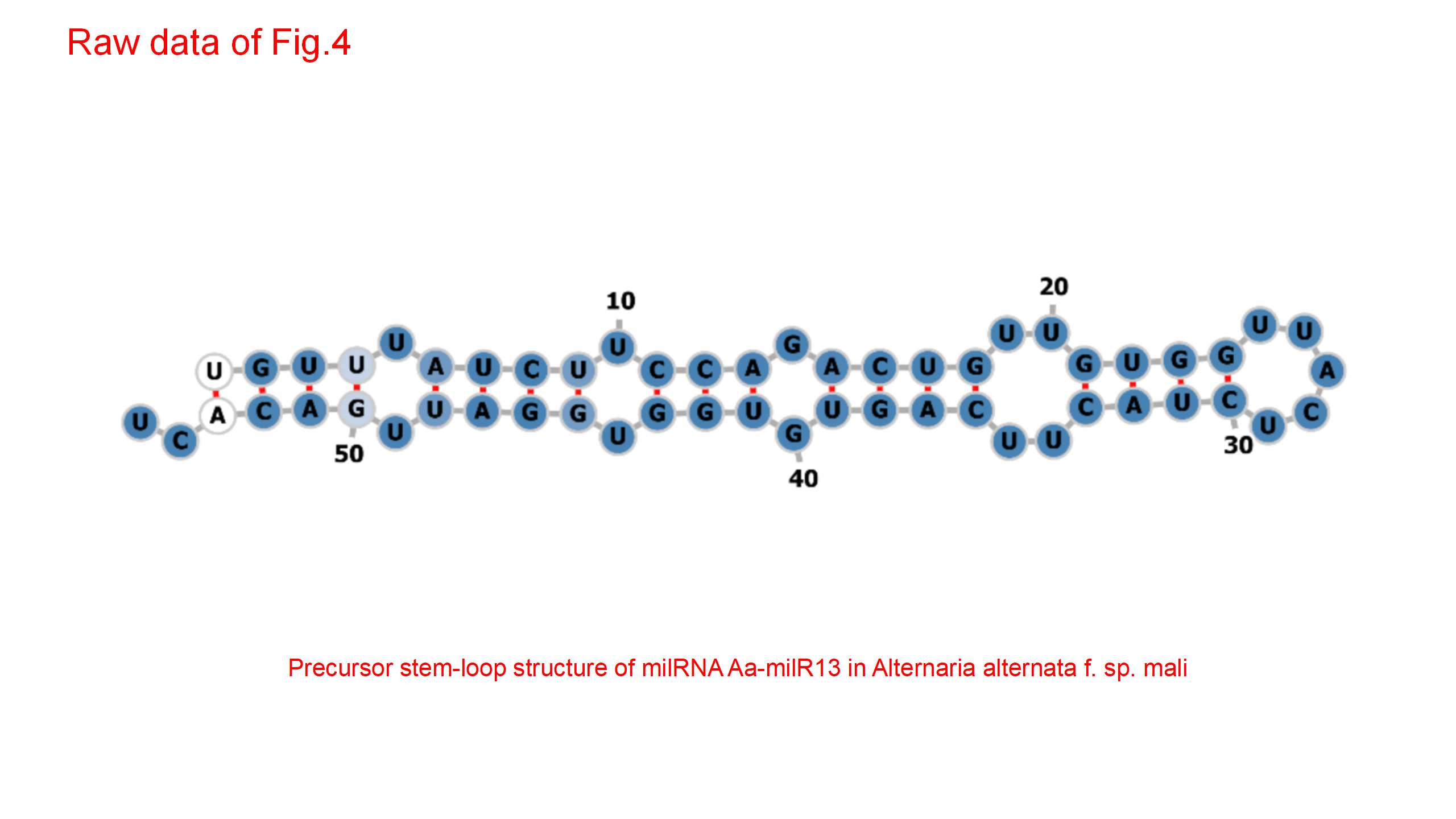

Supplement: Supplementary file 3 [file DataSheet3.zip › New Raw Images Fig1-6/New Fig.4 Precursor stem-loop structure of milRNA Aa-milR13 in Alternaria alternata f. sp. mali.tif]

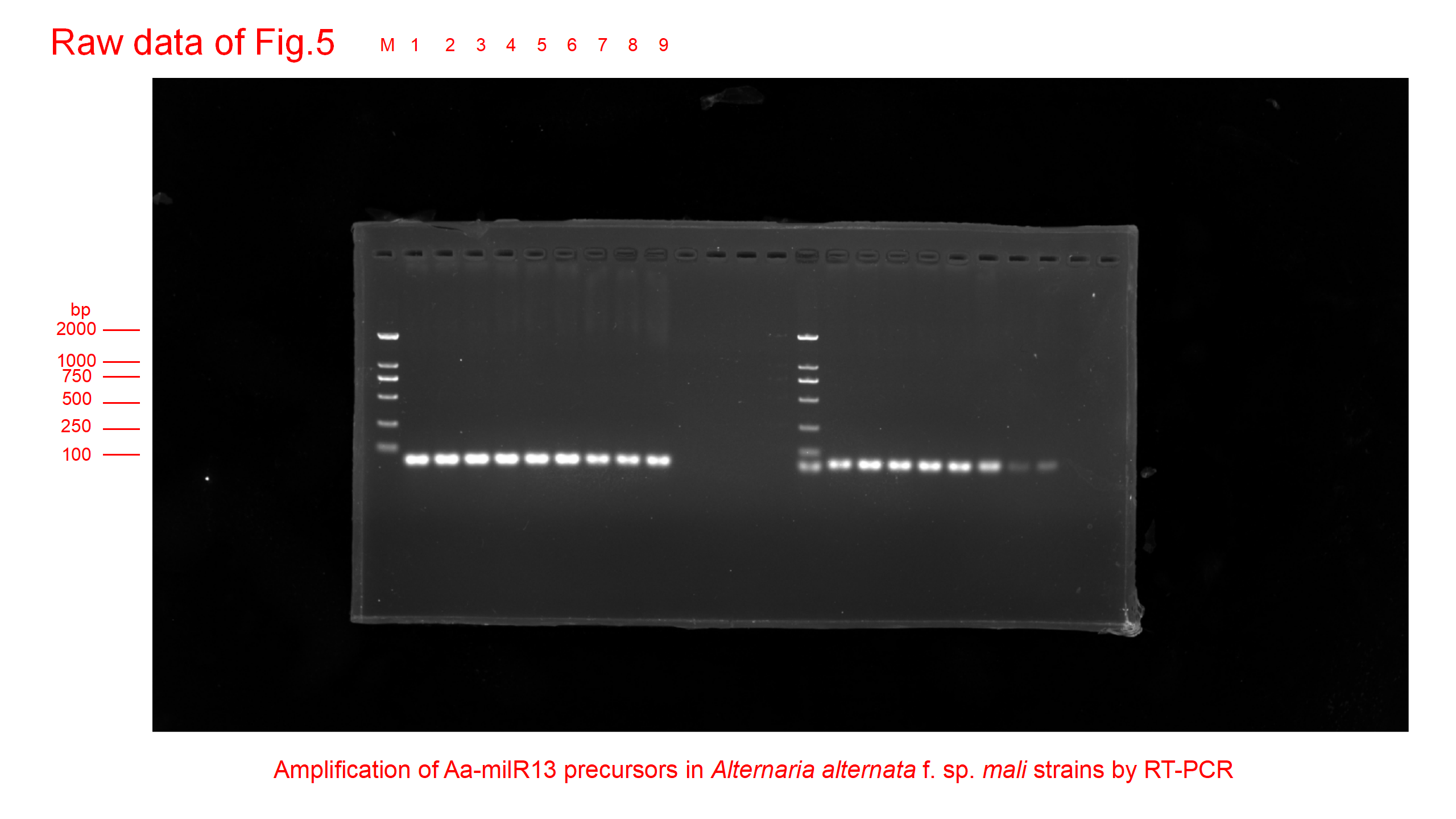

Supplement: Supplementary file 3 [file DataSheet3.zip › New Raw Images Fig1-6/New Fig.5 Amplification of Aa-milR13 precursors in Alternaria alternata f. sp. mali strains by RT-PCR.tif]

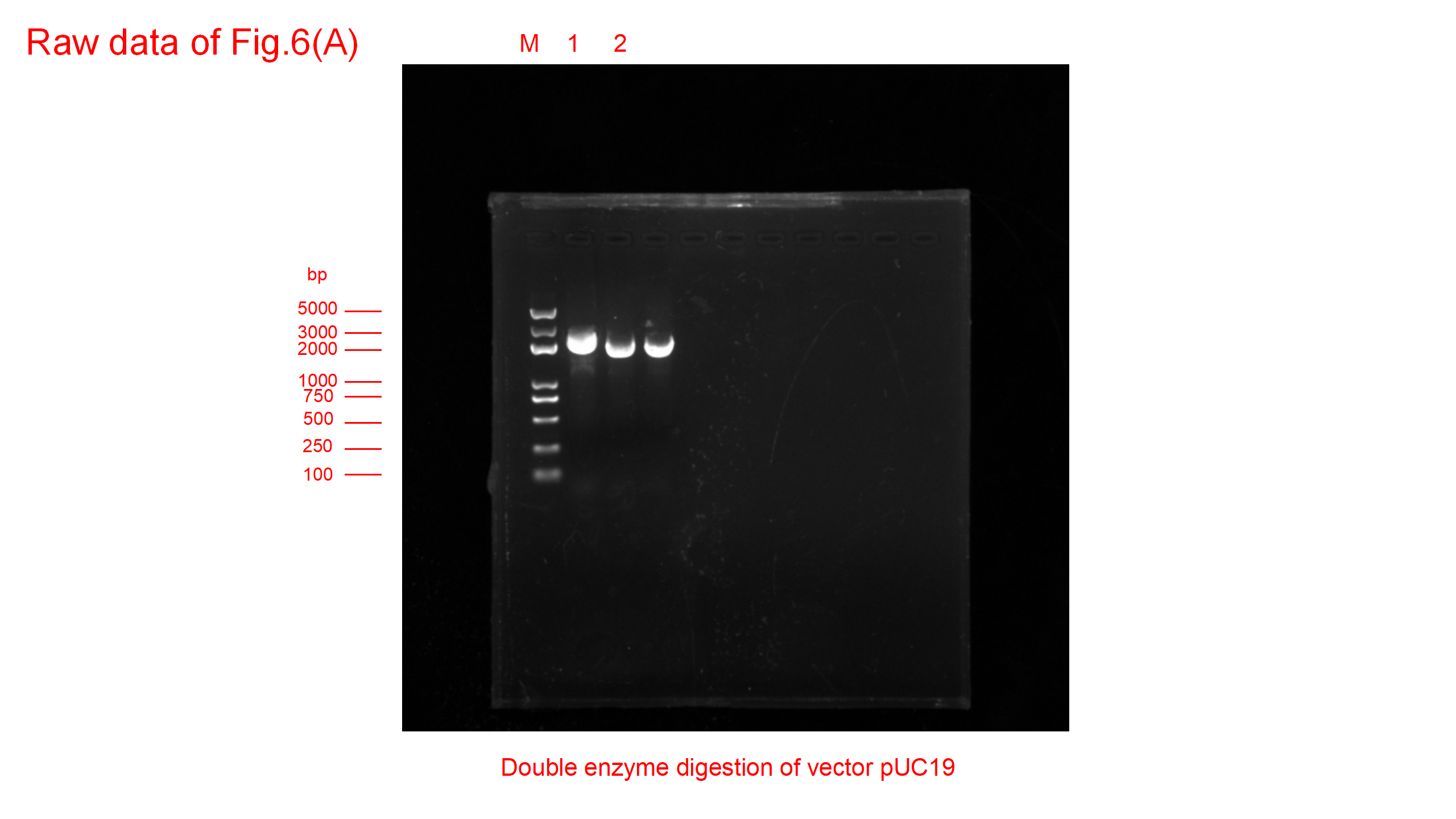

Supplement: Supplementary file 3 [file DataSheet3.zip › New Raw Images Fig1-6/New Fig.6 (A) Double enzyme digestion of vector pUC19.tif]

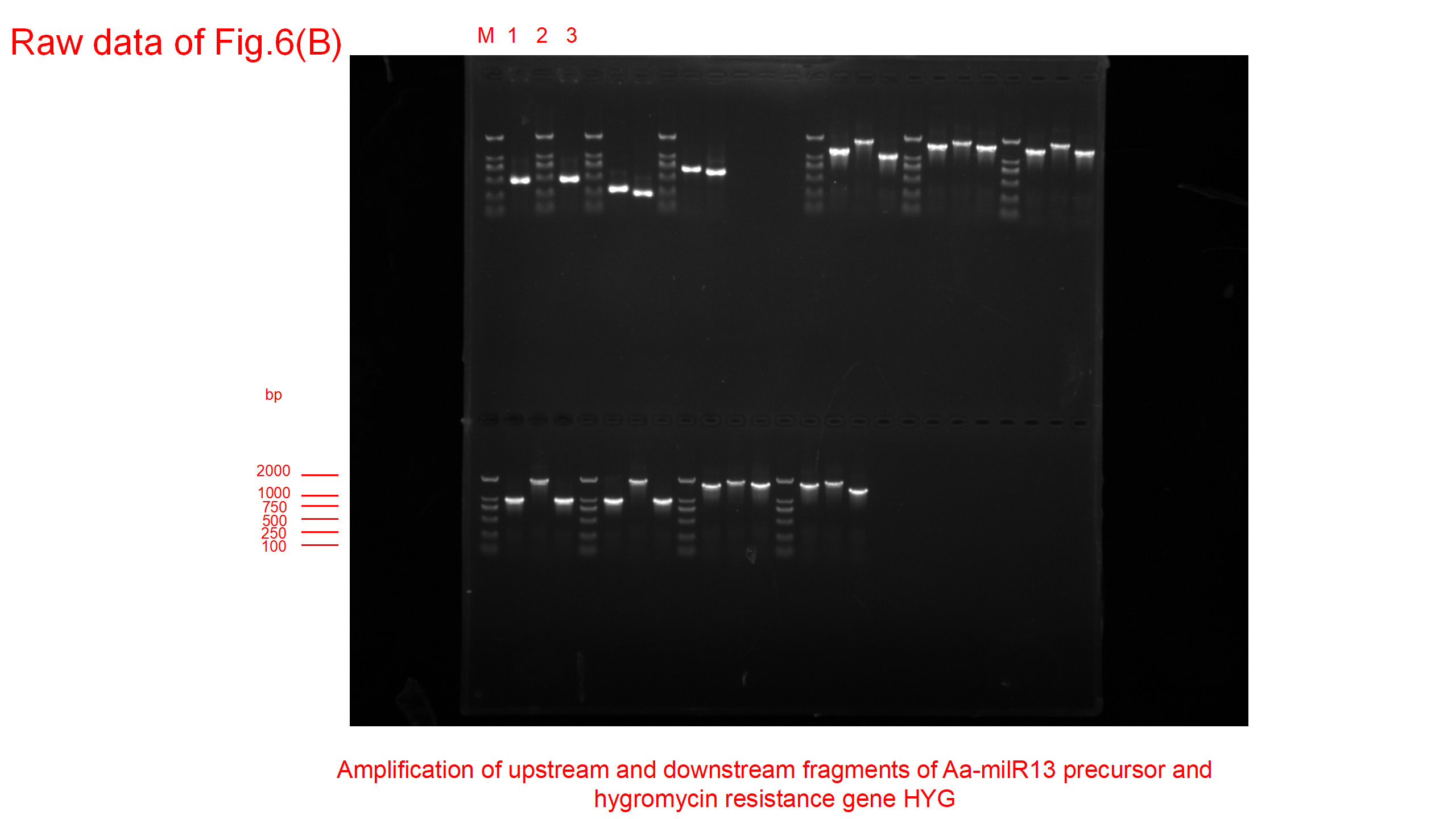

Supplement: Supplementary file 3 [file DataSheet3.zip › New Raw Images Fig1-6/New Fig.6 (B) Amplification of upstream and downstream fragments of Aa-milR13 precursor and hygromycin resistance gene HYG.tif]

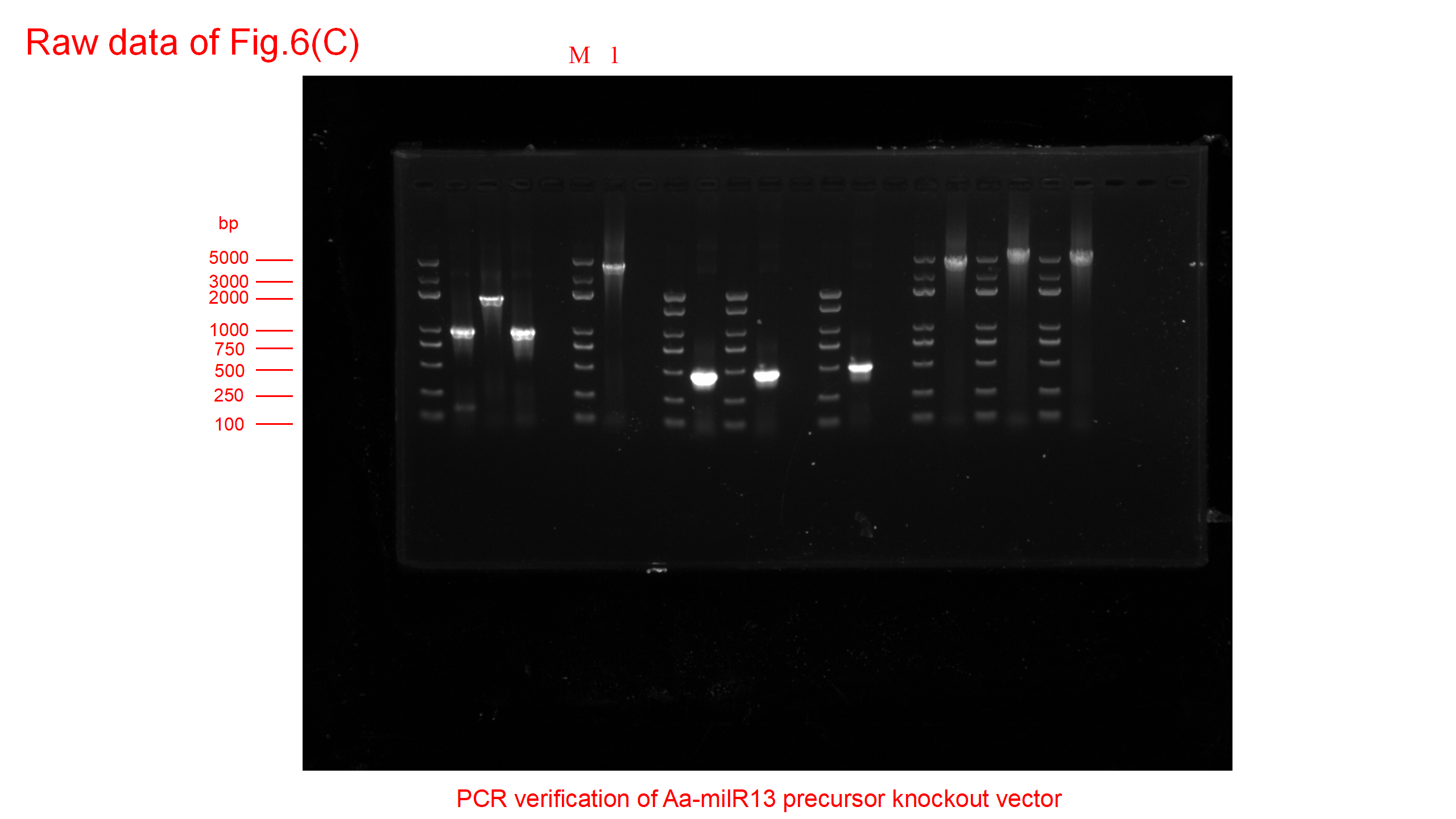

Supplement: Supplementary file 3 [file DataSheet3.zip › New Raw Images Fig1-6/New Fig.6 (C) PCR verification of Aa-milR13 precursor knockout vector.tif]

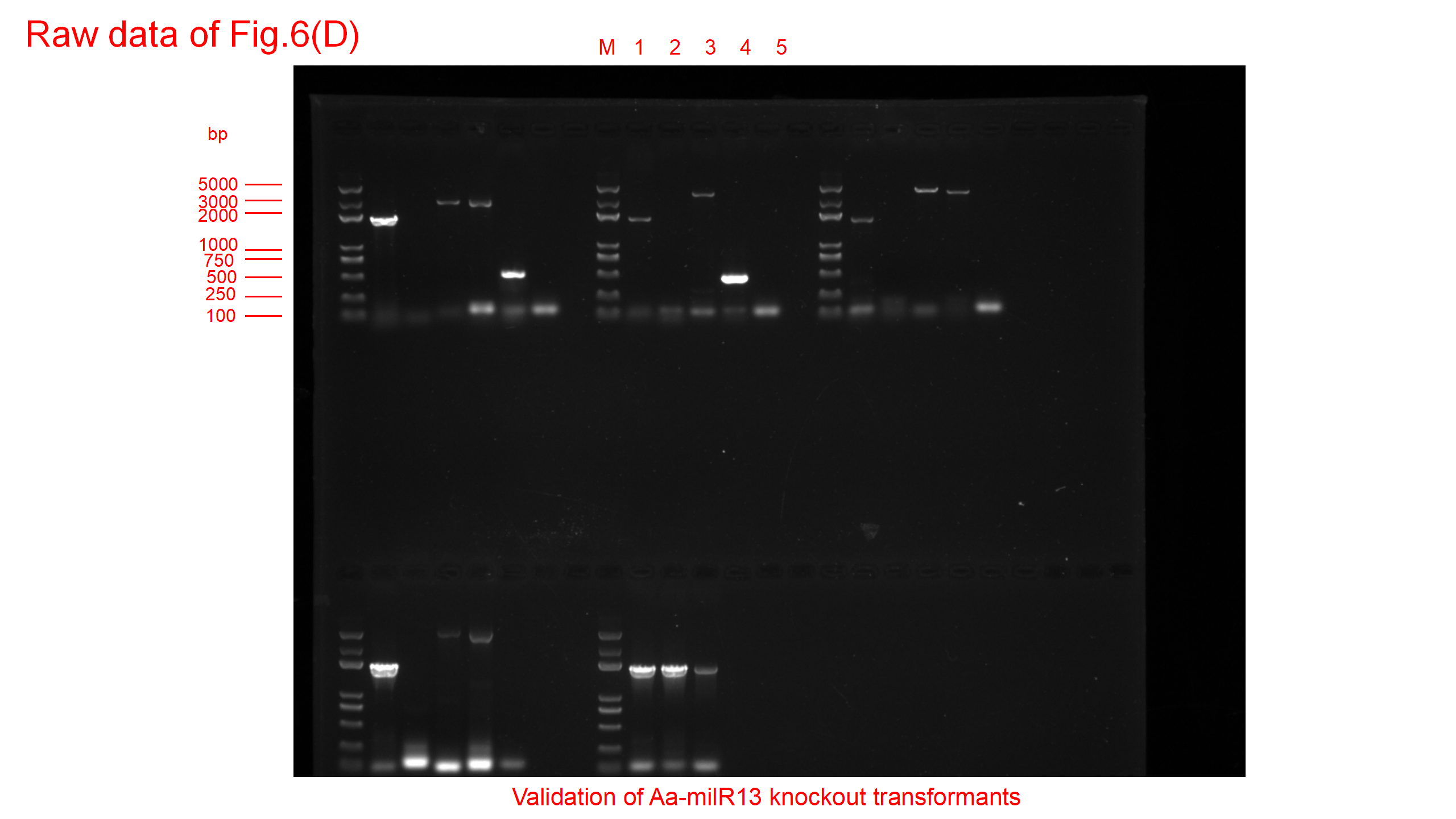

Supplement: Supplementary file 3 [file DataSheet3.zip › New Raw Images Fig1-6/New Fig.6 (D) Validation of Aa-milR13 knockout transformants.tif]

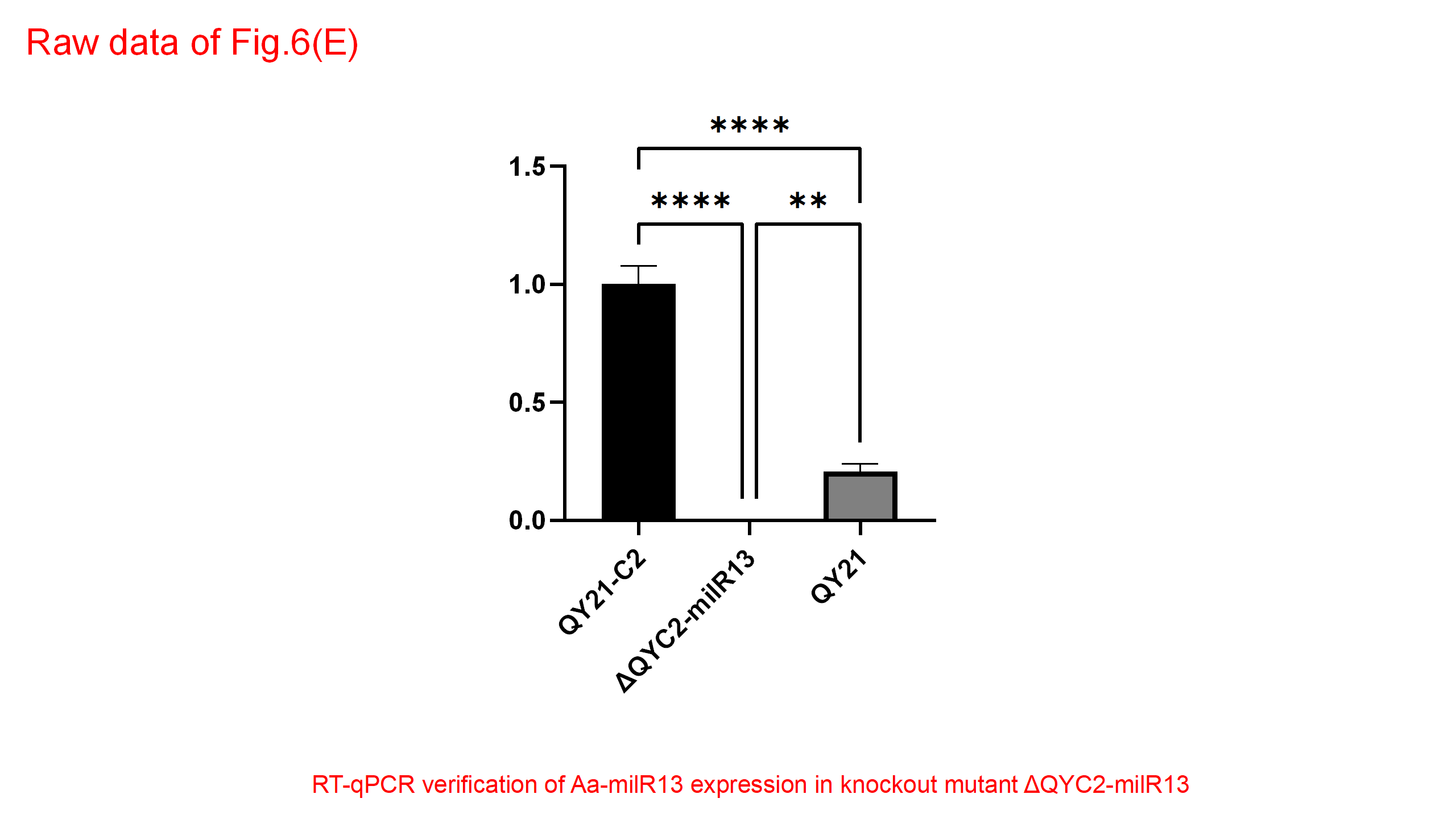

Supplement: Supplementary file 3 [file DataSheet3.zip › New Raw Images Fig1-6/New Fig.6 (E) RT-qPCR verification of Aa-milR13 expression in knockout mutant ΔQYC2-milR13.tif]

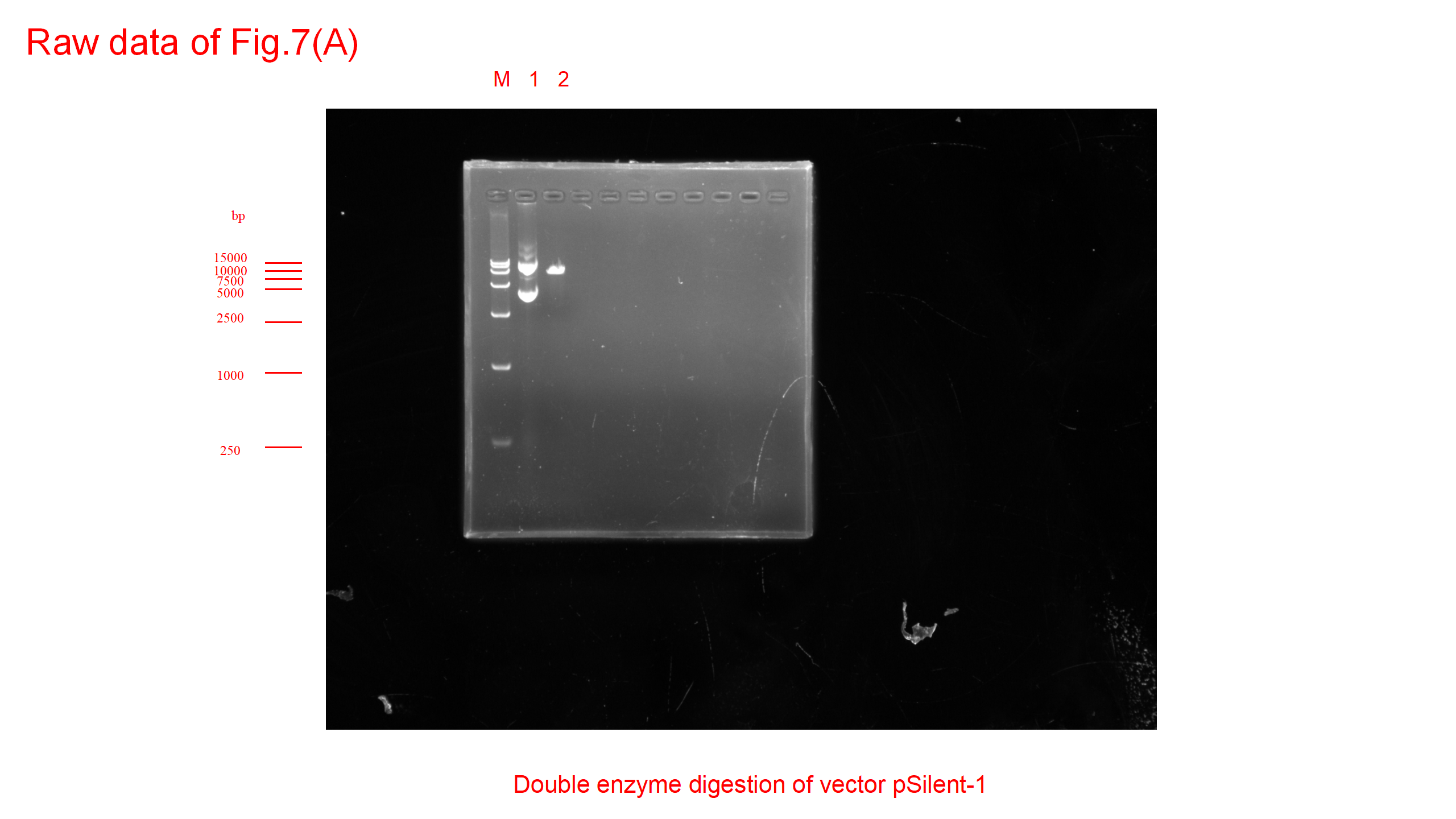

Supplement: Supplementary file 4 [file DataSheet4.zip › New Raw Images Fig7/New Fig.7 (A) Double enzyme digestion of vector pSilent-1.tif]

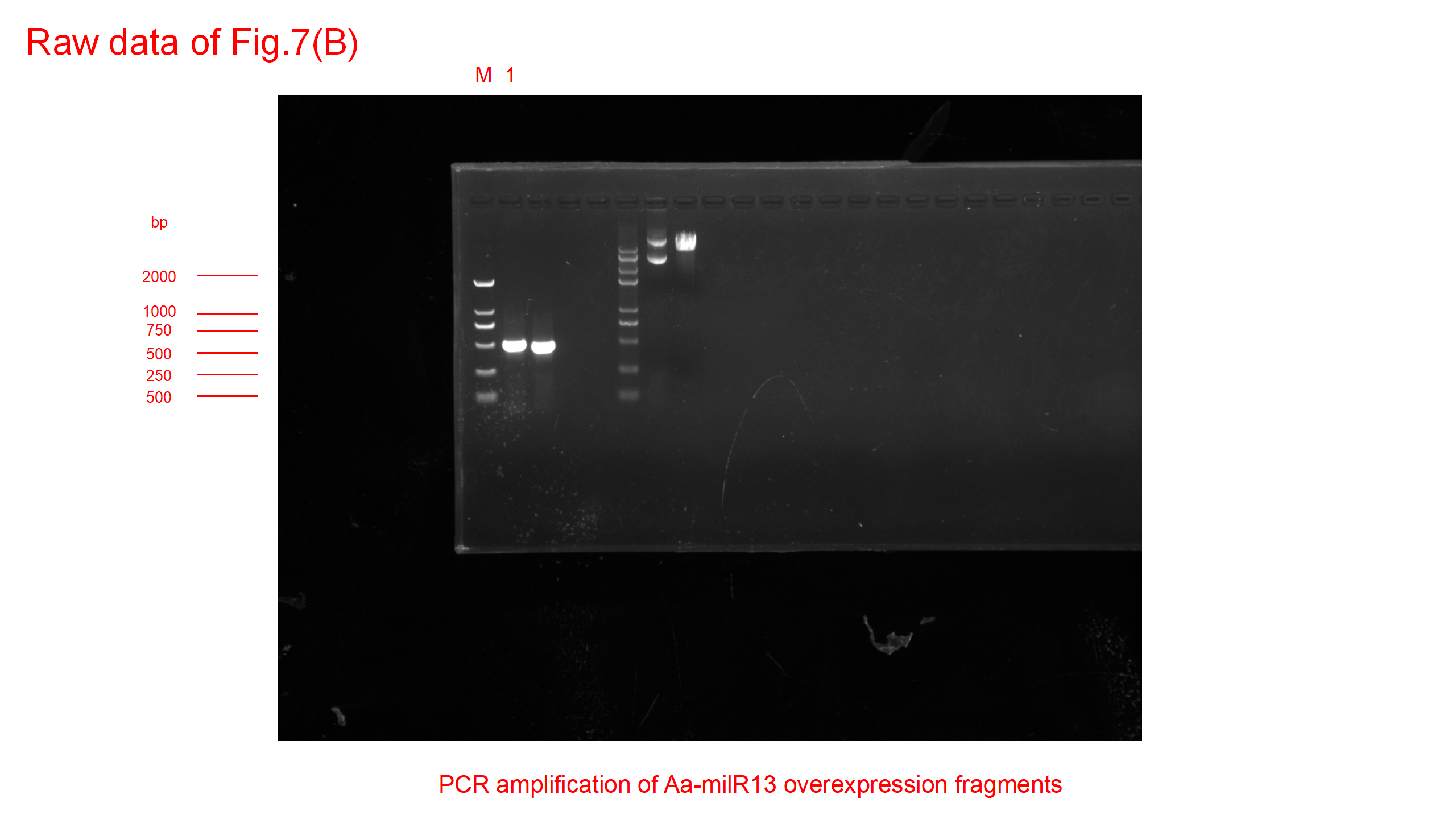

Supplement: Supplementary file 4 [file DataSheet4.zip › New Raw Images Fig7/New Fig.7 (B) PCR amplification of Aa-milR13 overexpression fragments.tif]

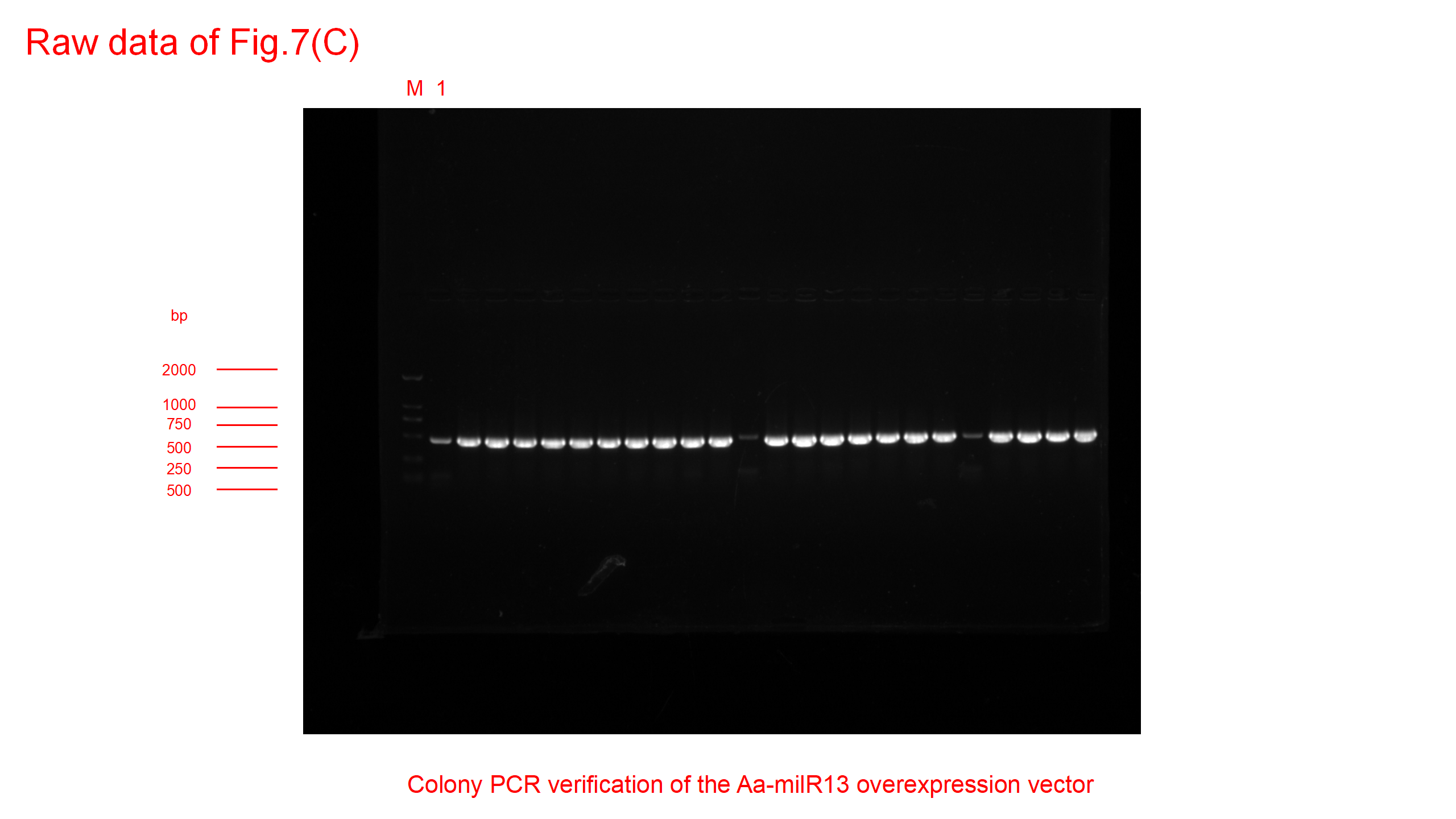

Supplement: Supplementary file 4 [file DataSheet4.zip › New Raw Images Fig7/New Fig.7 (C) Colony PCR verification of the Aa-milR13 overexpression vector.tif]

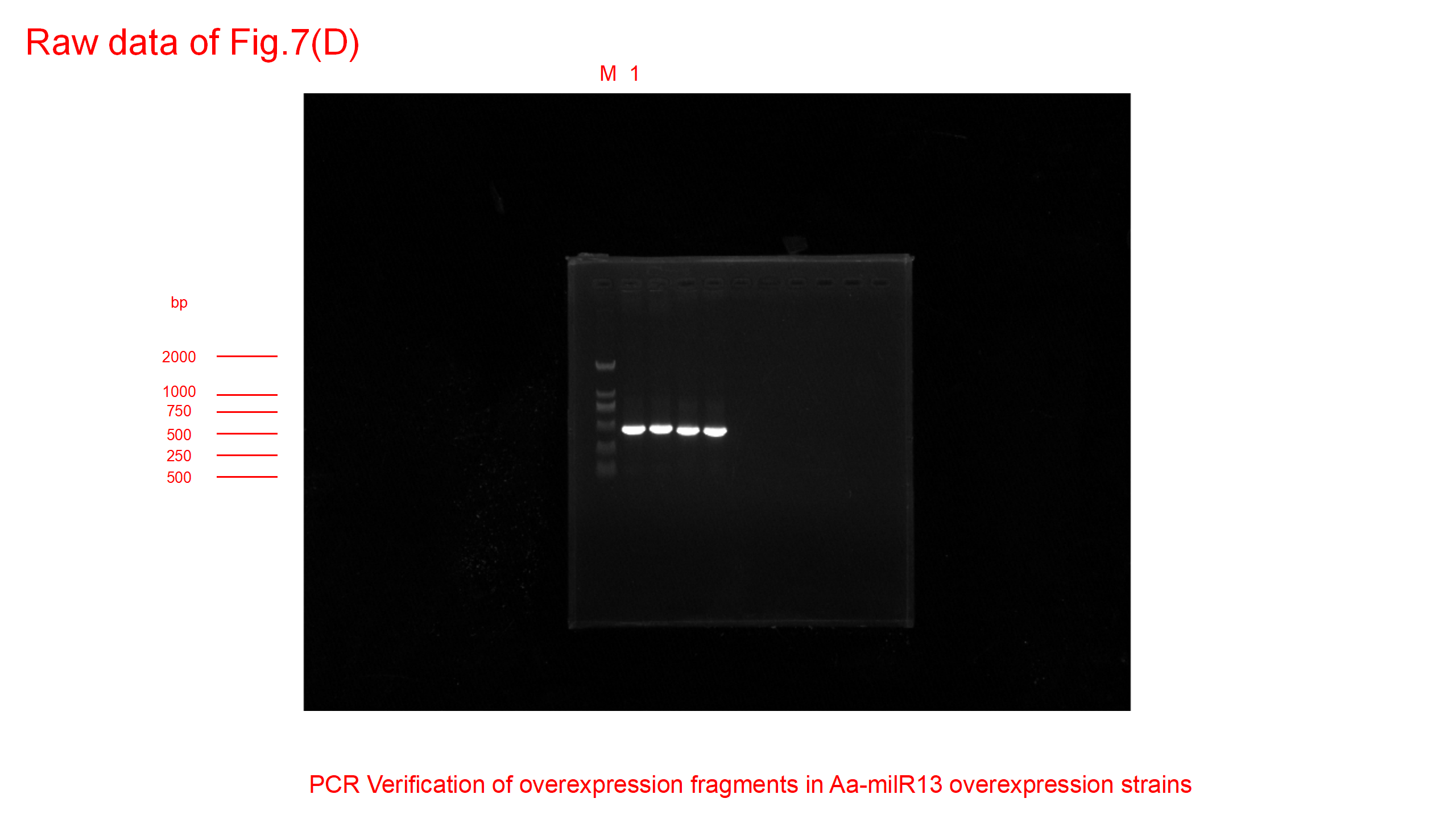

Supplement: Supplementary file 4 [file DataSheet4.zip › New Raw Images Fig7/New Fig.7 (D) PCR Verification of overexpression fragments in Aa-milR13 overexpression strains.tif]

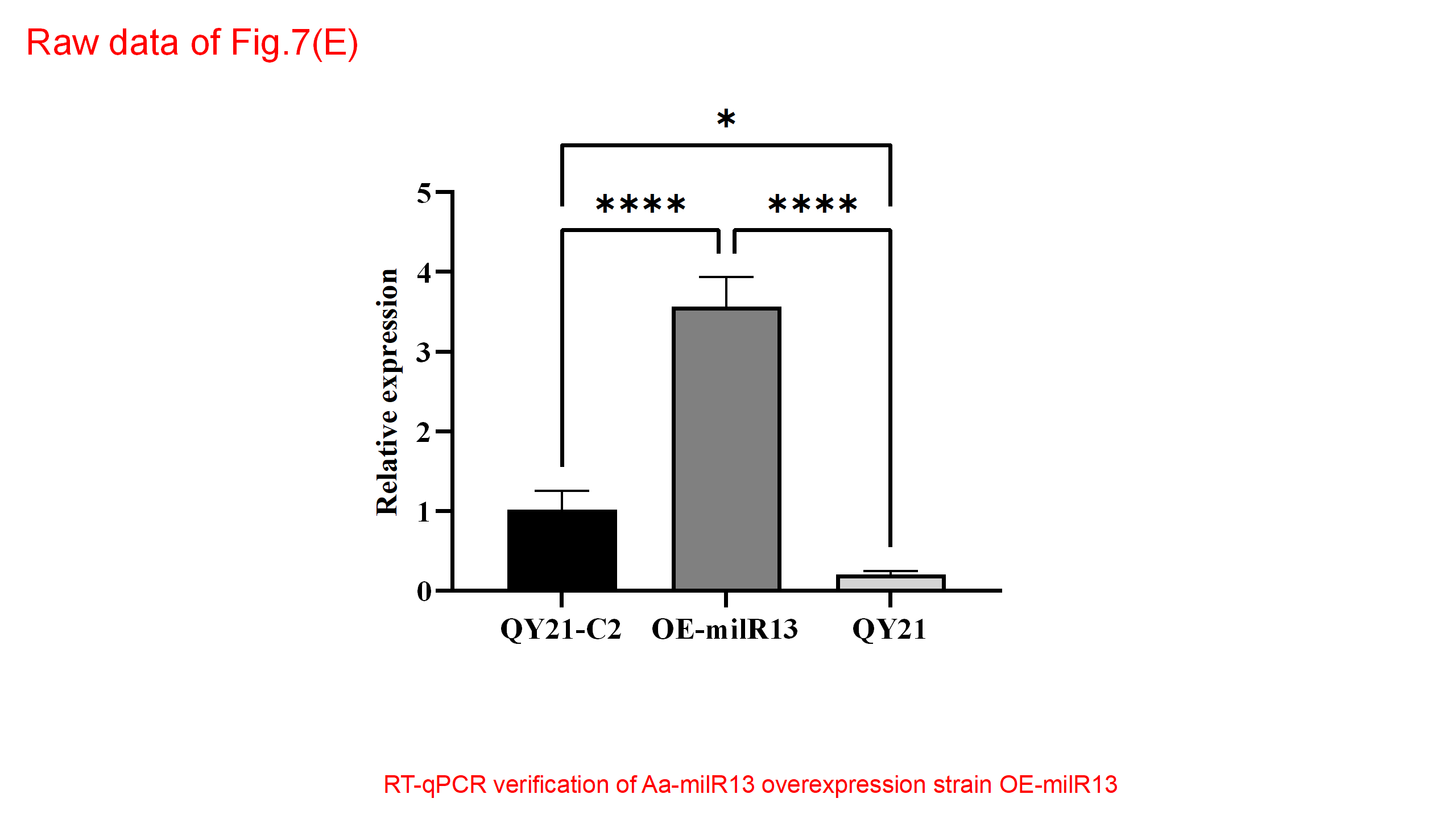

Supplement: Supplementary file 4 [file DataSheet4.zip › New Raw Images Fig7/New Fig.7 (E) RT-qPCR verification of Aa-milR13 overexpression strain OE-milR13.tif]

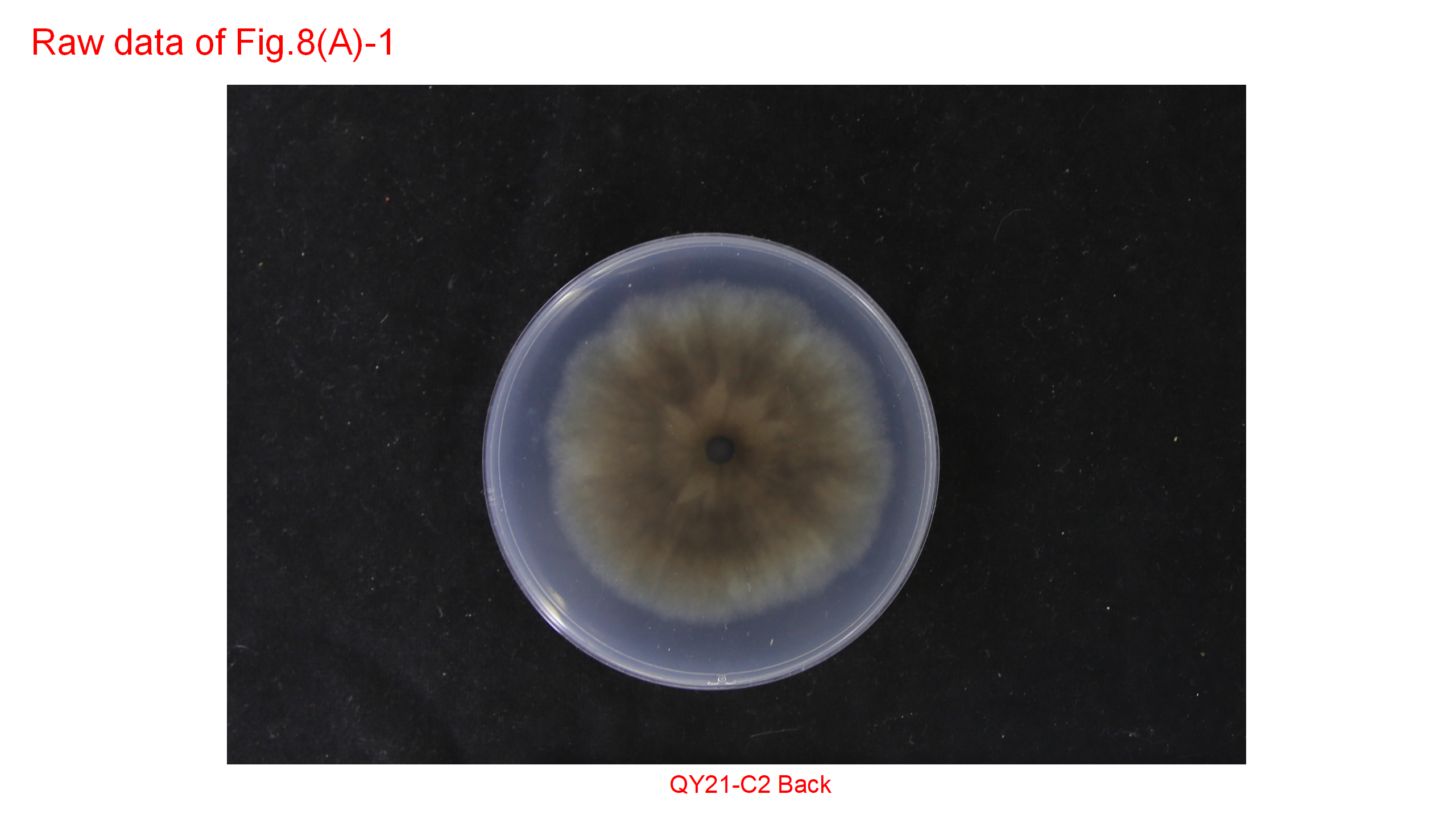

Supplement: Supplementary file 5 [file DataSheet5.zip › New Raw Images Fig8/New Fig.8 (A)-1 QY21-C2 Back.tif]

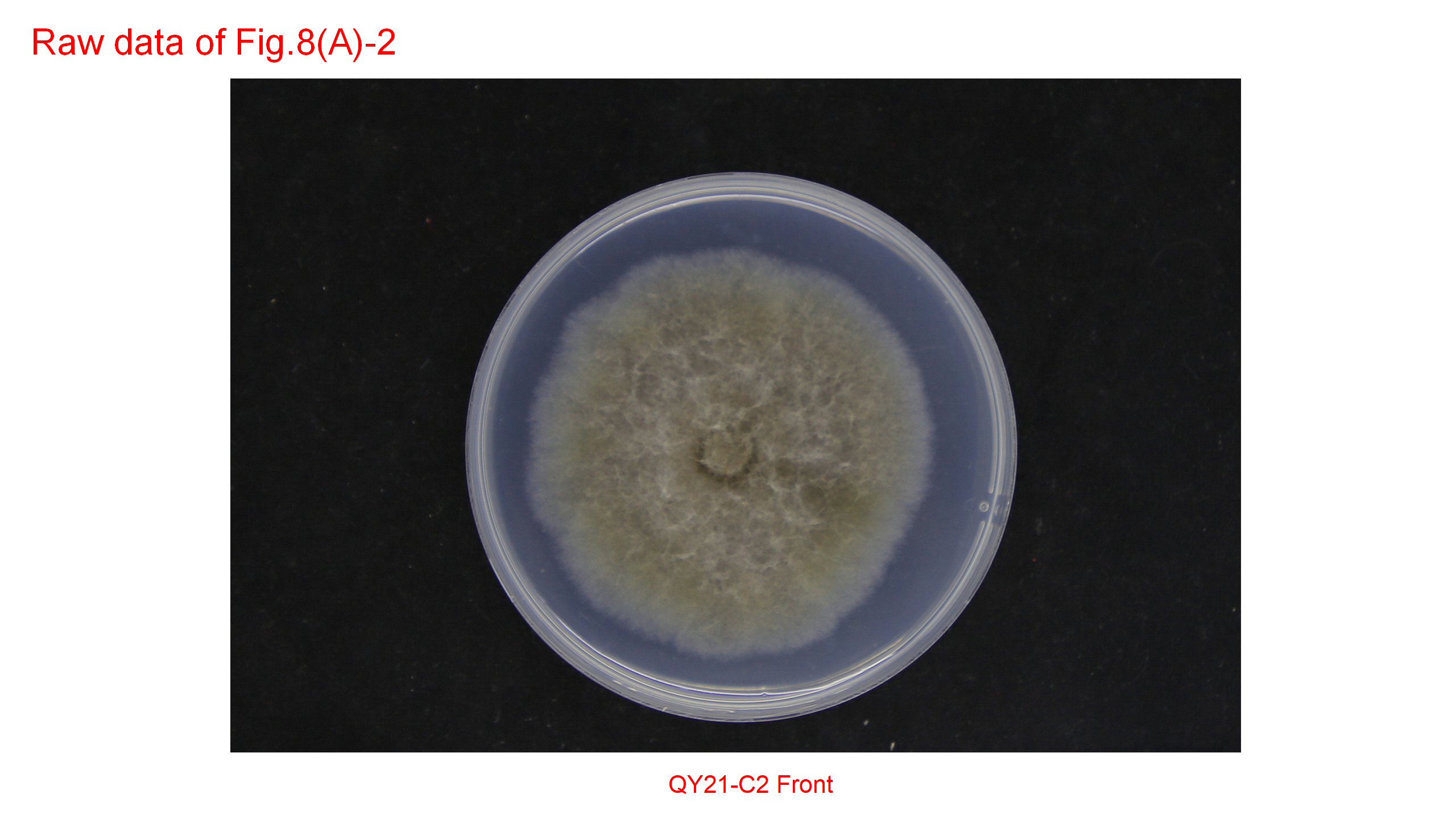

Supplement: Supplementary file 5 [file DataSheet5.zip › New Raw Images Fig8/New Fig.8 (A)-2 QY21-C2 Front.tif]

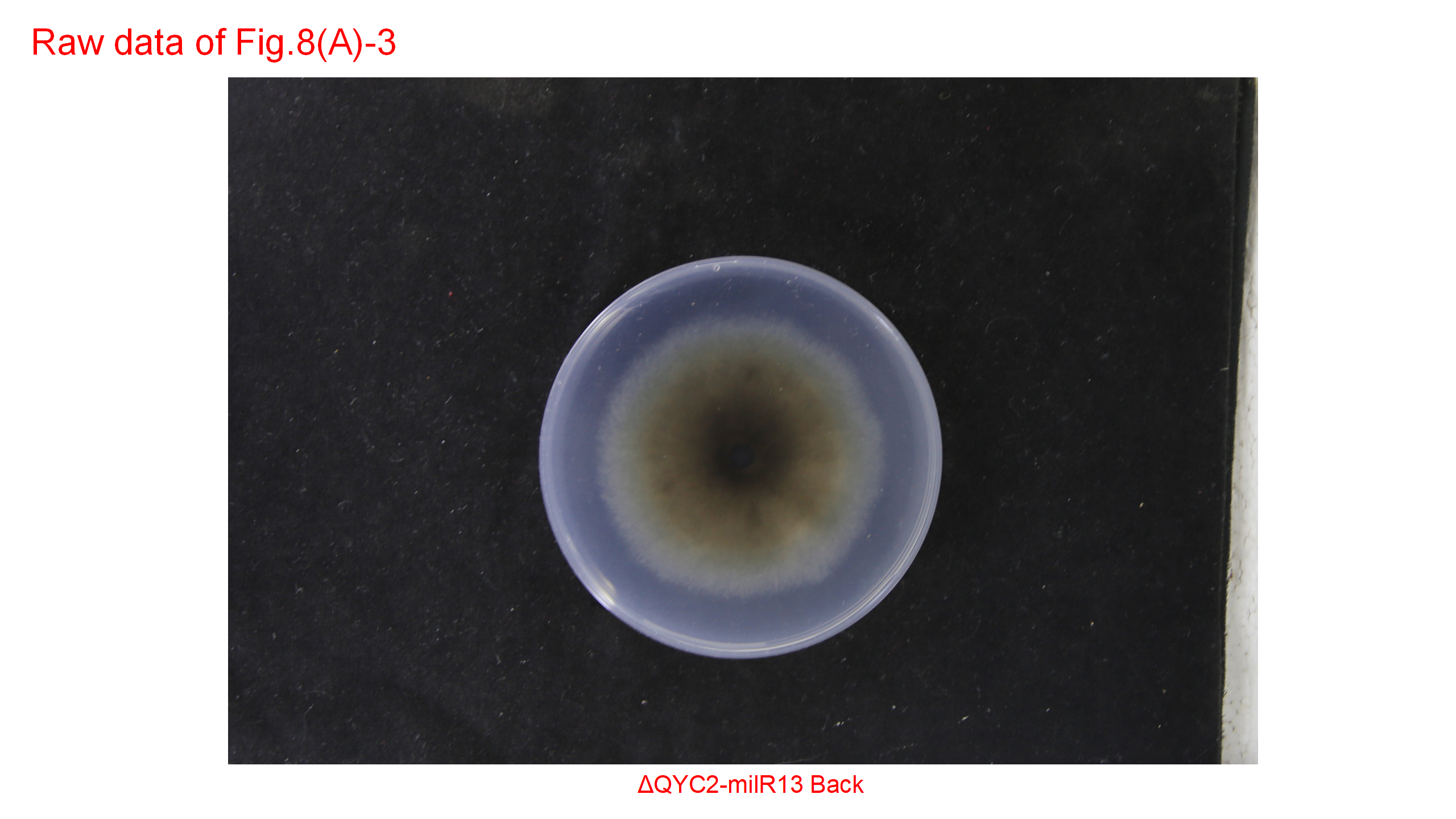

Supplement: Supplementary file 5 [file DataSheet5.zip › New Raw Images Fig8/New Fig.8 (A)-3 ΔQYC2-milR13 Back.tif]

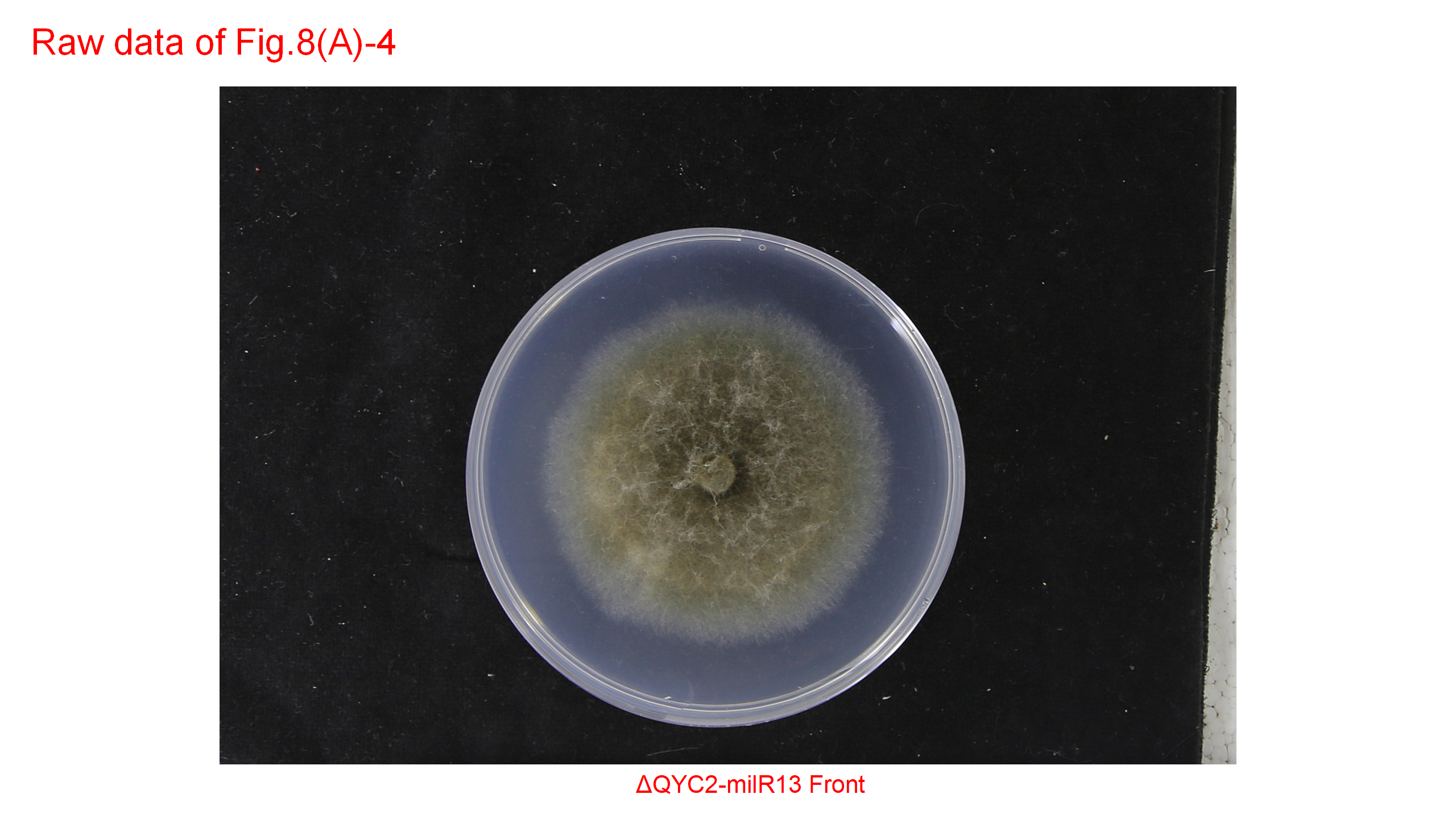

Supplement: Supplementary file 5 [file DataSheet5.zip › New Raw Images Fig8/New Fig.8 (A)-4 ΔQYC2-milR13 Front.tif]

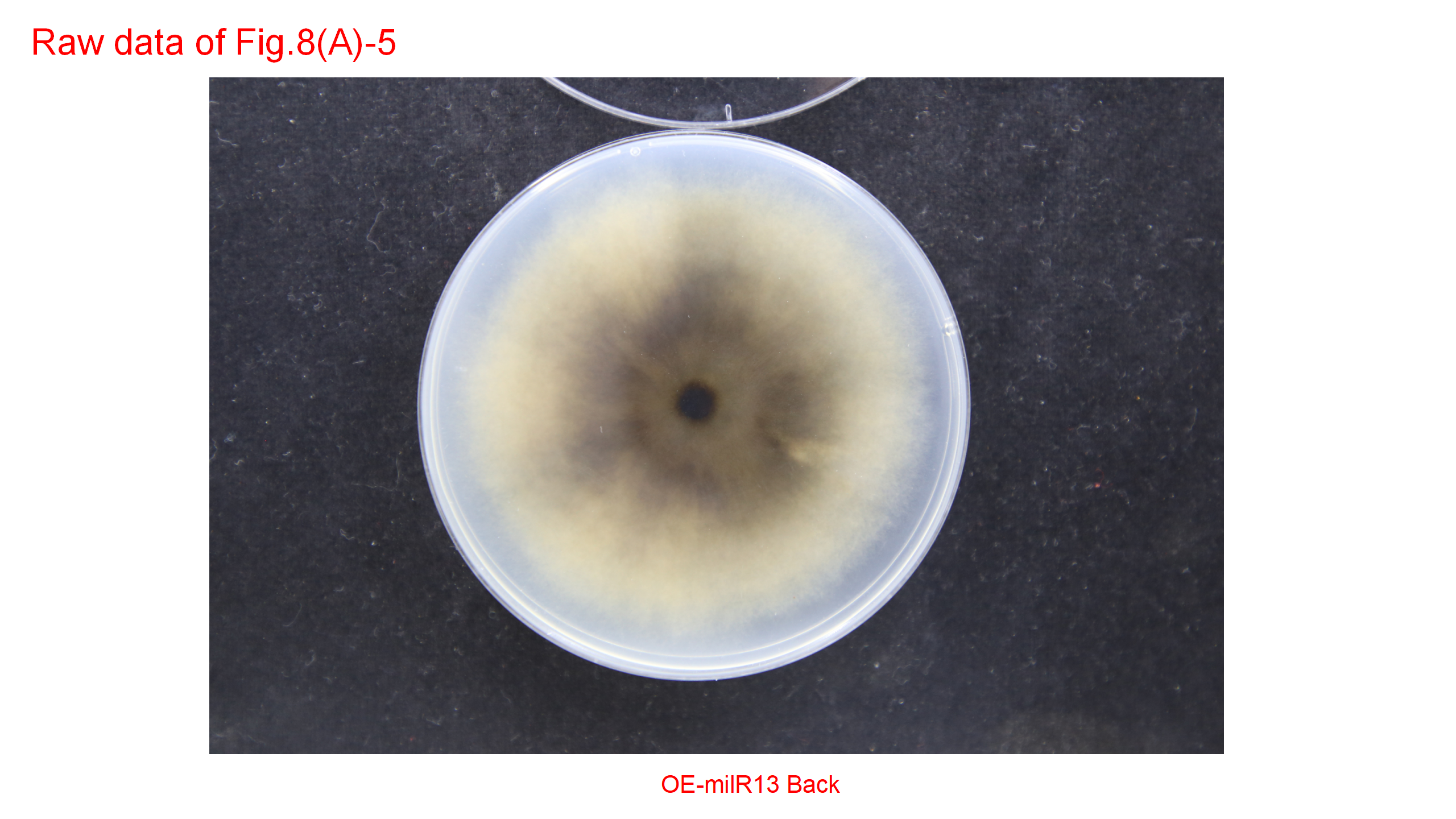

Supplement: Supplementary file 5 [file DataSheet5.zip › New Raw Images Fig8/New Fig.8 (A)-5 OE-milR13 Back.tif]

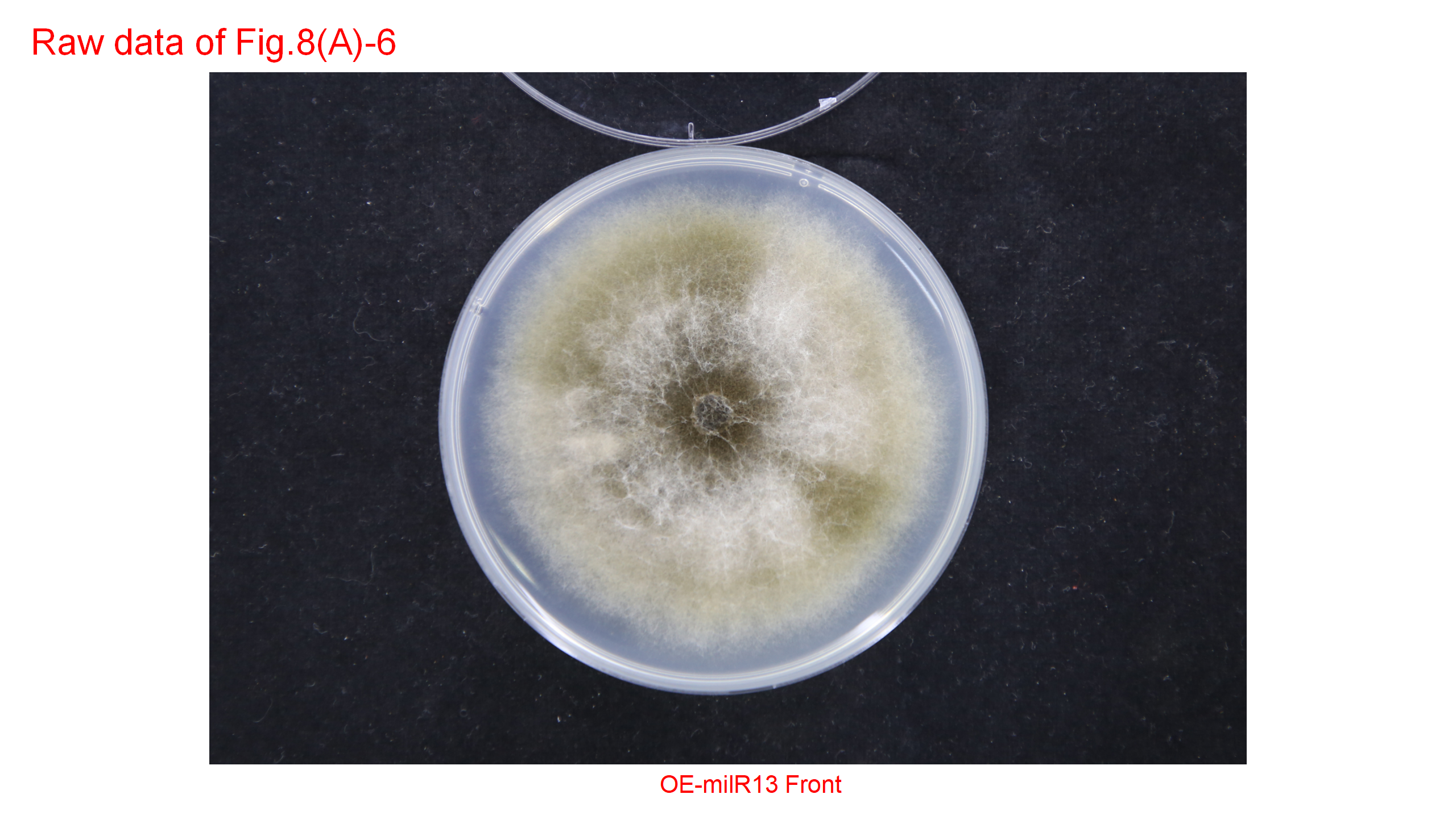

Supplement: Supplementary file 5 [file DataSheet5.zip › New Raw Images Fig8/New Fig.8 (A)-6 OE-milR13 Front.tif]

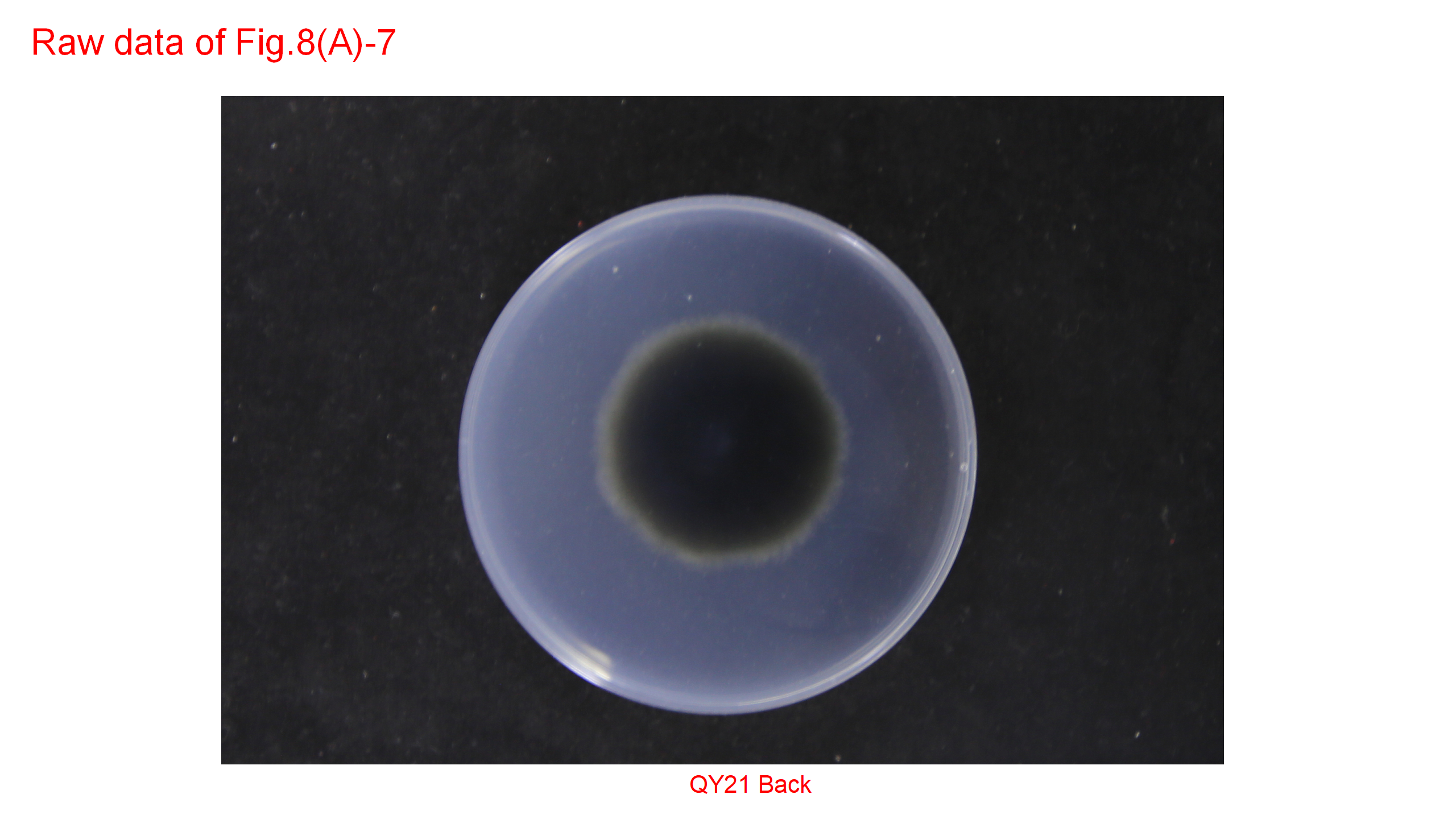

Supplement: Supplementary file 5 [file DataSheet5.zip › New Raw Images Fig8/New Fig.8 (A)-7 QY21 Back.tif]

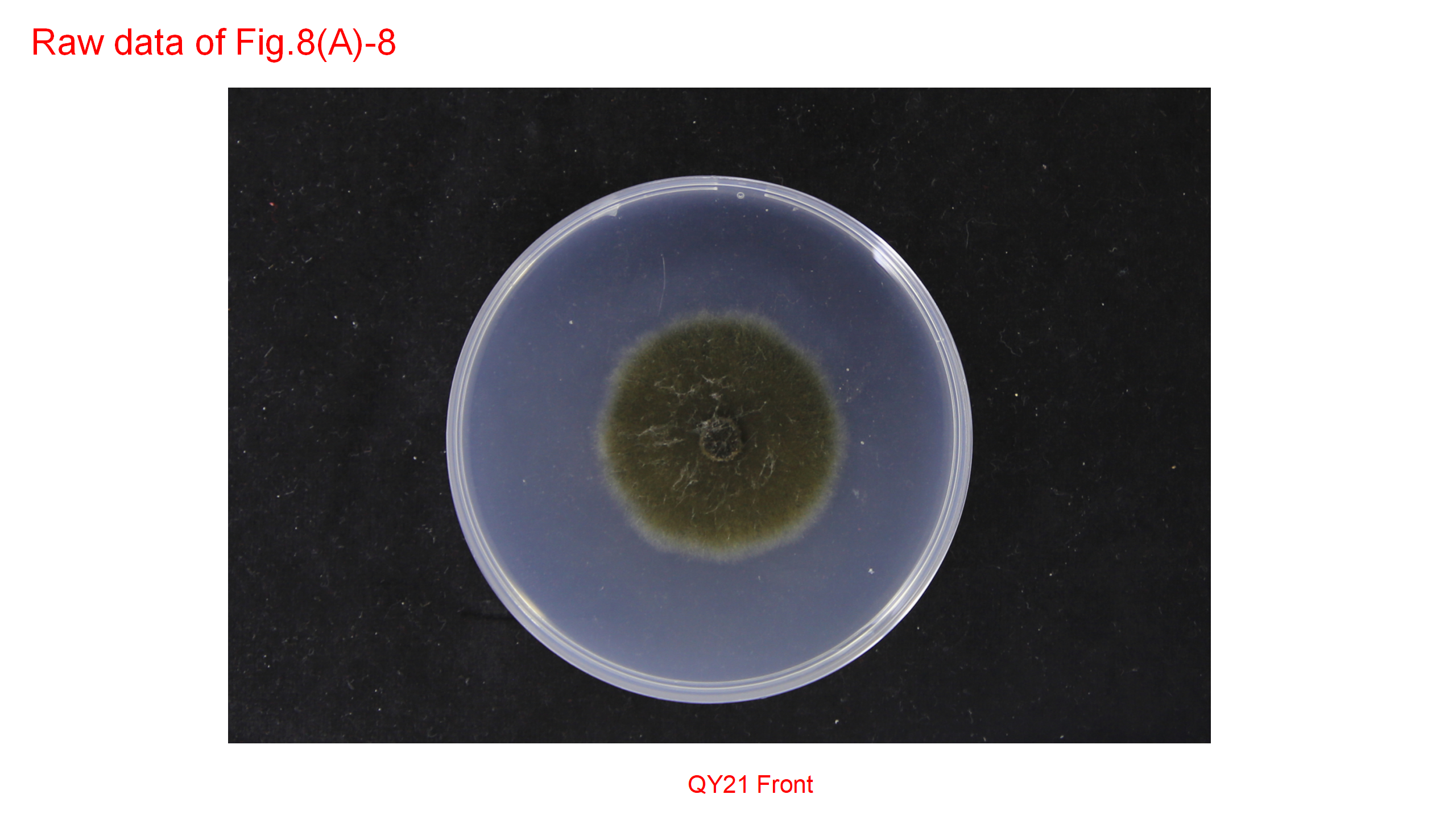

Supplement: Supplementary file 5 [file DataSheet5.zip › New Raw Images Fig8/New Fig.8 (A)-8 QY21 Front.tif]

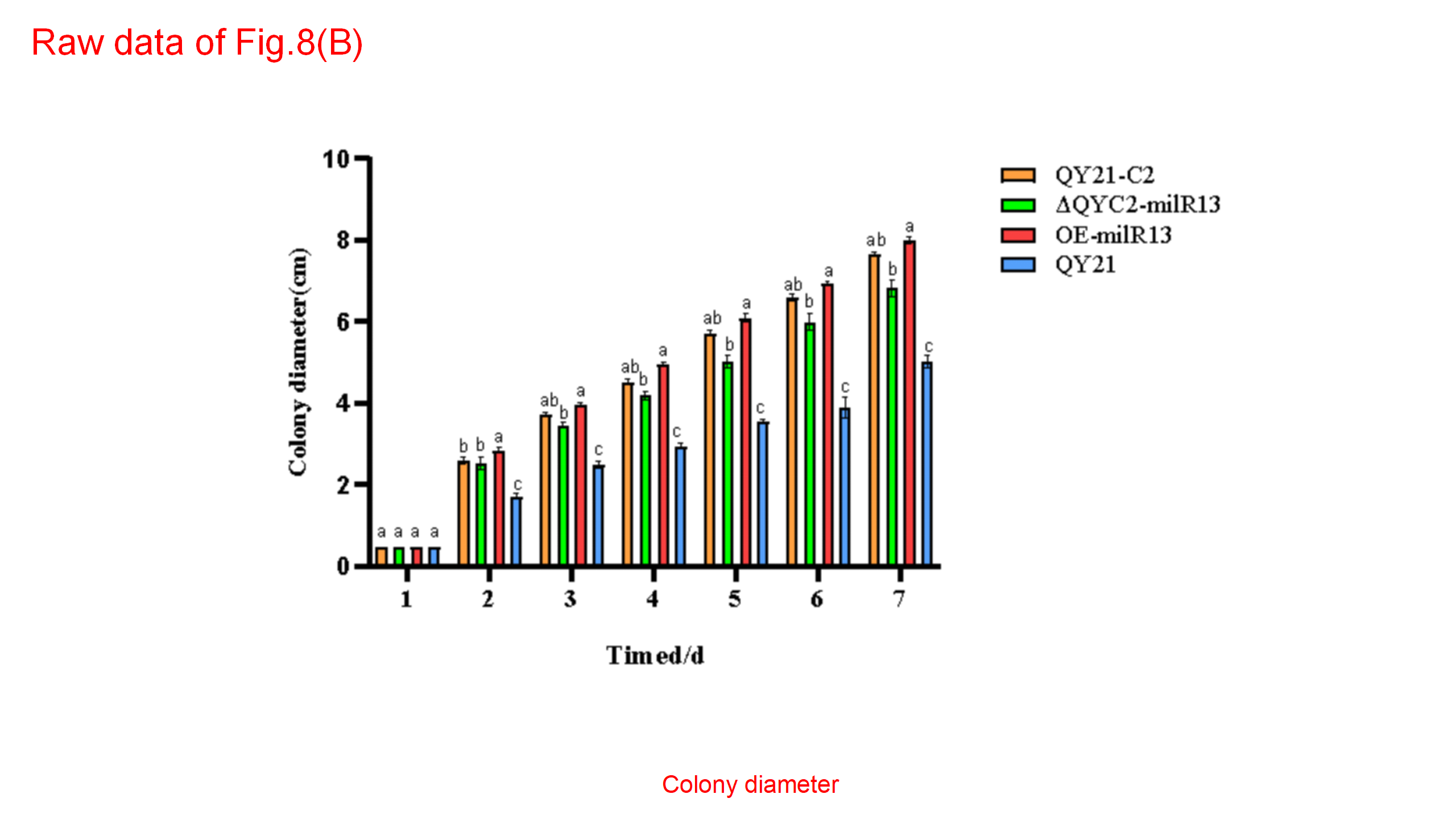

Supplement: Supplementary file 5 [file DataSheet5.zip › New Raw Images Fig8/New Fig.8 (B) Colony diameter.tif]

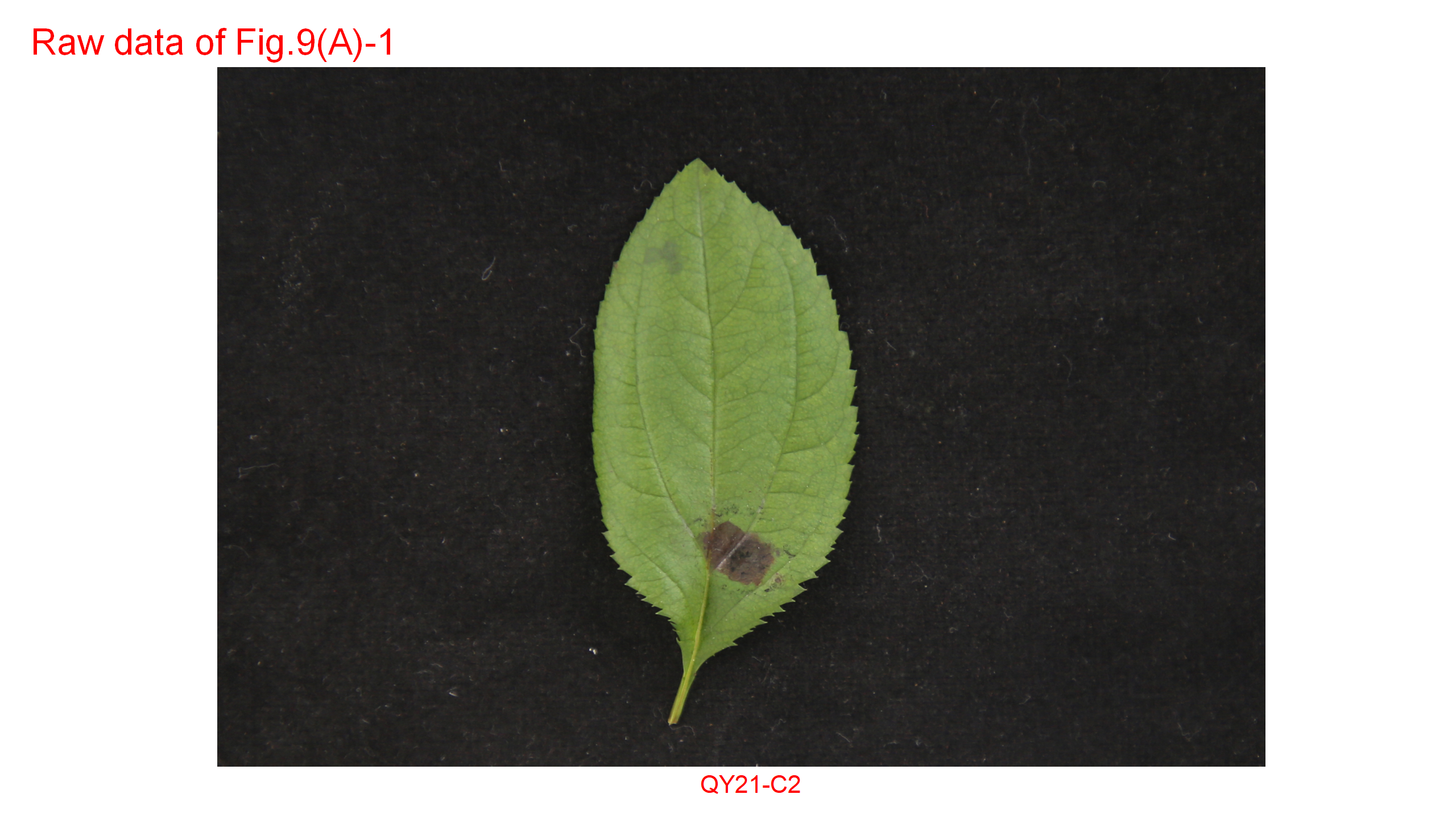

Supplement: Supplementary file 6 [file DataSheet6.zip › New Raw Images Fig9/New Fig.9 (A)-1 QY21-C2 leaf.tif]

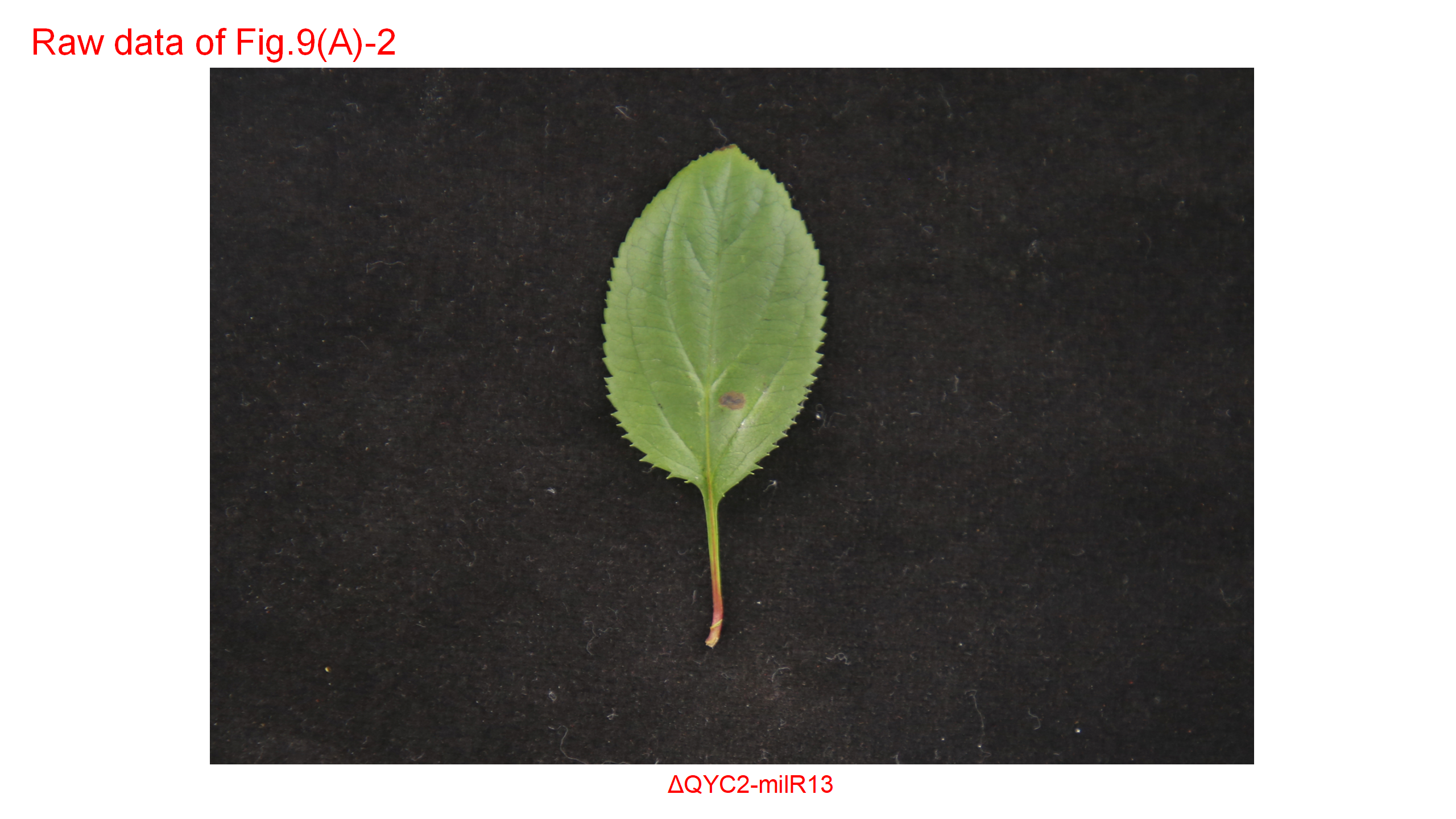

Supplement: Supplementary file 6 [file DataSheet6.zip › New Raw Images Fig9/New Fig.9 (A)-2 ΔQYC2-milR13 leaf.tif]

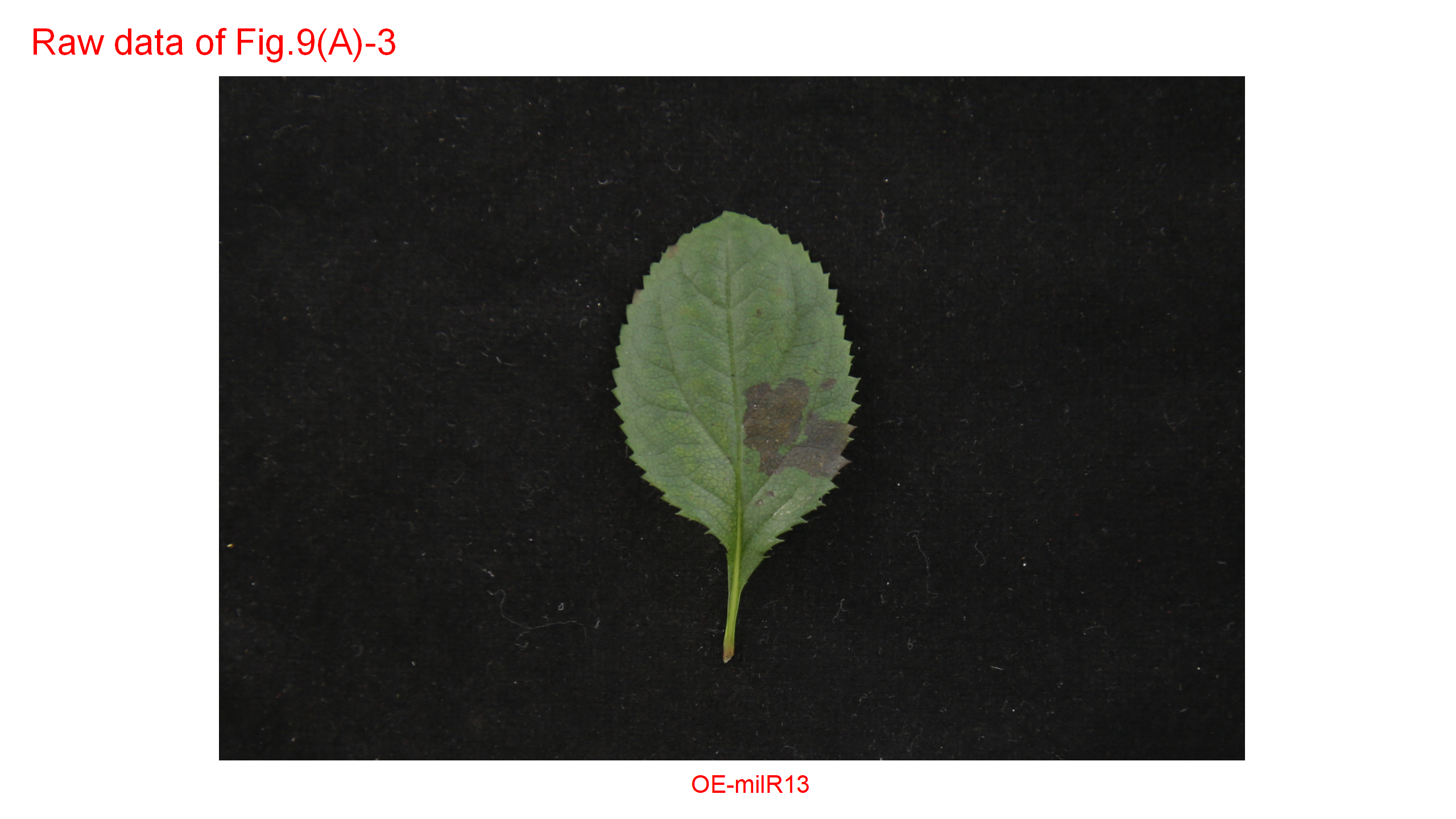

Supplement: Supplementary file 6 [file DataSheet6.zip › New Raw Images Fig9/New Fig.9 (A)-3 OE-milR13 leaf.tif]

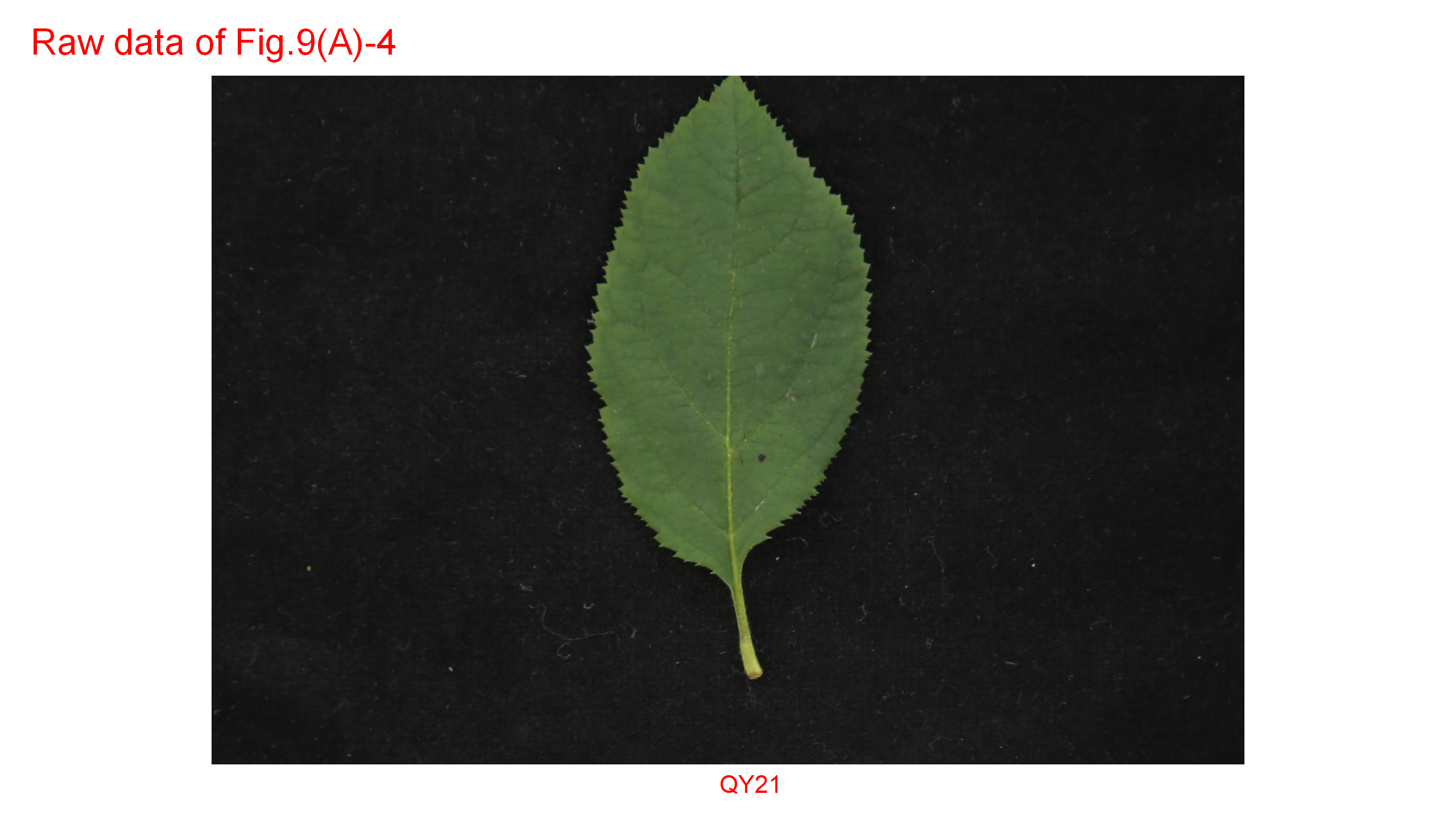

Supplement: Supplementary file 6 [file DataSheet6.zip › New Raw Images Fig9/New Fig.9 (A)-4 QY21 leaf.tif]

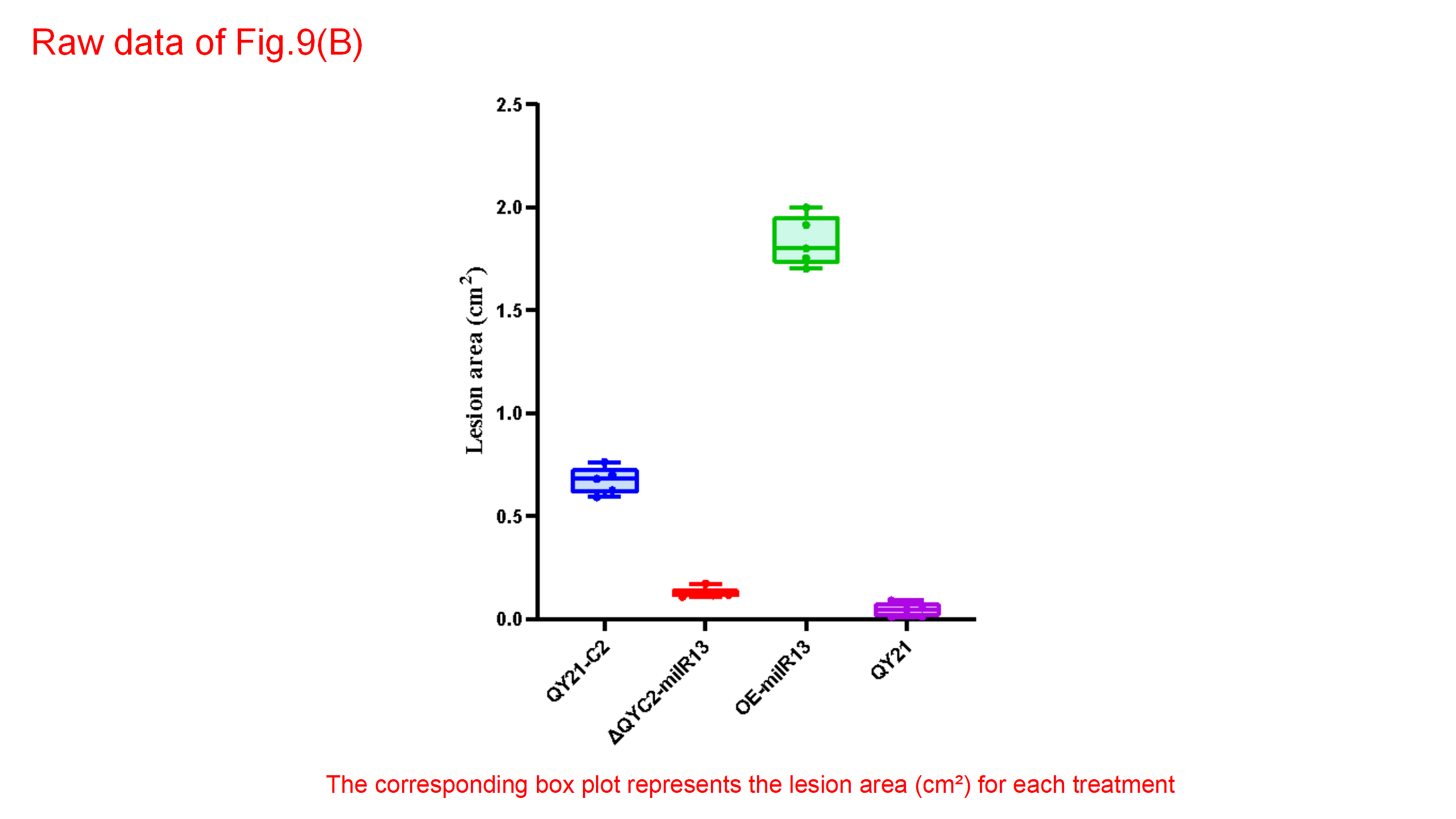

Supplement: Supplementary file 6 [file DataSheet6.zip › New Raw Images Fig9/New Fig.9 (B) The corresponding box plot represents the lesion area (cm2) for each treatment on leaves.tif]

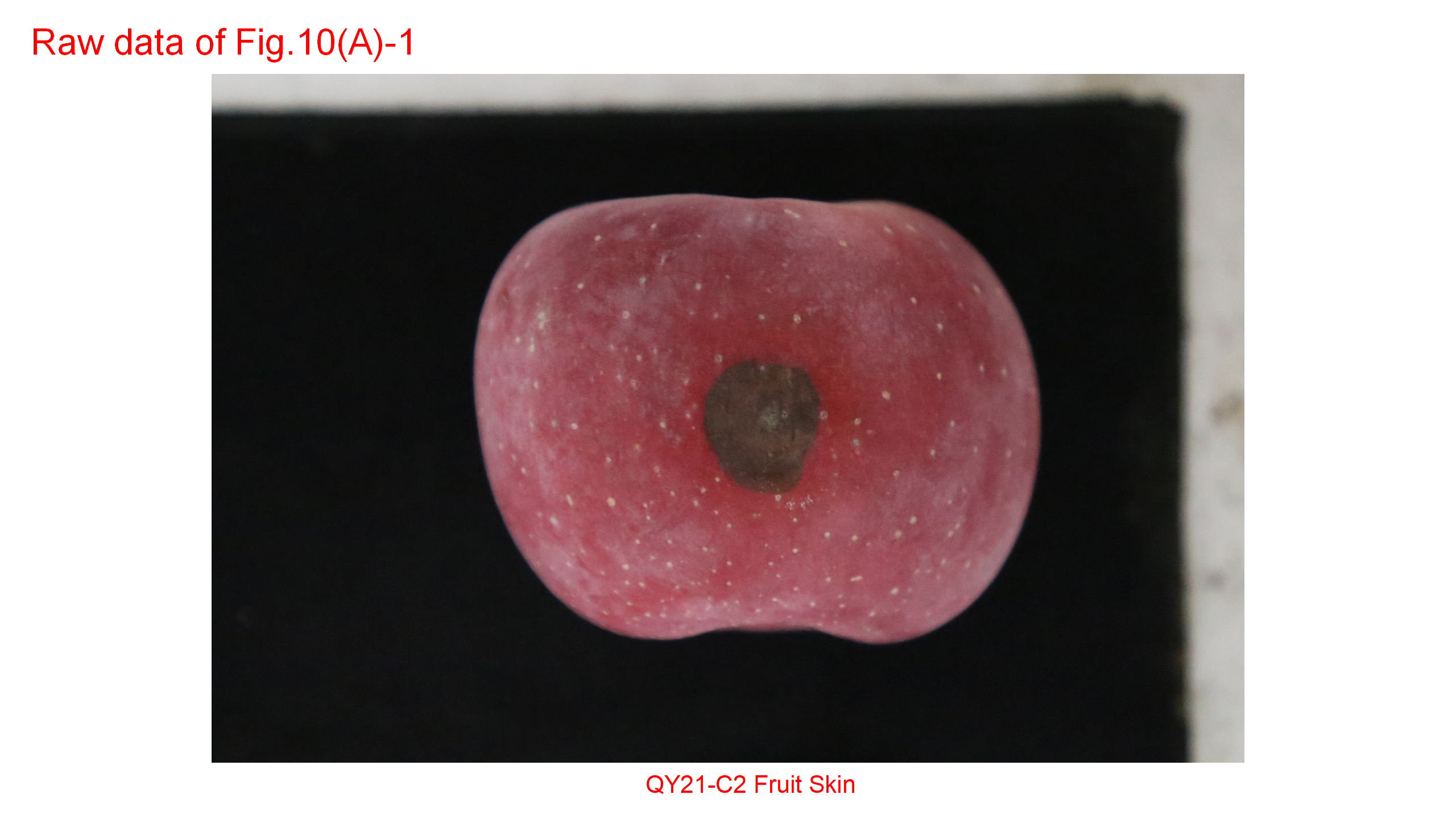

Supplement: Supplementary file 7 [file DataSheet7.zip › New Raw Images Fig10-13/New Fig.10 (A)-1 QY21-C2 Apple Fruit Skin.tif]

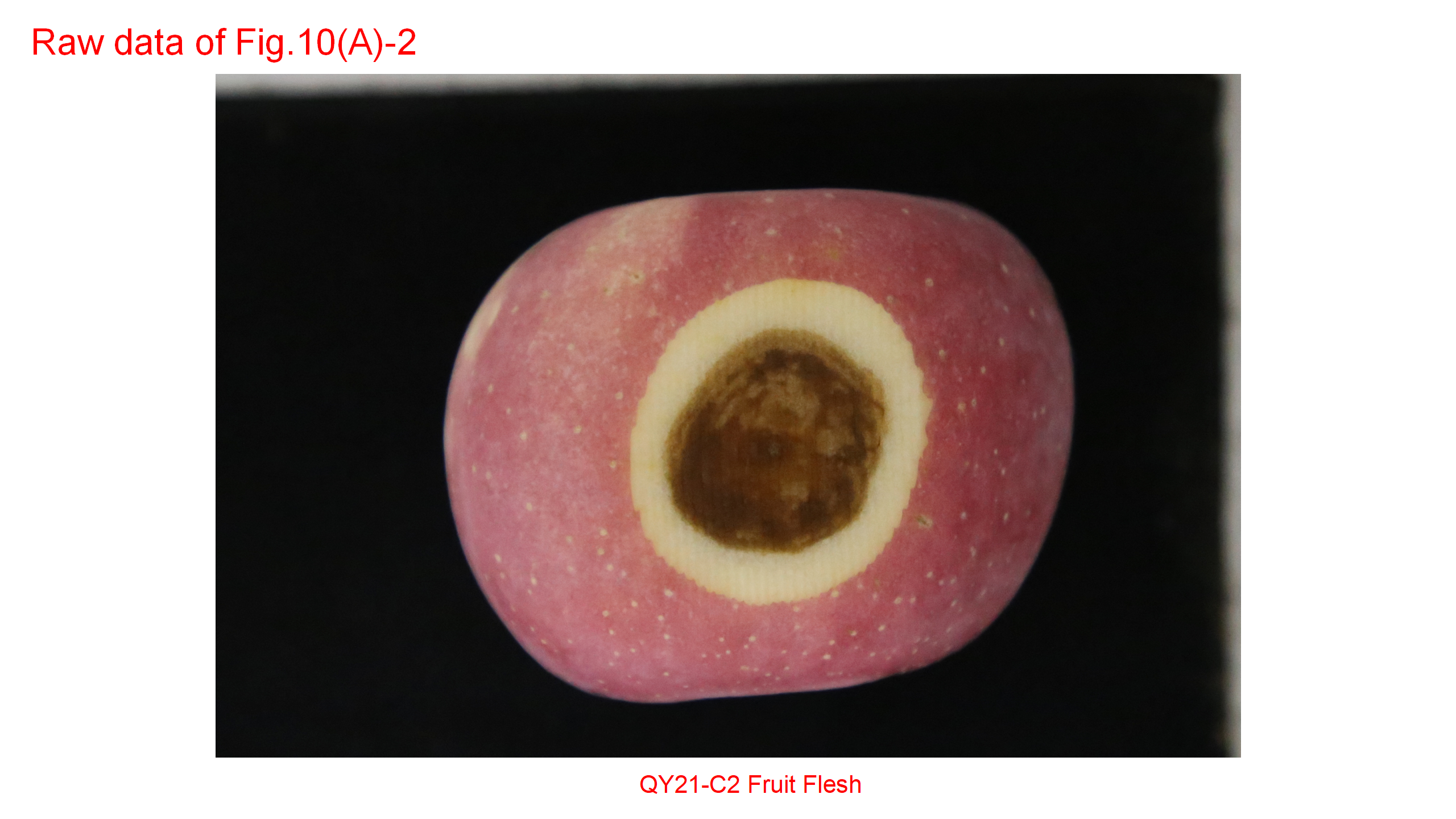

Supplement: Supplementary file 7 [file DataSheet7.zip › New Raw Images Fig10-13/New Fig.10 (A)-2 QY21-C2 Apple Fruit Flesh.tif]

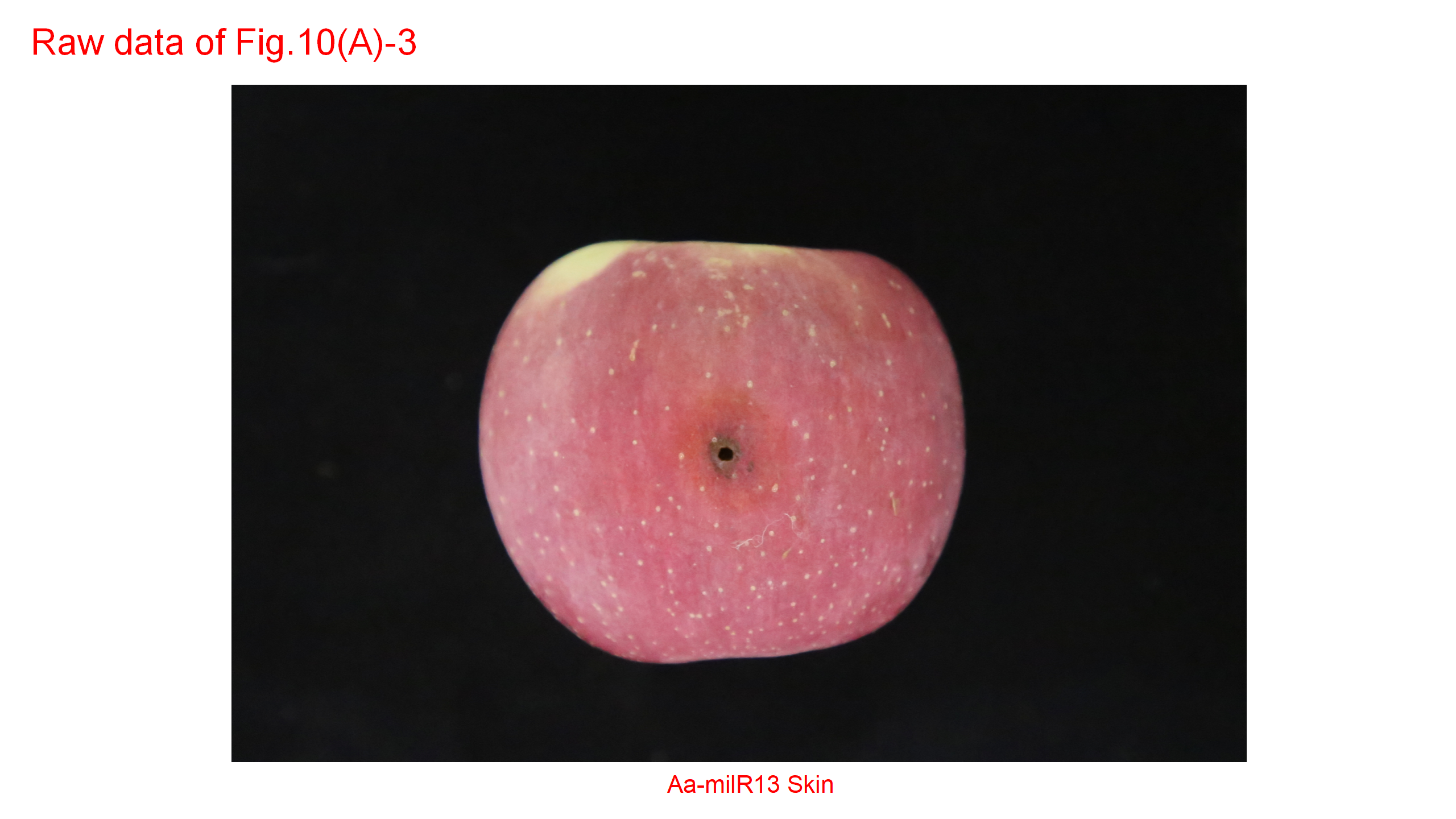

Supplement: Supplementary file 7 [file DataSheet7.zip › New Raw Images Fig10-13/New Fig.10 (A)-3 ΔQYC2-milR13 Apple Fruit Skin.tif]

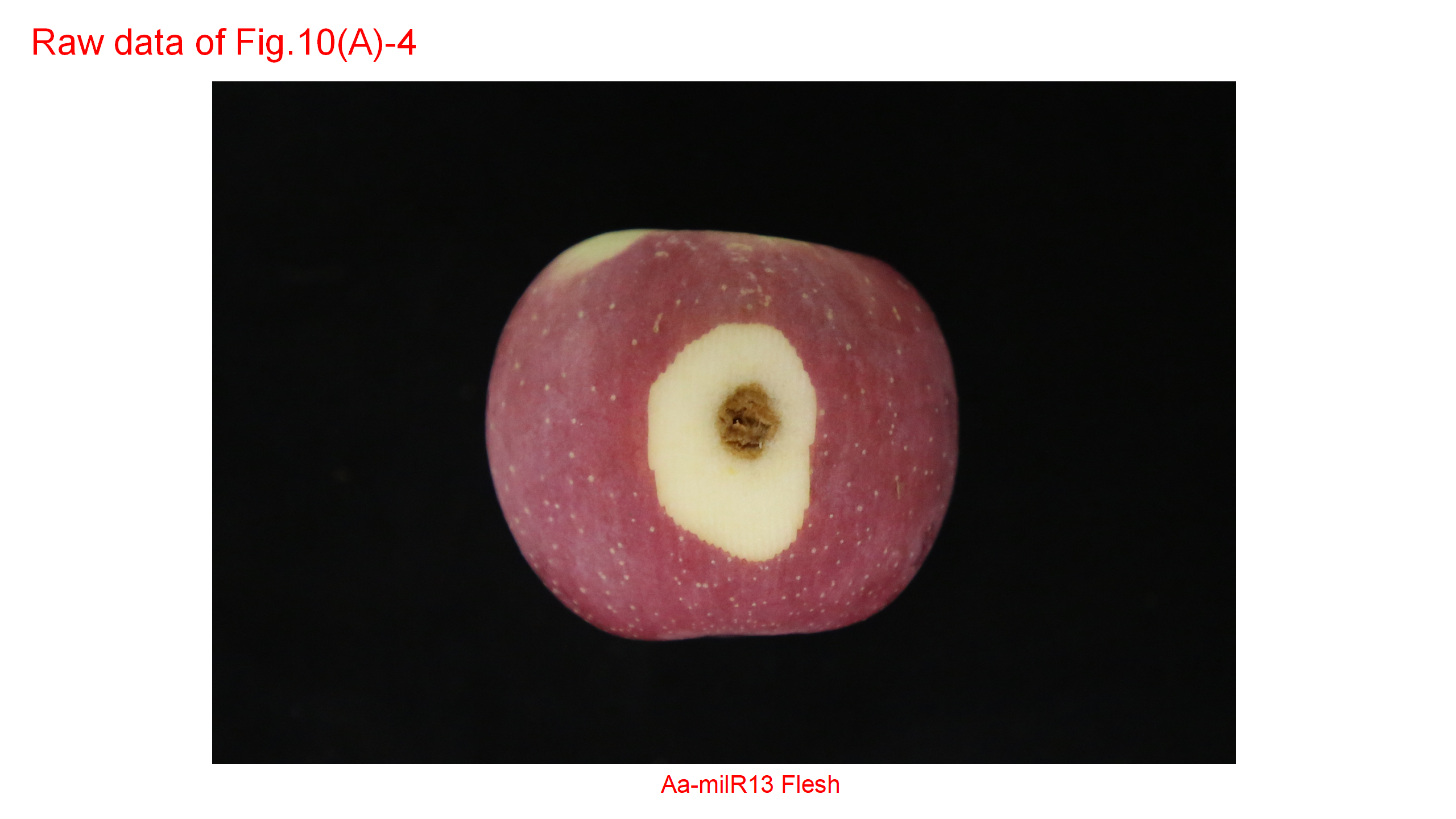

Supplement: Supplementary file 7 [file DataSheet7.zip › New Raw Images Fig10-13/New Fig.10 (A)-4 ΔQYC2-milR13 Apple Fruit Flesh.tif]

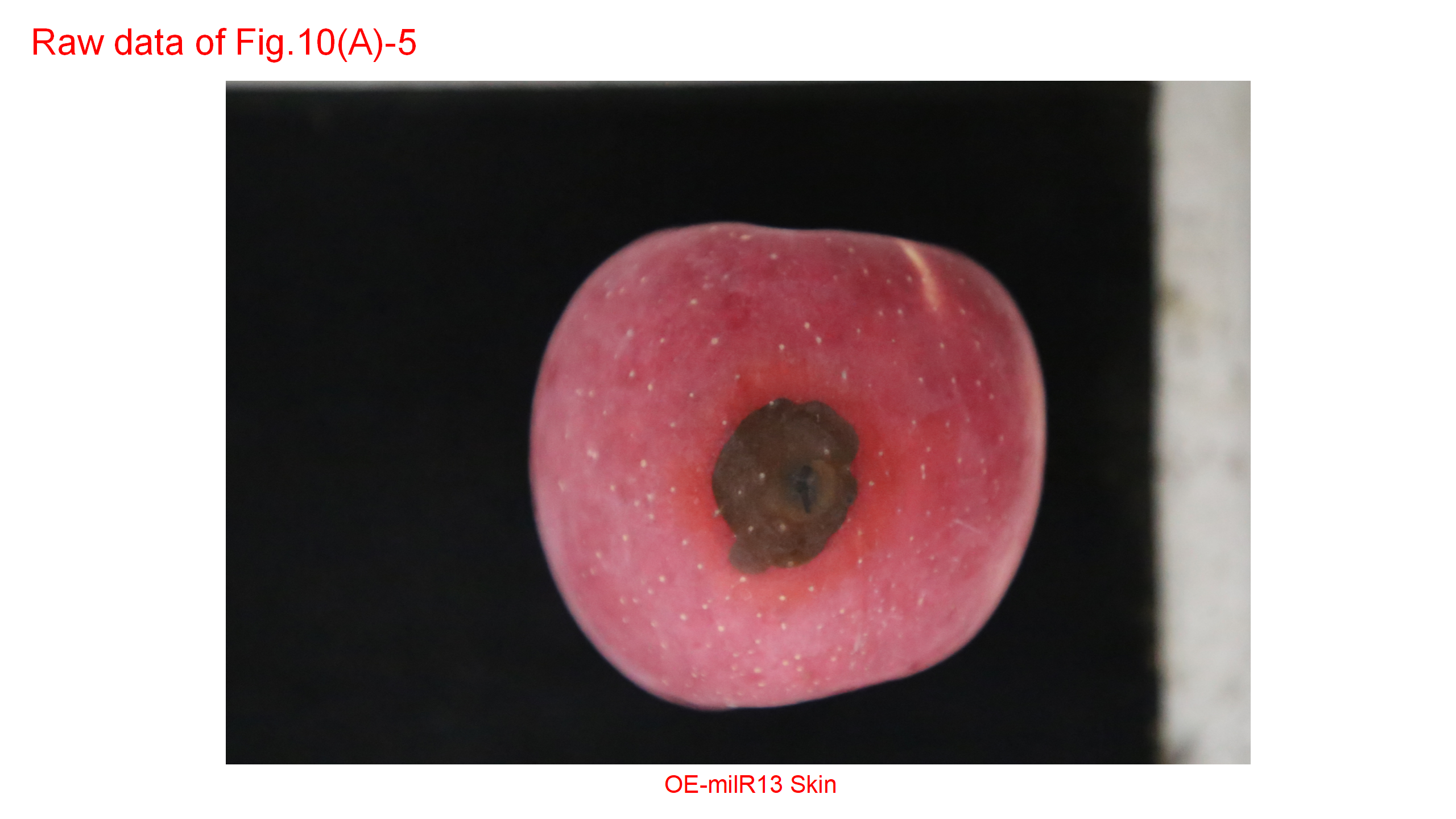

Supplement: Supplementary file 7 [file DataSheet7.zip › New Raw Images Fig10-13/New Fig.10 (A)-5 OE-milR13 Apple Fruit Skin.tif]

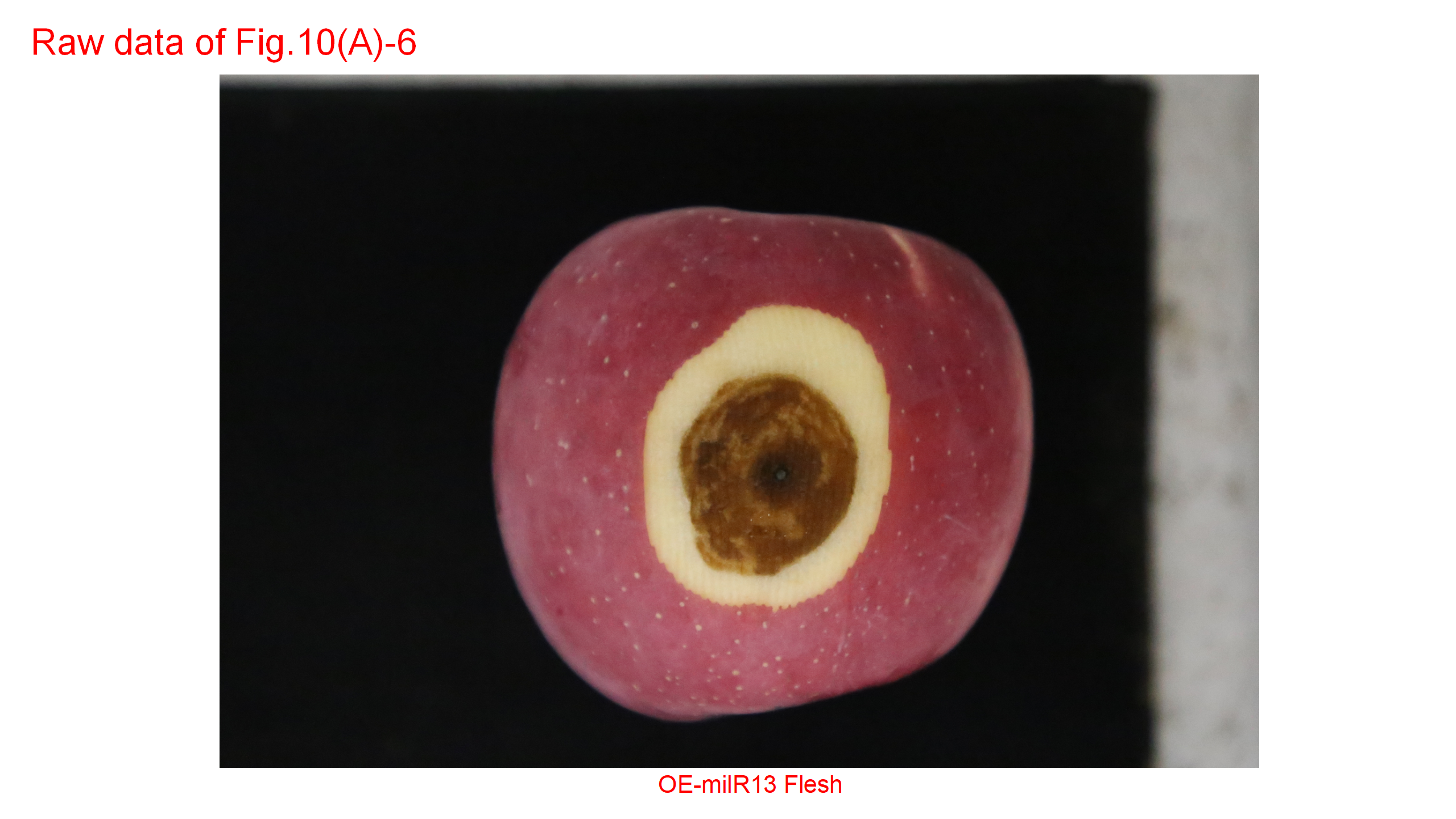

Supplement: Supplementary file 7 [file DataSheet7.zip › New Raw Images Fig10-13/New Fig.10 (A)-6 OE-milR13 Apple Fruit Flesh.tif]

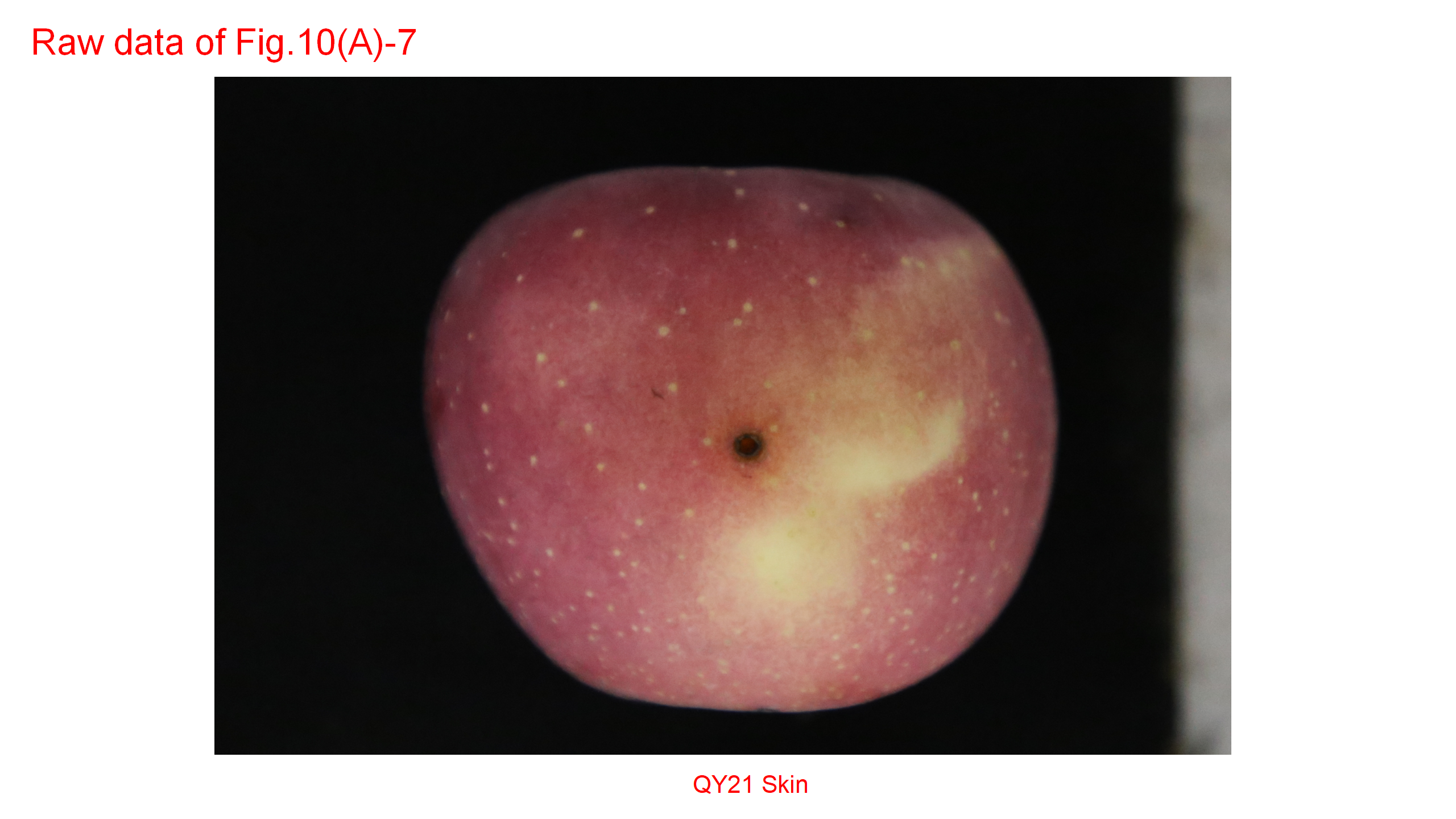

Supplement: Supplementary file 7 [file DataSheet7.zip › New Raw Images Fig10-13/New Fig.10 (A)-7 QY21 Apple Fruit Skin.tif]

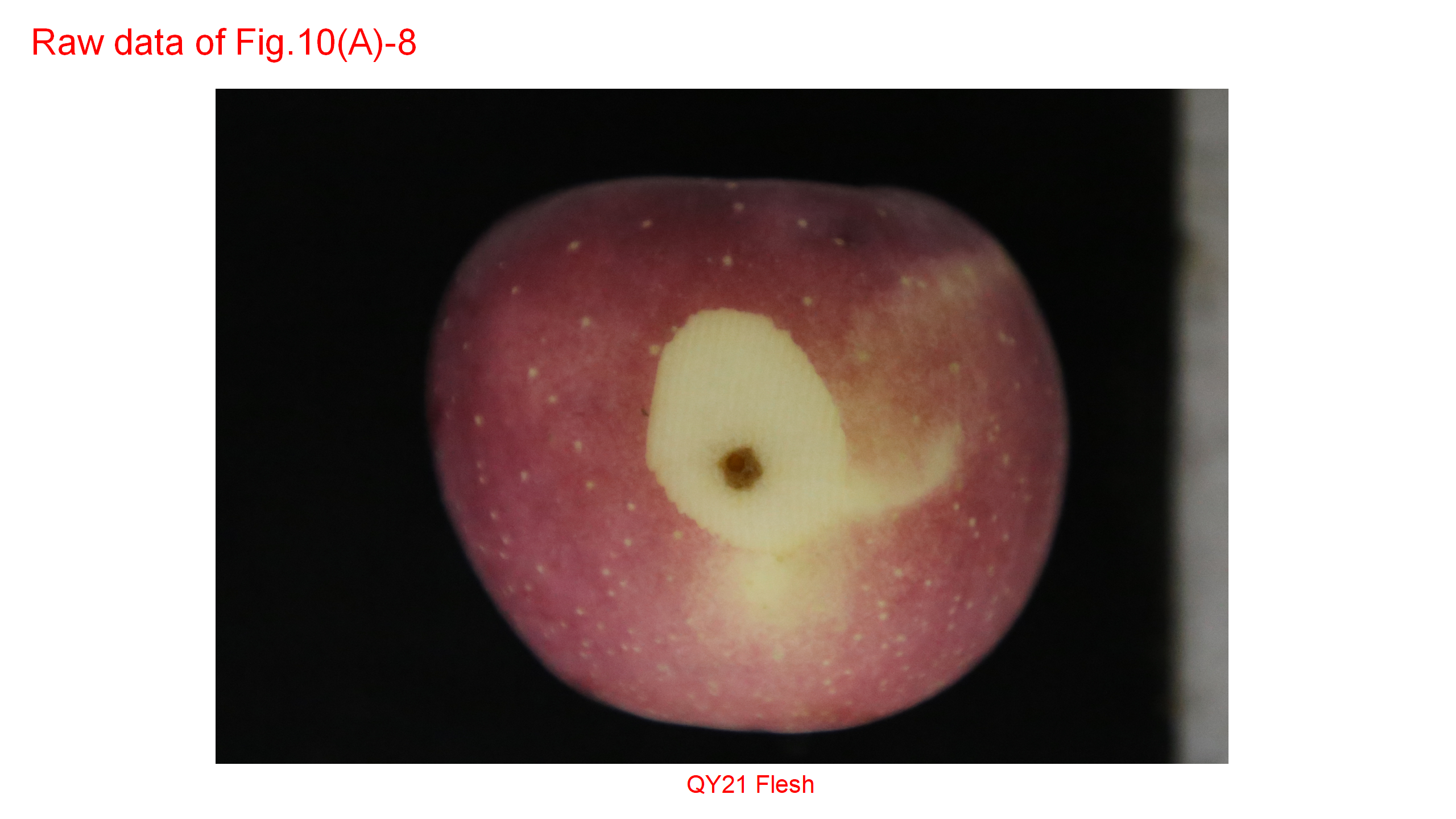

Supplement: Supplementary file 7 [file DataSheet7.zip › New Raw Images Fig10-13/New Fig.10 (A)-8 QY21 Apple Fruit Flesh.tif]

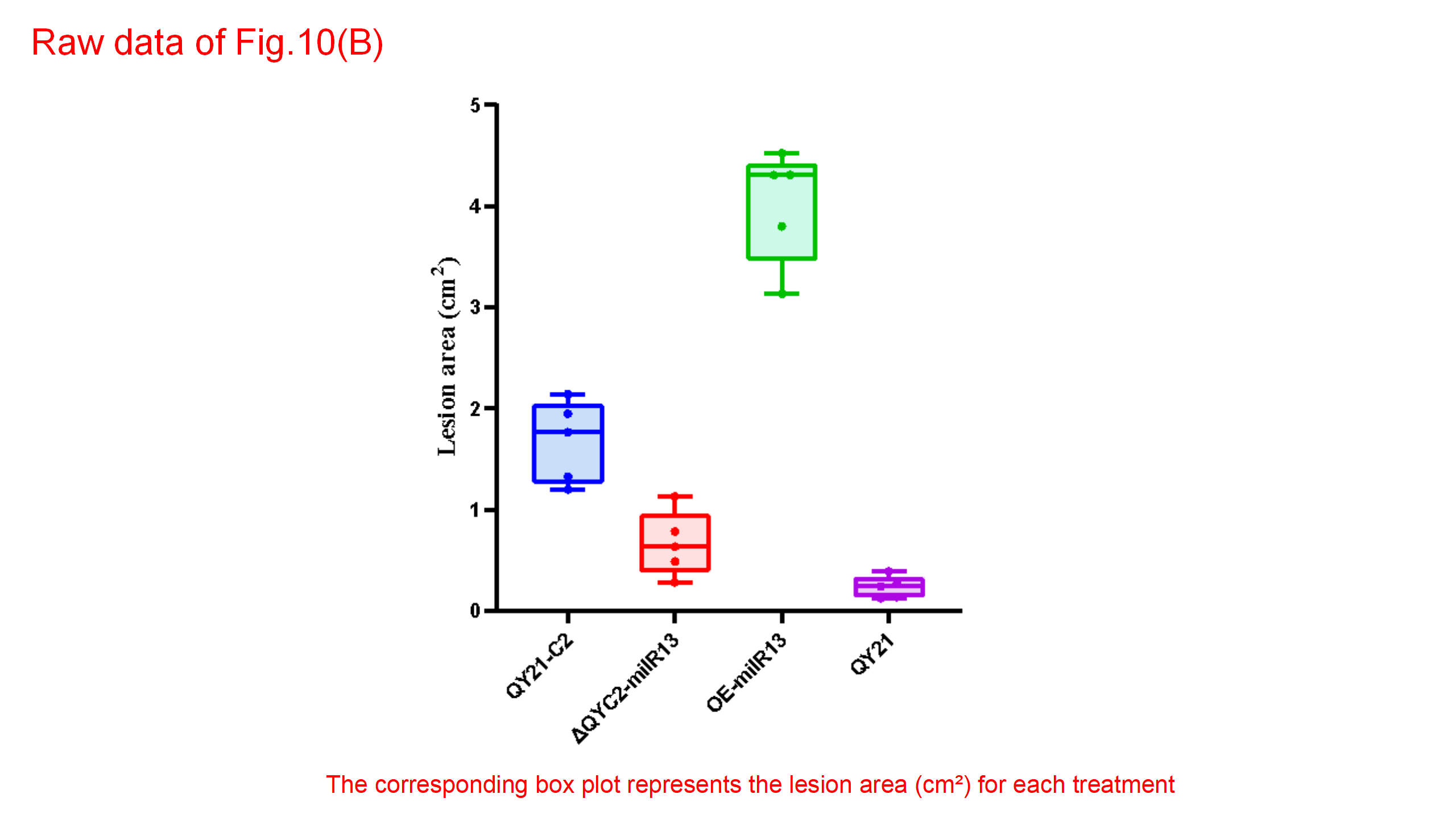

Supplement: Supplementary file 7 [file DataSheet7.zip › New Raw Images Fig10-13/New Fig.10 (B) The corresponding box plot represents the lesion area (cm2) for each treatment in apples.tif]

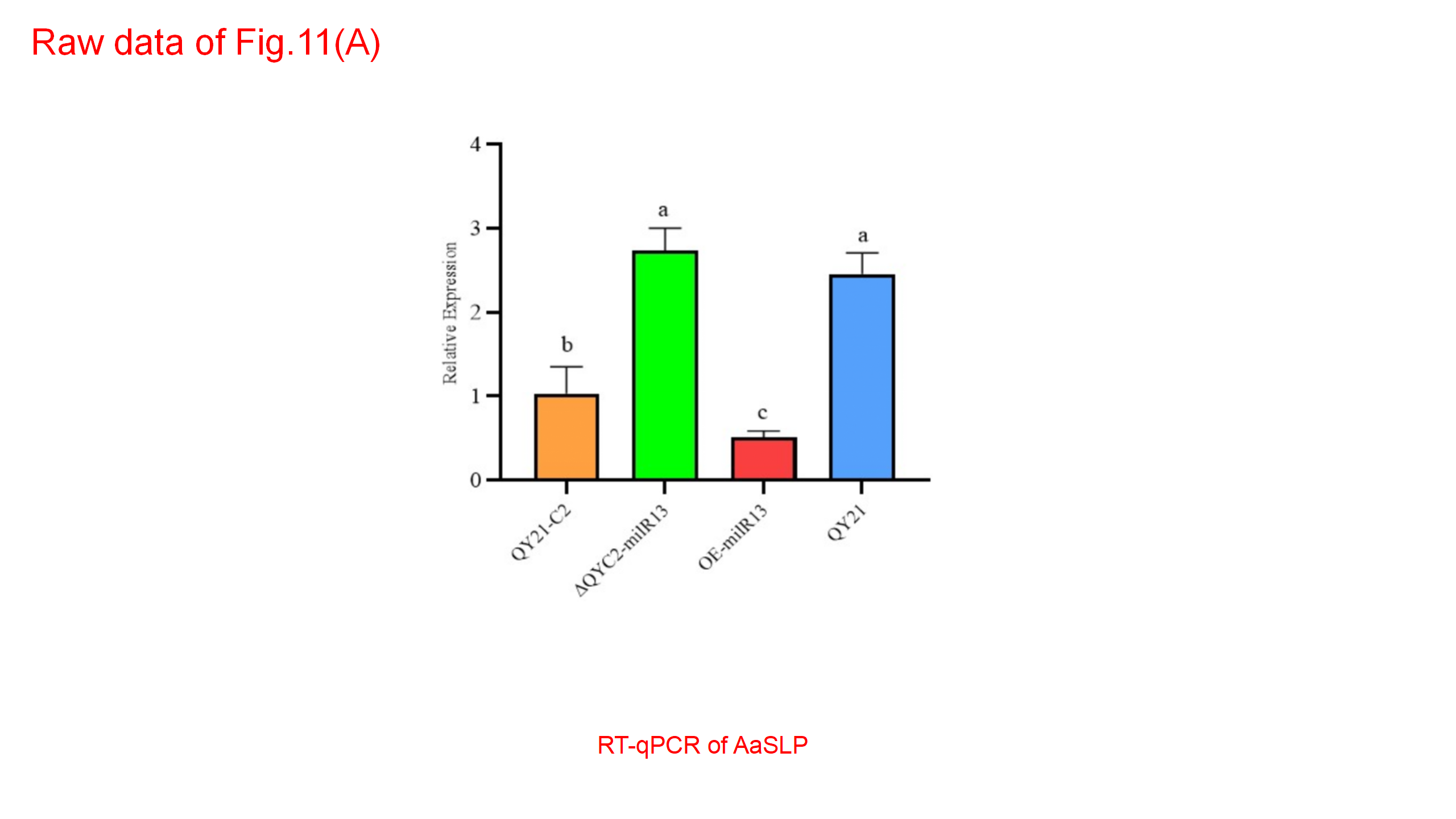

Supplement: Supplementary file 7 [file DataSheet7.zip › New Raw Images Fig10-13/New Fig.11 (A) RT-qPCR of AaSLP.tif]

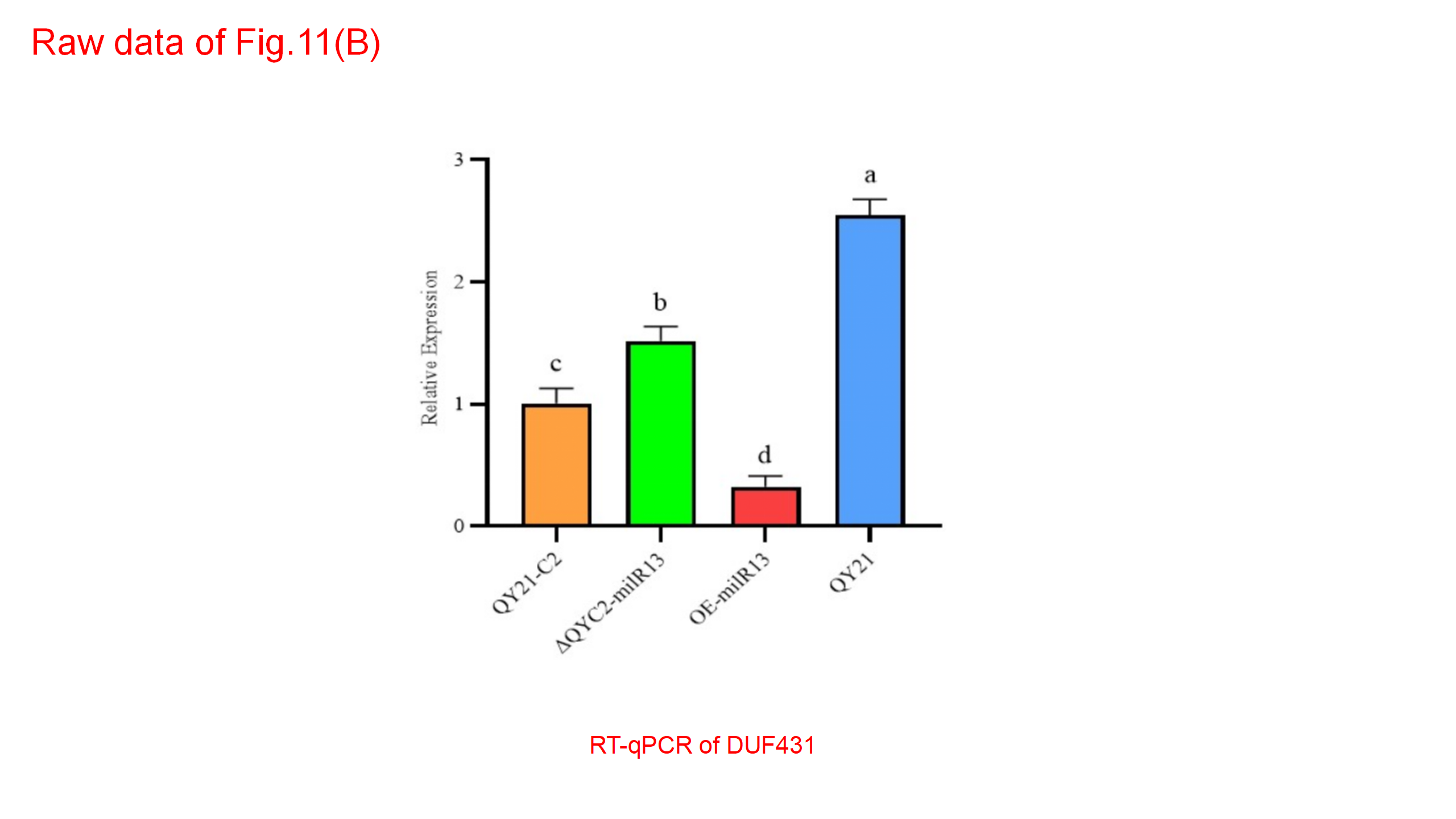

Supplement: Supplementary file 7 [file DataSheet7.zip › New Raw Images Fig10-13/New Fig.11 (B) RT-qPCR of DUF431.tif]

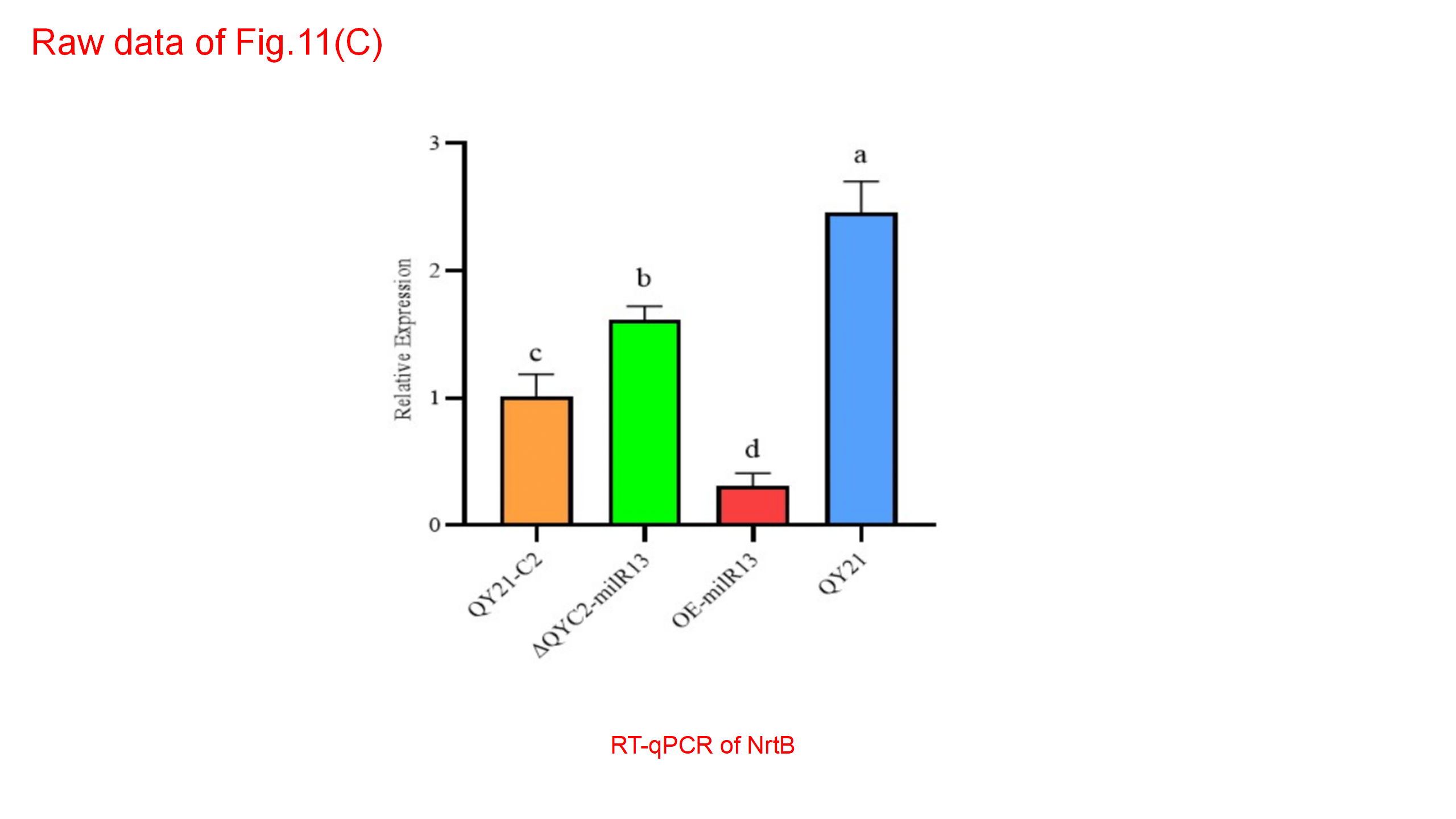

Supplement: Supplementary file 7 [file DataSheet7.zip › New Raw Images Fig10-13/New Fig.11 (C) RT-qPCR of NrtB.tif]

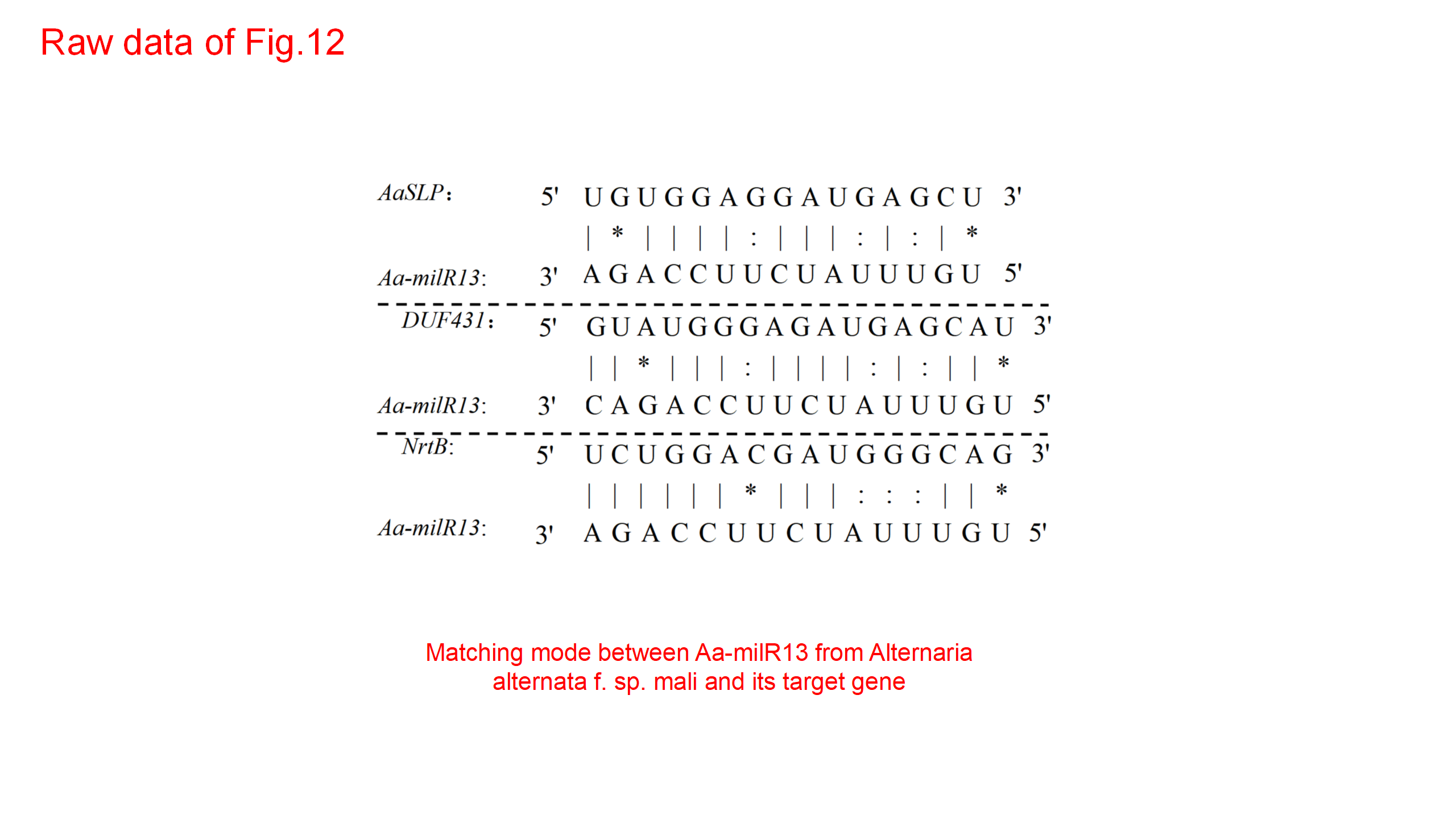

Supplement: Supplementary file 7 [file DataSheet7.zip › New Raw Images Fig10-13/New Fig.12 Matching mode between Aa-milR13 from Alternaria alternata f. sp. mali and its target gene.tif]

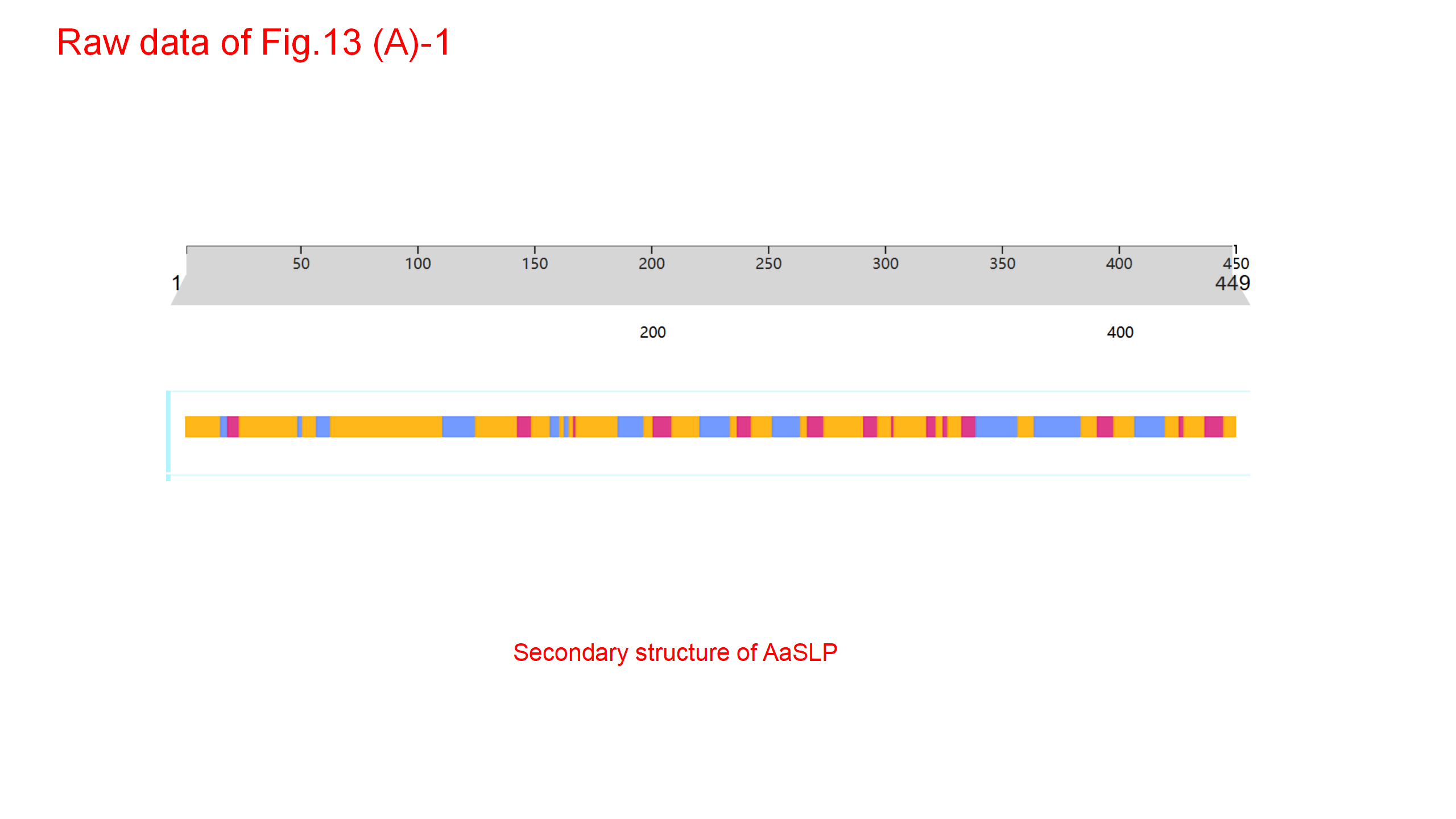

Supplement: Supplementary file 7 [file DataSheet7.zip › New Raw Images Fig10-13/New Fig.13 (A)-1 Secondary structure of AaSLP.tif]

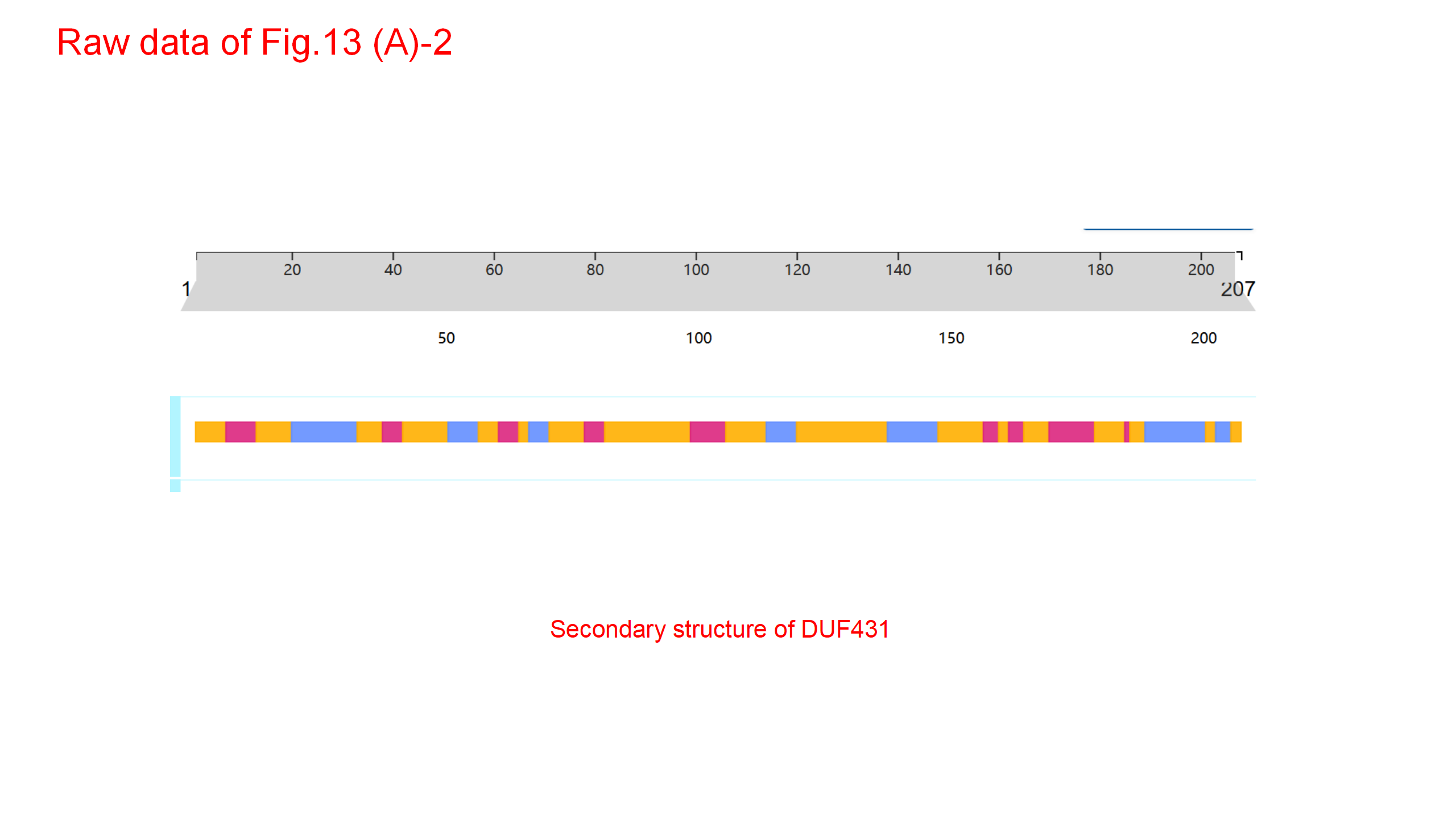

Supplement: Supplementary file 7 [file DataSheet7.zip › New Raw Images Fig10-13/New Fig.13 (A)-2 Secondary structure of DUF431.tif]

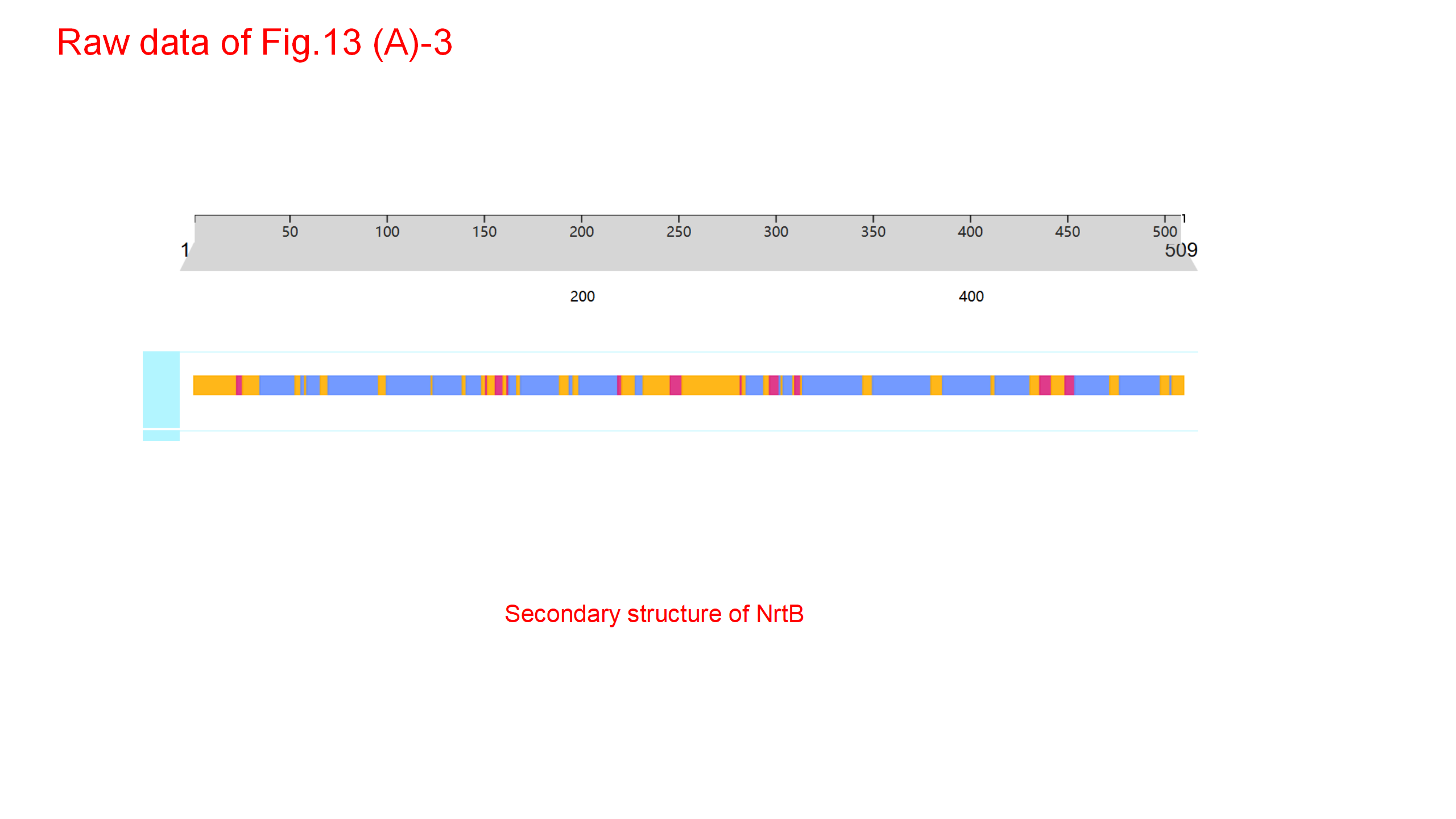

Supplement: Supplementary file 7 [file DataSheet7.zip › New Raw Images Fig10-13/New Fig.13 (A)-3 Secondary structure of NrtB.tif]

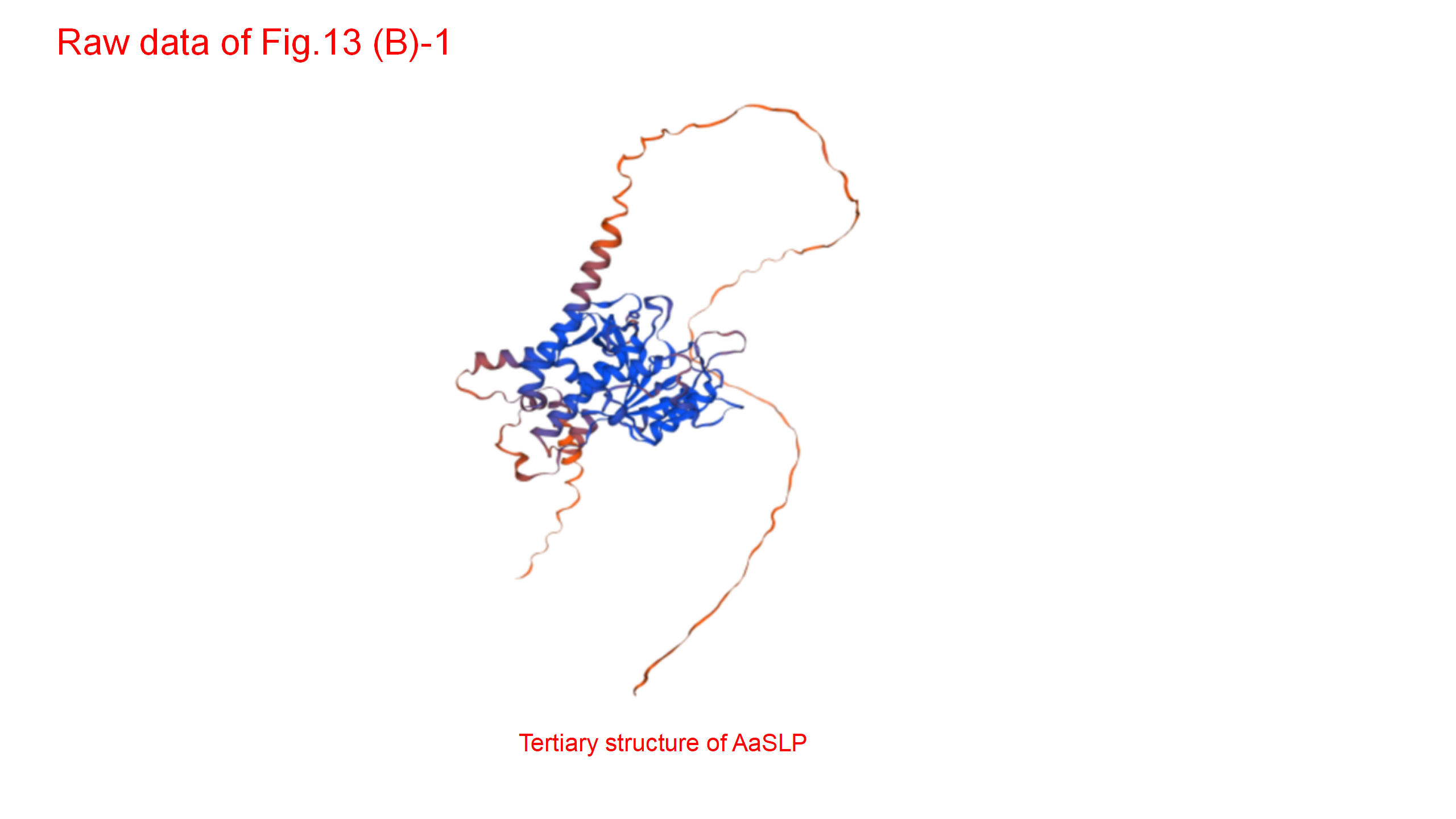

Supplement: Supplementary file 7 [file DataSheet7.zip › New Raw Images Fig10-13/New Fig.13 (B)-1 Tertiary structure of AaSLP.tif]

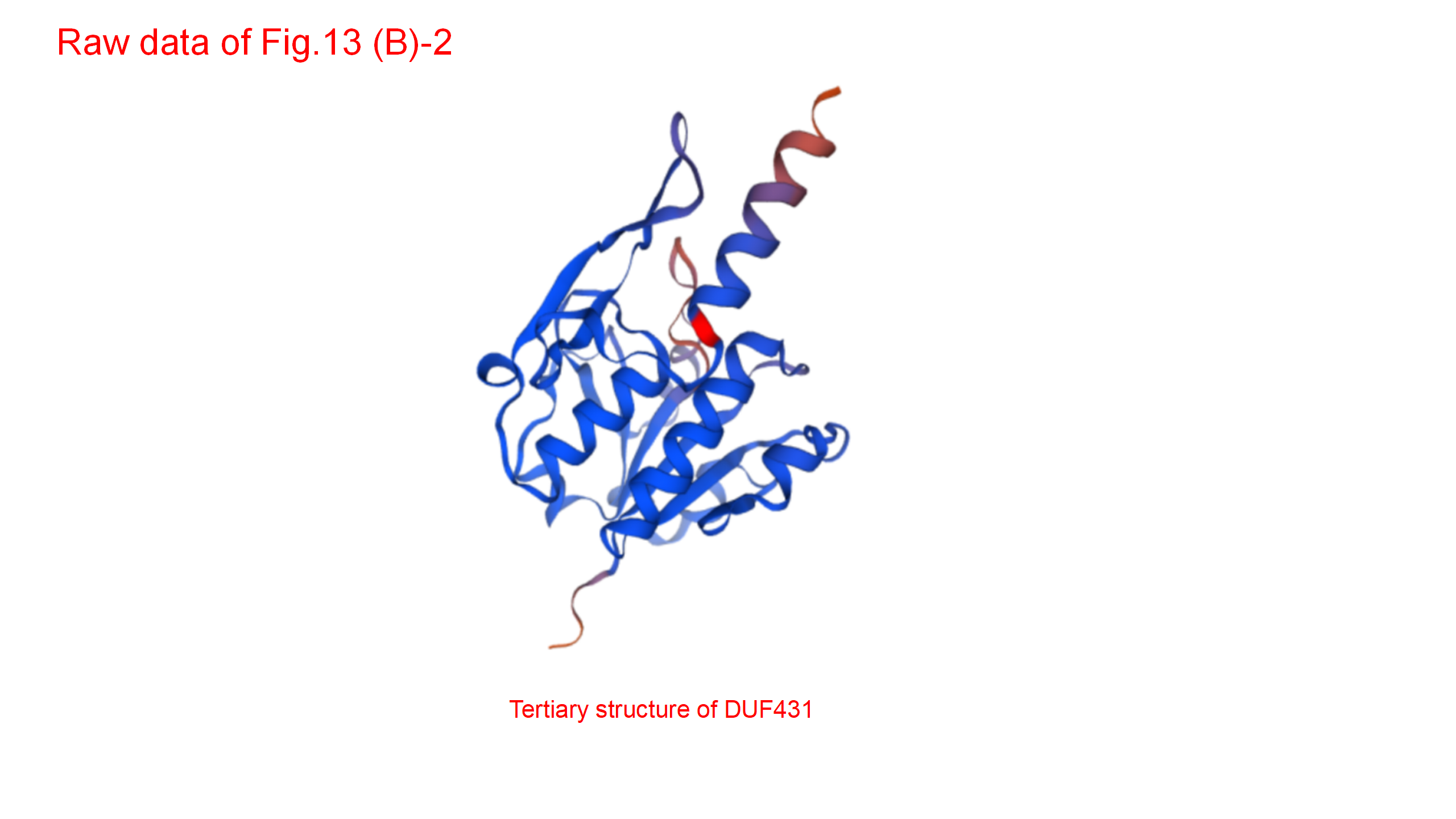

Supplement: Supplementary file 7 [file DataSheet7.zip › New Raw Images Fig10-13/New Fig.13 (B)-2 Tertiary structure of DUF431.tif]

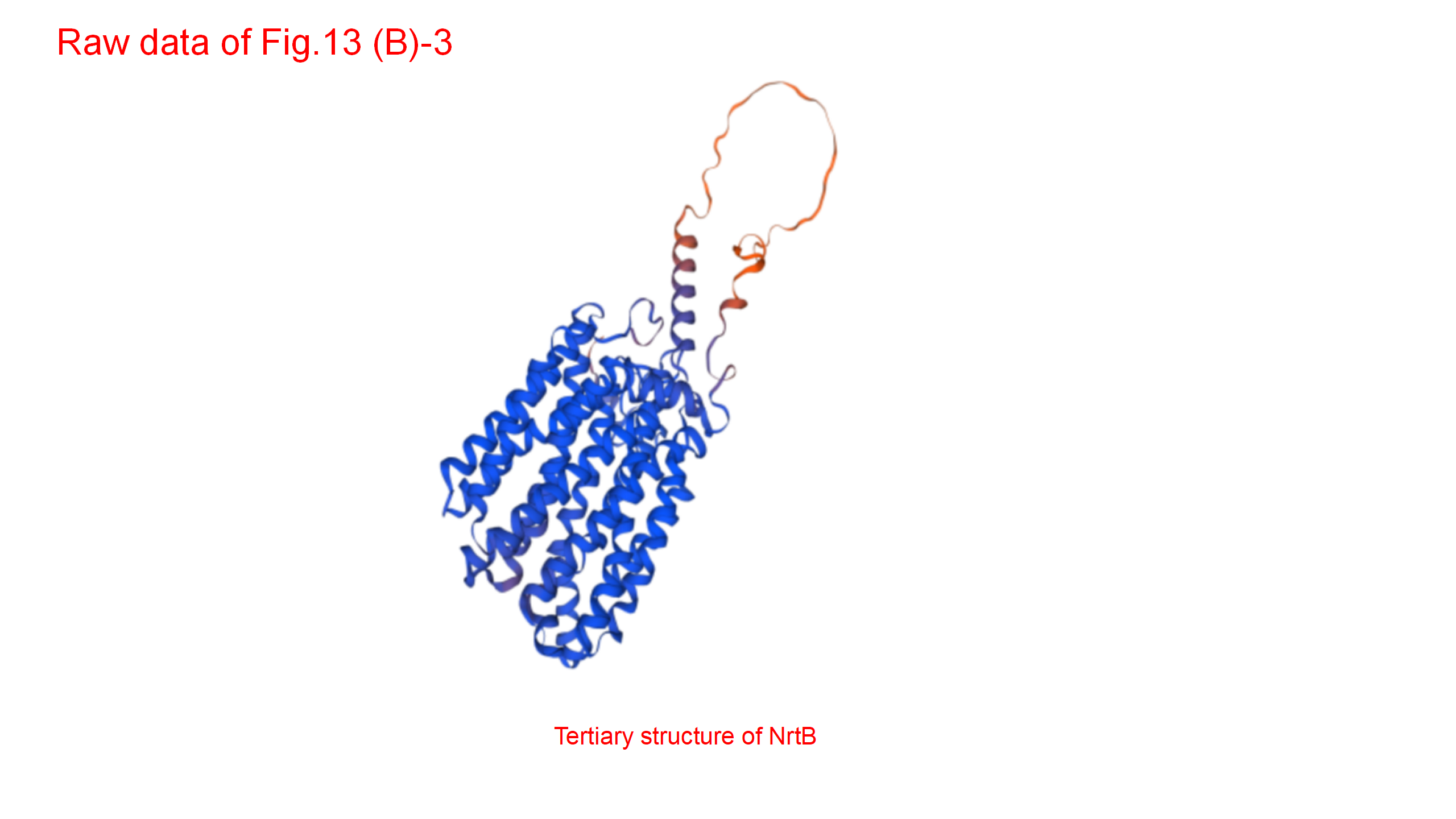

Supplement: Supplementary file 7 [file DataSheet7.zip › New Raw Images Fig10-13/New Fig.13 (B)-3 Tertiary structure of NrtB.tif]
